# Supplementary material for: Addressing Vaccine Hesitancy Through a Comprehensive Resident Vaccine Curriculum
Source: MedEdPORTAL. 2022 Dec 27;18:11292. doi: 10.15766/mep_2374-8265.11292 (PMC9792628; doi:10.15766/mep_2374-8265.11292)
Supplement: Supplementary file 1 — Vaccine Curriculum Facilitator Guide.docxVaccines Part 1.pptxVaccines Part 2.pptxVaccines Part 3 - Myths and Facts.pptxVaccines Part 4 - Communication Skills.pptxVaccine Hesitancy Communication Cases.docxVaccine Pretest.docxVaccine Posttest.docxPre- and Posttest Answer Key.docxSP Case and Notes for SP.docxSP Case Development Tool.docxSP Case - Learner Version.docxSP Assessment Checklist.docx [file mep_2374-8265.11292-s001.zip › A. Vaccine Curriculum Facilitator Guide.docx]

Vaccine Curriculum – Facilitator’s Guide:

The vaccine curriculum is divided into four sessions, each approximately 40 minutes (may take longer depending on amount of discussion generated). In our curriculum, four sessions were given over the course of 10 months to accommodate each clinic sessions’ unique schedules. The time between sessions varied by clinic. Please note, the presentations below have been slightly modified since initial implementation and evaluation of the curriculum, to account for updates such as modified vaccine schedule recommendations.

**Session 1 (Appendix B): Vaccines, Part 1 (PowerPoint presentation with interactive discussion)**

**Session 2 (Appendix C): Vaccines, Part 2 (PowerPoint presentation with interactive discussion)**

**Session 3 (Appendix D): Vaccine Myths and Facts (PowerPoint presentation with interactive discussion)**

For sessions 1-3, facilitators should use the words and information on the power point slides to guide the didactic talk and interactive discussion. All pertinent information is included on the slides with some additional commentary in the “notes” section. Facilitators may vary in the desired amount of audience/learner participation. In our experience, asking for audience participation throughout the talk creates a more engaging learning environment. One example may include asking the group if anyone knows what the potential side effects of the varicella vaccine are just prior to advancing the slide with that information on it. Another example of audience involvement may include asking the learners if anyone has had a patient experiencing side of effects of vaccines and how they counseled them. The Vaccine Myths and Facts presentation is designed in question/answer format to encourage more interactive discussion. To stimulate additional audience engagement, each slide deck includes a brief interactive “quiz” at the end.

**Session 4 (Appendix E): Vaccine Communication Skills (PowerPoint slides and role play cases)**

For session 4, the bulk of the didactic portion (information about communication skills in vaccine hesitancy) is included on the slides, with additional information again included in the “notes” section.

After the didactic portion is complete (~10-15 minutes), the bulk of the session is dedicated to role playing. We created three case vignettes consisting of common vaccine concerns and questions (included in document entitled “Communication Skills Cases”, or Appendix F). Facilitators may choose to break learners up into pairs or small groups, or choose to have two learners role play in front of the whole audience – this may depend on group size and comfort of the learners. We selected two learners to role play each vignette – for each vignette, one learner played the part of the parent and one learner played the part of the physician. After approximately 5 minutes of role play, the facilitator led a discussion about which communication skills techniques were used, what went well, what was challenging and what could be done differently the next time. Time-permitting, role playing was repeated after feedback was given.

This could also be done by breaking the large group into three smaller groups of 2-3 learners per small group. Each small group would take one case vignette and have 5 minutes to role play, with one learner playing the part of the parent and one learner playing the part of the physician. If there is an additional learner in the group, they can be given the role of observer/reported. After 5 minutes, learners should discuss with their small groups for 2 minutes, after which the large group reconvenes. Each small group then described their case, which communication techniques were used, what went well, what was challenging, and what could be done differently next time.
