# Supplementary material for: Addressing Vaccine Hesitancy Through a Comprehensive Resident Vaccine Curriculum
Source: MedEdPORTAL. 2022 Dec 27;18:11292. doi: 10.15766/mep_2374-8265.11292 (PMC9792628; doi:10.15766/mep_2374-8265.11292)
Supplement: Supplementary file 1 — Vaccine Curriculum Facilitator Guide.docxVaccines Part 1.pptxVaccines Part 2.pptxVaccines Part 3 - Myths and Facts.pptxVaccines Part 4 - Communication Skills.pptxVaccine Hesitancy Communication Cases.docxVaccine Pretest.docxVaccine Posttest.docxPre- and Posttest Answer Key.docxSP Case and Notes for SP.docxSP Case Development Tool.docxSP Case - Learner Version.docxSP Assessment Checklist.docx [file mep_2374-8265.11292-s001.zip › B. Vaccines Part 1.pptx]

## Slide 1
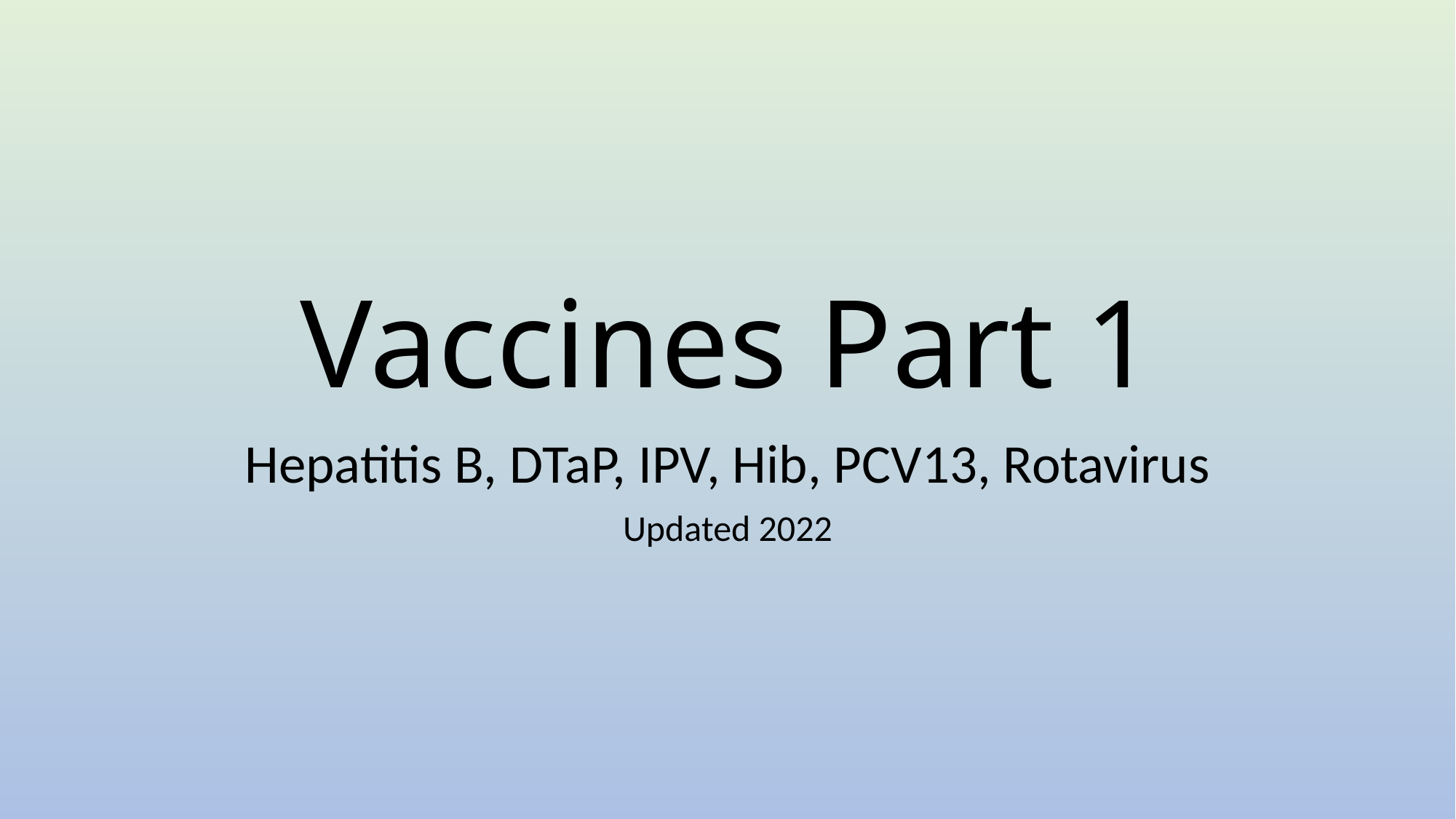

# Vaccines Part 1
Hepatitis B, DTaP, IPV, Hib, PCV13, Rotavirus
Updated 2022

## Slide 2
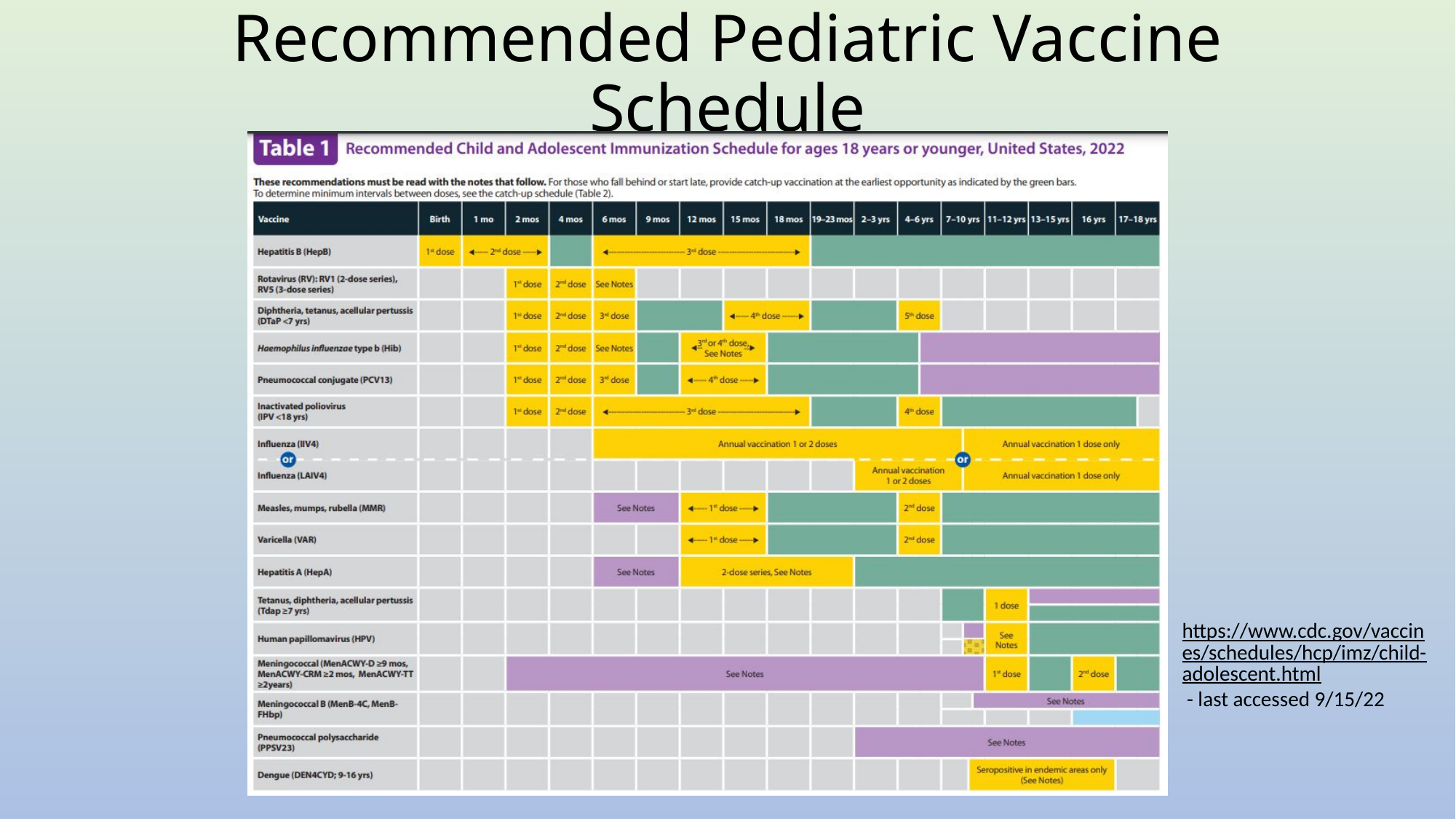

# Recommended Pediatric Vaccine Schedule
https://www.cdc.gov/vaccines/schedules/hcp/imz/child-adolescent.html - last accessed 9/15/22

## Slide 3
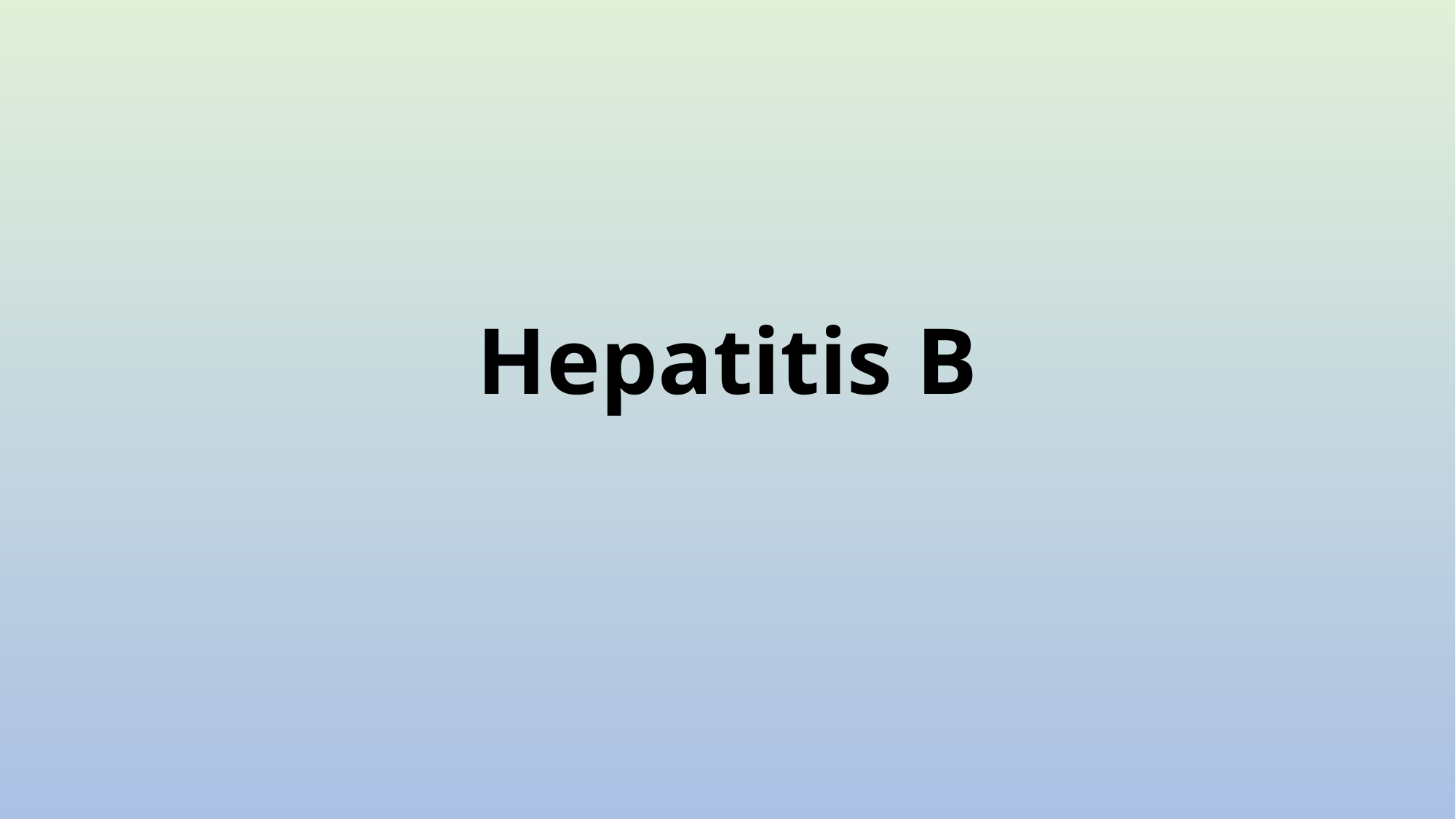

# Hepatitis B

## Slide 4
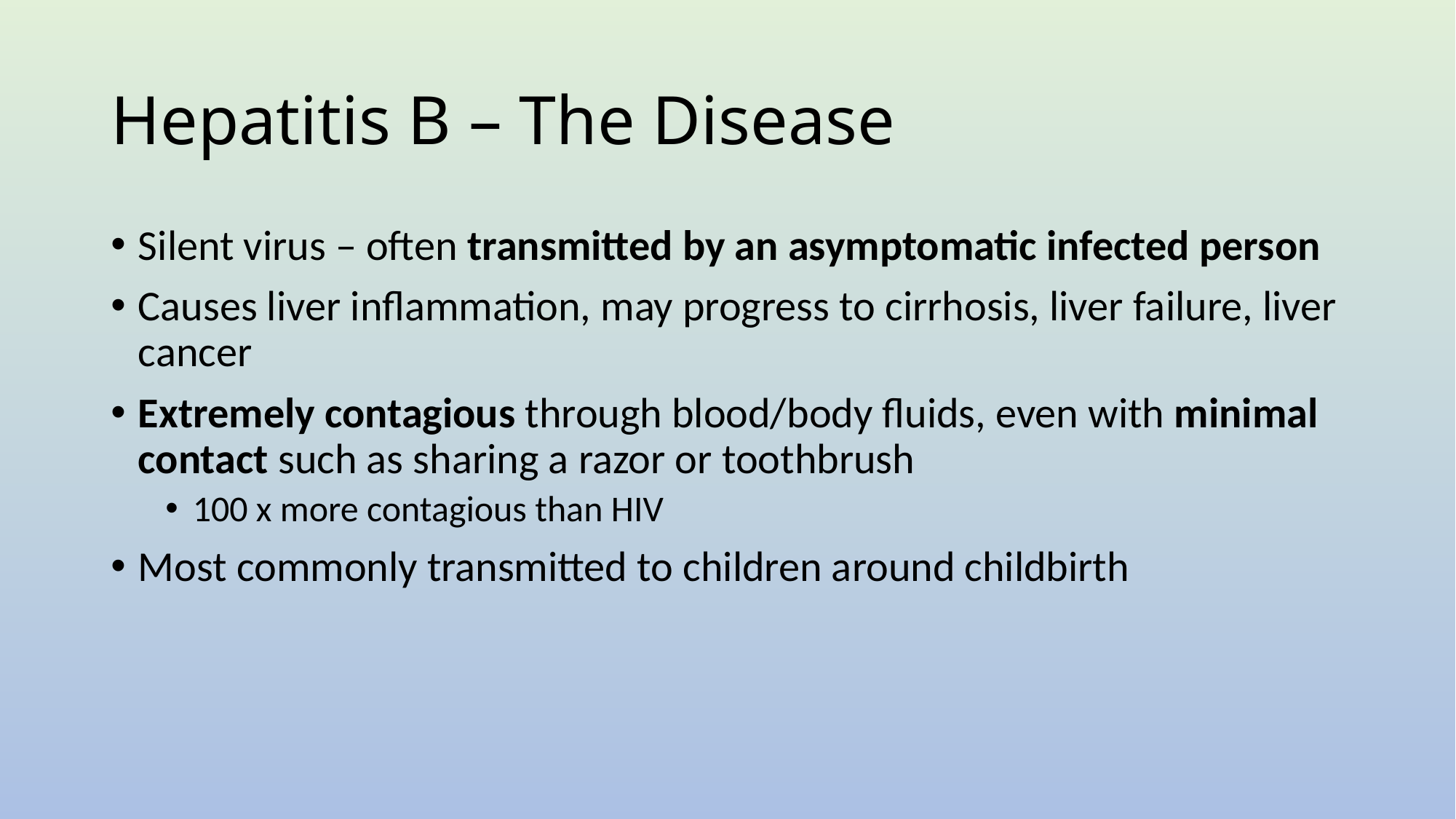

# Hepatitis B – The Disease
Silent virus – often transmitted by an asymptomatic infected person
Causes liver inflammation, may progress to cirrhosis, liver failure, liver cancer
Extremely contagious through blood/body fluids, even with minimal contact such as sharing a razor or toothbrush
100 x more contagious than HIV
Most commonly transmitted to children around childbirth

## Slide 5
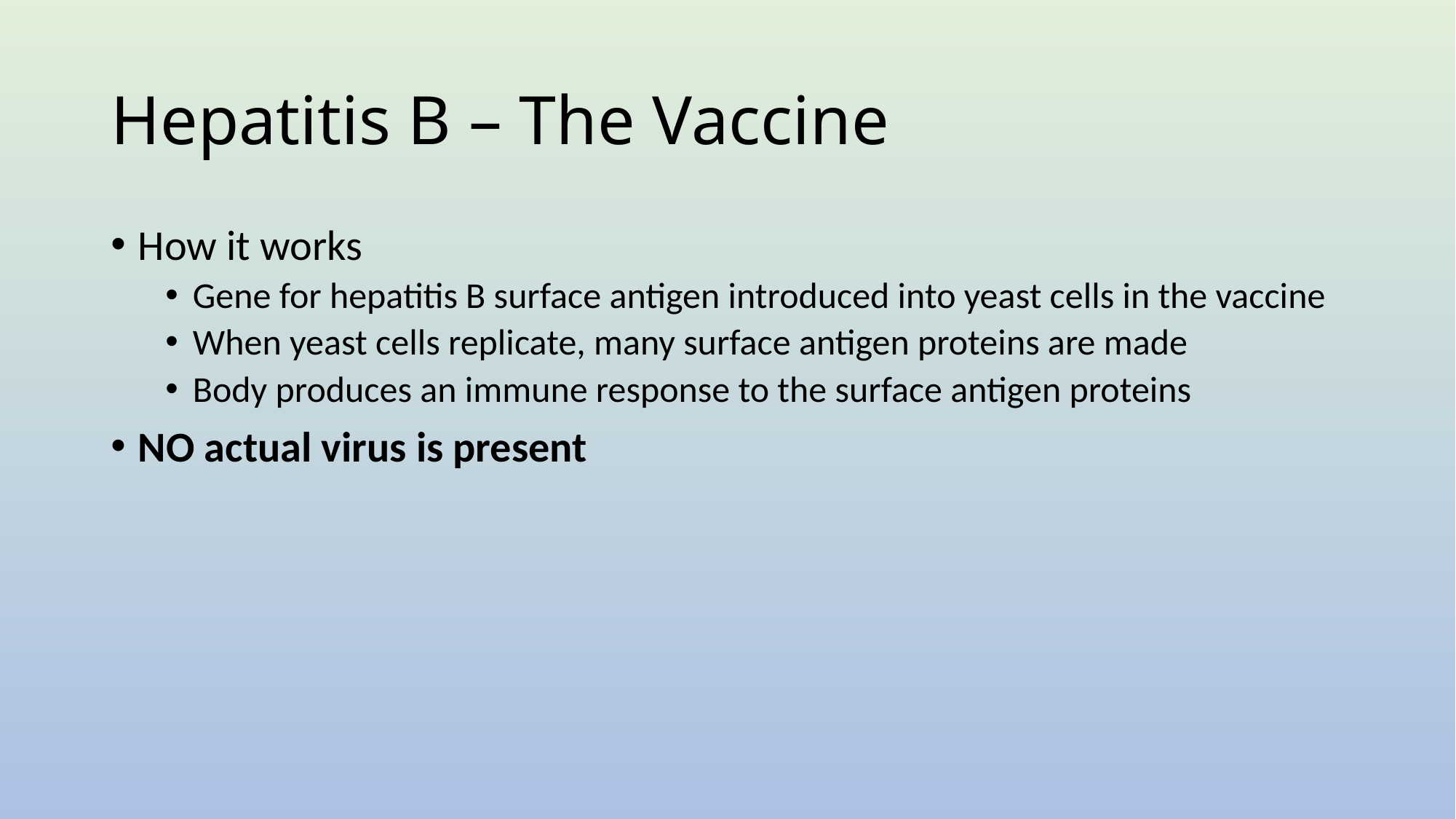

# Hepatitis B – The Vaccine
How it works
Gene for hepatitis B surface antigen introduced into yeast cells in the vaccine
When yeast cells replicate, many surface antigen proteins are made
Body produces an immune response to the surface antigen proteins
NO actual virus is present

## Slide 6
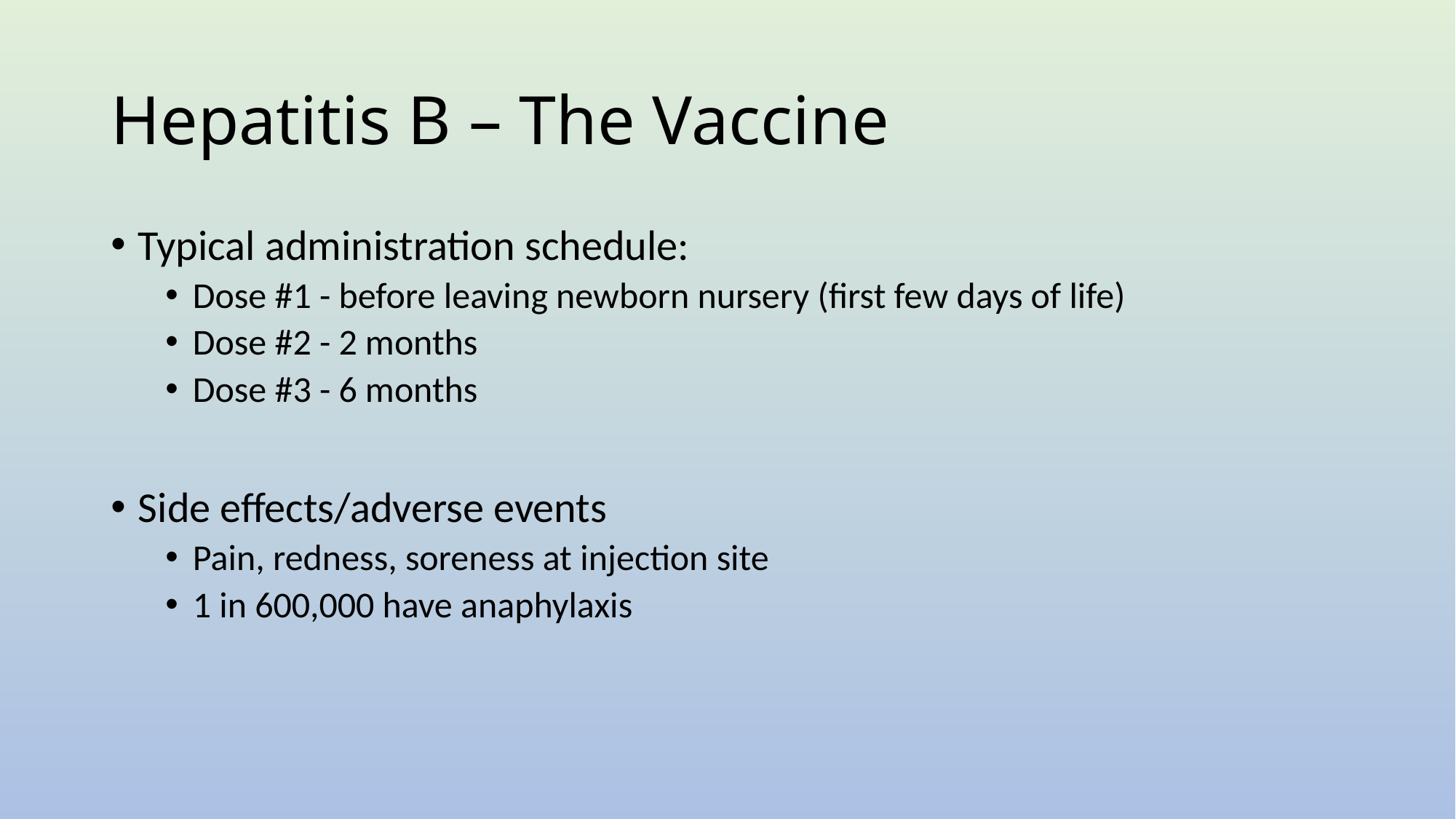

# Hepatitis B – The Vaccine
Typical administration schedule:
Dose #1 - before leaving newborn nursery (first few days of life)
Dose #2 - 2 months
Dose #3 - 6 months
Side effects/adverse events
Pain, redness, soreness at injection site
1 in 600,000 have anaphylaxis

## Slide 7
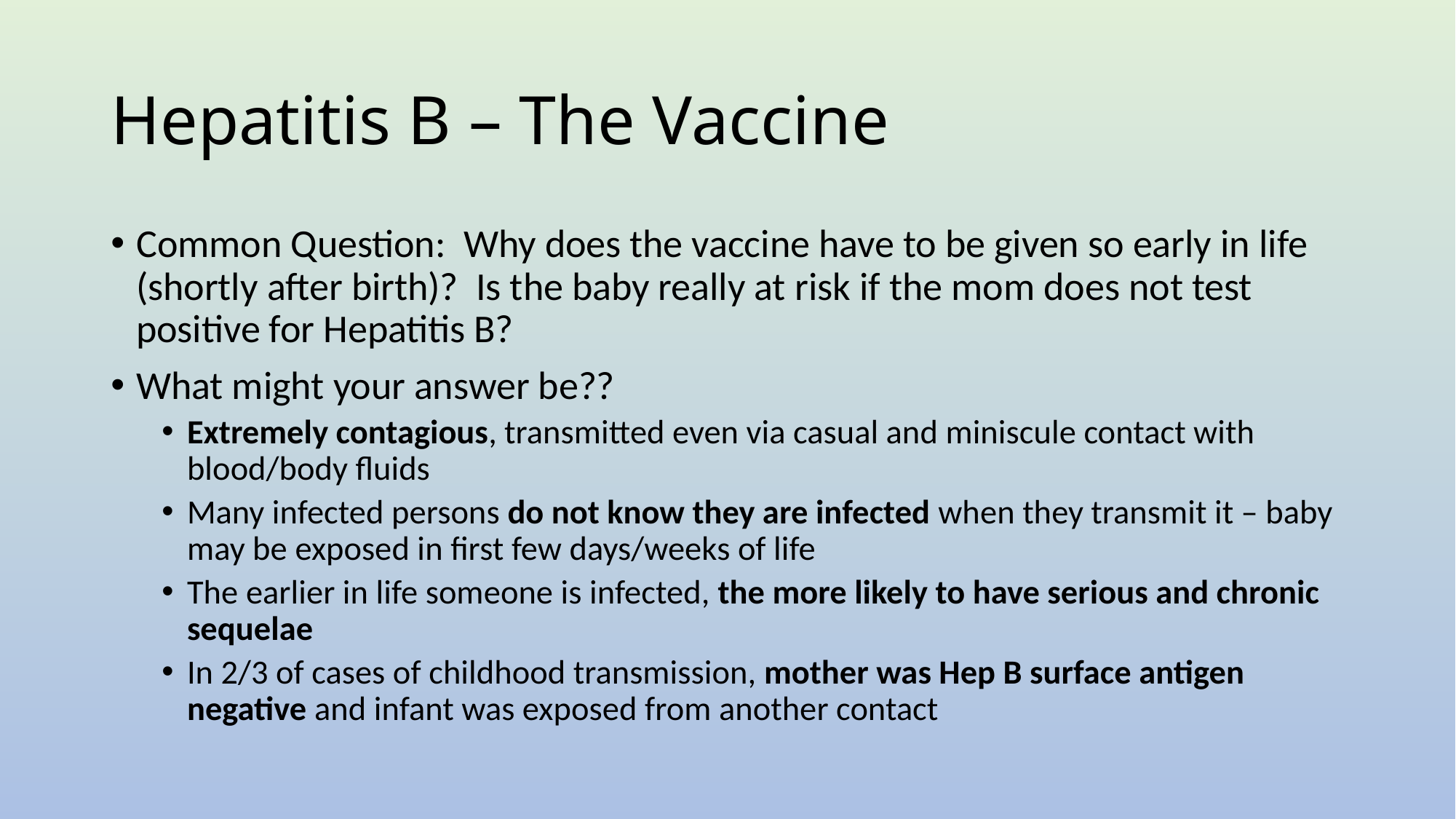

# Hepatitis B – The Vaccine
Common Question: Why does the vaccine have to be given so early in life (shortly after birth)? Is the baby really at risk if the mom does not test positive for Hepatitis B?
What might your answer be??
Extremely contagious, transmitted even via casual and miniscule contact with blood/body fluids
Many infected persons do not know they are infected when they transmit it – baby may be exposed in first few days/weeks of life
The earlier in life someone is infected, the more likely to have serious and chronic sequelae
In 2/3 of cases of childhood transmission, mother was Hep B surface antigen negative and infant was exposed from another contact

## Slide 8
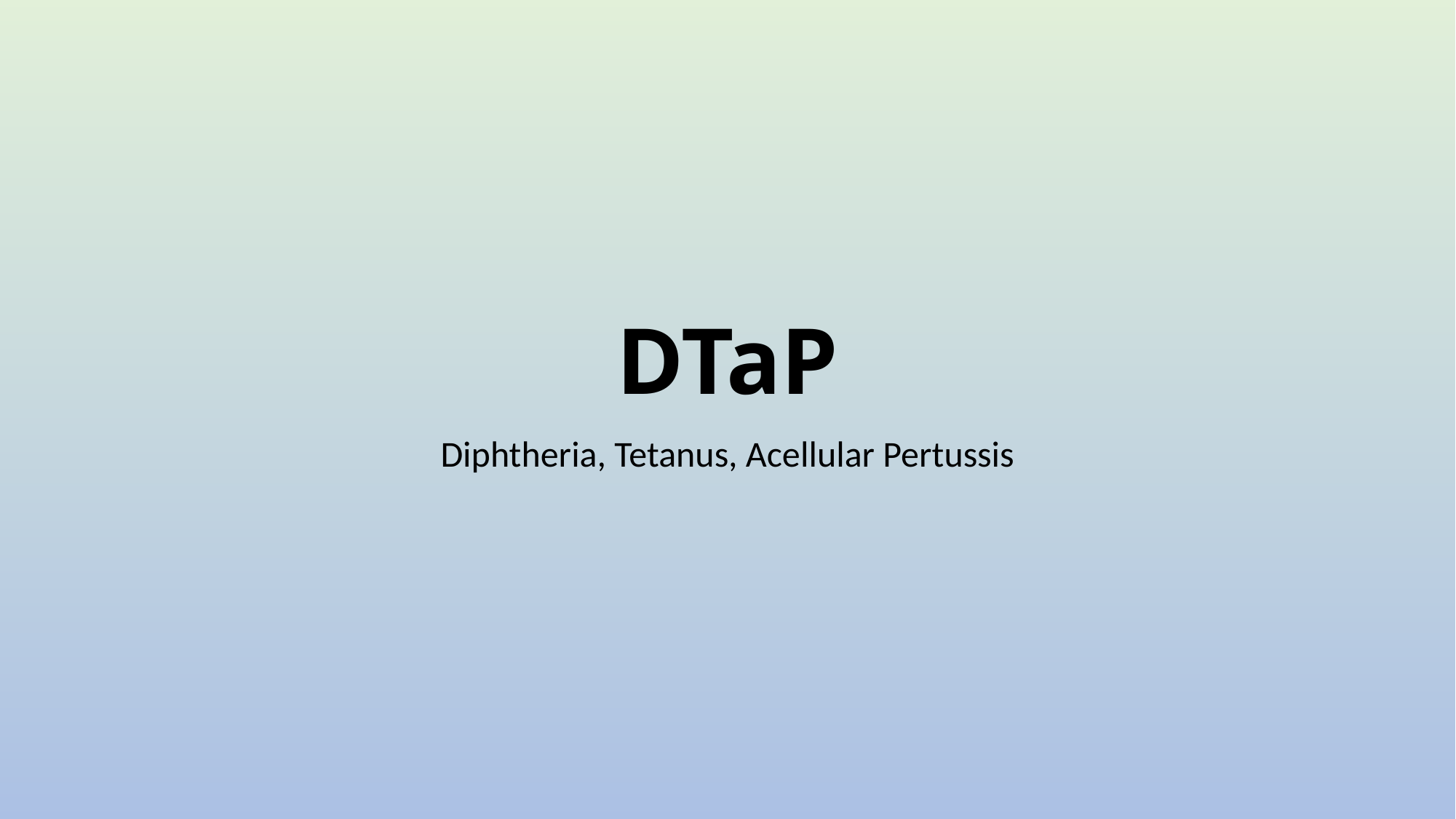

# DTaP
Diphtheria, Tetanus, Acellular Pertussis

## Slide 9
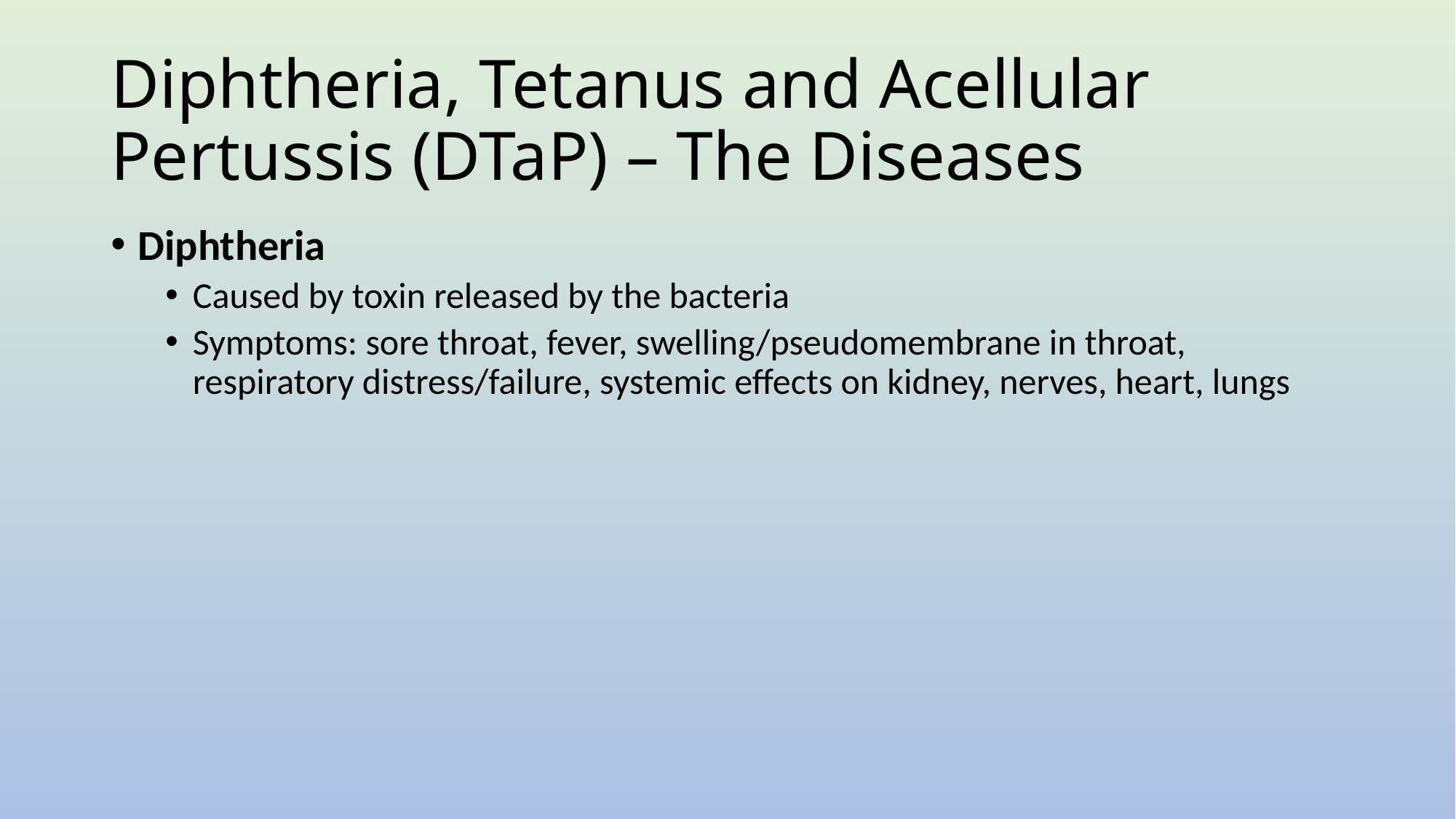

# Diphtheria, Tetanus and Acellular Pertussis (DTaP) – The Diseases
Diphtheria
Caused by toxin released by the bacteria
Symptoms: sore throat, fever, swelling/pseudomembrane in throat, respiratory distress/failure, systemic effects on kidney, nerves, heart, lungs

## Slide 10
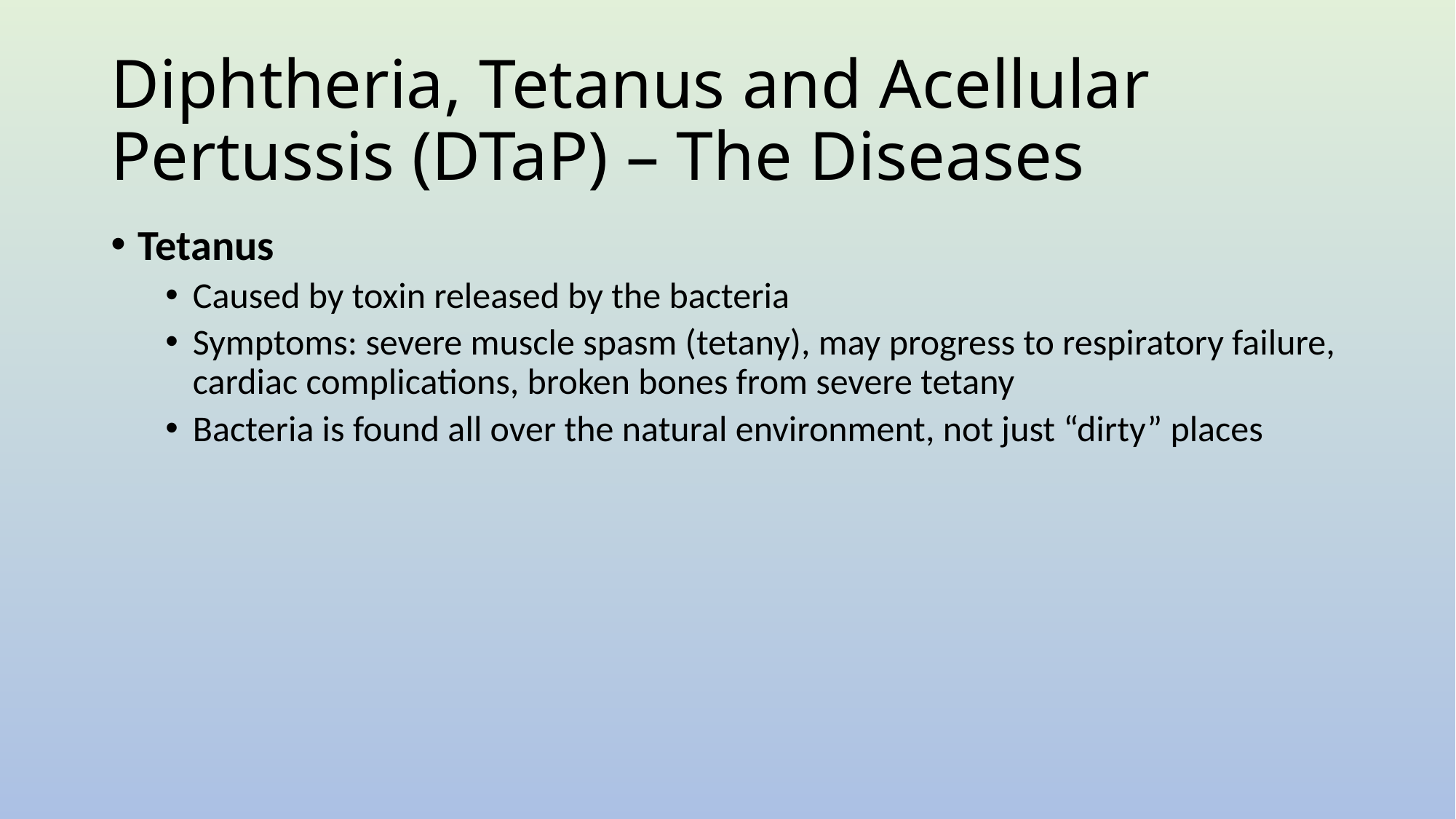

# Diphtheria, Tetanus and Acellular Pertussis (DTaP) – The Diseases
Tetanus
Caused by toxin released by the bacteria
Symptoms: severe muscle spasm (tetany), may progress to respiratory failure, cardiac complications, broken bones from severe tetany
Bacteria is found all over the natural environment, not just “dirty” places

## Slide 11
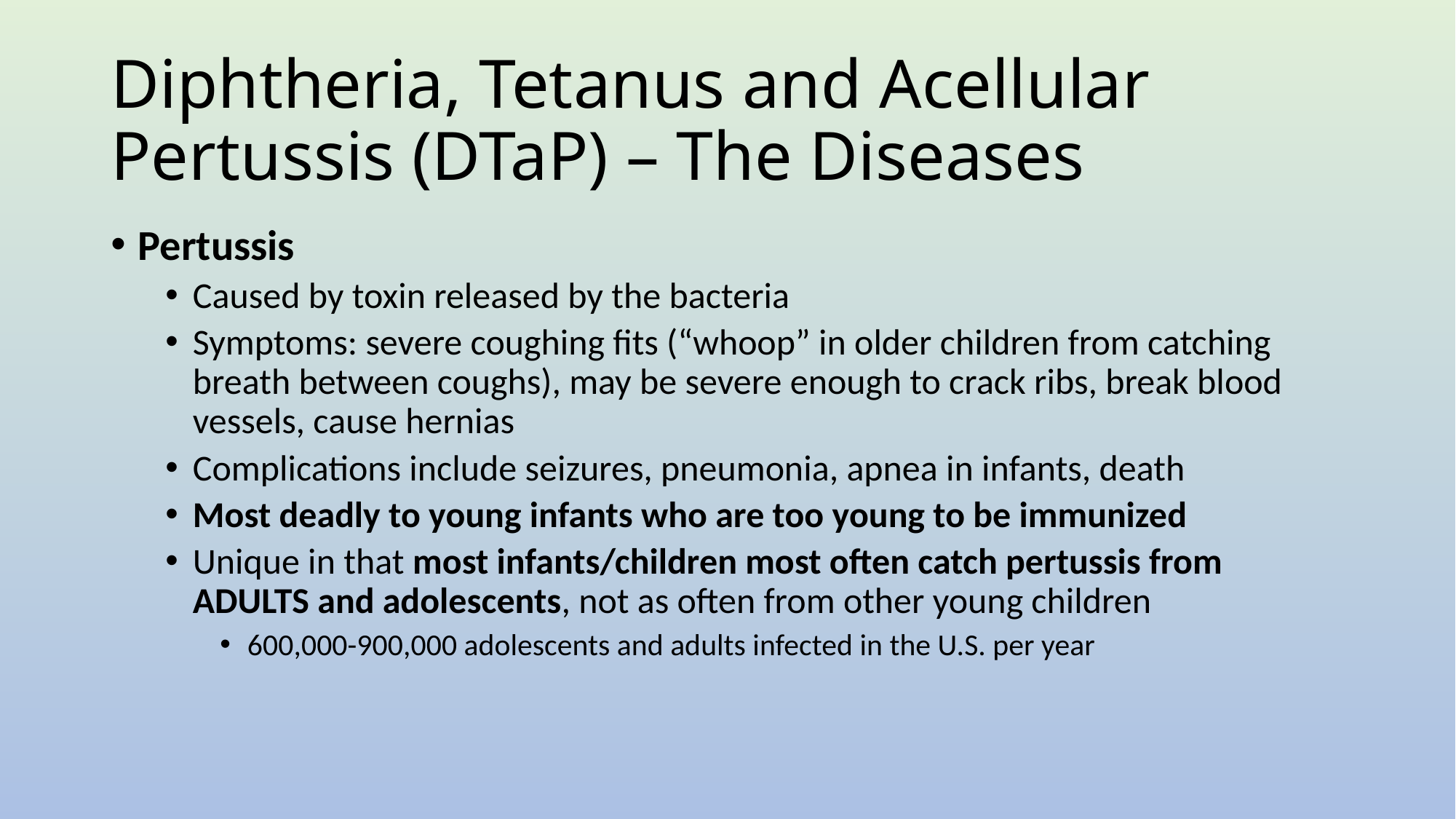

# Diphtheria, Tetanus and Acellular Pertussis (DTaP) – The Diseases
Pertussis
Caused by toxin released by the bacteria
Symptoms: severe coughing fits (“whoop” in older children from catching breath between coughs), may be severe enough to crack ribs, break blood vessels, cause hernias
Complications include seizures, pneumonia, apnea in infants, death
Most deadly to young infants who are too young to be immunized
Unique in that most infants/children most often catch pertussis from ADULTS and adolescents, not as often from other young children
600,000-900,000 adolescents and adults infected in the U.S. per year

## Slide 12
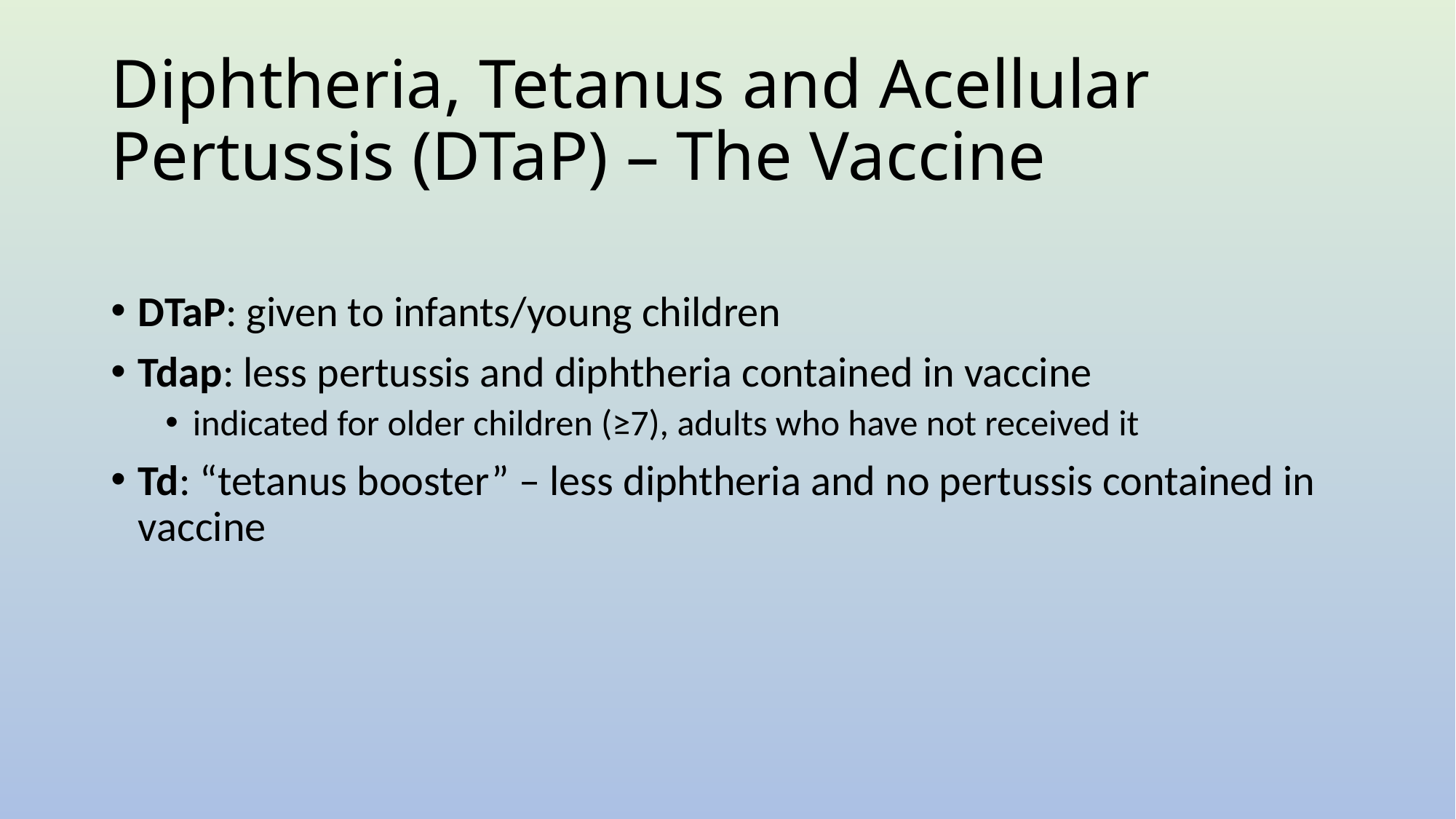

# Diphtheria, Tetanus and Acellular Pertussis (DTaP) – The Vaccine
DTaP: given to infants/young children
Tdap: less pertussis and diphtheria contained in vaccine
indicated for older children (≥7), adults who have not received it
Td: “tetanus booster” – less diphtheria and no pertussis contained in vaccine

## Slide 13
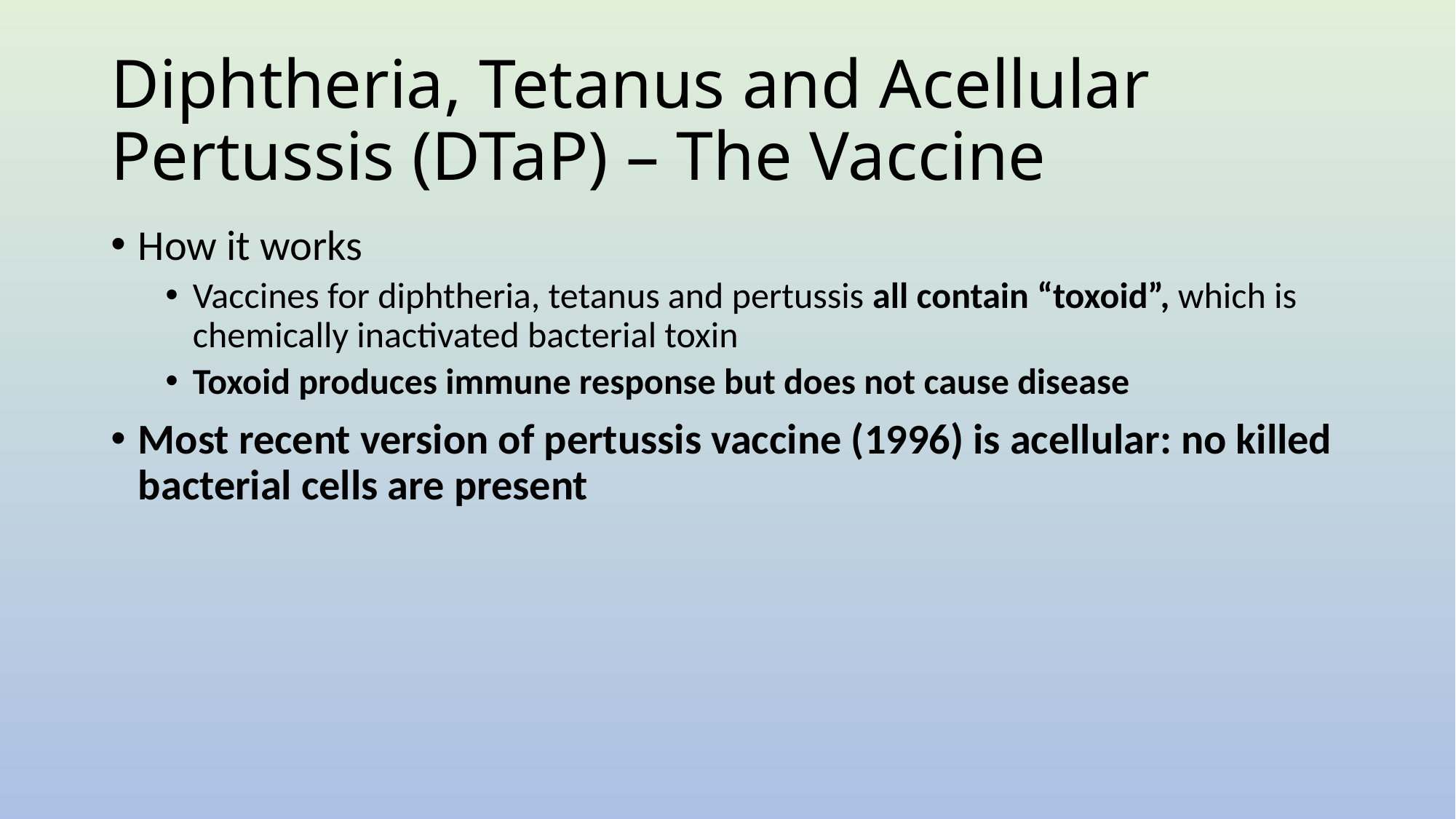

# Diphtheria, Tetanus and Acellular Pertussis (DTaP) – The Vaccine
How it works
Vaccines for diphtheria, tetanus and pertussis all contain “toxoid”, which is chemically inactivated bacterial toxin
Toxoid produces immune response but does not cause disease
Most recent version of pertussis vaccine (1996) is acellular: no killed bacterial cells are present

## Slide 14
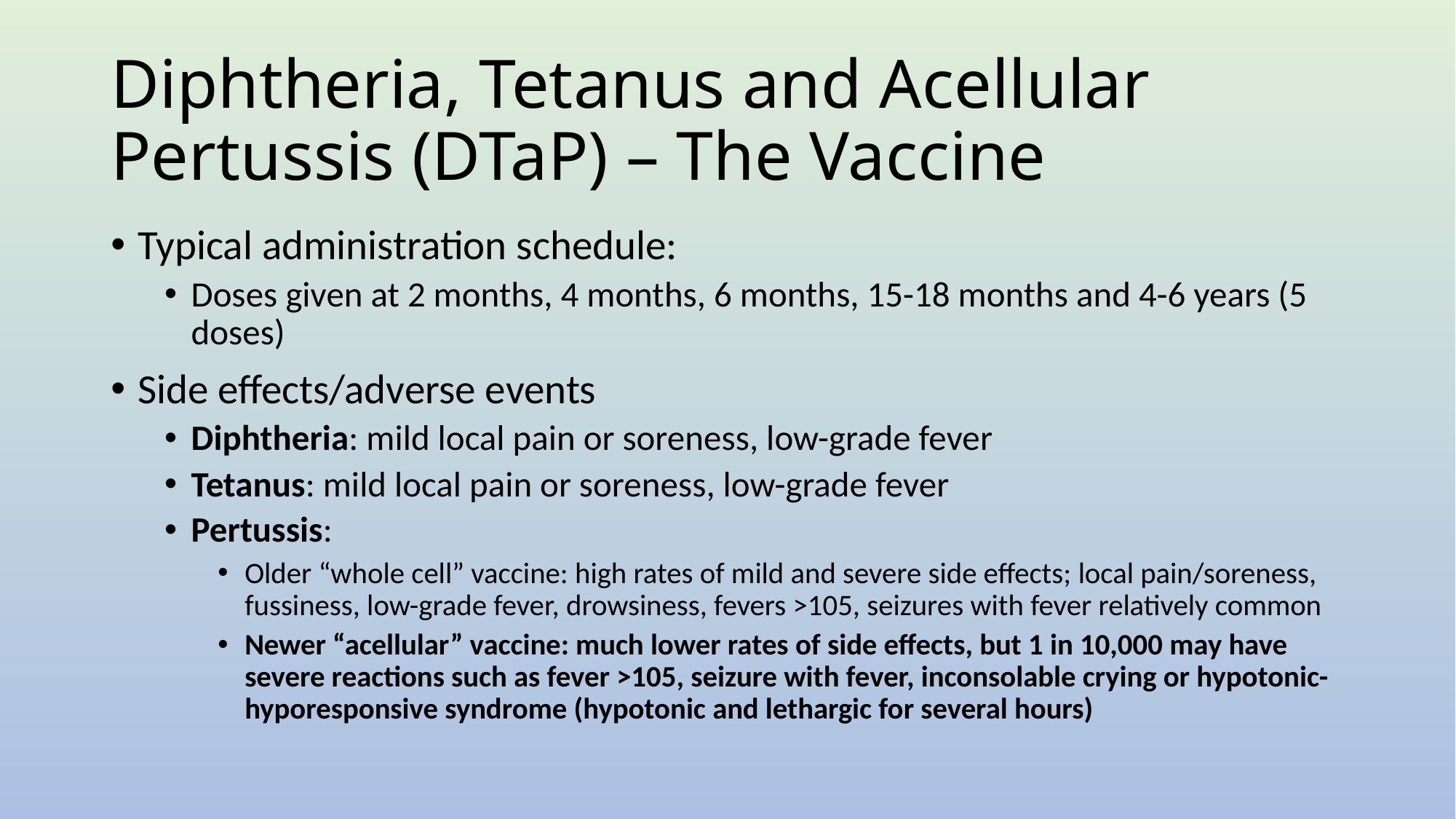

# Diphtheria, Tetanus and Acellular Pertussis (DTaP) – The Vaccine
Typical administration schedule:
Doses given at 2 months, 4 months, 6 months, 15-18 months and 4-6 years (5 doses)
Side effects/adverse events
Diphtheria: mild local pain or soreness, low-grade fever
Tetanus: mild local pain or soreness, low-grade fever
Pertussis:
Older “whole cell” vaccine: high rates of mild and severe side effects; local pain/soreness, fussiness, low-grade fever, drowsiness, fevers >105, seizures with fever relatively common
Newer “acellular” vaccine: much lower rates of side effects, but 1 in 10,000 may have severe reactions such as fever >105, seizure with fever, inconsolable crying or hypotonic-hyporesponsive syndrome (hypotonic and lethargic for several hours)

## Slide 15
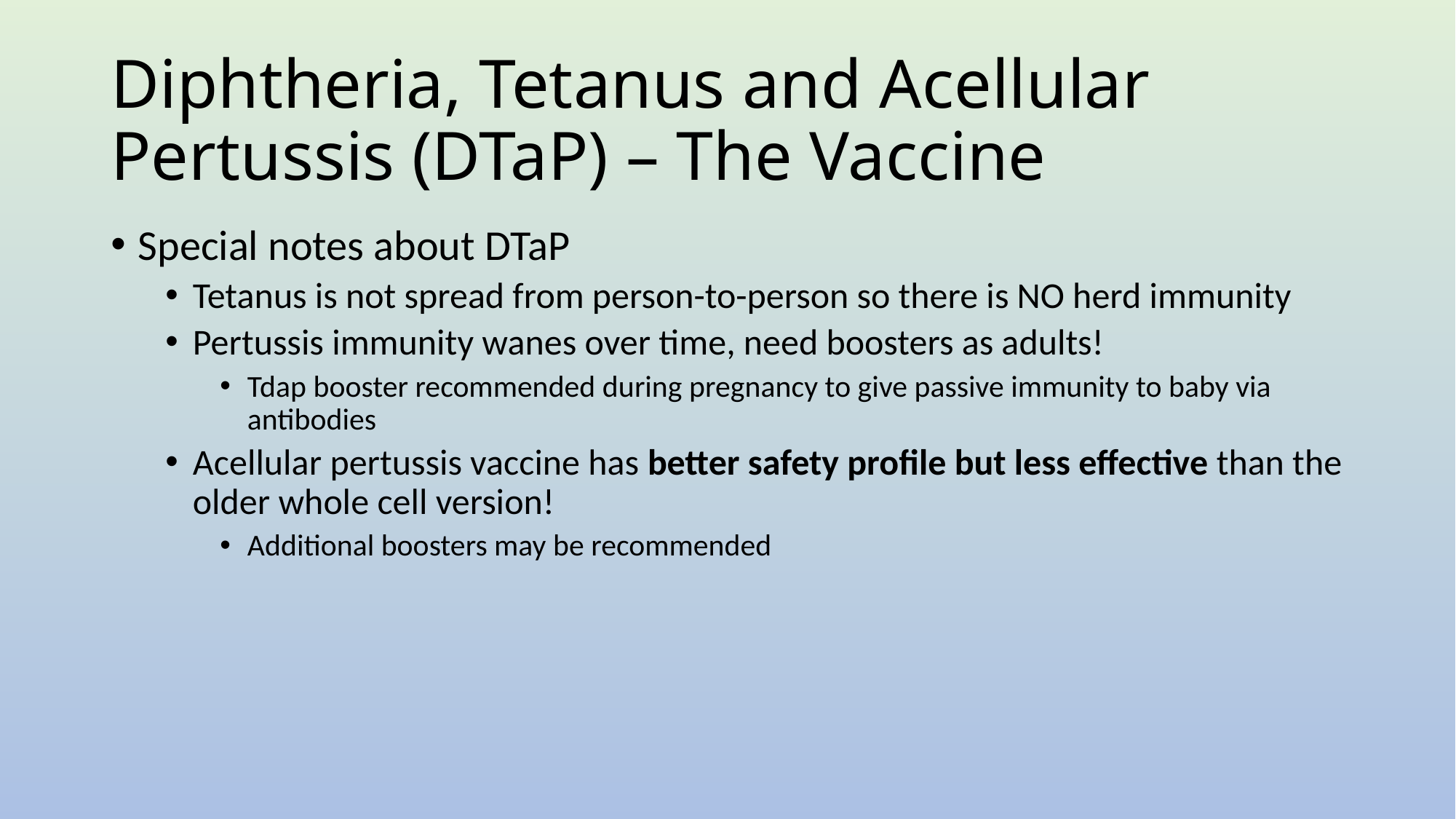

# Diphtheria, Tetanus and Acellular Pertussis (DTaP) – The Vaccine
Special notes about DTaP
Tetanus is not spread from person-to-person so there is NO herd immunity
Pertussis immunity wanes over time, need boosters as adults!
Tdap booster recommended during pregnancy to give passive immunity to baby via antibodies
Acellular pertussis vaccine has better safety profile but less effective than the older whole cell version!
Additional boosters may be recommended

## Slide 16
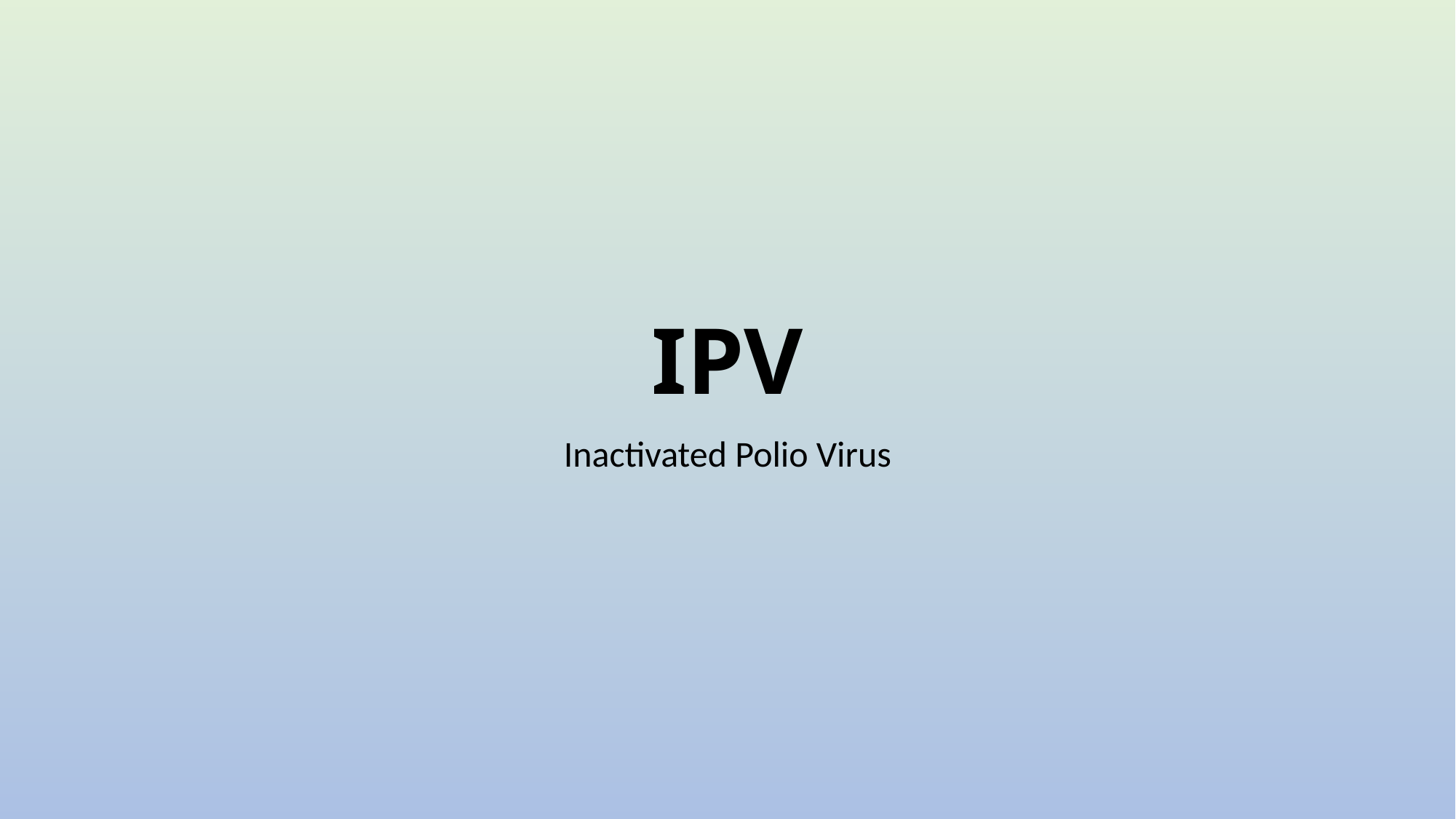

# IPV
Inactivated Polio Virus

## Slide 17
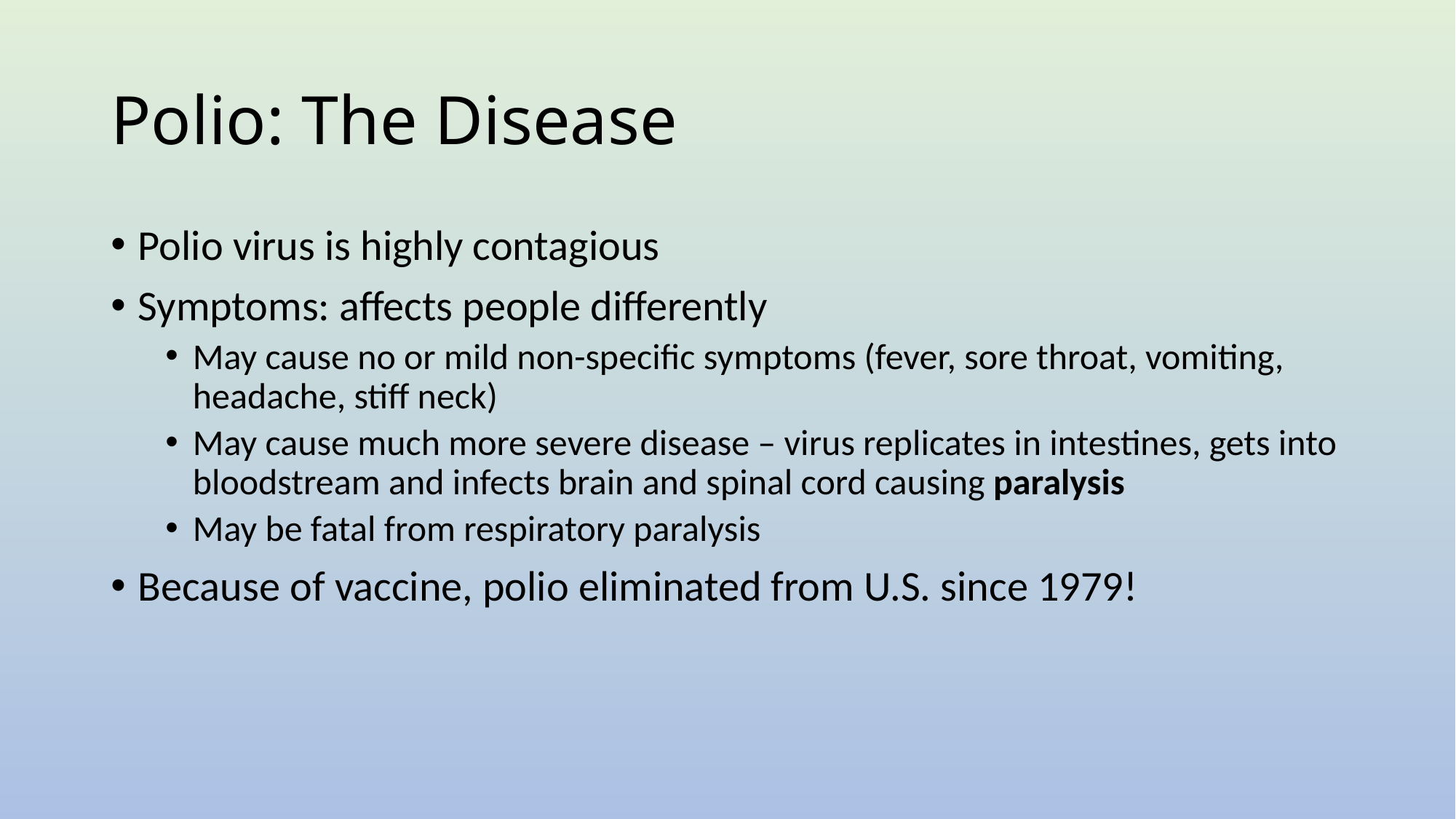

# Polio: The Disease
Polio virus is highly contagious
Symptoms: affects people differently
May cause no or mild non-specific symptoms (fever, sore throat, vomiting, headache, stiff neck)
May cause much more severe disease – virus replicates in intestines, gets into bloodstream and infects brain and spinal cord causing paralysis
May be fatal from respiratory paralysis
Because of vaccine, polio eliminated from U.S. since 1979!

## Slide 18
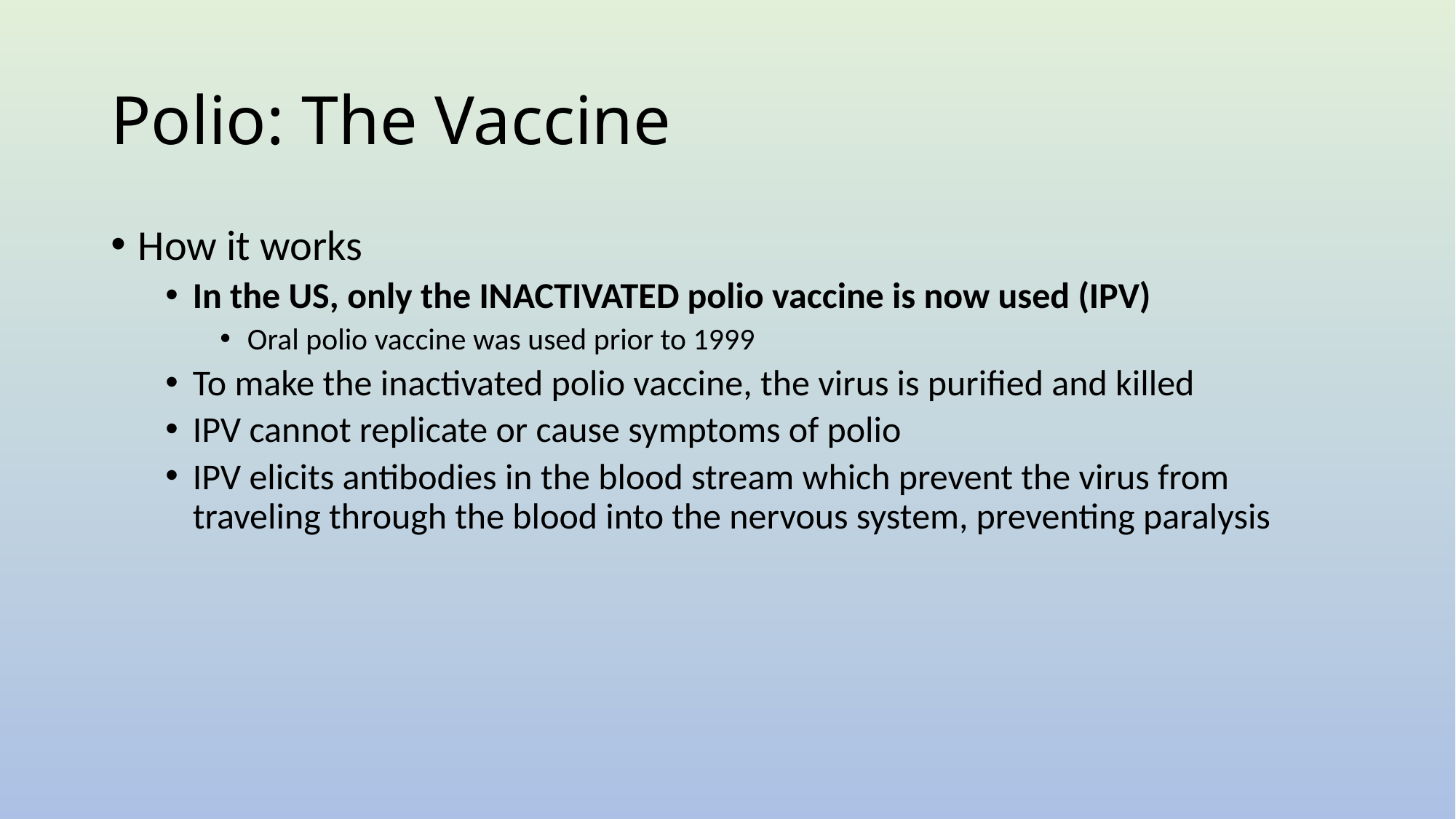

# Polio: The Vaccine
How it works
In the US, only the INACTIVATED polio vaccine is now used (IPV)
Oral polio vaccine was used prior to 1999
To make the inactivated polio vaccine, the virus is purified and killed
IPV cannot replicate or cause symptoms of polio
IPV elicits antibodies in the blood stream which prevent the virus from traveling through the blood into the nervous system, preventing paralysis

## Slide 19
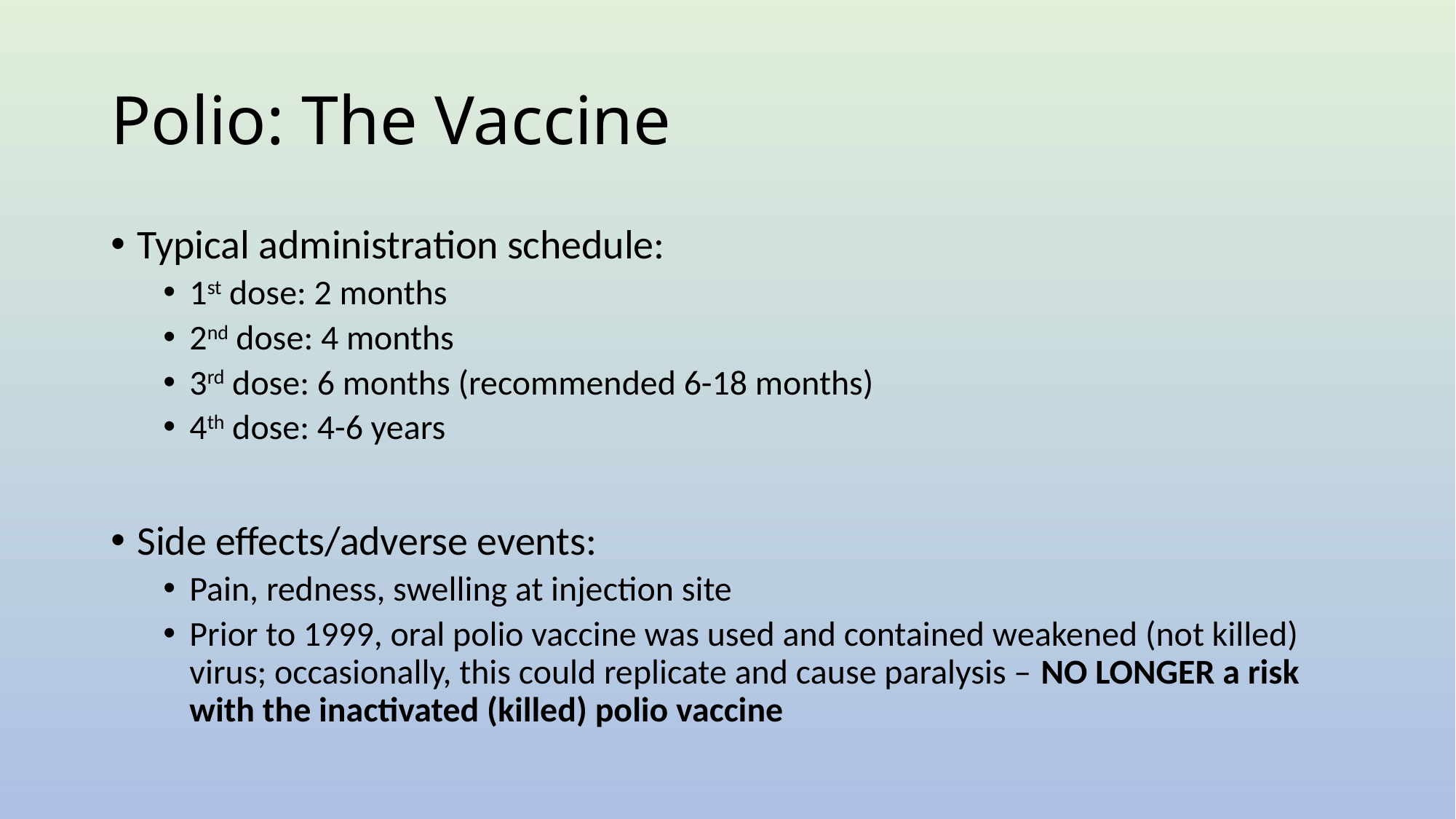

# Polio: The Vaccine
Typical administration schedule:
1st dose: 2 months
2nd dose: 4 months
3rd dose: 6 months (recommended 6-18 months)
4th dose: 4-6 years
Side effects/adverse events:
Pain, redness, swelling at injection site
Prior to 1999, oral polio vaccine was used and contained weakened (not killed) virus; occasionally, this could replicate and cause paralysis – NO LONGER a risk with the inactivated (killed) polio vaccine

## Slide 20
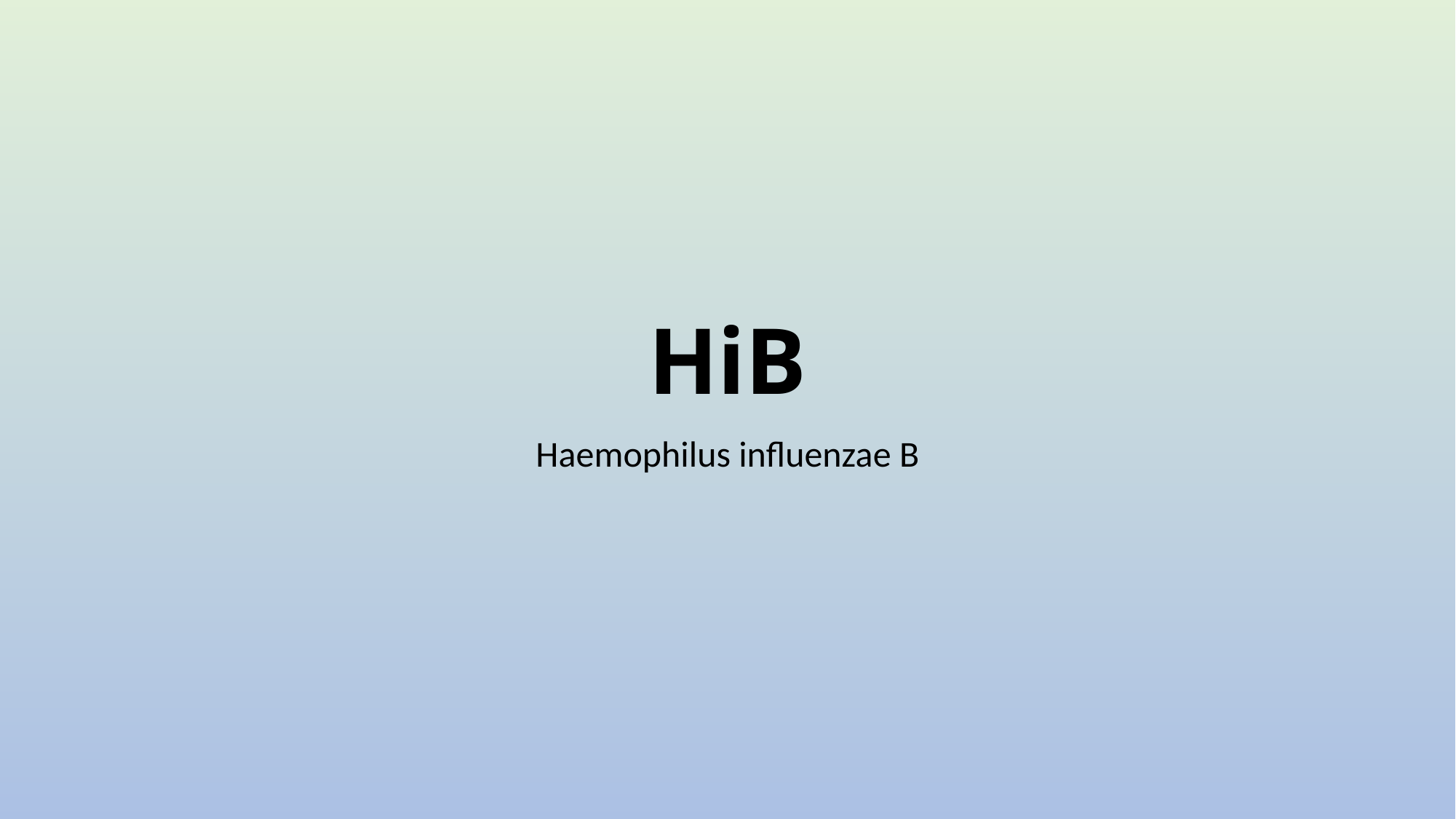

# HiB
Haemophilus influenzae B

## Slide 21
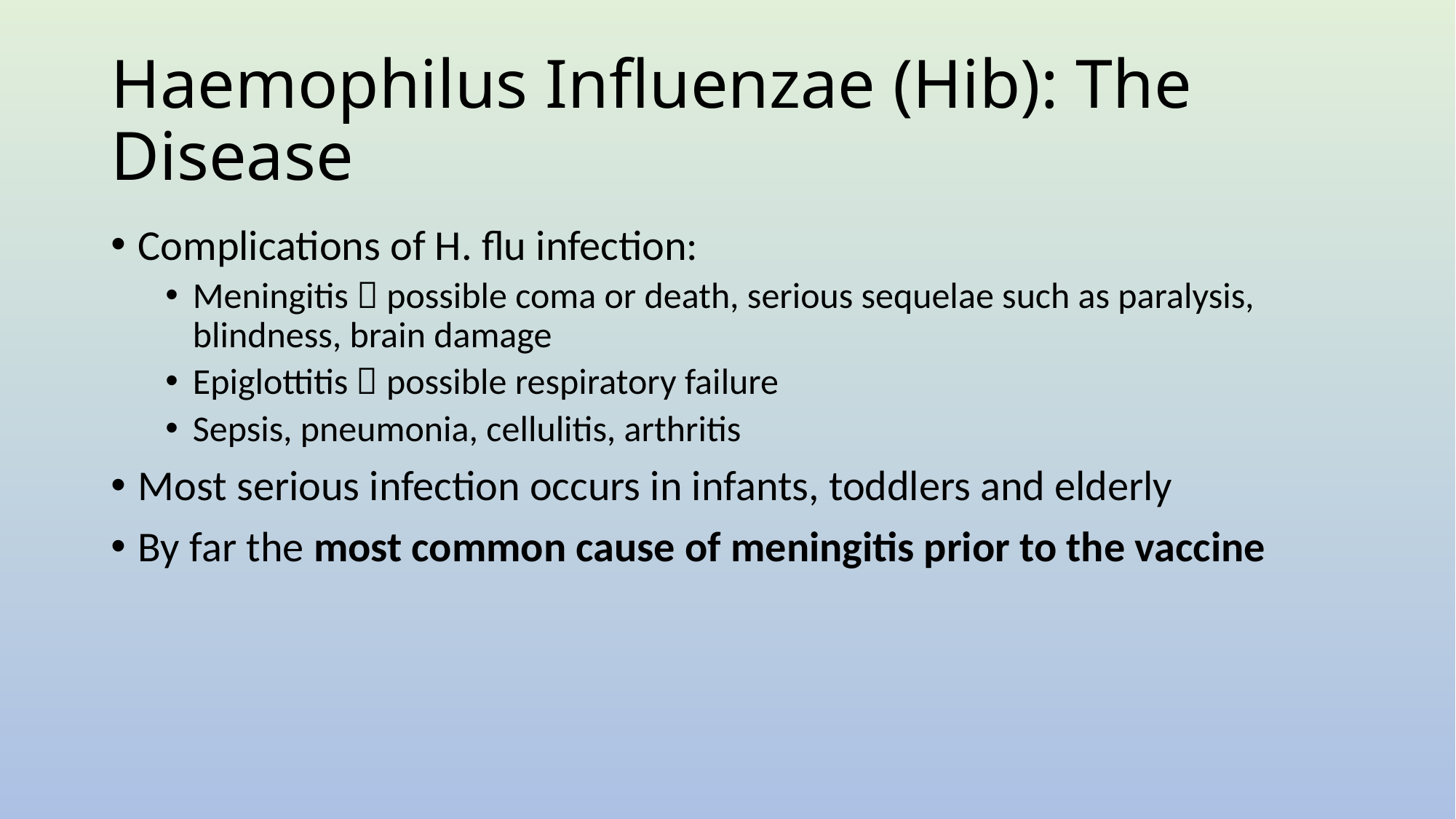

# Haemophilus Influenzae (Hib): The Disease
Complications of H. flu infection:
Meningitis  possible coma or death, serious sequelae such as paralysis, blindness, brain damage
Epiglottitis  possible respiratory failure
Sepsis, pneumonia, cellulitis, arthritis
Most serious infection occurs in infants, toddlers and elderly
By far the most common cause of meningitis prior to the vaccine

## Slide 22
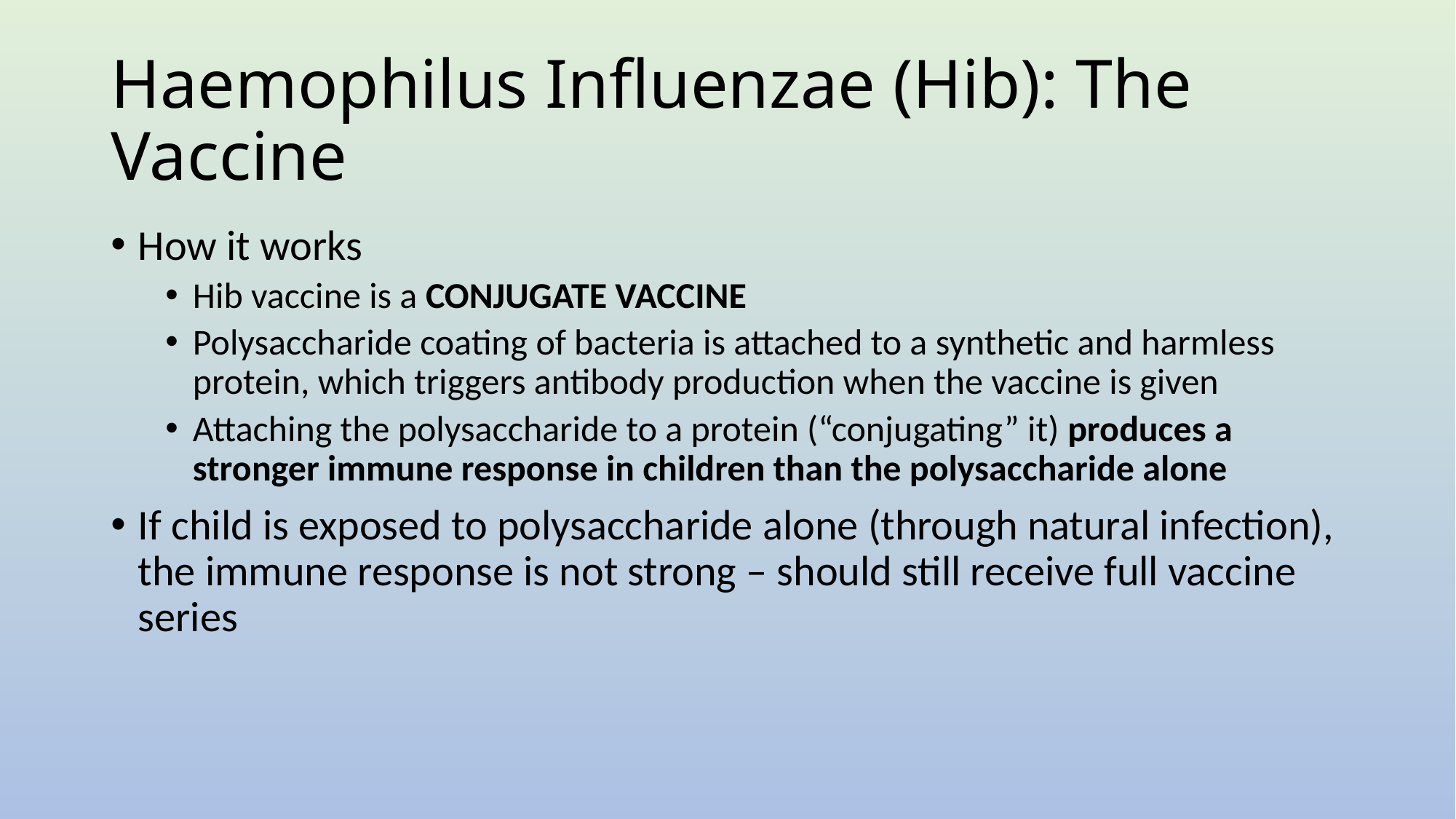

# Haemophilus Influenzae (Hib): The Vaccine
How it works
Hib vaccine is a CONJUGATE VACCINE
Polysaccharide coating of bacteria is attached to a synthetic and harmless protein, which triggers antibody production when the vaccine is given
Attaching the polysaccharide to a protein (“conjugating” it) produces a stronger immune response in children than the polysaccharide alone
If child is exposed to polysaccharide alone (through natural infection), the immune response is not strong – should still receive full vaccine series

## Slide 23
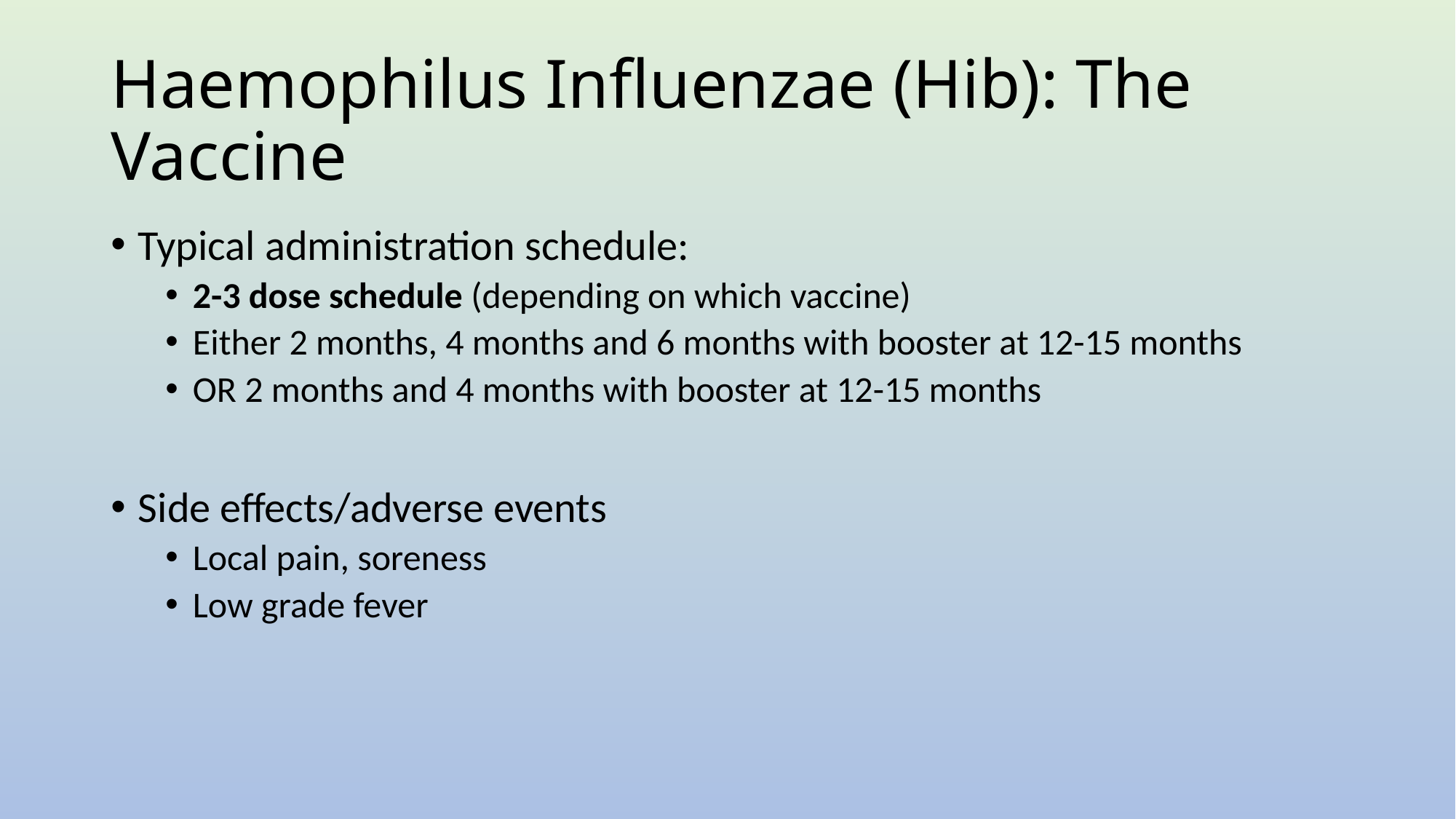

# Haemophilus Influenzae (Hib): The Vaccine
Typical administration schedule:
2-3 dose schedule (depending on which vaccine)
Either 2 months, 4 months and 6 months with booster at 12-15 months
OR 2 months and 4 months with booster at 12-15 months
Side effects/adverse events
Local pain, soreness
Low grade fever

## Slide 24
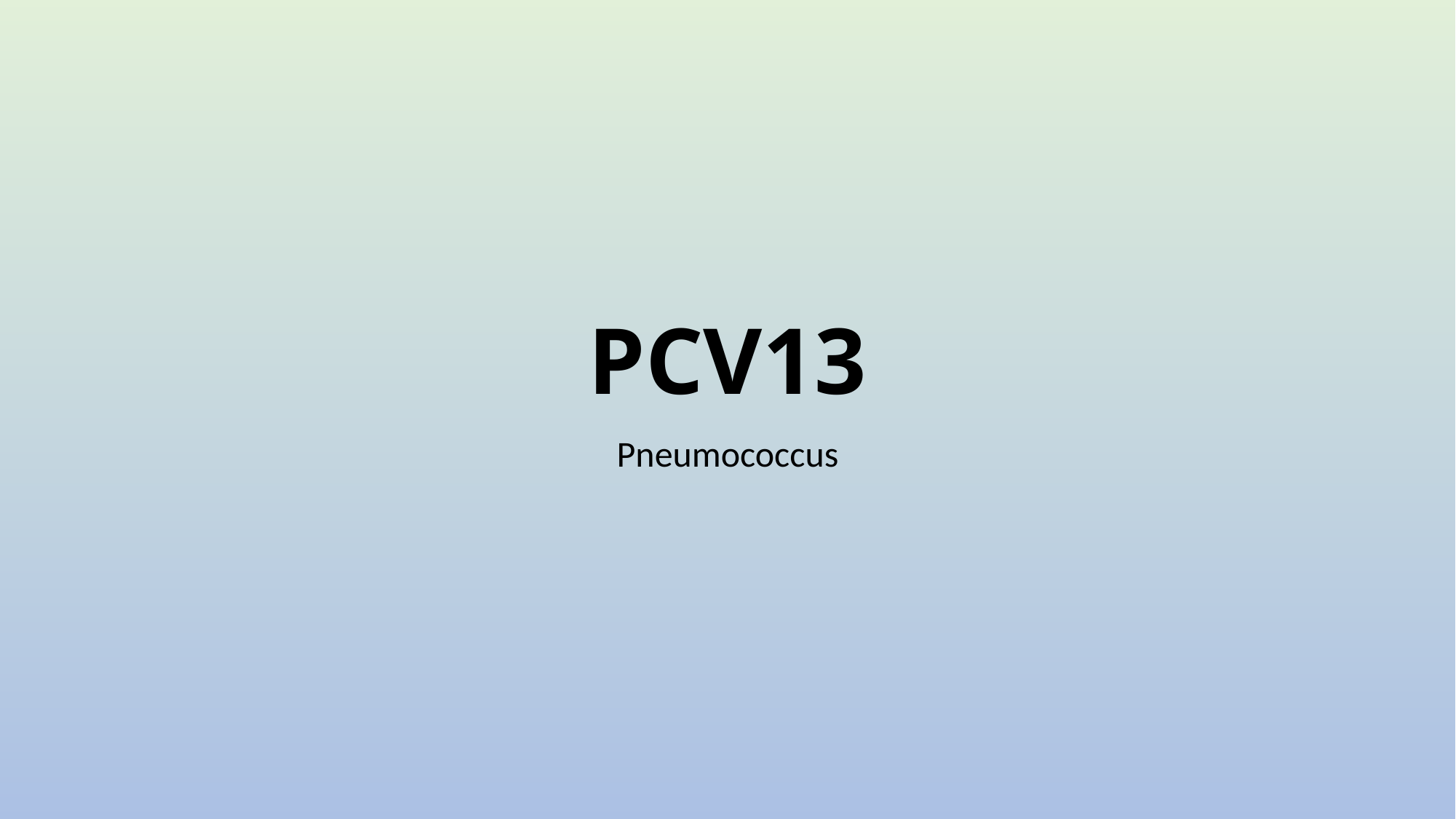

# PCV13
Pneumococcus

## Slide 25
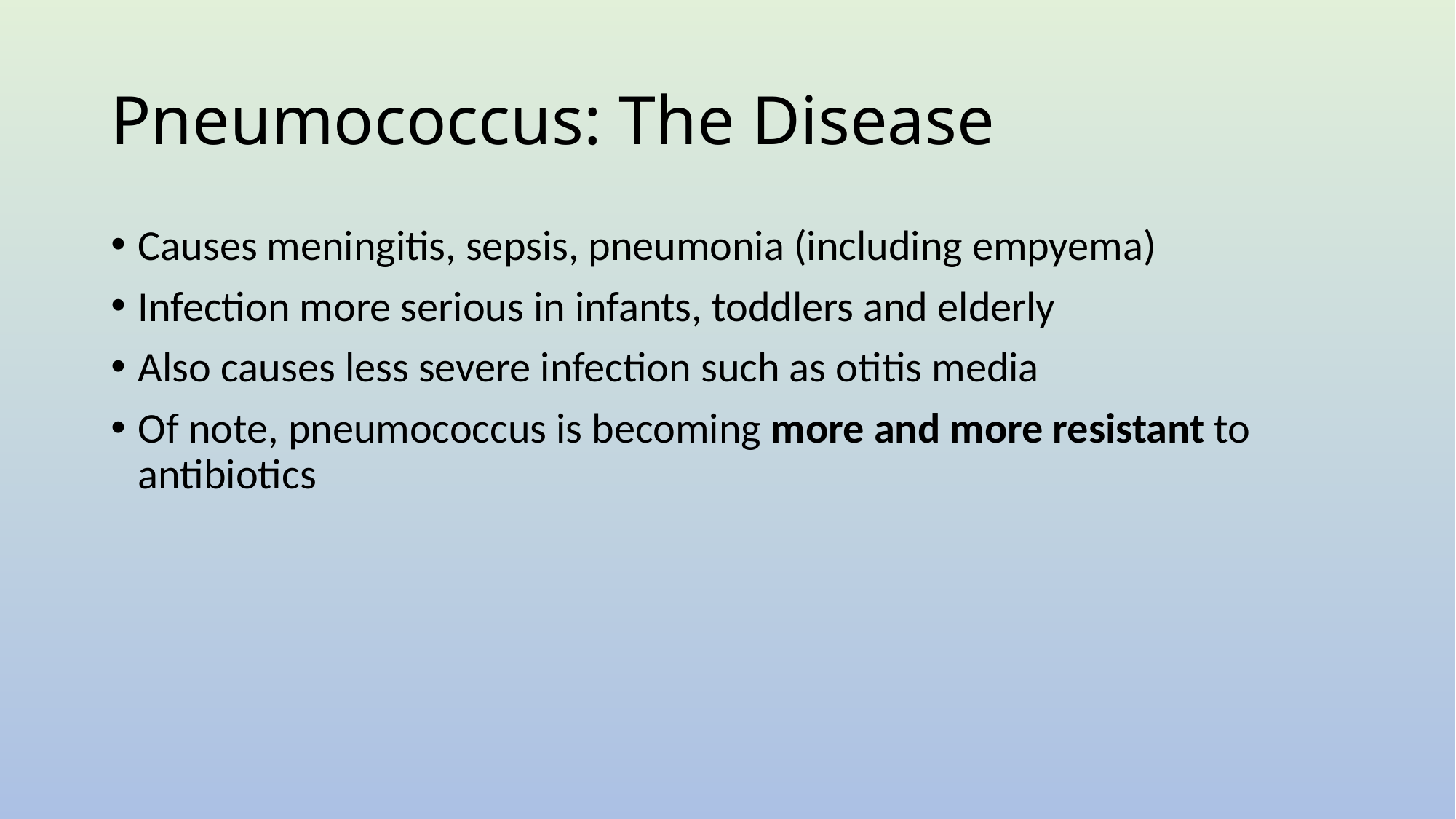

# Pneumococcus: The Disease
Causes meningitis, sepsis, pneumonia (including empyema)
Infection more serious in infants, toddlers and elderly
Also causes less severe infection such as otitis media
Of note, pneumococcus is becoming more and more resistant to antibiotics

## Slide 26
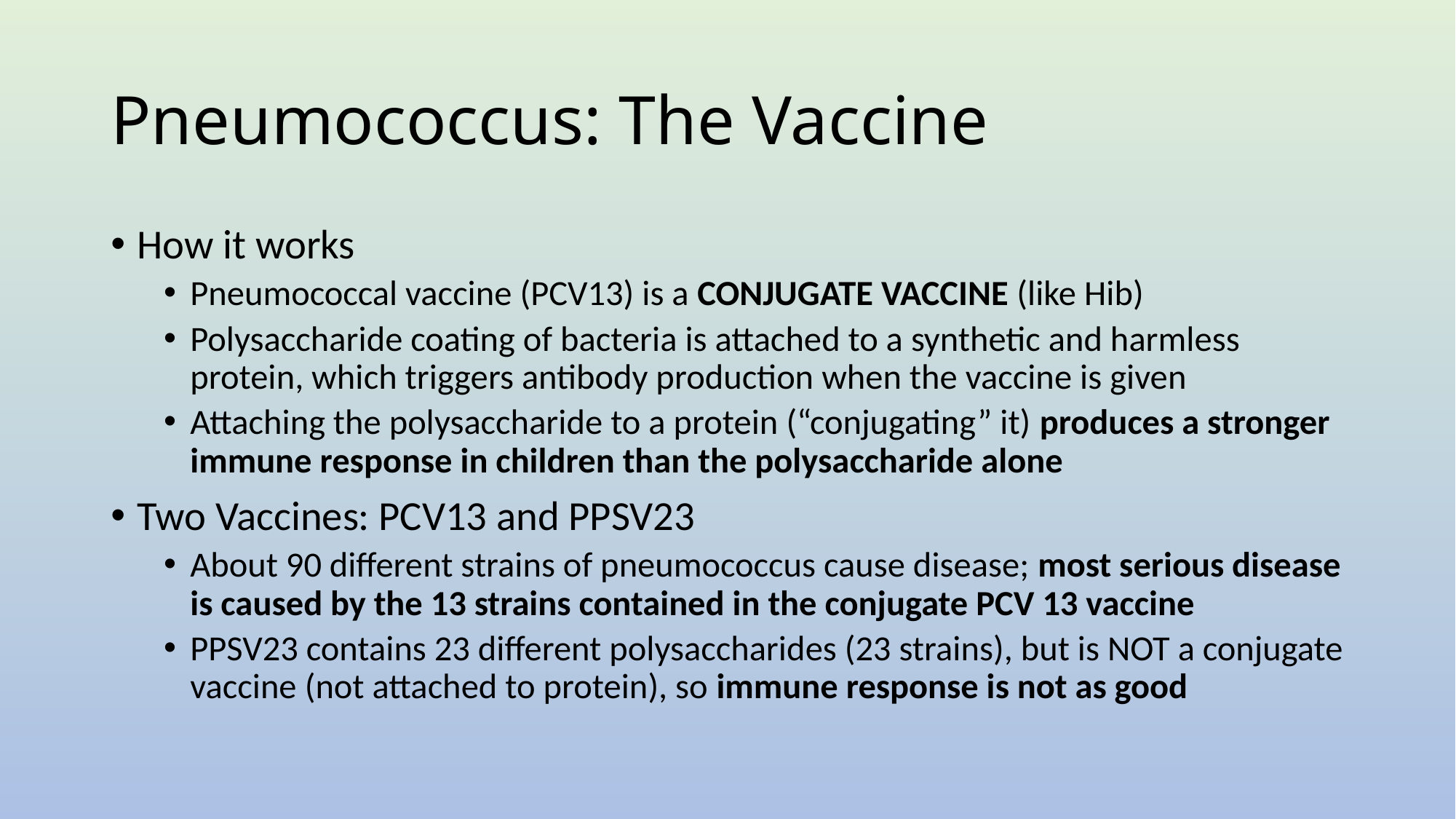

# Pneumococcus: The Vaccine
How it works
Pneumococcal vaccine (PCV13) is a CONJUGATE VACCINE (like Hib)
Polysaccharide coating of bacteria is attached to a synthetic and harmless protein, which triggers antibody production when the vaccine is given
Attaching the polysaccharide to a protein (“conjugating” it) produces a stronger immune response in children than the polysaccharide alone
Two Vaccines: PCV13 and PPSV23
About 90 different strains of pneumococcus cause disease; most serious disease is caused by the 13 strains contained in the conjugate PCV 13 vaccine
PPSV23 contains 23 different polysaccharides (23 strains), but is NOT a conjugate vaccine (not attached to protein), so immune response is not as good

## Slide 27
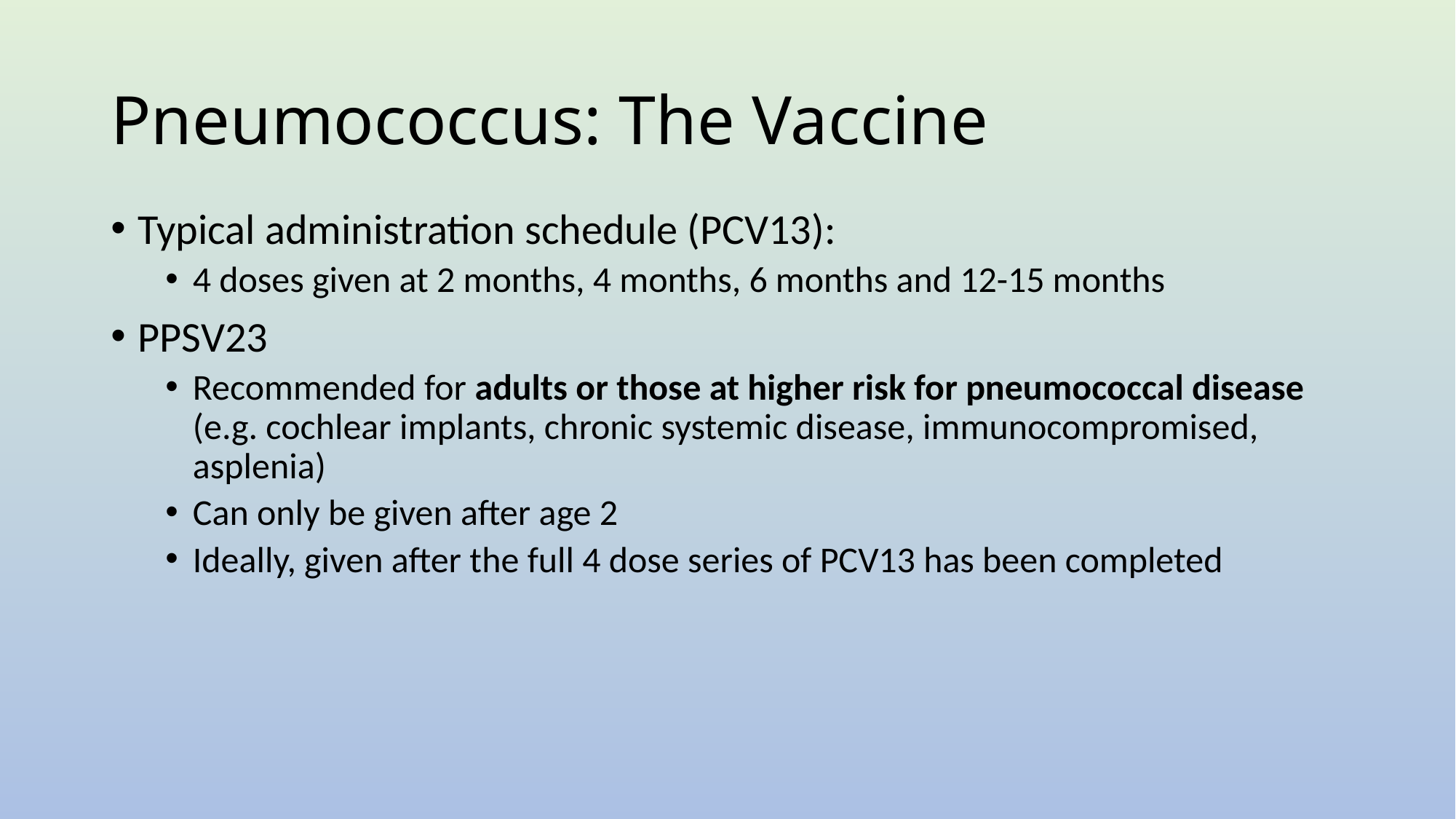

# Pneumococcus: The Vaccine
Typical administration schedule (PCV13):
4 doses given at 2 months, 4 months, 6 months and 12-15 months
PPSV23
Recommended for adults or those at higher risk for pneumococcal disease (e.g. cochlear implants, chronic systemic disease, immunocompromised, asplenia)
Can only be given after age 2
Ideally, given after the full 4 dose series of PCV13 has been completed

## Slide 28
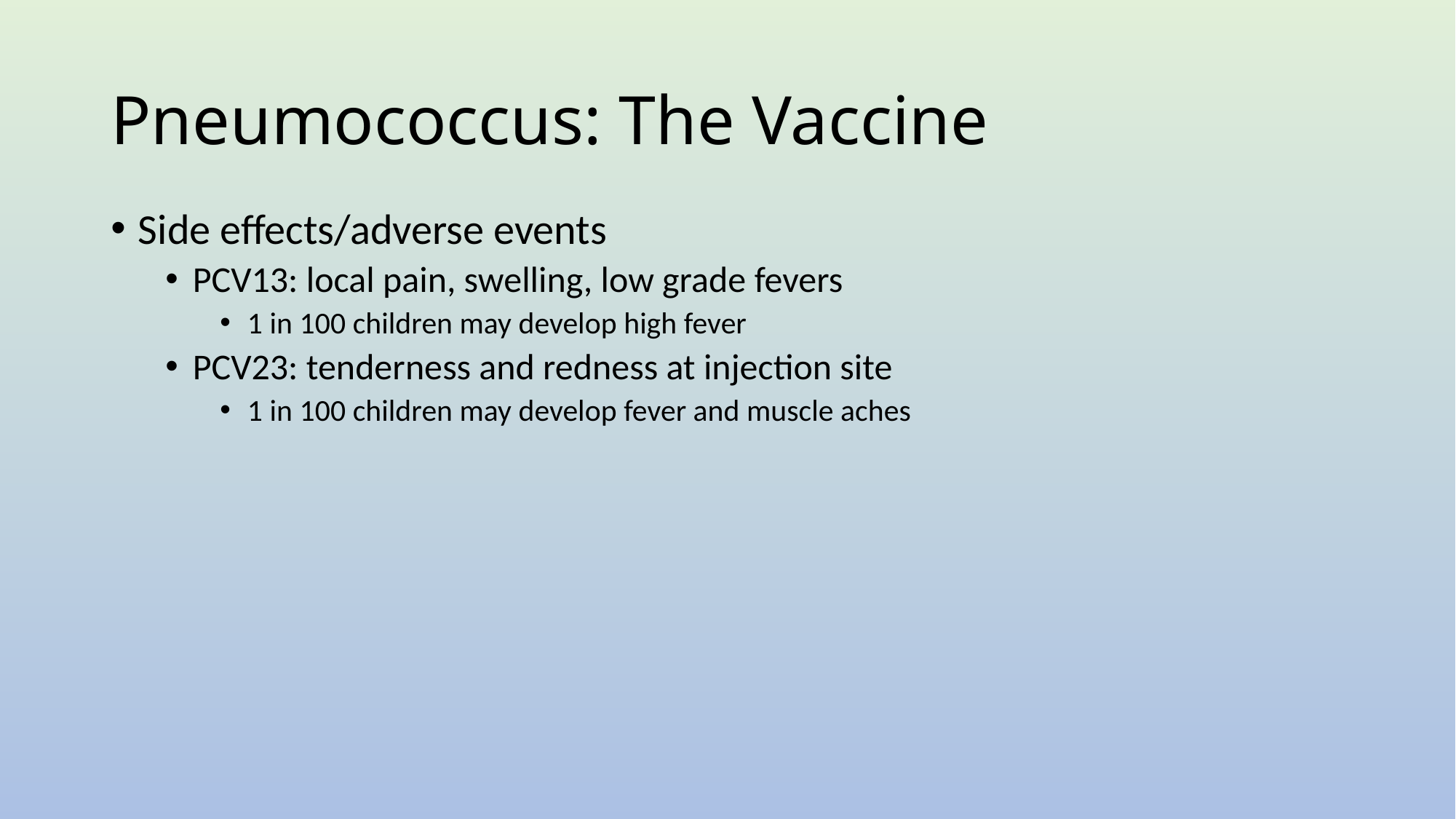

# Pneumococcus: The Vaccine
Side effects/adverse events
PCV13: local pain, swelling, low grade fevers
1 in 100 children may develop high fever
PCV23: tenderness and redness at injection site
1 in 100 children may develop fever and muscle aches

## Slide 29
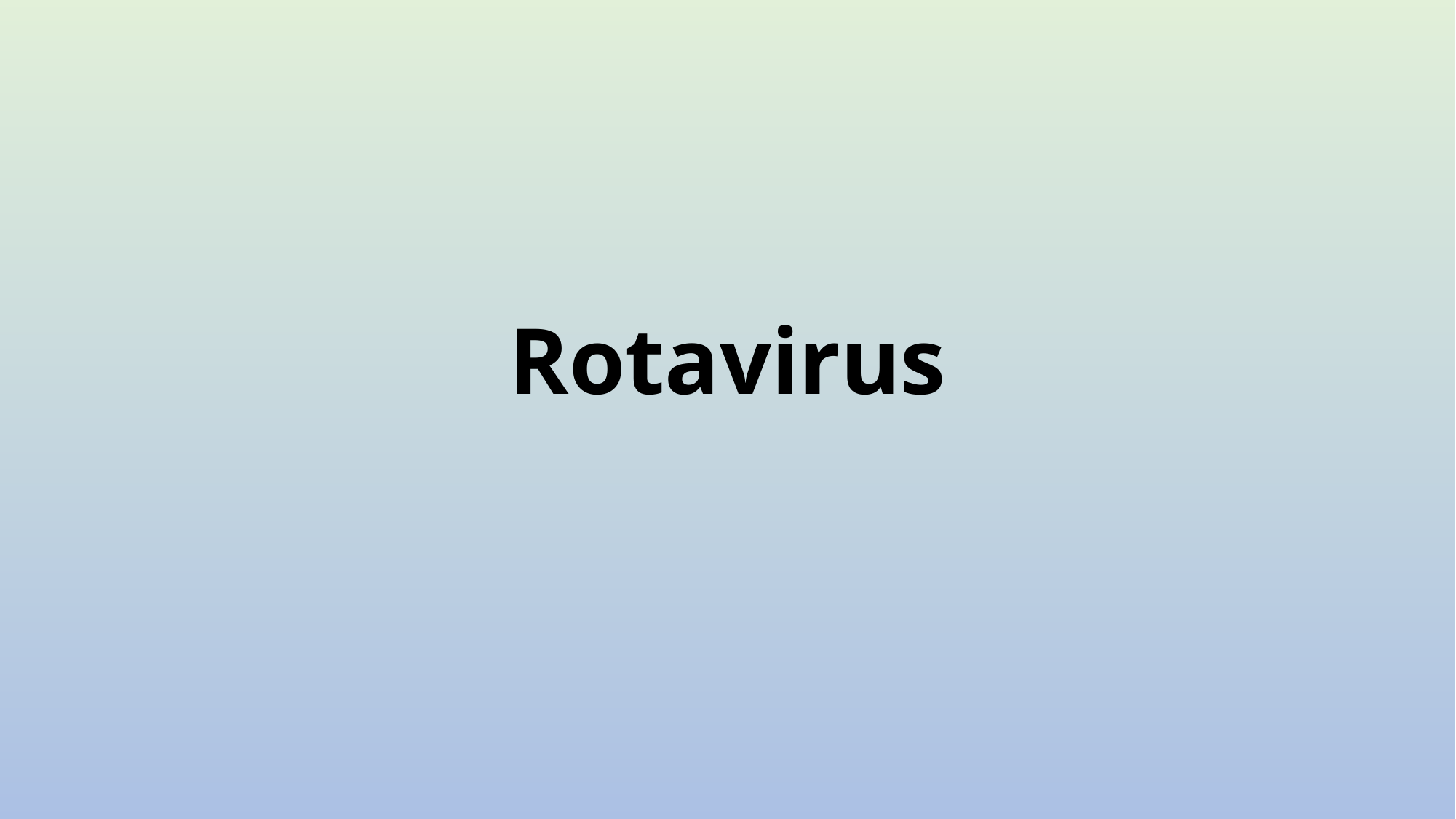

# Rotavirus

## Slide 30
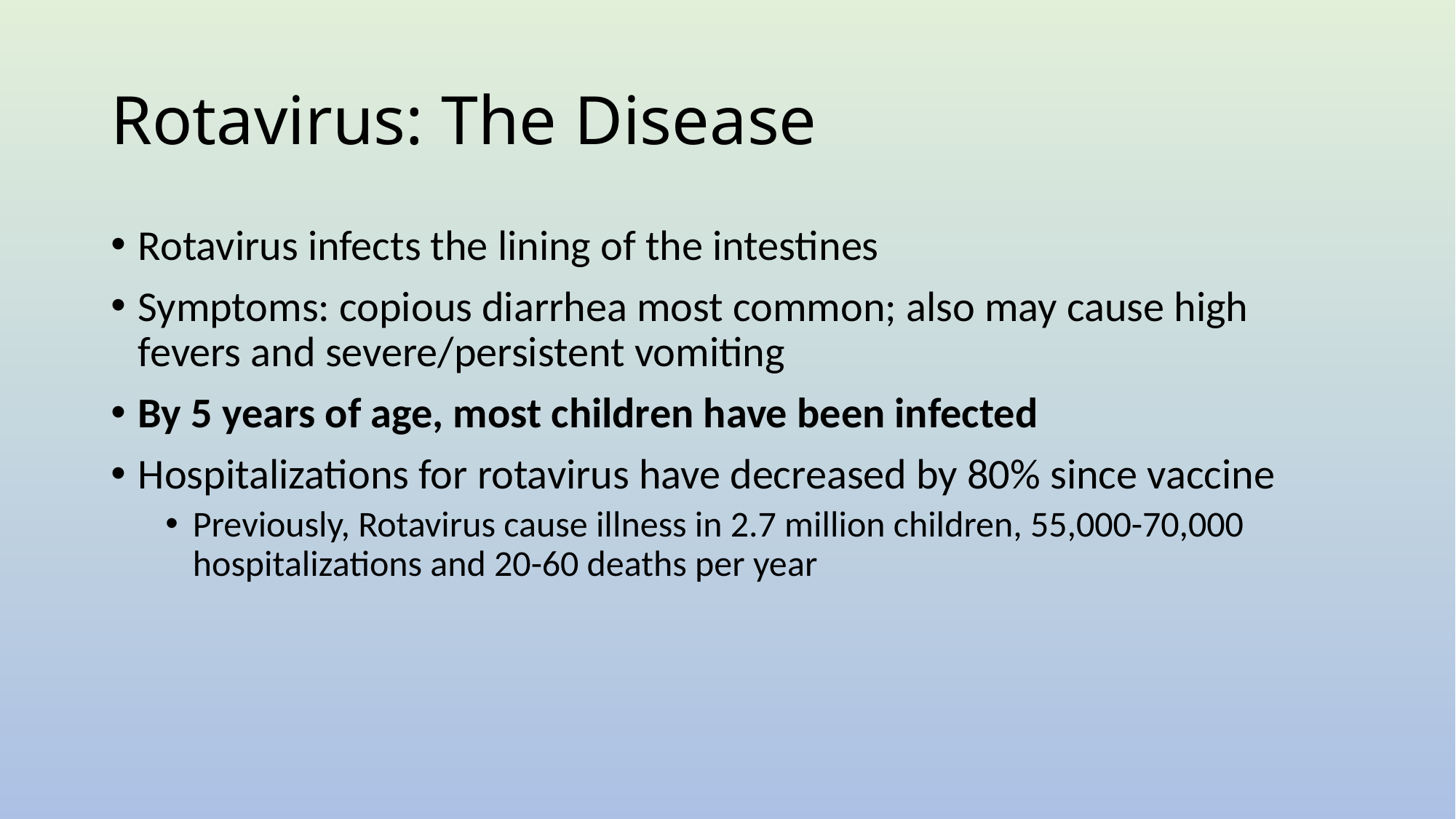

# Rotavirus: The Disease
Rotavirus infects the lining of the intestines
Symptoms: copious diarrhea most common; also may cause high fevers and severe/persistent vomiting
By 5 years of age, most children have been infected
Hospitalizations for rotavirus have decreased by 80% since vaccine
Previously, Rotavirus cause illness in 2.7 million children, 55,000-70,000 hospitalizations and 20-60 deaths per year

## Slide 31
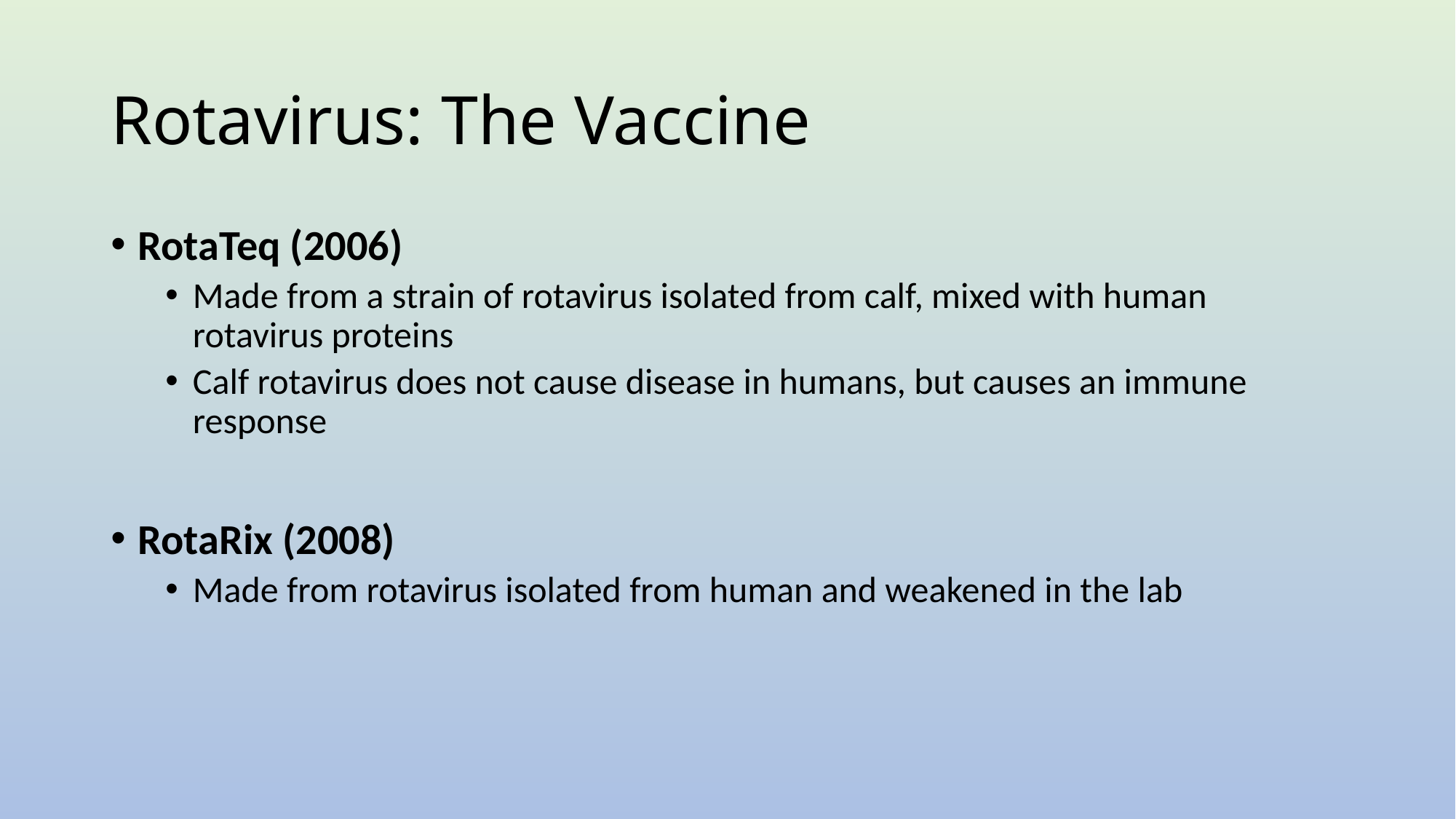

# Rotavirus: The Vaccine
RotaTeq (2006)
Made from a strain of rotavirus isolated from calf, mixed with human rotavirus proteins
Calf rotavirus does not cause disease in humans, but causes an immune response
RotaRix (2008)
Made from rotavirus isolated from human and weakened in the lab

## Slide 32
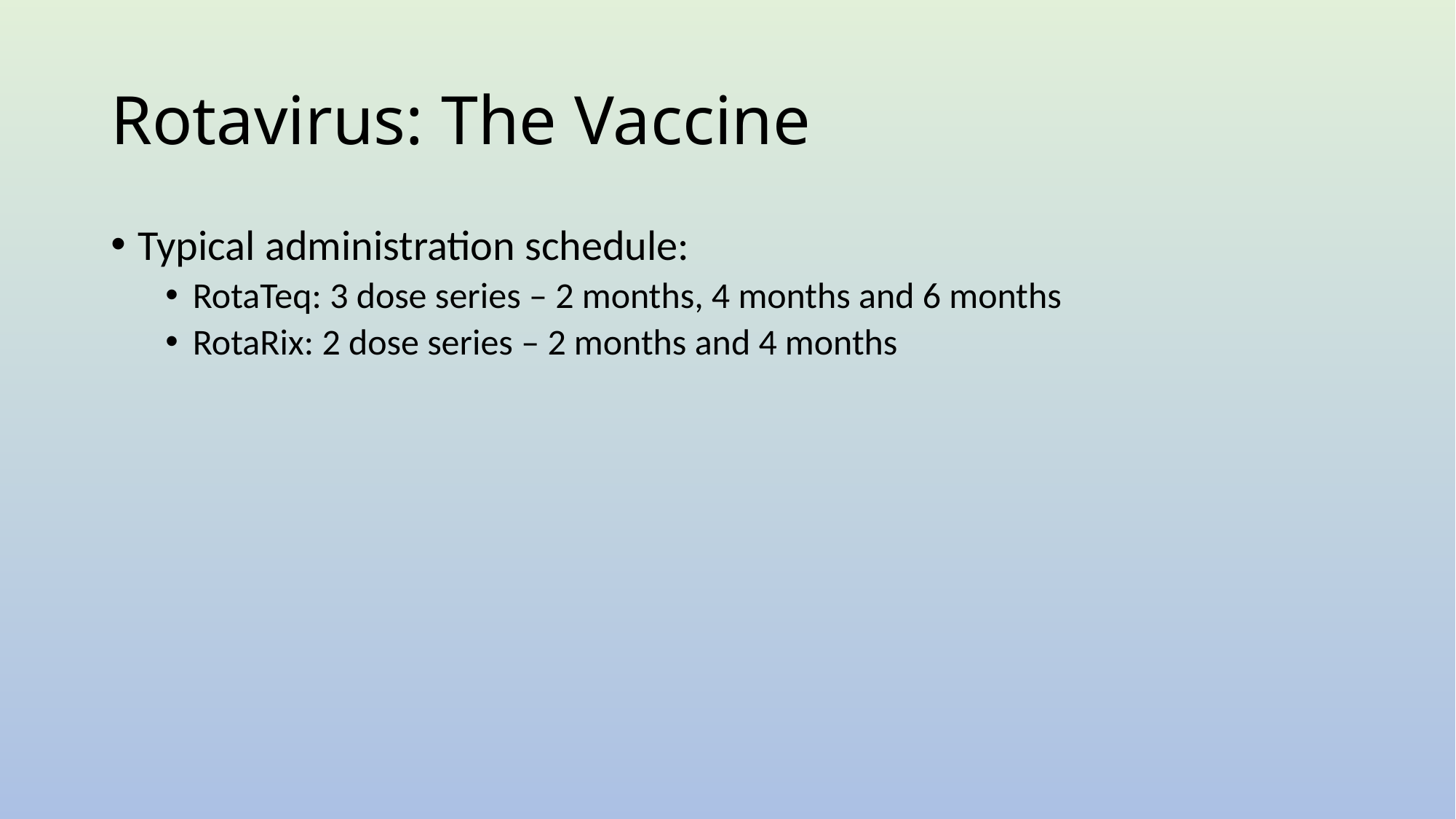

# Rotavirus: The Vaccine
Typical administration schedule:
RotaTeq: 3 dose series – 2 months, 4 months and 6 months
RotaRix: 2 dose series – 2 months and 4 months

## Slide 33
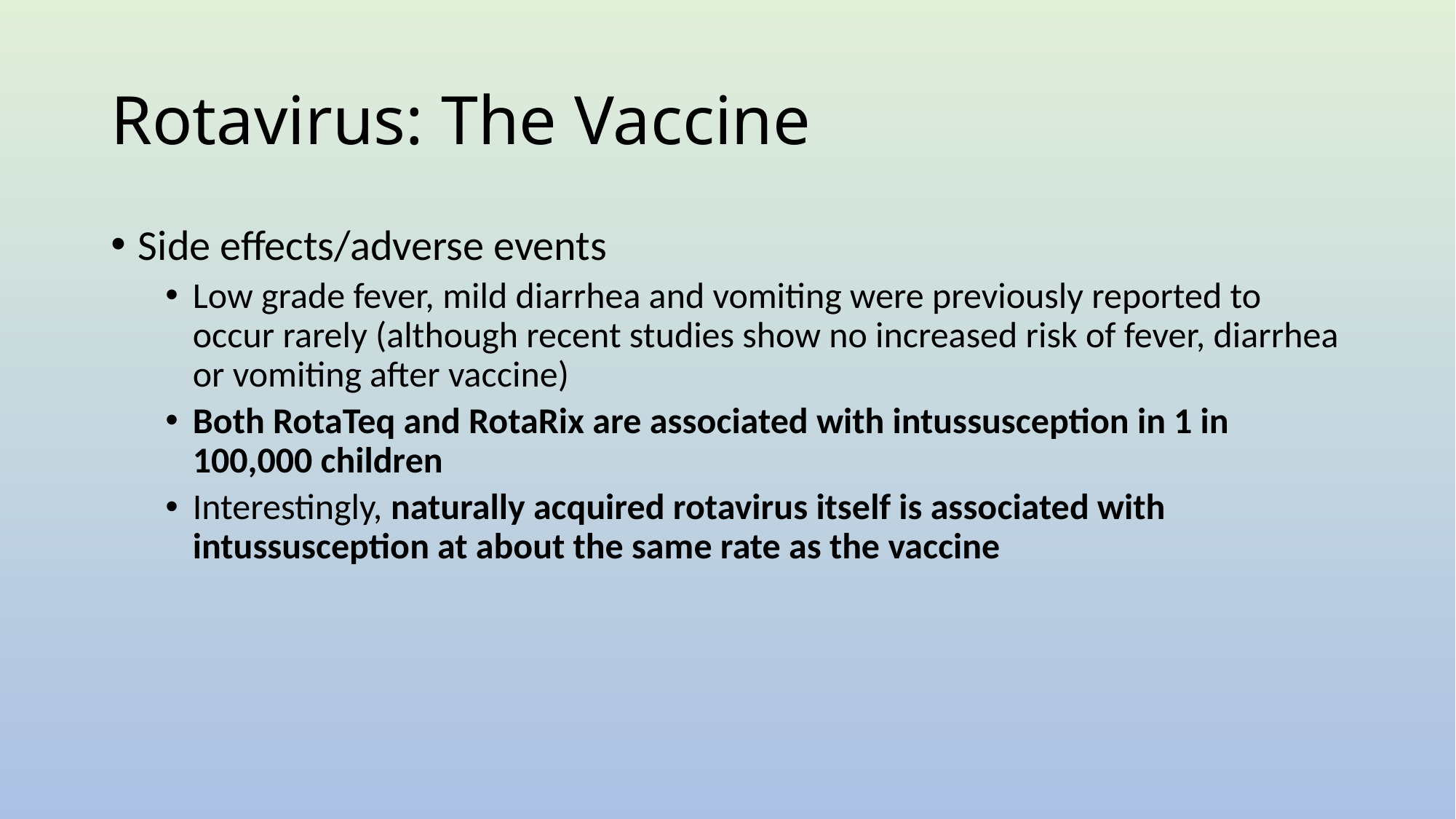

# Rotavirus: The Vaccine
Side effects/adverse events
Low grade fever, mild diarrhea and vomiting were previously reported to occur rarely (although recent studies show no increased risk of fever, diarrhea or vomiting after vaccine)
Both RotaTeq and RotaRix are associated with intussusception in 1 in 100,000 children
Interestingly, naturally acquired rotavirus itself is associated with intussusception at about the same rate as the vaccine

## Slide 34
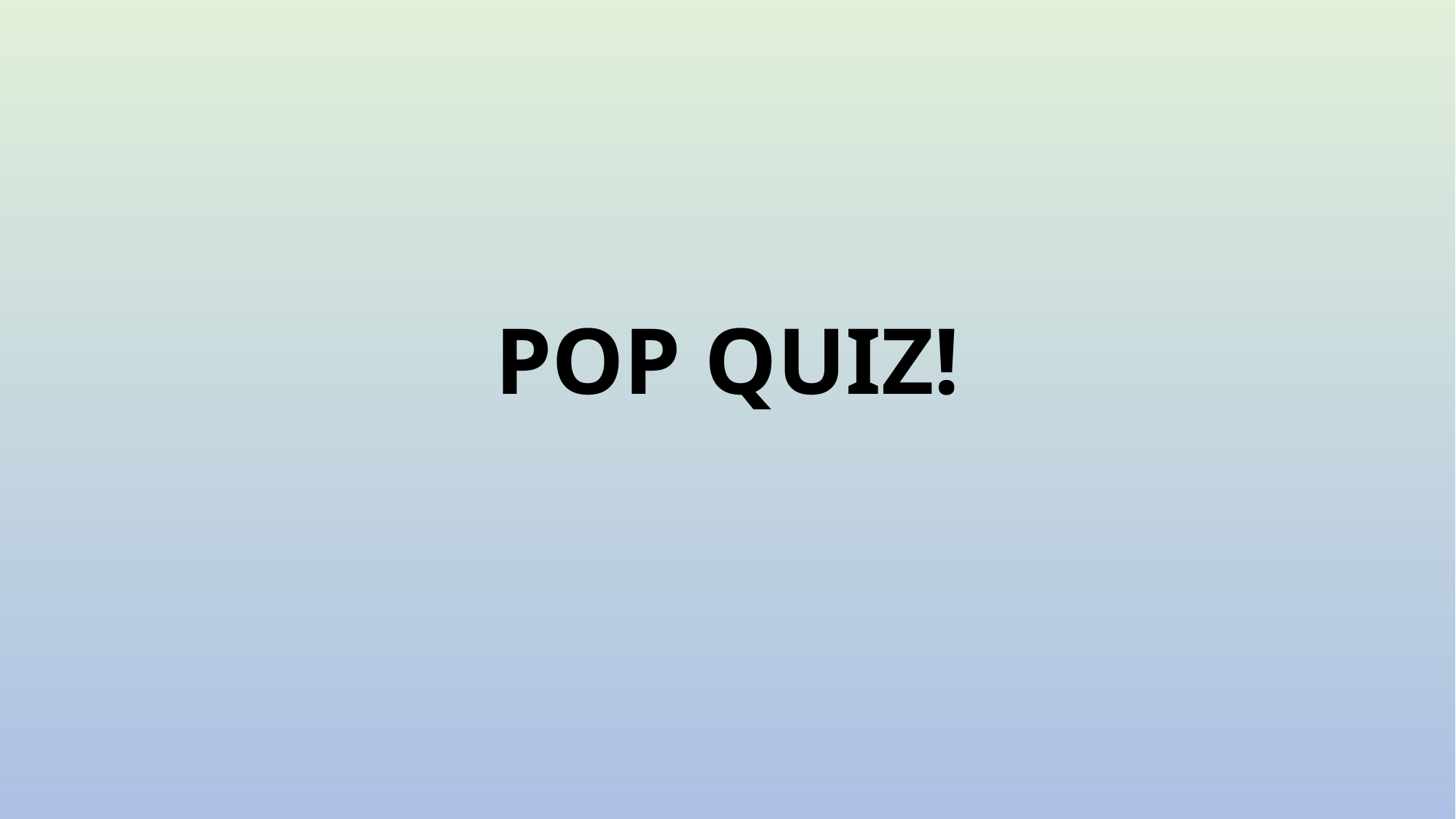

# POP QUIZ!

## Slide 35
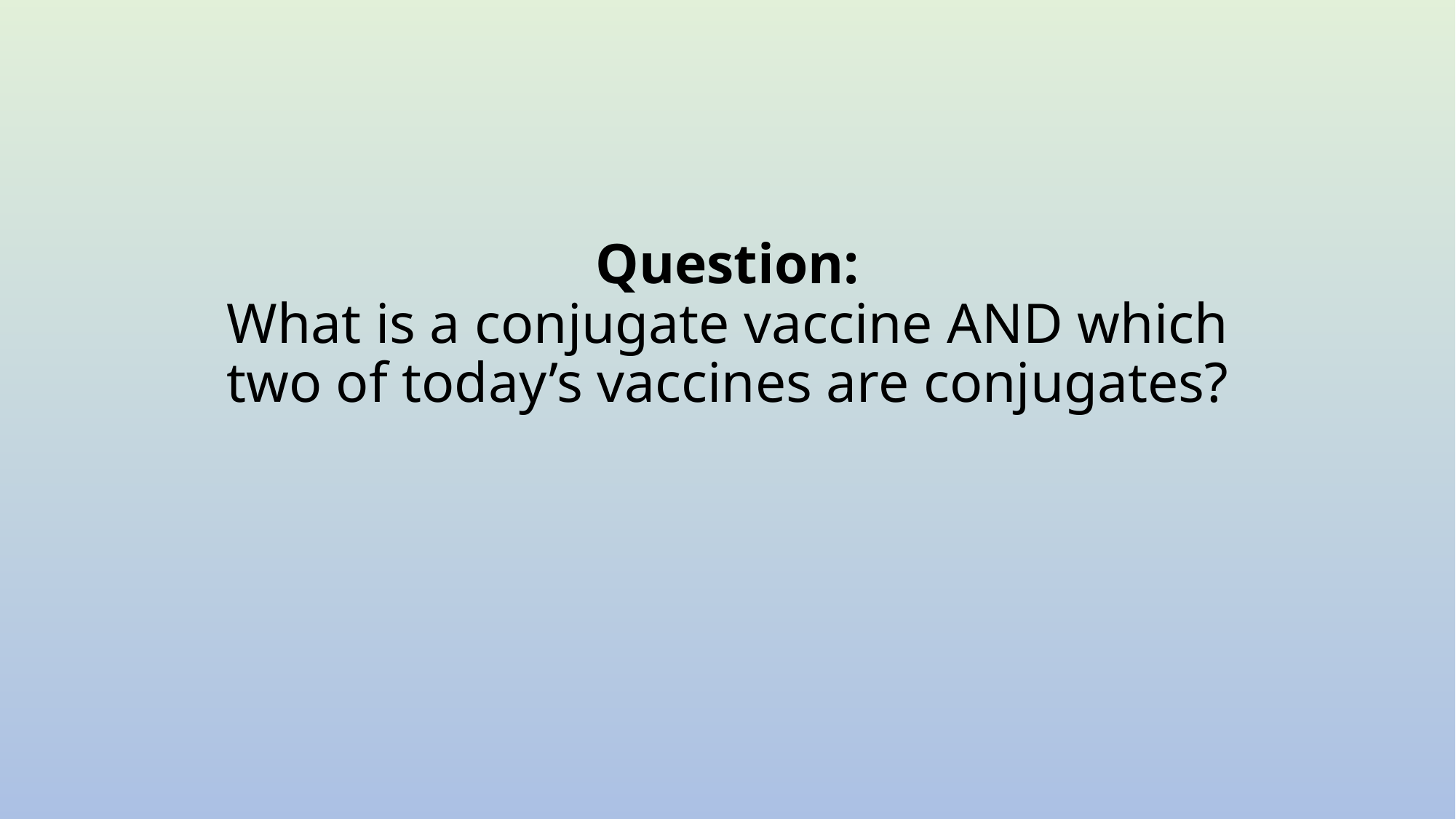

# Question: What is a conjugate vaccine AND which two of today’s vaccines are conjugates?

## Slide 36
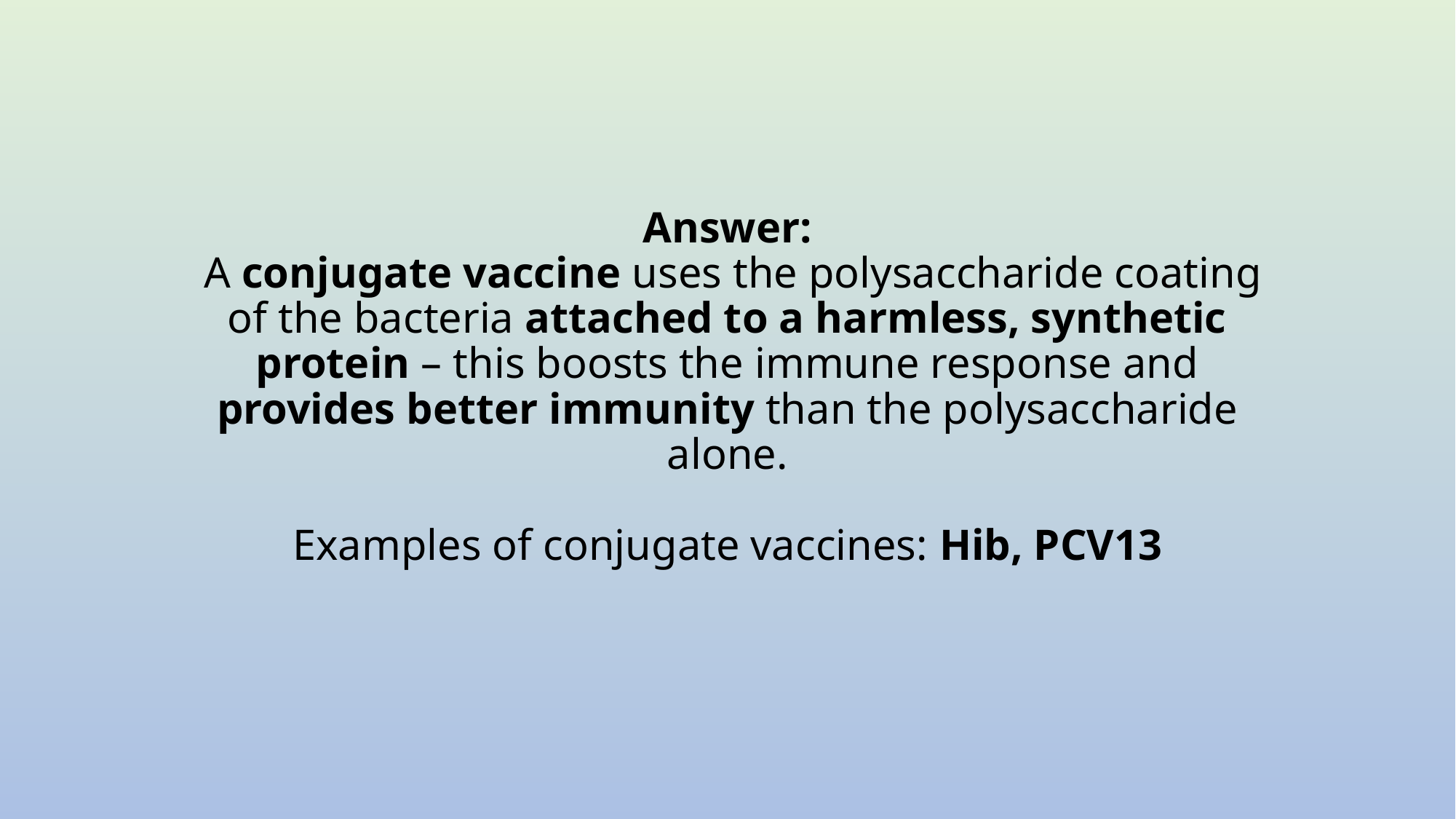

# Answer: A conjugate vaccine uses the polysaccharide coating of the bacteria attached to a harmless, synthetic protein – this boosts the immune response and provides better immunity than the polysaccharide alone. Examples of conjugate vaccines: Hib, PCV13

## Slide 37
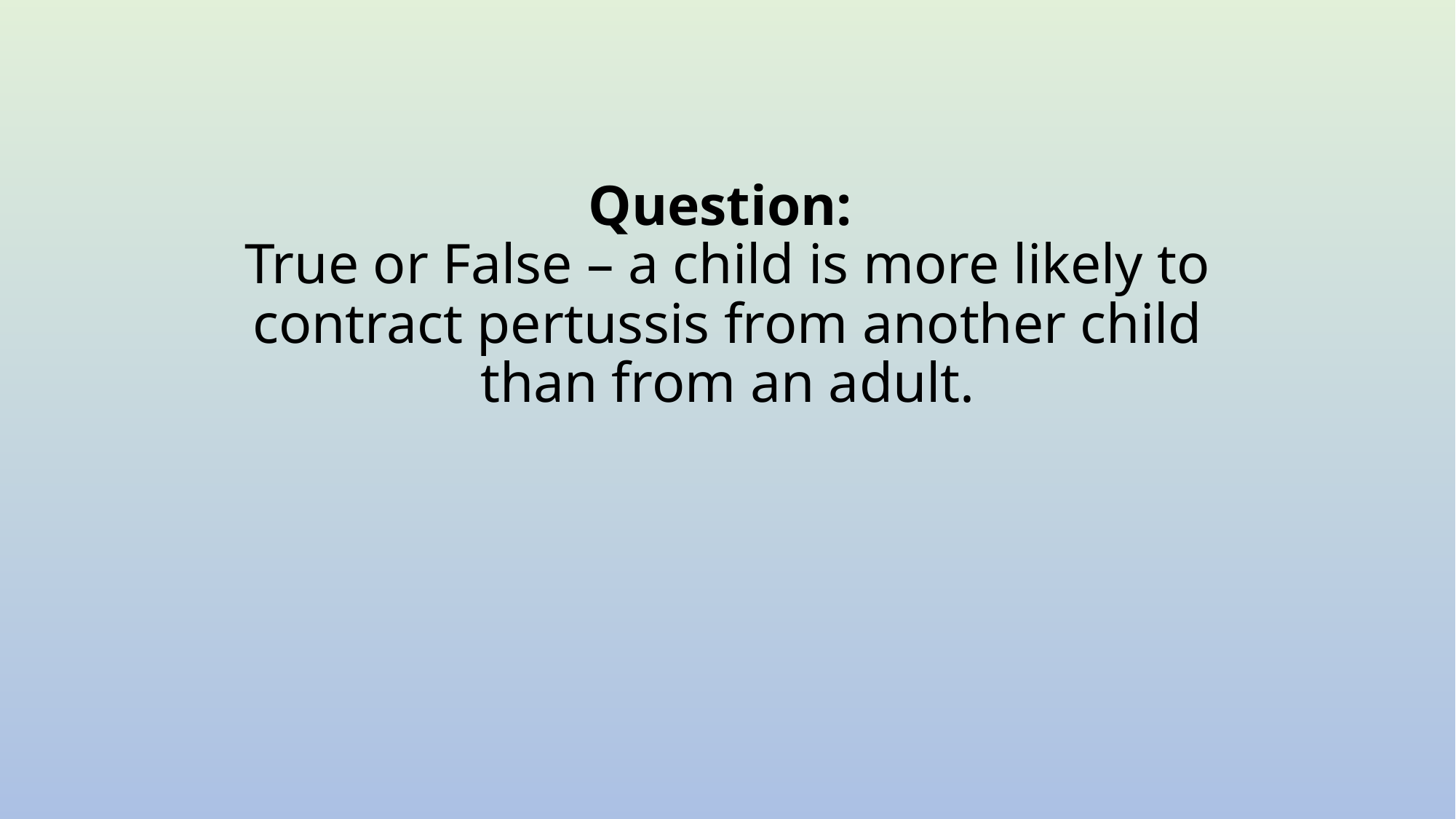

# Question: True or False – a child is more likely to contract pertussis from another child than from an adult.

## Slide 38
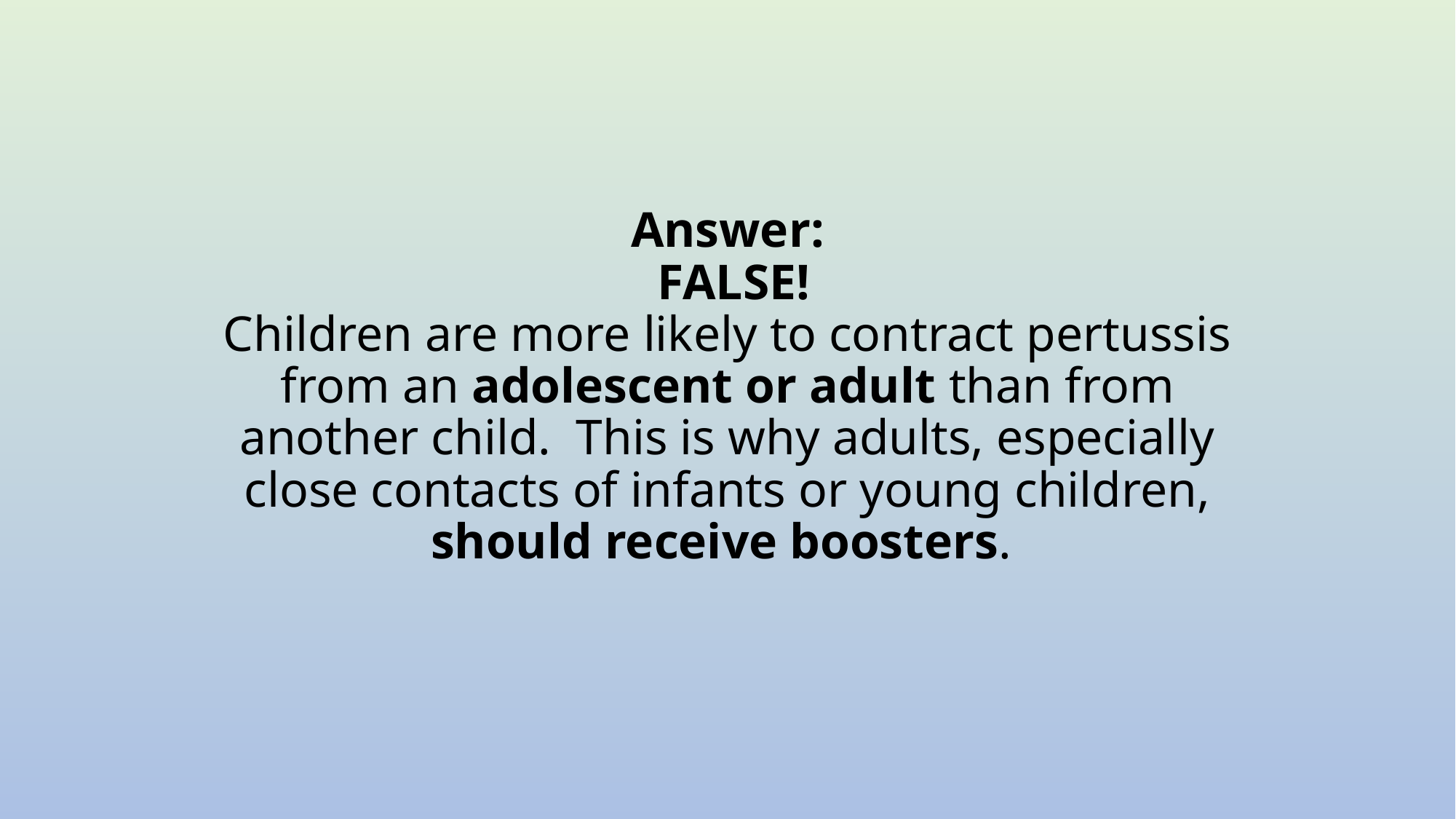

# Answer: FALSE! Children are more likely to contract pertussis from an adolescent or adult than from another child. This is why adults, especially close contacts of infants or young children, should receive boosters.

## Slide 39
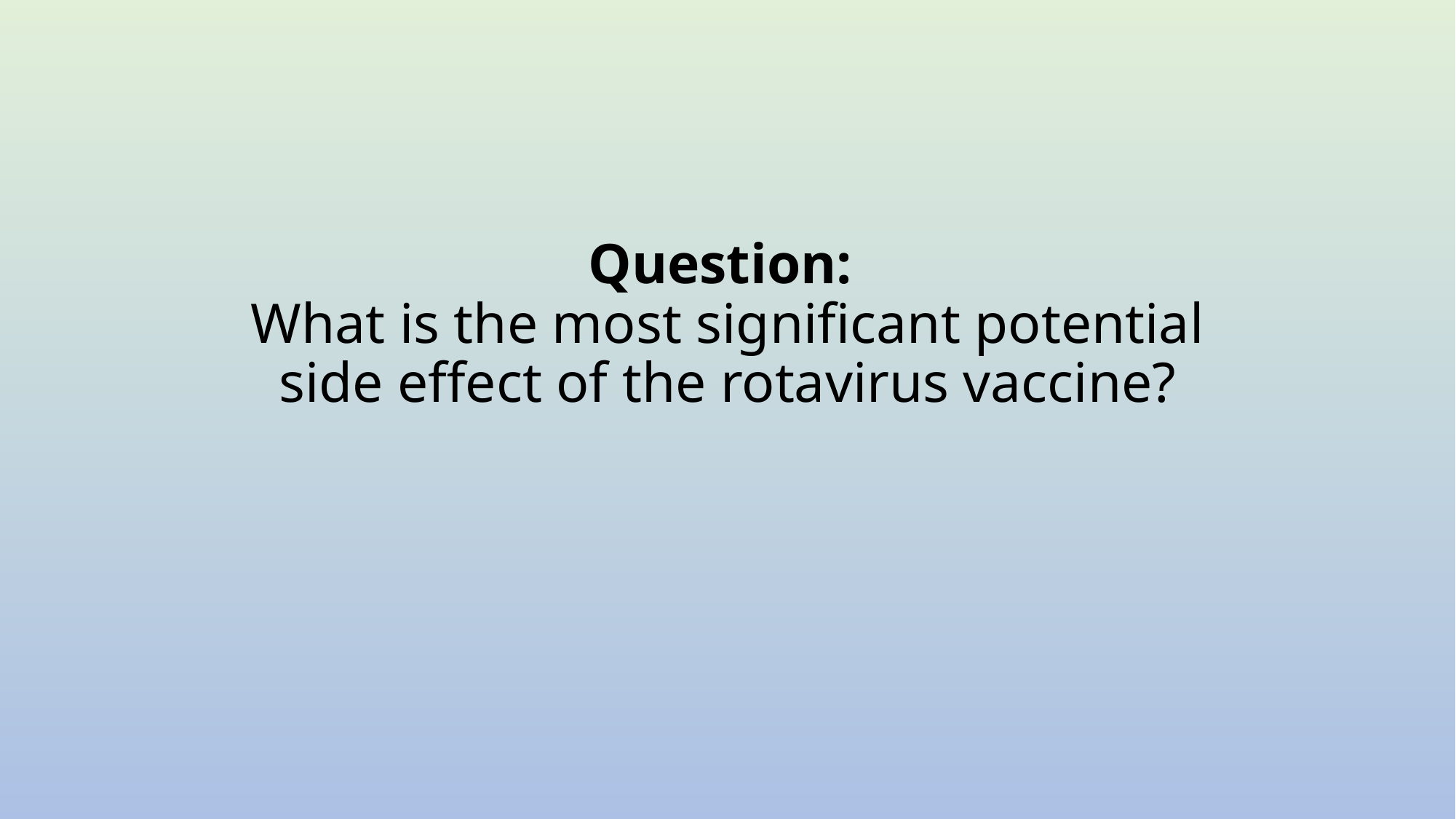

# Question: What is the most significant potential side effect of the rotavirus vaccine?

## Slide 40
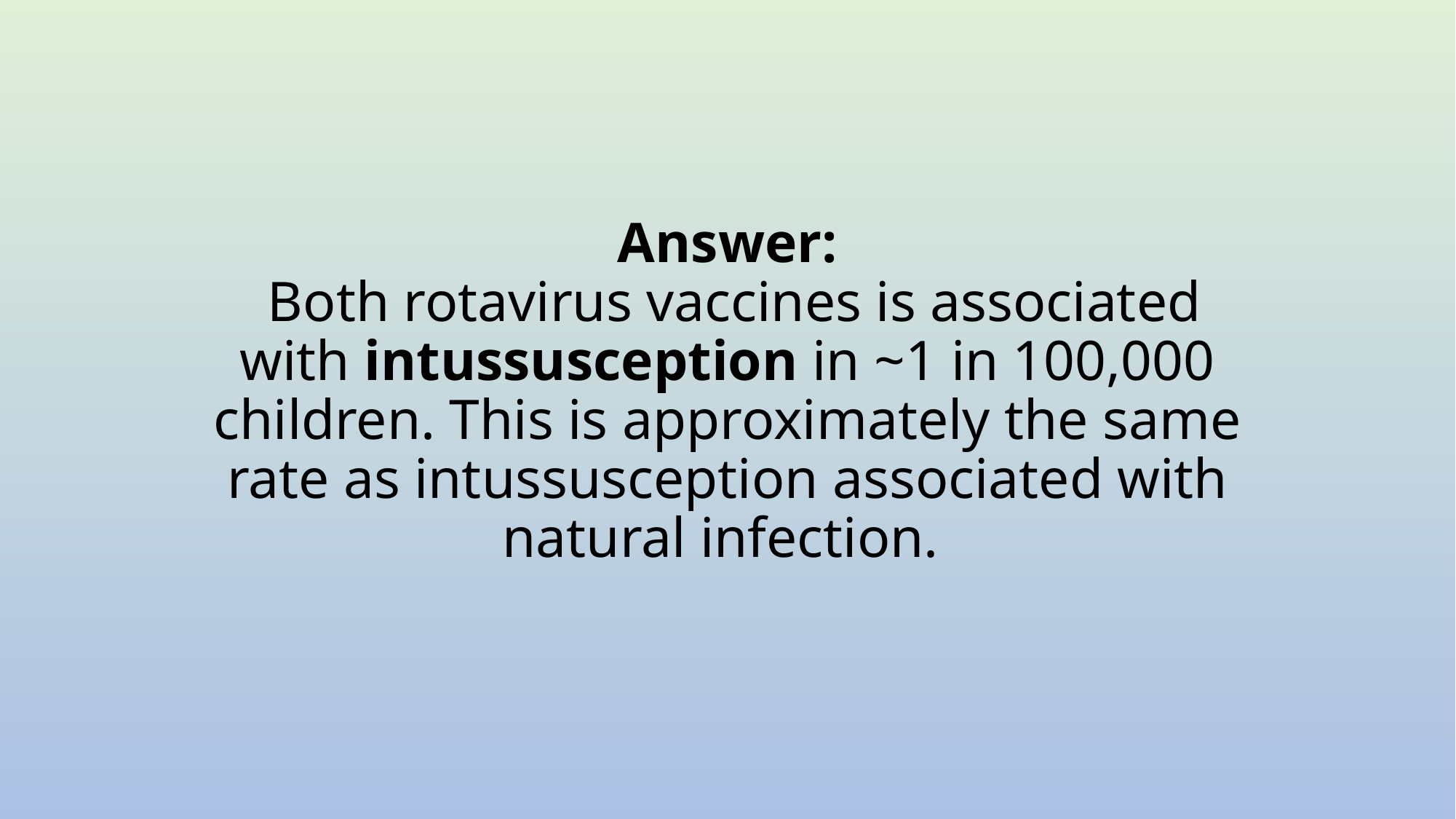

# Answer: Both rotavirus vaccines is associated with intussusception in ~1 in 100,000 children. This is approximately the same rate as intussusception associated with natural infection.

## Slide 41
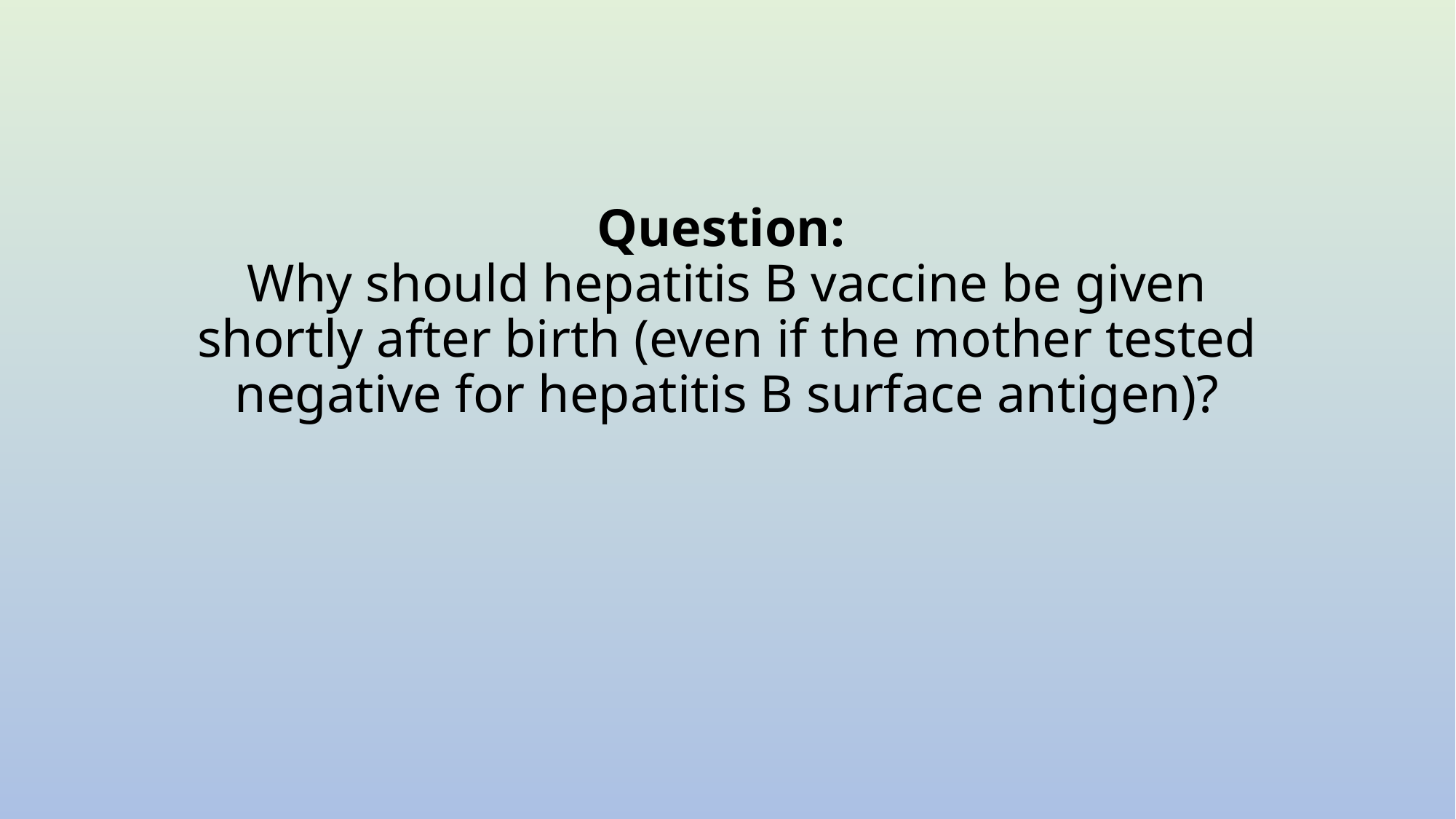

# Question: Why should hepatitis B vaccine be given shortly after birth (even if the mother tested negative for hepatitis B surface antigen)?

## Slide 42
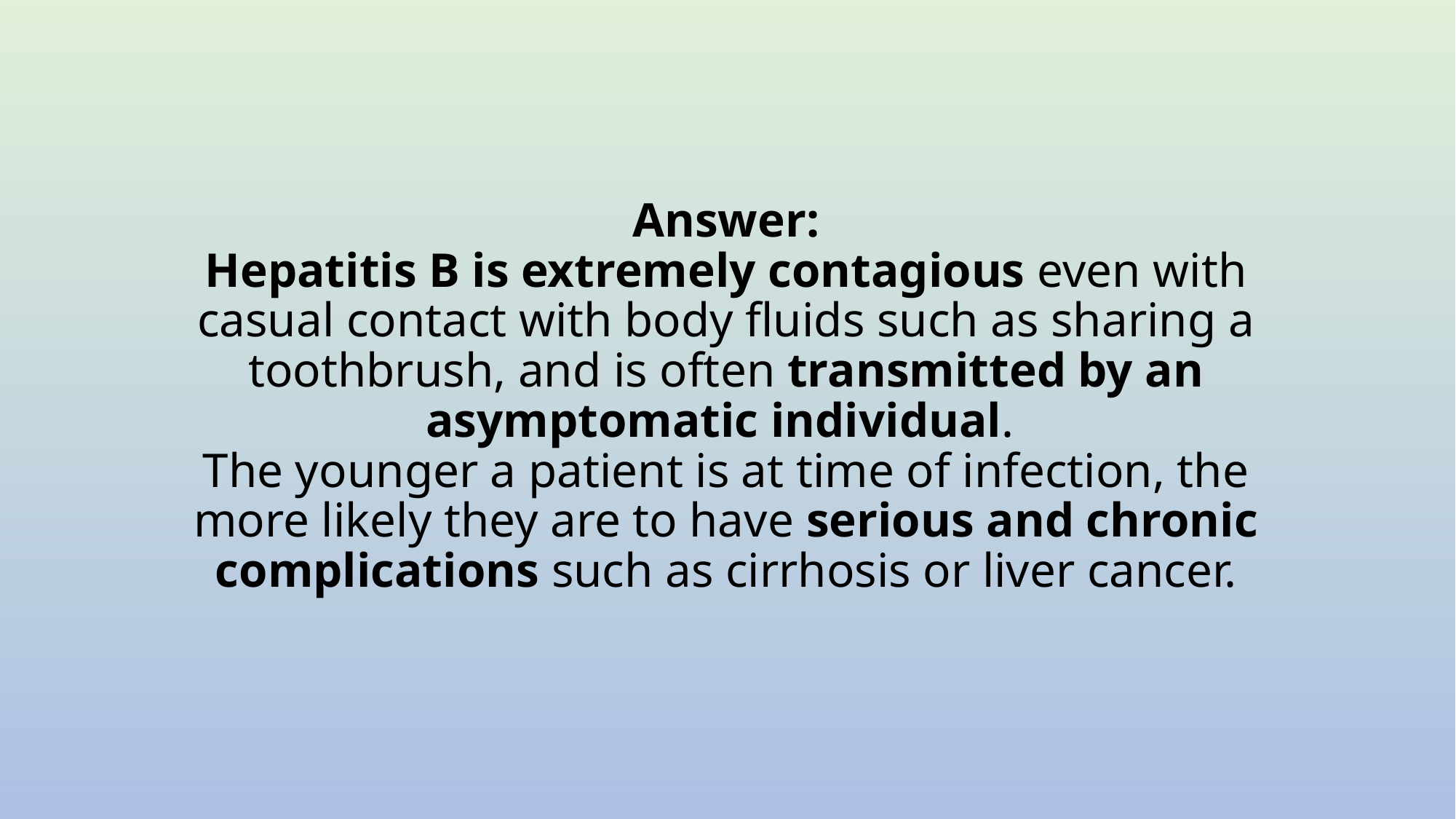

# Answer: Hepatitis B is extremely contagious even with casual contact with body fluids such as sharing a toothbrush, and is often transmitted by an asymptomatic individual. The younger a patient is at time of infection, the more likely they are to have serious and chronic complications such as cirrhosis or liver cancer.

## Slide 43
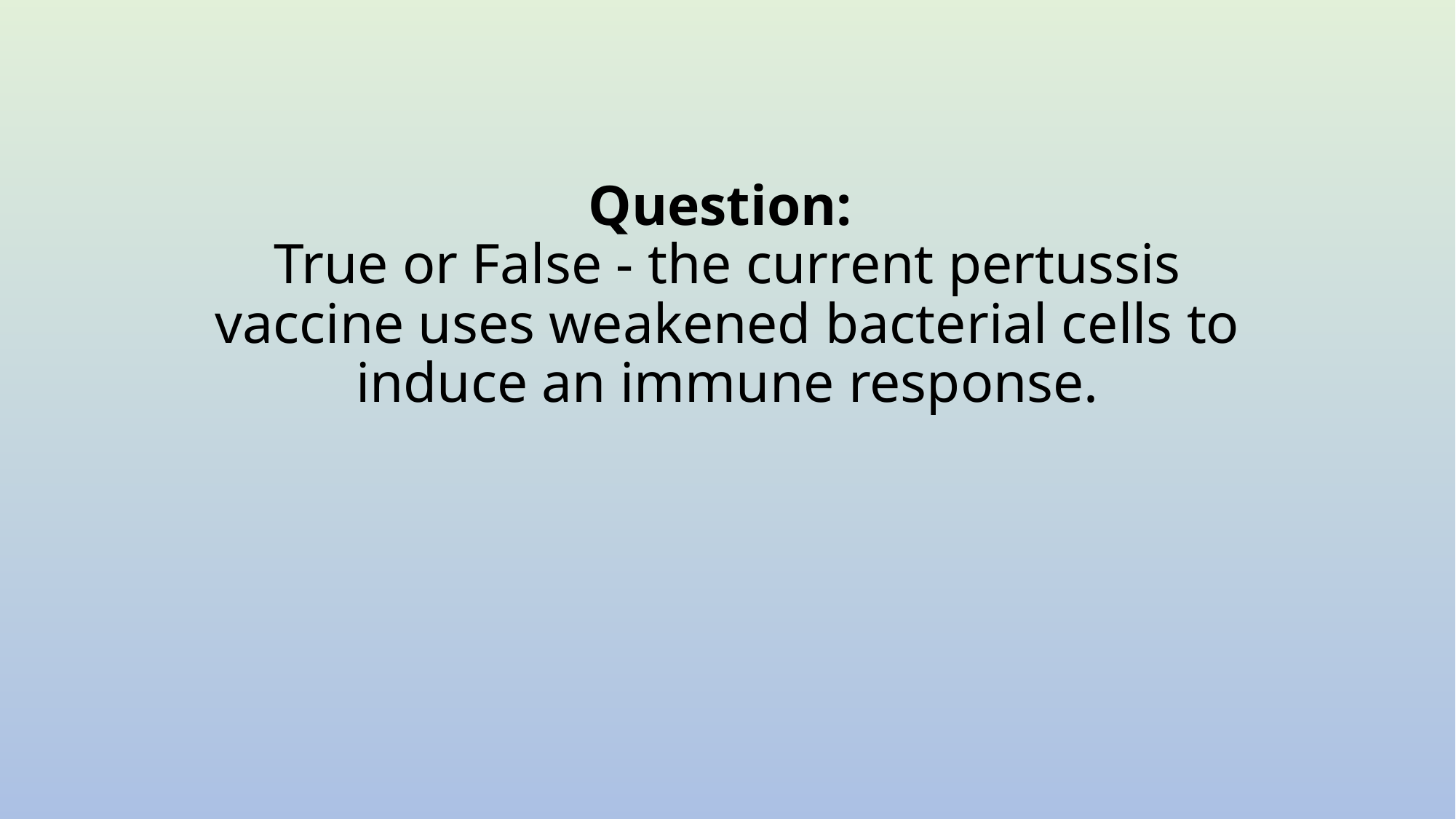

# Question: True or False - the current pertussis vaccine uses weakened bacterial cells to induce an immune response.

## Slide 44
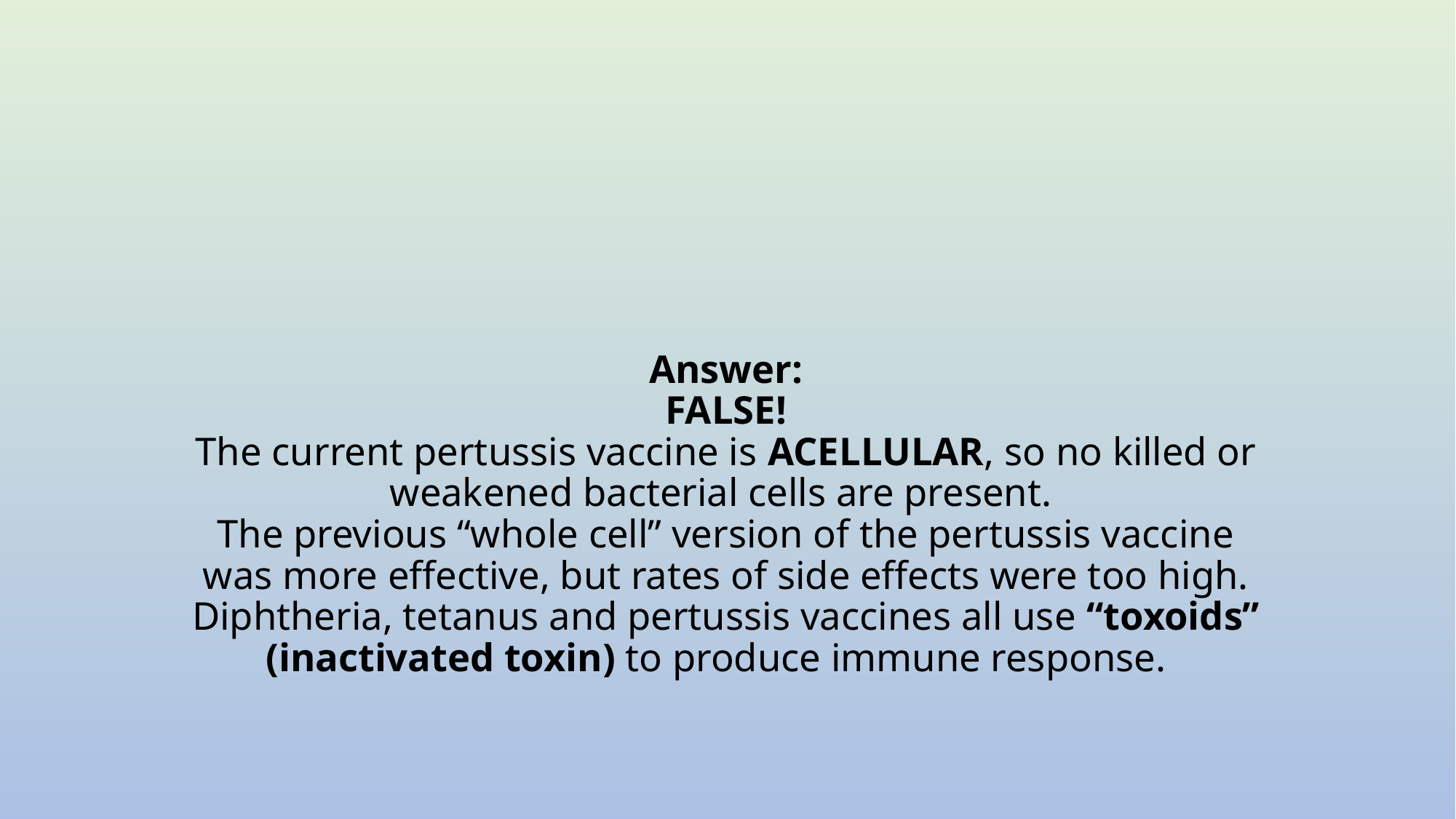

# Answer: FALSE! The current pertussis vaccine is ACELLULAR, so no killed or weakened bacterial cells are present. The previous “whole cell” version of the pertussis vaccine was more effective, but rates of side effects were too high. Diphtheria, tetanus and pertussis vaccines all use “toxoids” (inactivated toxin) to produce immune response.

## Slide 45
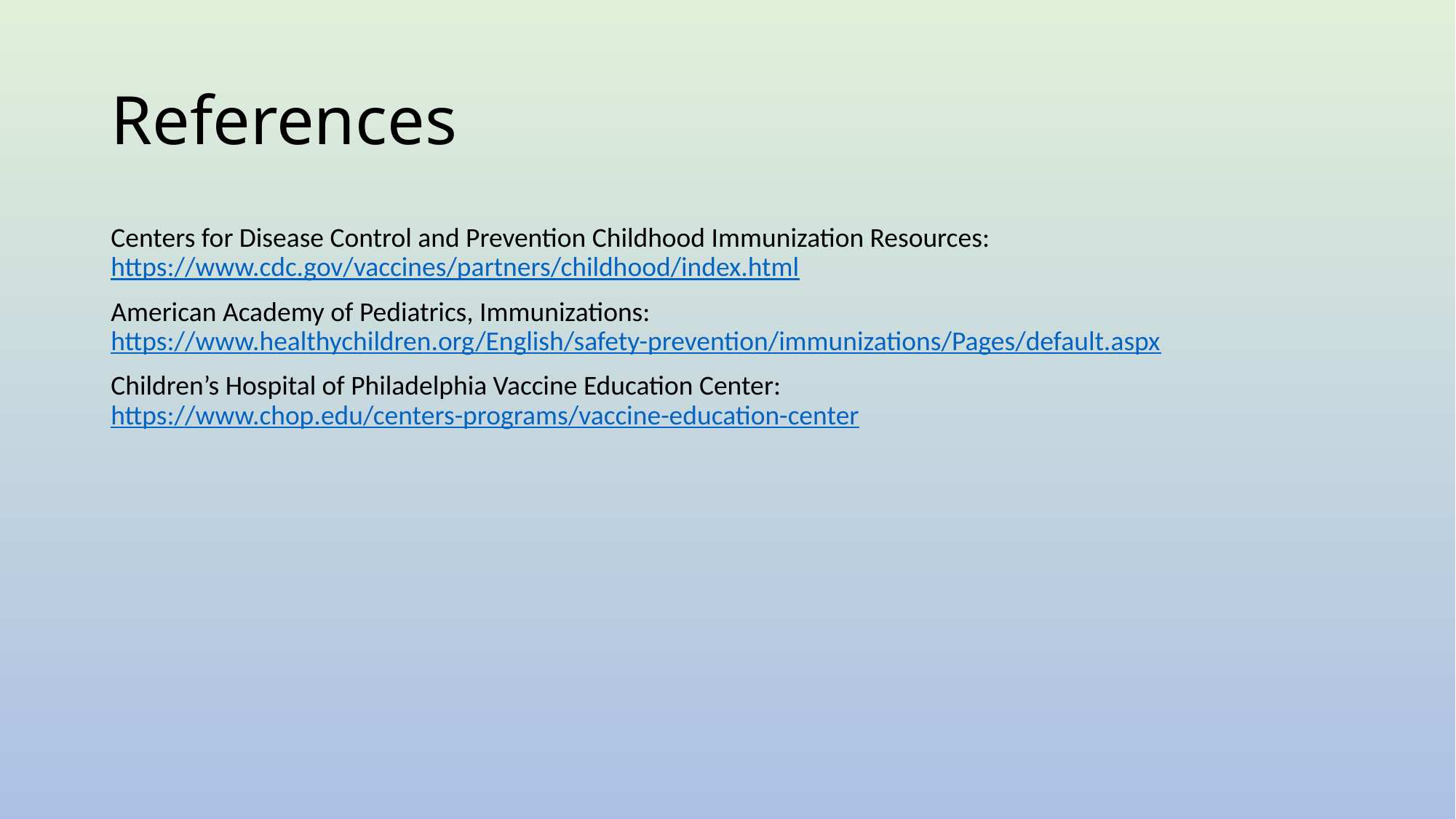

# References
Centers for Disease Control and Prevention Childhood Immunization Resources: https://www.cdc.gov/vaccines/partners/childhood/index.html
American Academy of Pediatrics, Immunizations: https://www.healthychildren.org/English/safety-prevention/immunizations/Pages/default.aspx
Children’s Hospital of Philadelphia Vaccine Education Center: https://www.chop.edu/centers-programs/vaccine-education-center
