# Supplementary material for: Addressing Vaccine Hesitancy Through a Comprehensive Resident Vaccine Curriculum
Source: MedEdPORTAL. 2022 Dec 27;18:11292. doi: 10.15766/mep_2374-8265.11292 (PMC9792628; doi:10.15766/mep_2374-8265.11292)
Supplement: Supplementary file 1 — Vaccine Curriculum Facilitator Guide.docxVaccines Part 1.pptxVaccines Part 2.pptxVaccines Part 3 - Myths and Facts.pptxVaccines Part 4 - Communication Skills.pptxVaccine Hesitancy Communication Cases.docxVaccine Pretest.docxVaccine Posttest.docxPre- and Posttest Answer Key.docxSP Case and Notes for SP.docxSP Case Development Tool.docxSP Case - Learner Version.docxSP Assessment Checklist.docx [file mep_2374-8265.11292-s001.zip › C. Vaccines Part 2.pptx]

## Slide 1
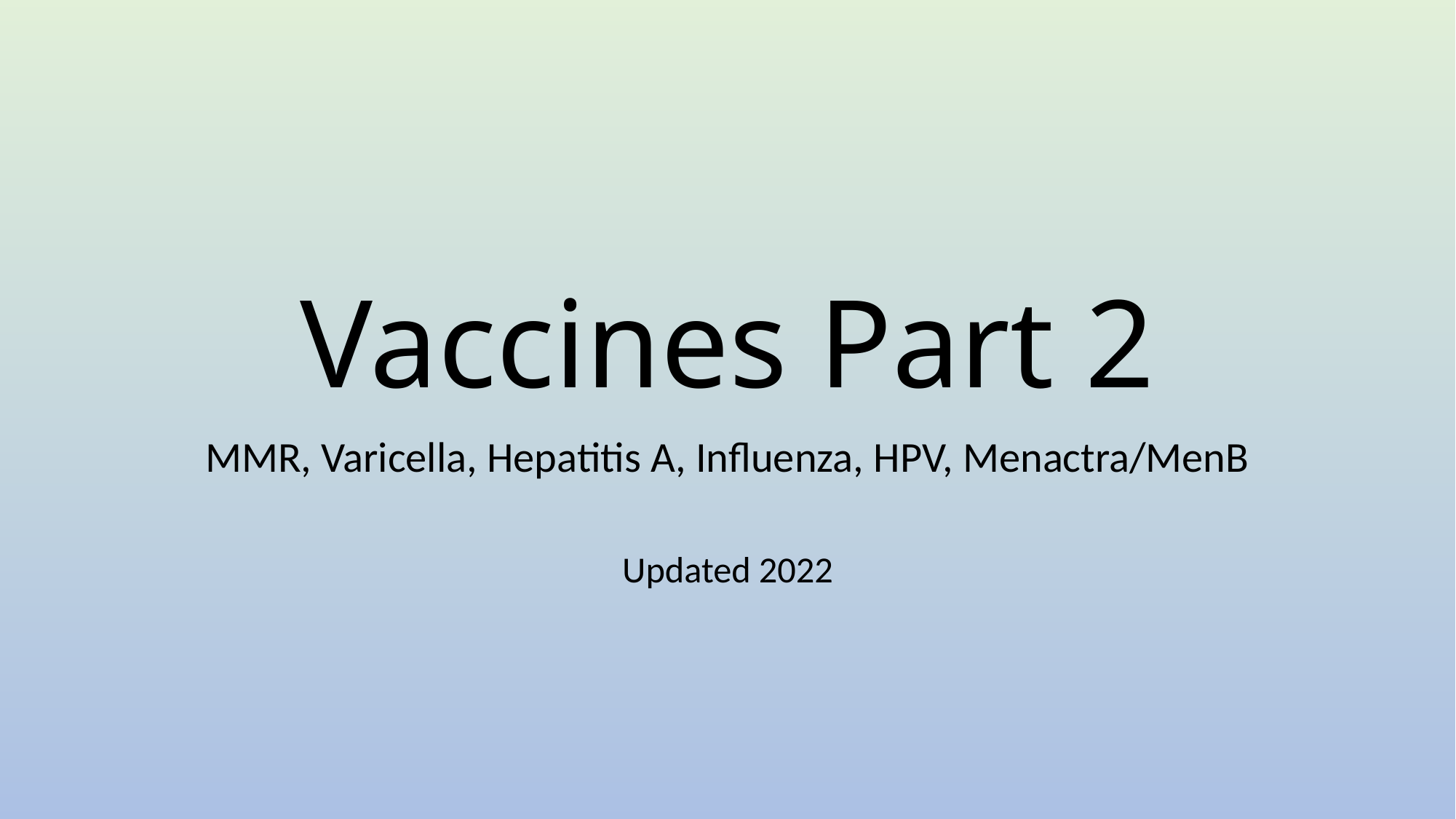

# Vaccines Part 2
MMR, Varicella, Hepatitis A, Influenza, HPV, Menactra/MenB
Updated 2022

## Slide 2
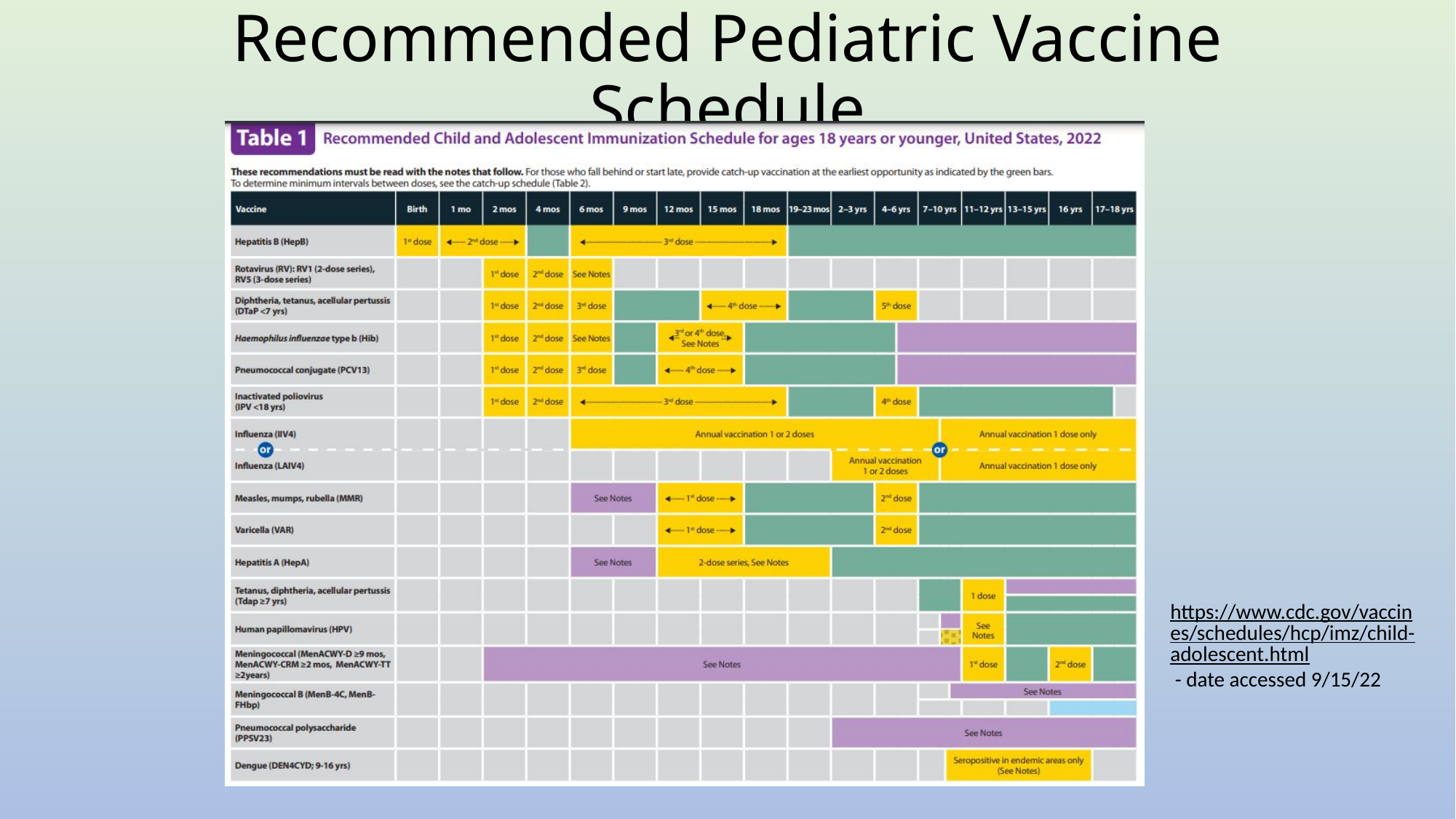

# Recommended Pediatric Vaccine Schedule
https://www.cdc.gov/vaccines/schedules/hcp/imz/child-adolescent.html - date accessed 9/15/22

## Slide 3
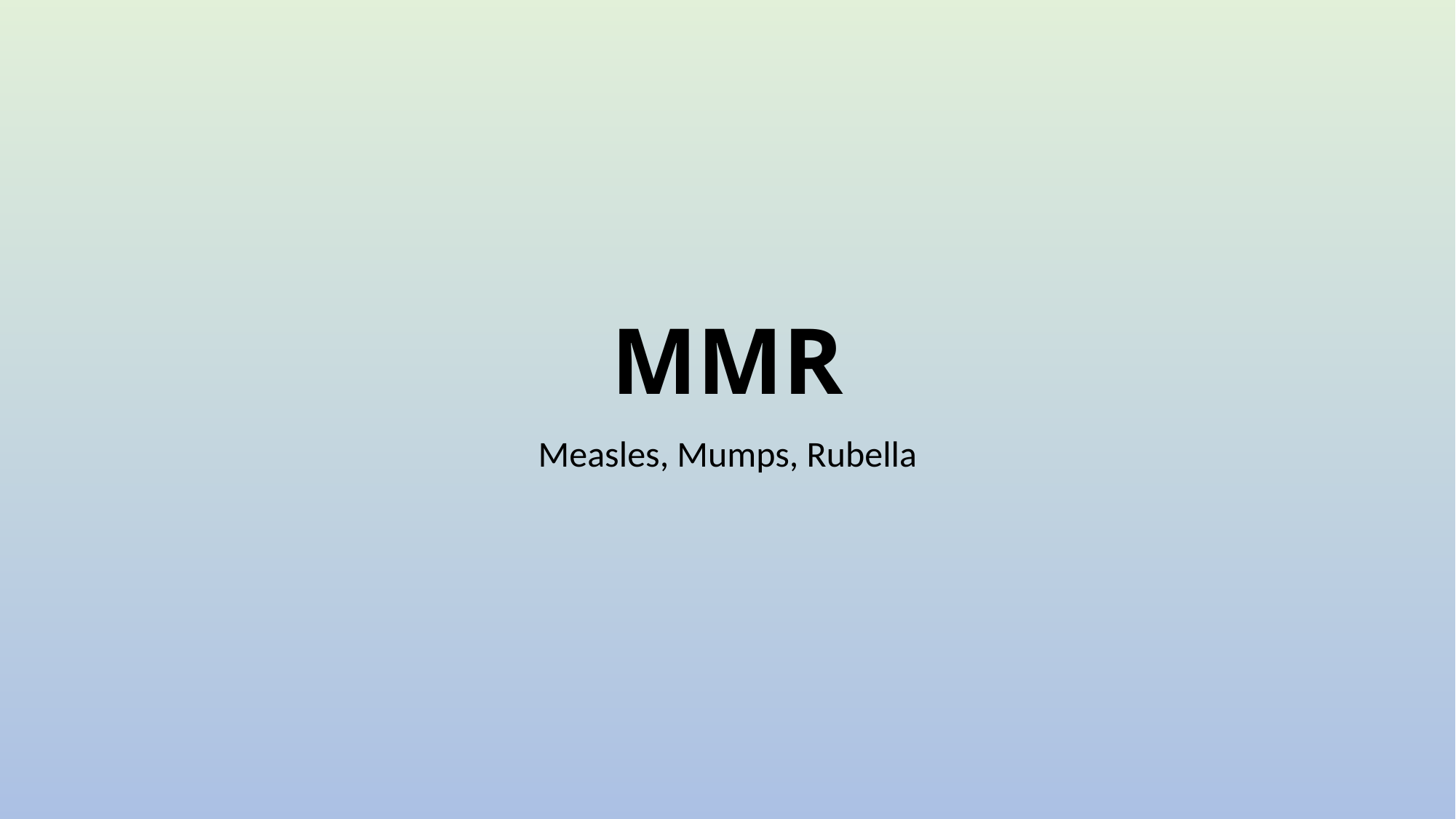

# MMR
Measles, Mumps, Rubella

## Slide 4
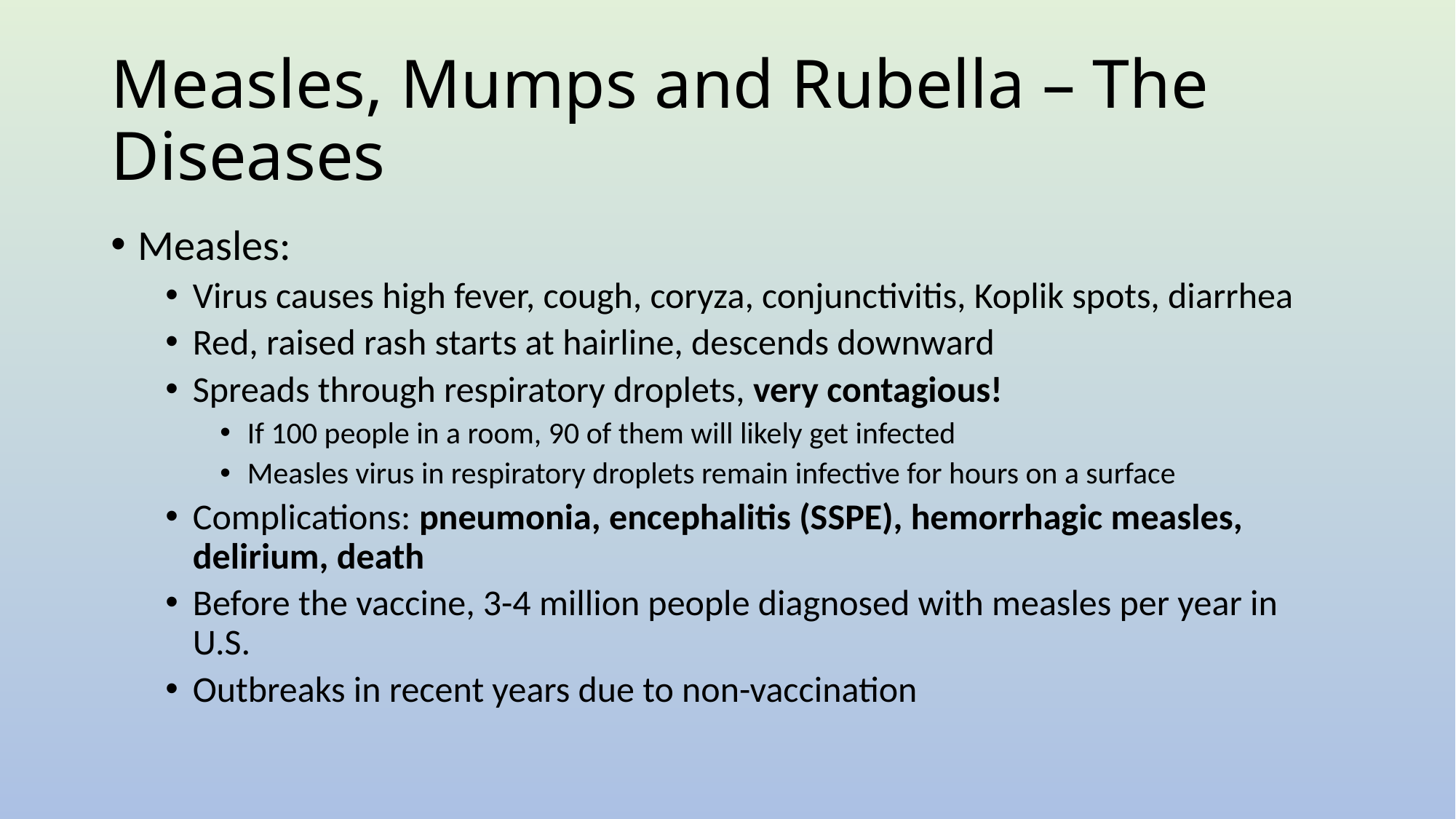

# Measles, Mumps and Rubella – The Diseases
Measles:
Virus causes high fever, cough, coryza, conjunctivitis, Koplik spots, diarrhea
Red, raised rash starts at hairline, descends downward
Spreads through respiratory droplets, very contagious!
If 100 people in a room, 90 of them will likely get infected
Measles virus in respiratory droplets remain infective for hours on a surface
Complications: pneumonia, encephalitis (SSPE), hemorrhagic measles, delirium, death
Before the vaccine, 3-4 million people diagnosed with measles per year in U.S.
Outbreaks in recent years due to non-vaccination

## Slide 5
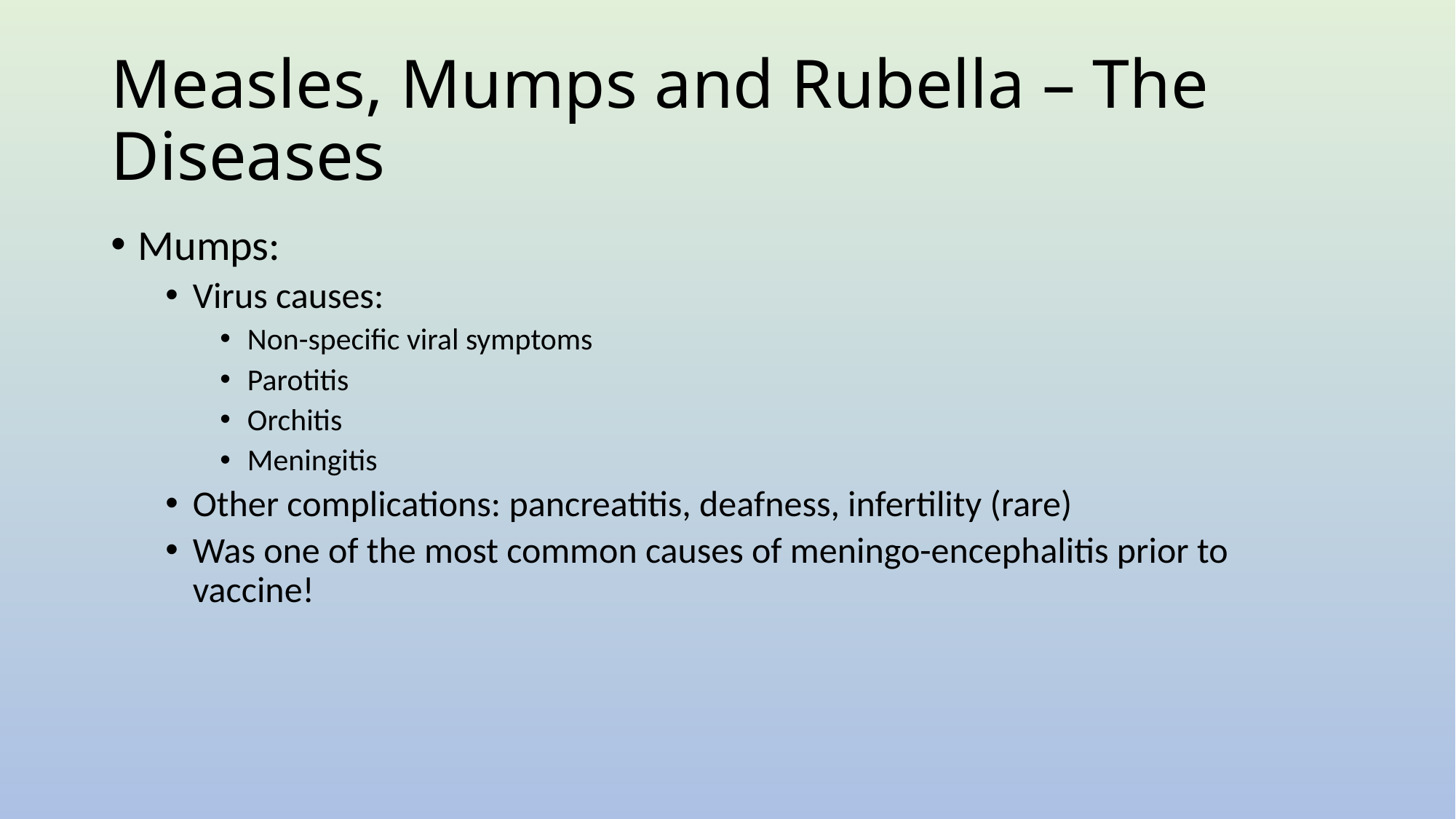

# Measles, Mumps and Rubella – The Diseases
Mumps:
Virus causes:
Non-specific viral symptoms
Parotitis
Orchitis
Meningitis
Other complications: pancreatitis, deafness, infertility (rare)
Was one of the most common causes of meningo-encephalitis prior to vaccine!

## Slide 6
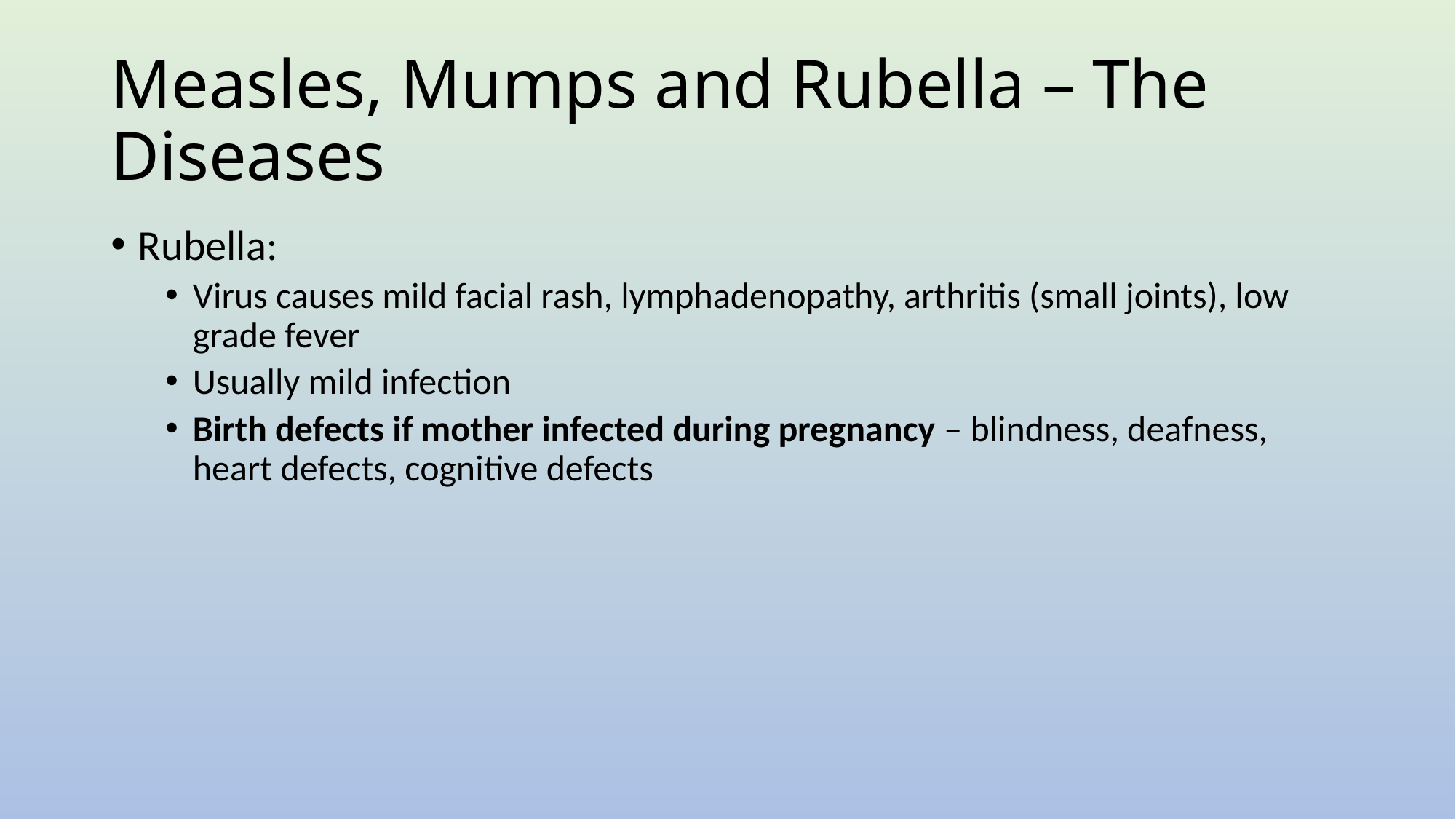

# Measles, Mumps and Rubella – The Diseases
Rubella:
Virus causes mild facial rash, lymphadenopathy, arthritis (small joints), low grade fever
Usually mild infection
Birth defects if mother infected during pregnancy – blindness, deafness, heart defects, cognitive defects

## Slide 7
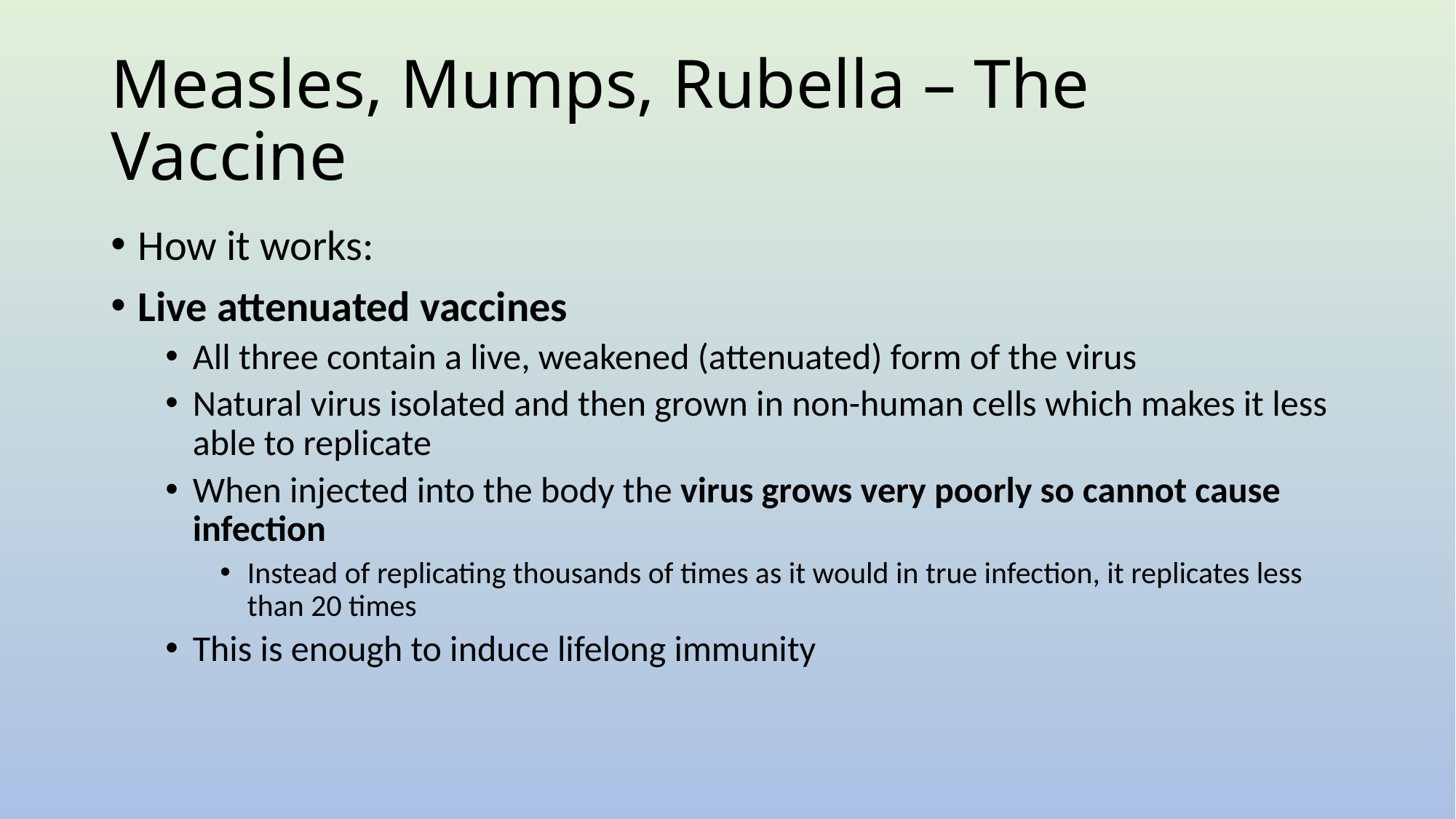

# Measles, Mumps, Rubella – The Vaccine
How it works:
Live attenuated vaccines
All three contain a live, weakened (attenuated) form of the virus
Natural virus isolated and then grown in non-human cells which makes it less able to replicate
When injected into the body the virus grows very poorly so cannot cause infection
Instead of replicating thousands of times as it would in true infection, it replicates less than 20 times
This is enough to induce lifelong immunity

## Slide 8
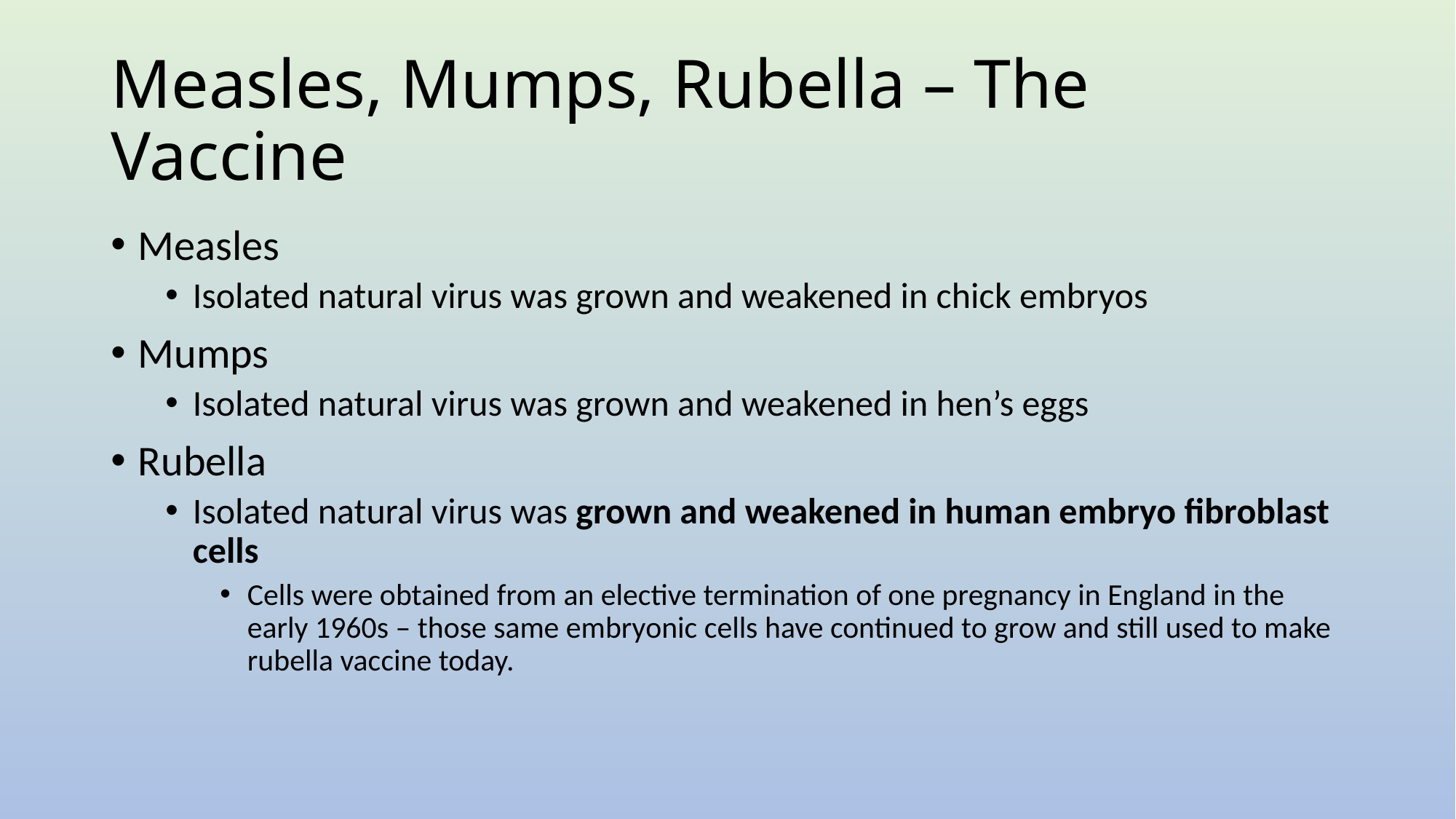

# Measles, Mumps, Rubella – The Vaccine
Measles
Isolated natural virus was grown and weakened in chick embryos
Mumps
Isolated natural virus was grown and weakened in hen’s eggs
Rubella
Isolated natural virus was grown and weakened in human embryo fibroblast cells
Cells were obtained from an elective termination of one pregnancy in England in the early 1960s – those same embryonic cells have continued to grow and still used to make rubella vaccine today.

## Slide 9
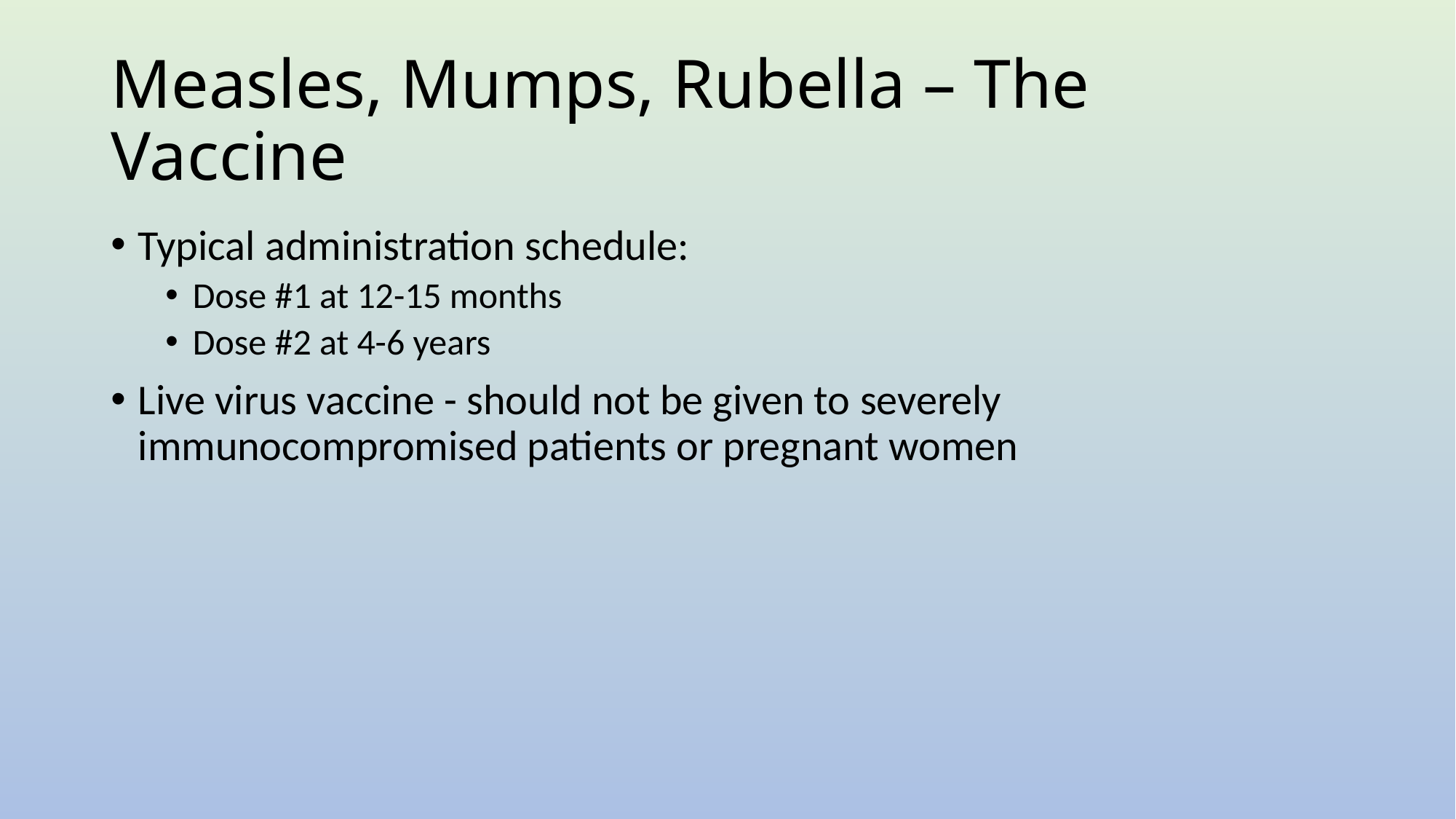

# Measles, Mumps, Rubella – The Vaccine
Typical administration schedule:
Dose #1 at 12-15 months
Dose #2 at 4-6 years
Live virus vaccine - should not be given to severely immunocompromised patients or pregnant women

## Slide 10
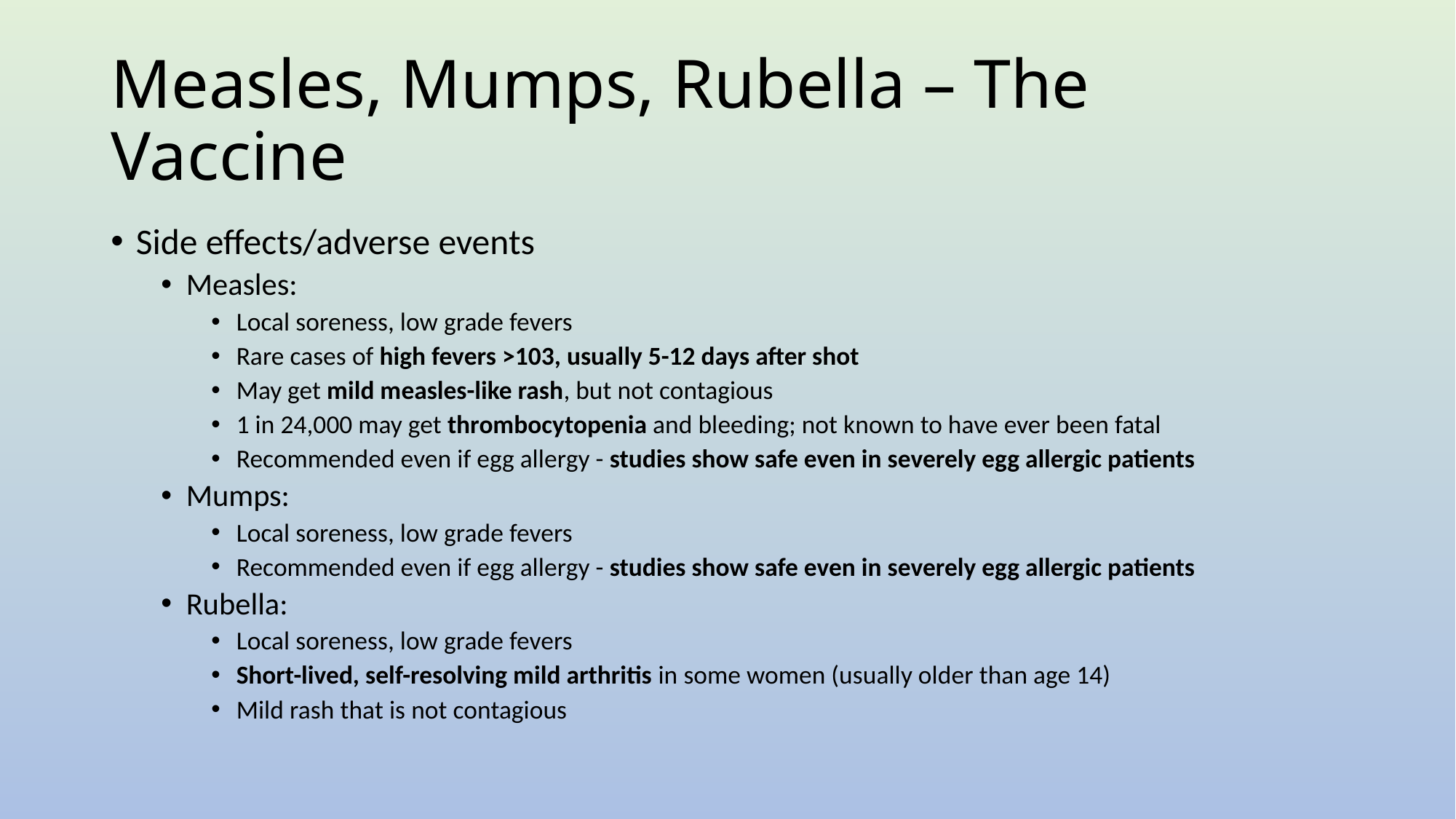

# Measles, Mumps, Rubella – The Vaccine
Side effects/adverse events
Measles:
Local soreness, low grade fevers
Rare cases of high fevers >103, usually 5-12 days after shot
May get mild measles-like rash, but not contagious
1 in 24,000 may get thrombocytopenia and bleeding; not known to have ever been fatal
Recommended even if egg allergy - studies show safe even in severely egg allergic patients
Mumps:
Local soreness, low grade fevers
Recommended even if egg allergy - studies show safe even in severely egg allergic patients
Rubella:
Local soreness, low grade fevers
Short-lived, self-resolving mild arthritis in some women (usually older than age 14)
Mild rash that is not contagious

## Slide 11
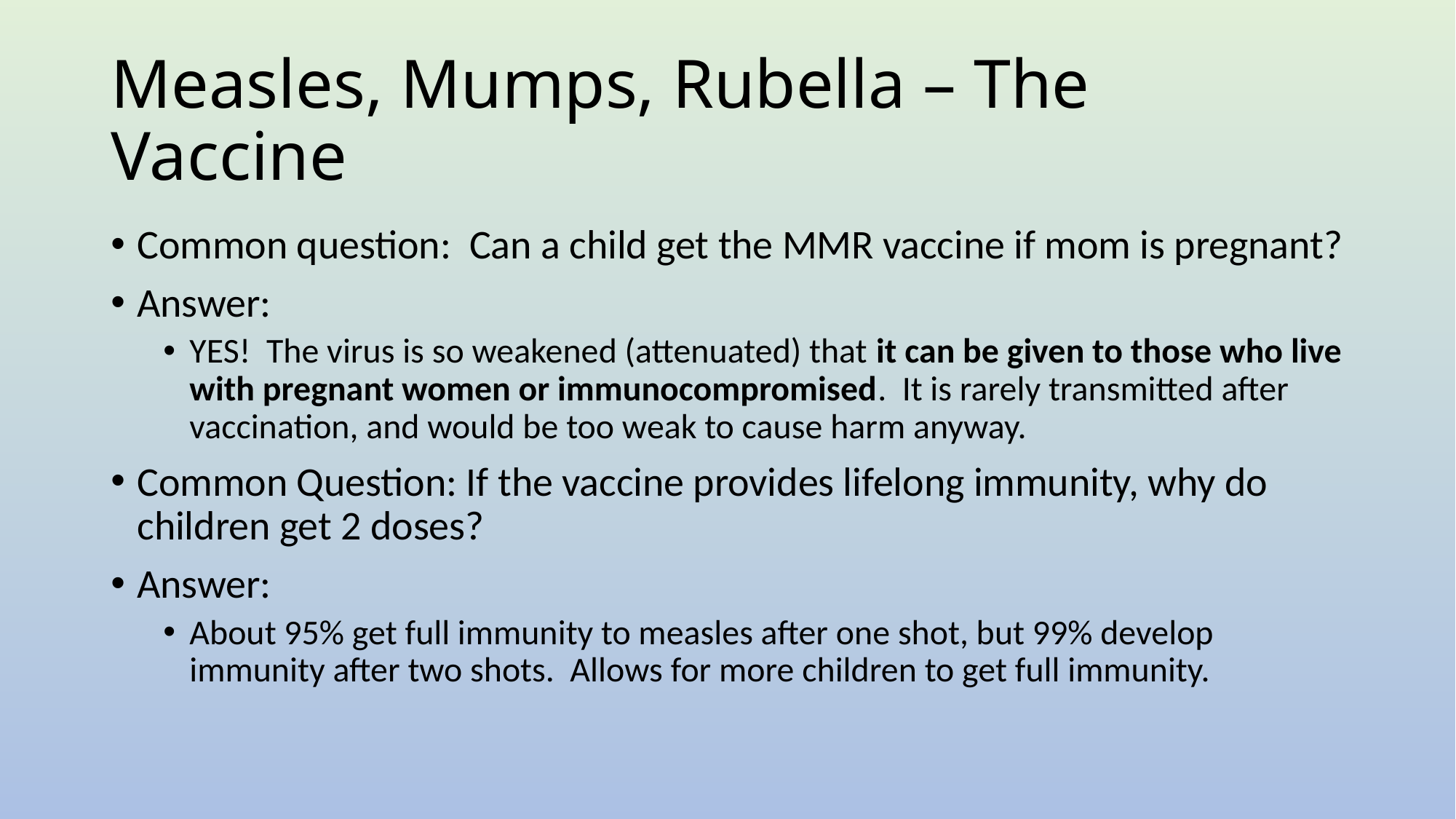

# Measles, Mumps, Rubella – The Vaccine
Common question: Can a child get the MMR vaccine if mom is pregnant?
Answer:
YES! The virus is so weakened (attenuated) that it can be given to those who live with pregnant women or immunocompromised. It is rarely transmitted after vaccination, and would be too weak to cause harm anyway.
Common Question: If the vaccine provides lifelong immunity, why do children get 2 doses?
Answer:
About 95% get full immunity to measles after one shot, but 99% develop immunity after two shots. Allows for more children to get full immunity.

## Slide 12
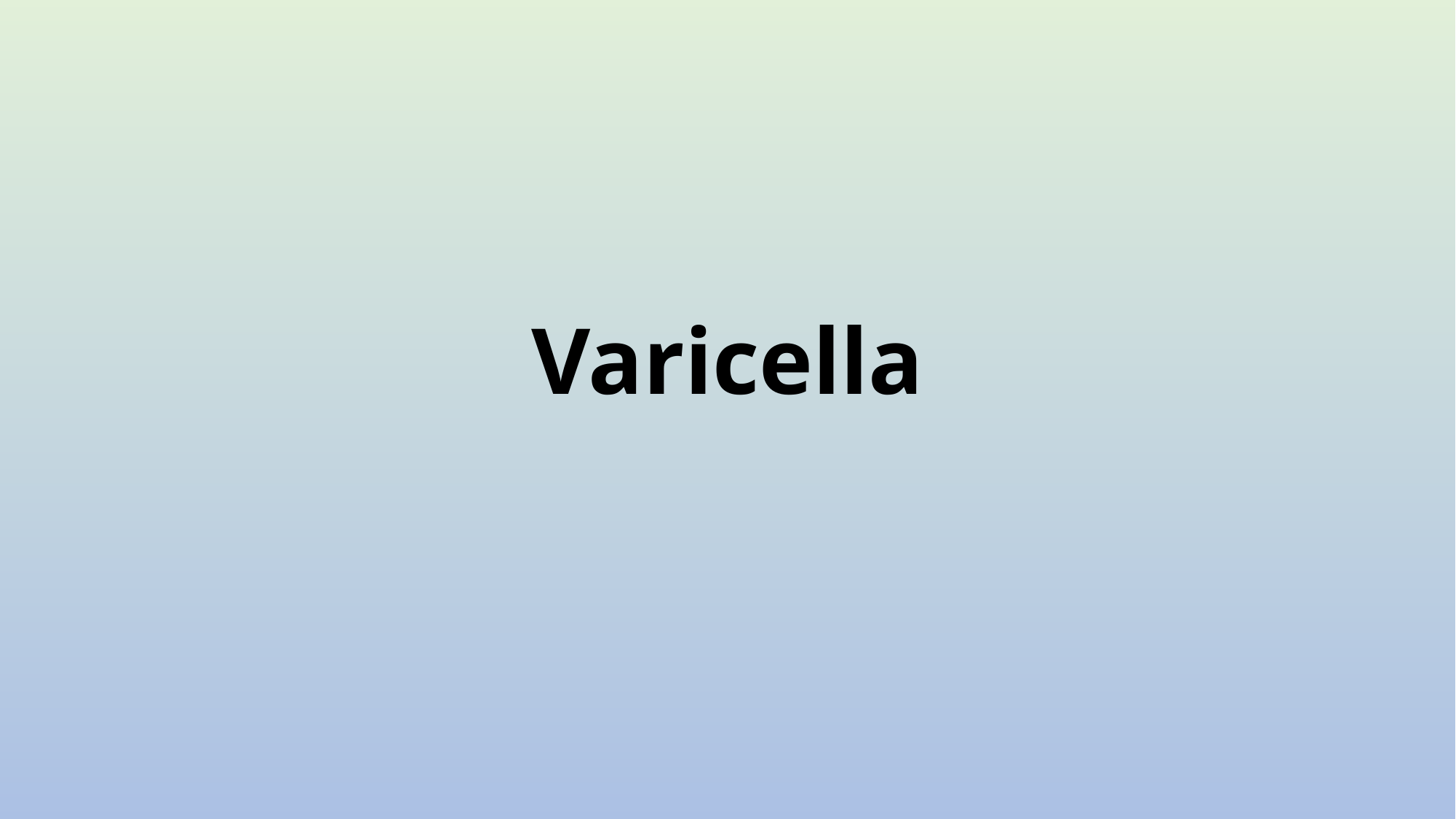

# Varicella

## Slide 13
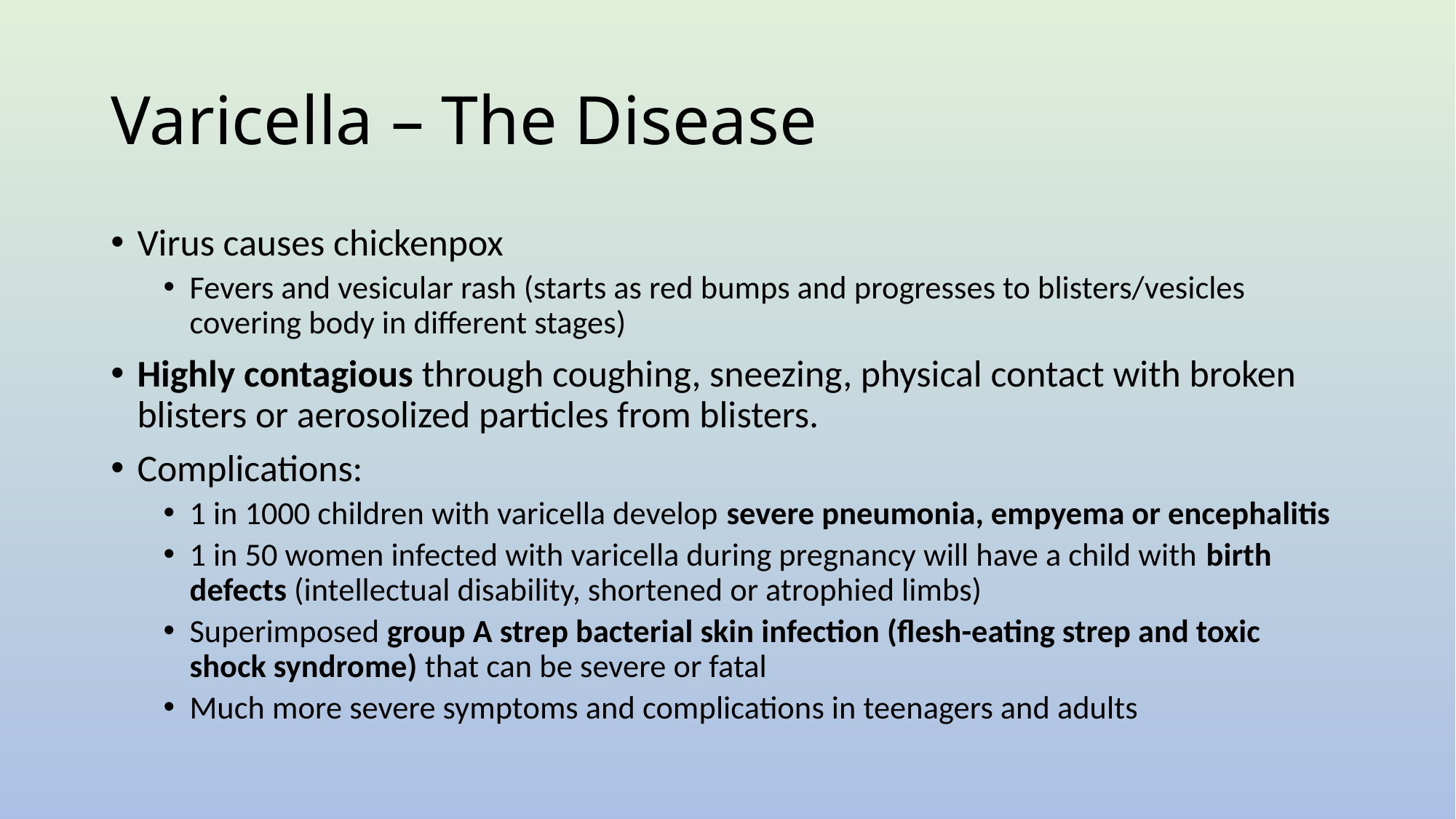

# Varicella – The Disease
Virus causes chickenpox
Fevers and vesicular rash (starts as red bumps and progresses to blisters/vesicles covering body in different stages)
Highly contagious through coughing, sneezing, physical contact with broken blisters or aerosolized particles from blisters.
Complications:
1 in 1000 children with varicella develop severe pneumonia, empyema or encephalitis
1 in 50 women infected with varicella during pregnancy will have a child with birth defects (intellectual disability, shortened or atrophied limbs)
Superimposed group A strep bacterial skin infection (flesh-eating strep and toxic shock syndrome) that can be severe or fatal
Much more severe symptoms and complications in teenagers and adults

## Slide 14
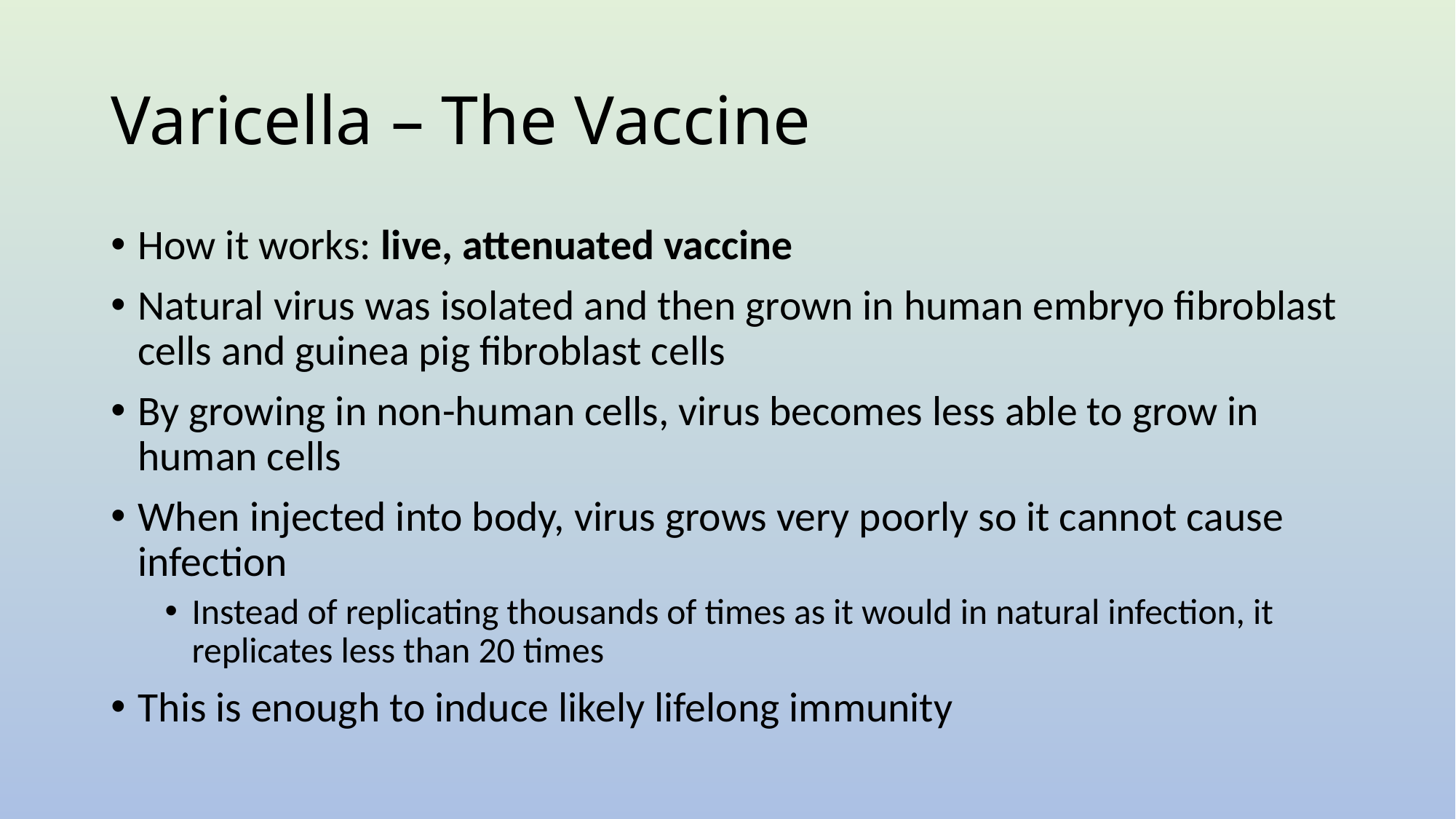

# Varicella – The Vaccine
How it works: live, attenuated vaccine
Natural virus was isolated and then grown in human embryo fibroblast cells and guinea pig fibroblast cells
By growing in non-human cells, virus becomes less able to grow in human cells
When injected into body, virus grows very poorly so it cannot cause infection
Instead of replicating thousands of times as it would in natural infection, it replicates less than 20 times
This is enough to induce likely lifelong immunity

## Slide 15
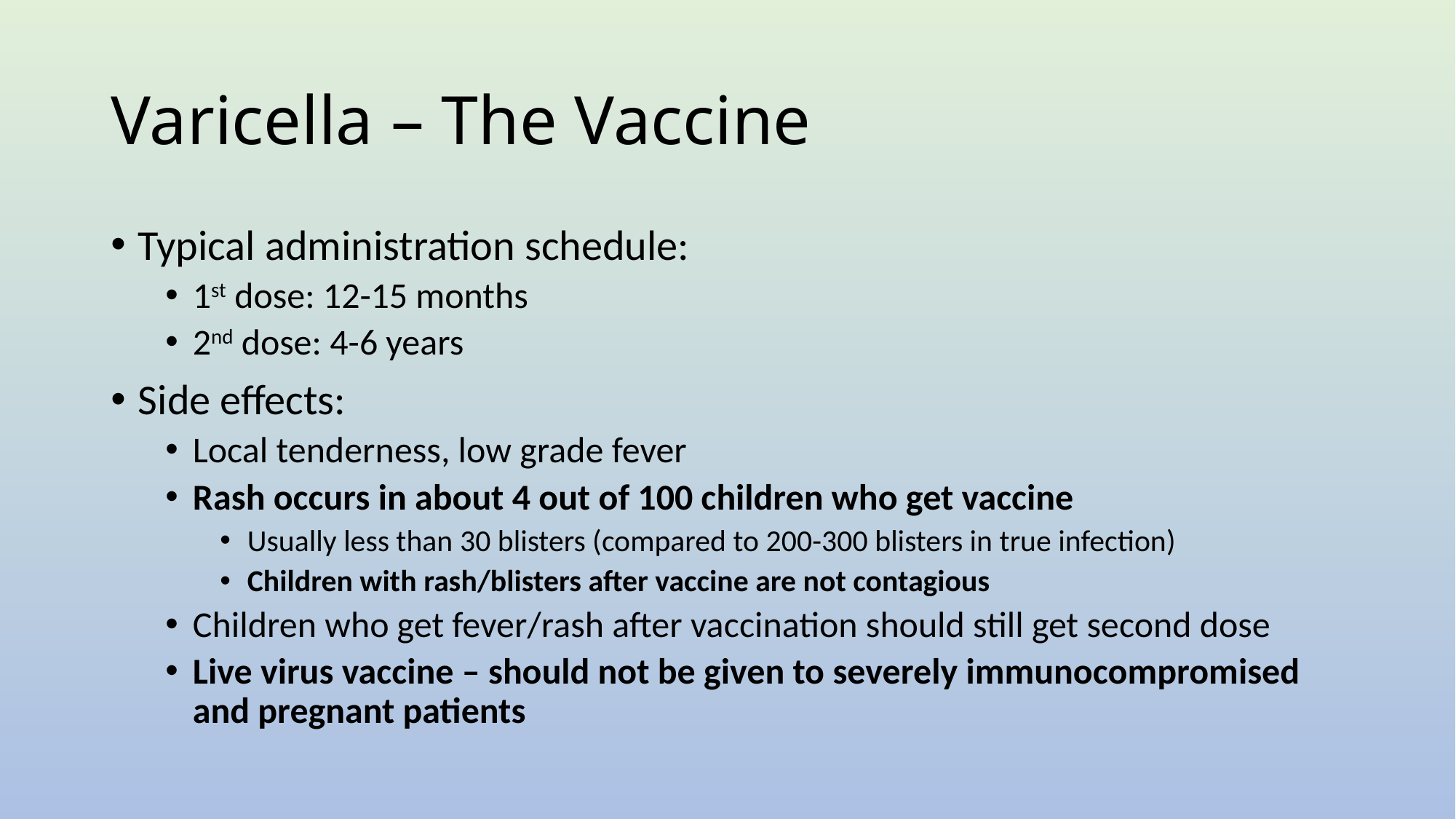

# Varicella – The Vaccine
Typical administration schedule:
1st dose: 12-15 months
2nd dose: 4-6 years
Side effects:
Local tenderness, low grade fever
Rash occurs in about 4 out of 100 children who get vaccine
Usually less than 30 blisters (compared to 200-300 blisters in true infection)
Children with rash/blisters after vaccine are not contagious
Children who get fever/rash after vaccination should still get second dose
Live virus vaccine – should not be given to severely immunocompromised and pregnant patients

## Slide 16
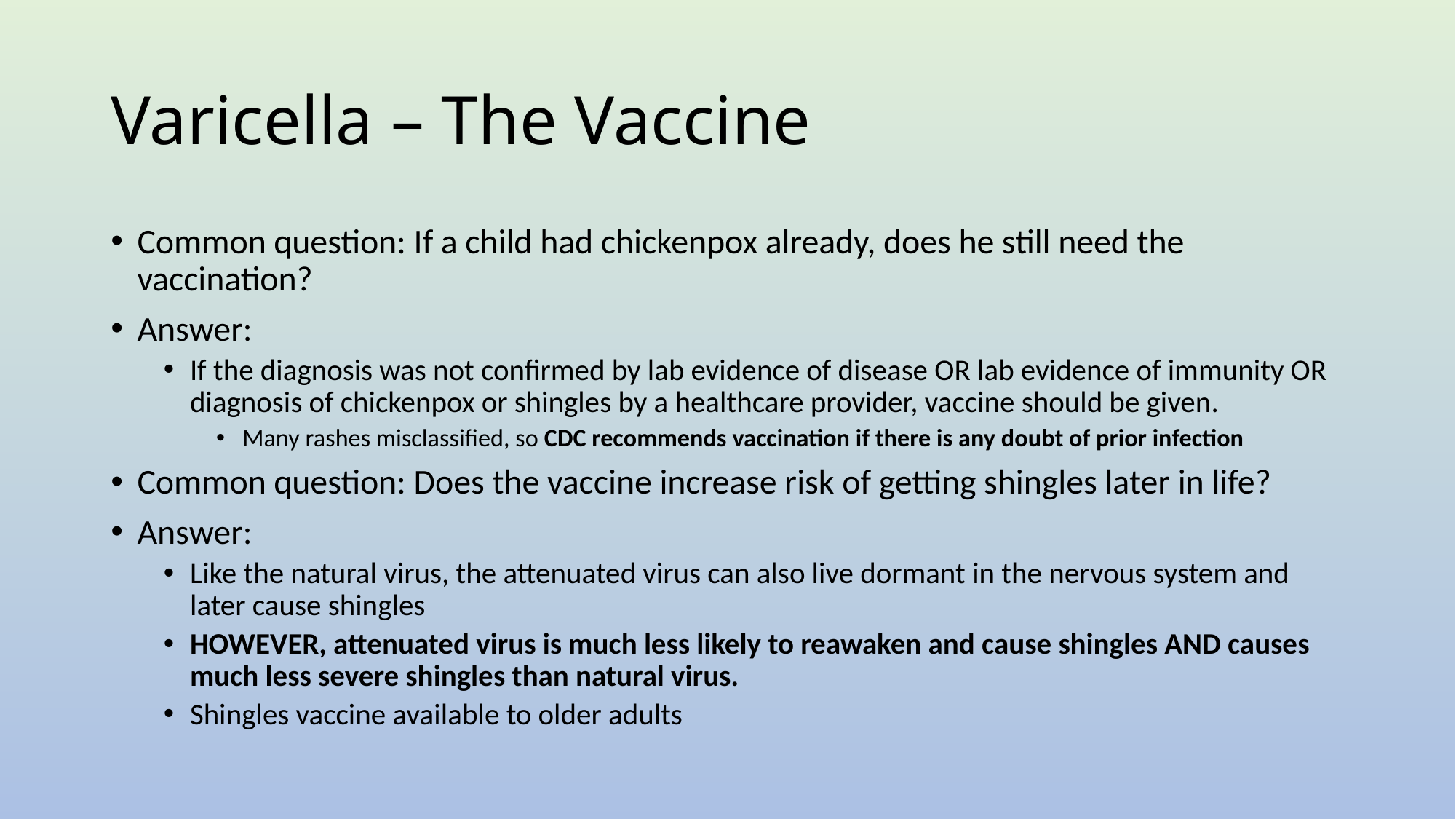

# Varicella – The Vaccine
Common question: If a child had chickenpox already, does he still need the vaccination?
Answer:
If the diagnosis was not confirmed by lab evidence of disease OR lab evidence of immunity OR diagnosis of chickenpox or shingles by a healthcare provider, vaccine should be given.
Many rashes misclassified, so CDC recommends vaccination if there is any doubt of prior infection
Common question: Does the vaccine increase risk of getting shingles later in life?
Answer:
Like the natural virus, the attenuated virus can also live dormant in the nervous system and later cause shingles
HOWEVER, attenuated virus is much less likely to reawaken and cause shingles AND causes much less severe shingles than natural virus.
Shingles vaccine available to older adults

## Slide 17
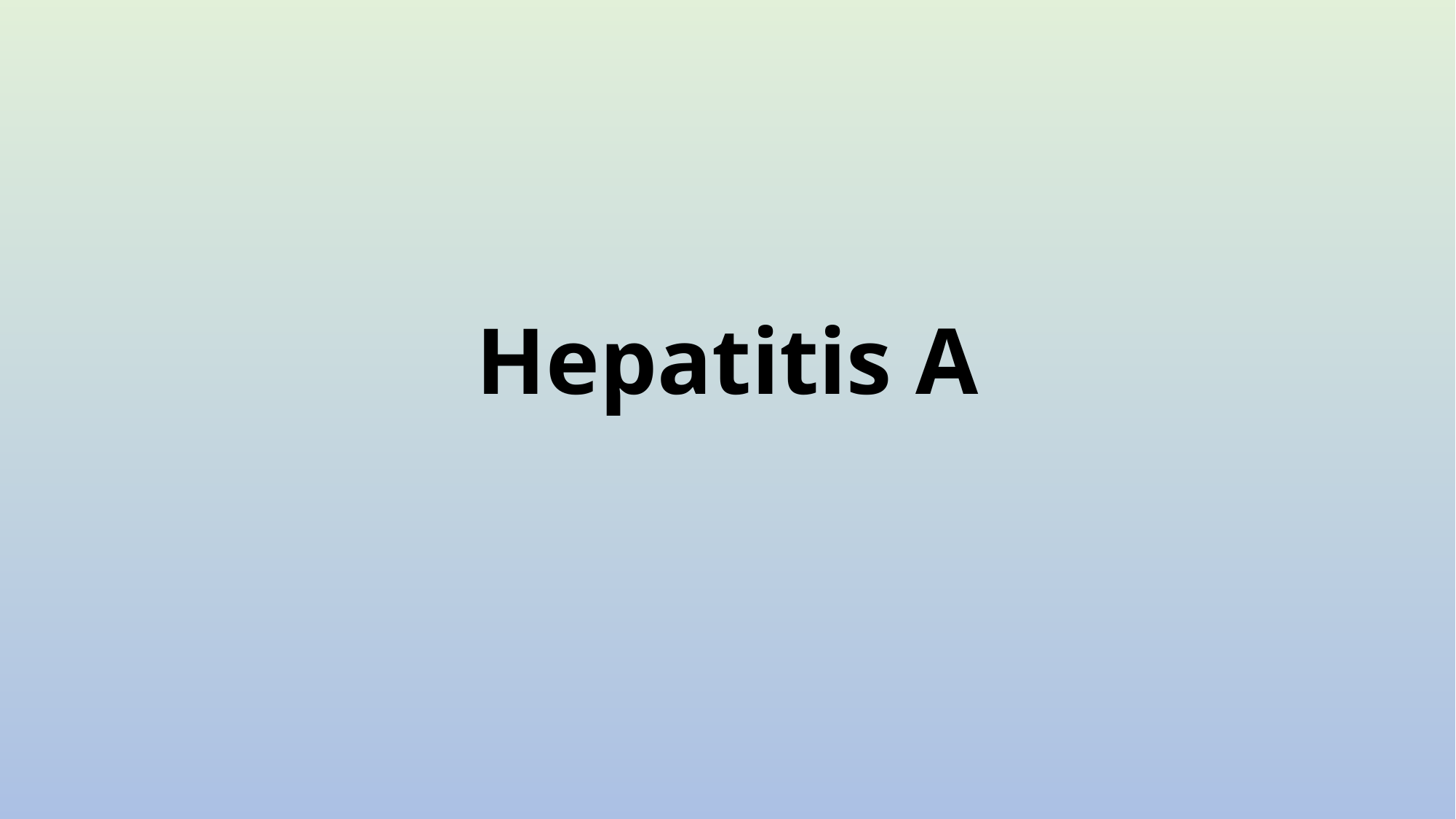

# Hepatitis A

## Slide 18
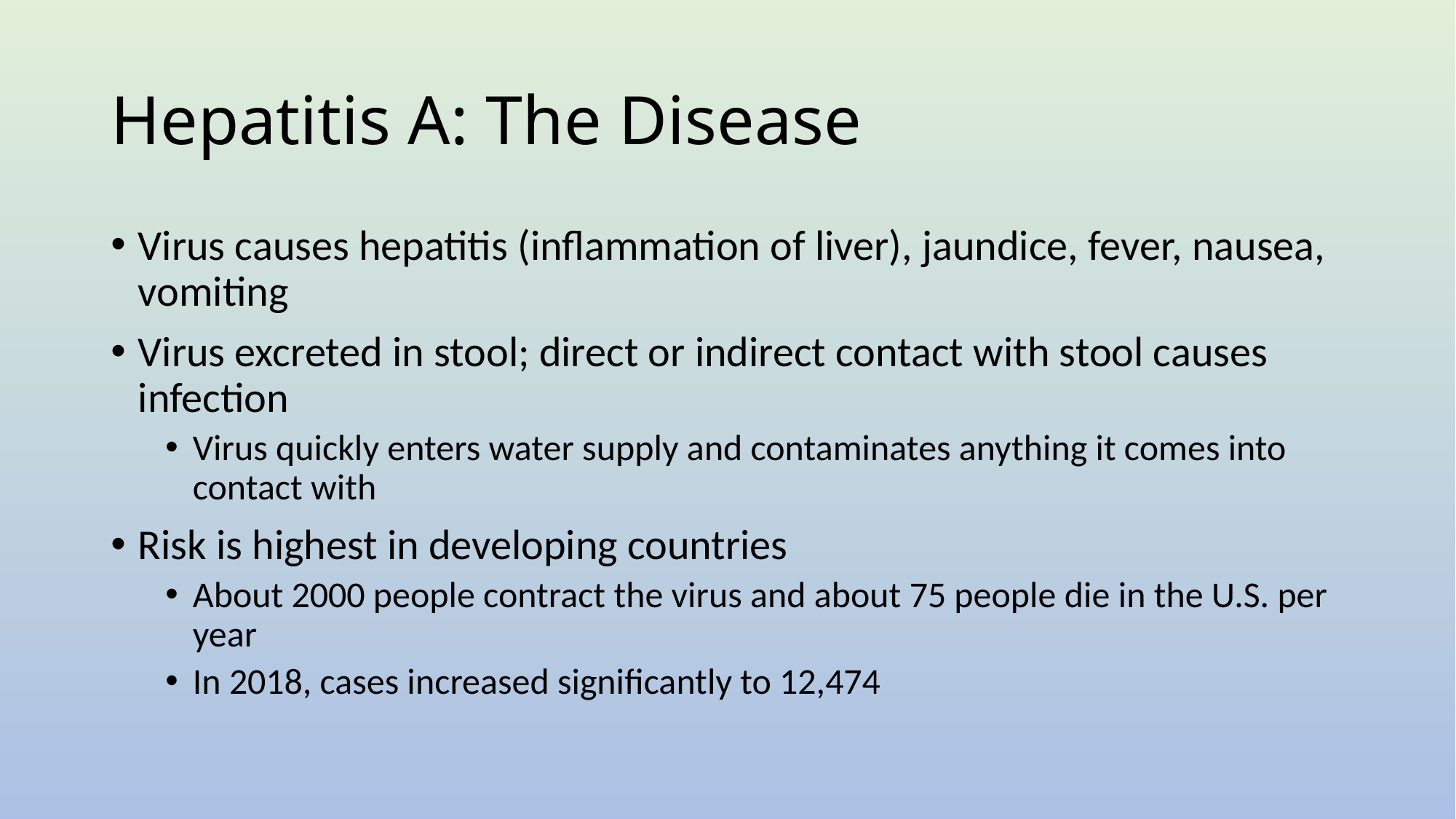

# Hepatitis A: The Disease
Virus causes hepatitis (inflammation of liver), jaundice, fever, nausea, vomiting
Virus excreted in stool; direct or indirect contact with stool causes infection
Virus quickly enters water supply and contaminates anything it comes into contact with
Risk is highest in developing countries
About 2000 people contract the virus and about 75 people die in the U.S. per year
In 2018, cases increased significantly to 12,474

## Slide 19
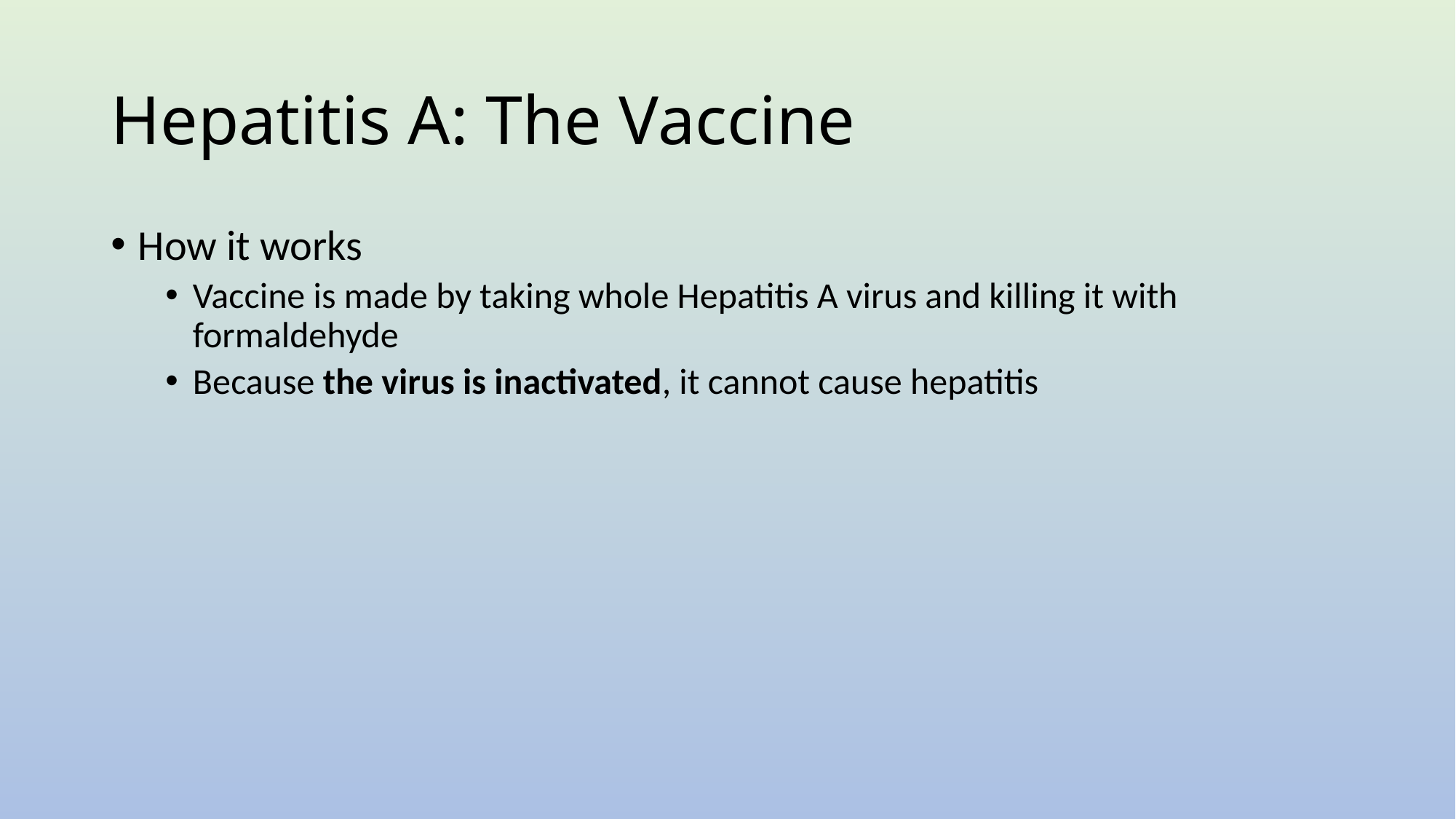

# Hepatitis A: The Vaccine
How it works
Vaccine is made by taking whole Hepatitis A virus and killing it with formaldehyde
Because the virus is inactivated, it cannot cause hepatitis

## Slide 20
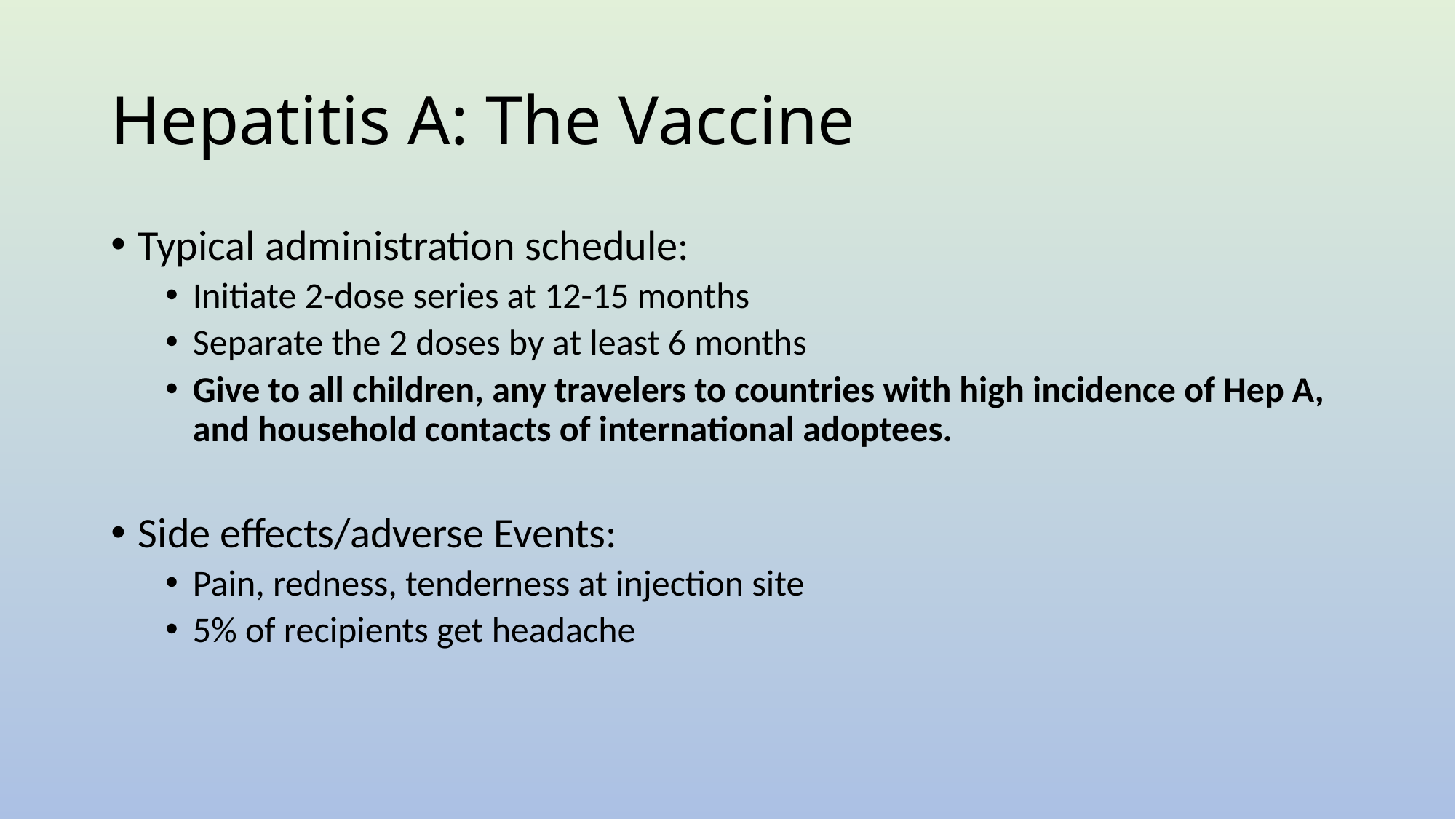

# Hepatitis A: The Vaccine
Typical administration schedule:
Initiate 2-dose series at 12-15 months
Separate the 2 doses by at least 6 months
Give to all children, any travelers to countries with high incidence of Hep A, and household contacts of international adoptees.
Side effects/adverse Events:
Pain, redness, tenderness at injection site
5% of recipients get headache

## Slide 21
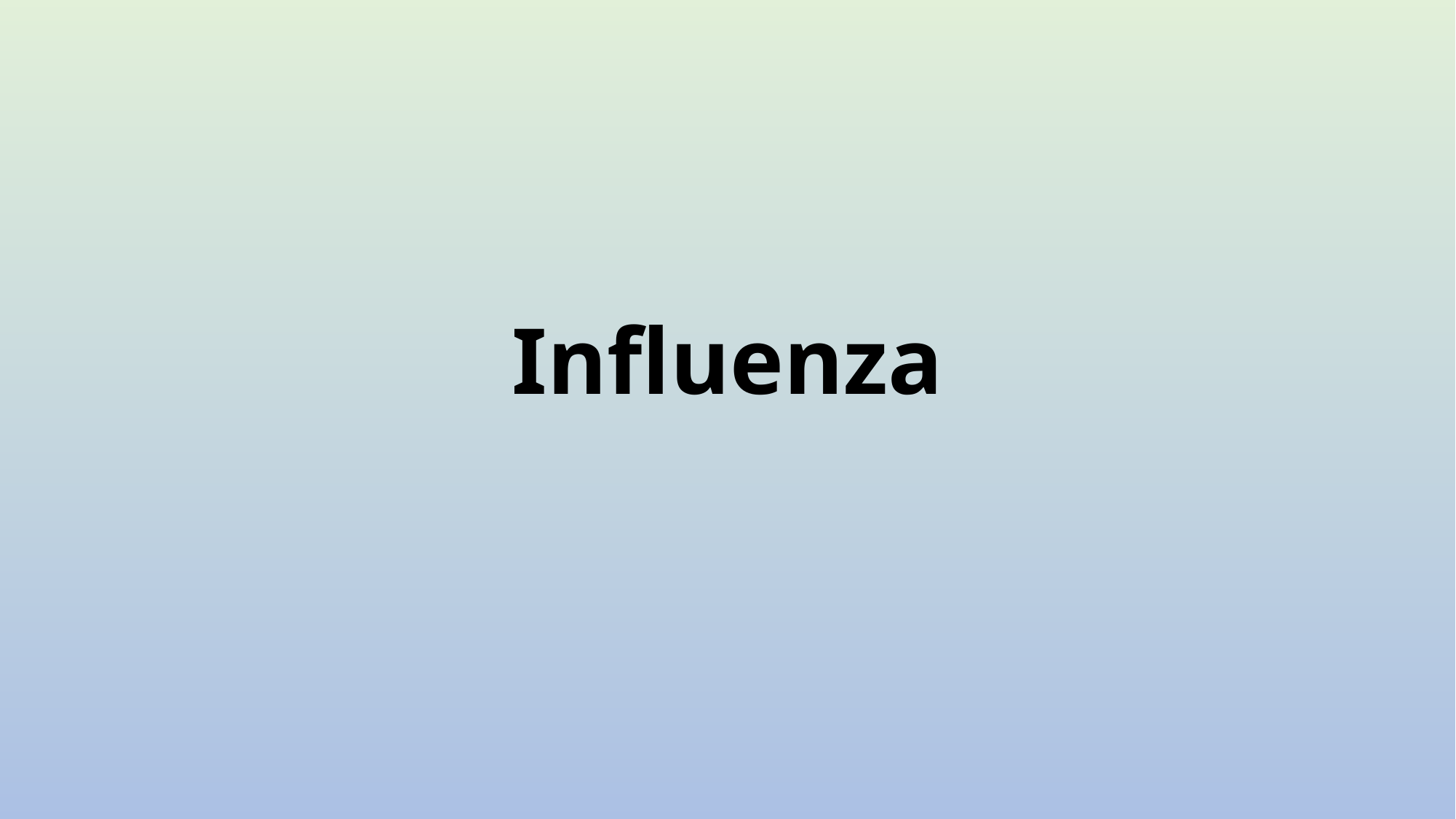

# Influenza

## Slide 22
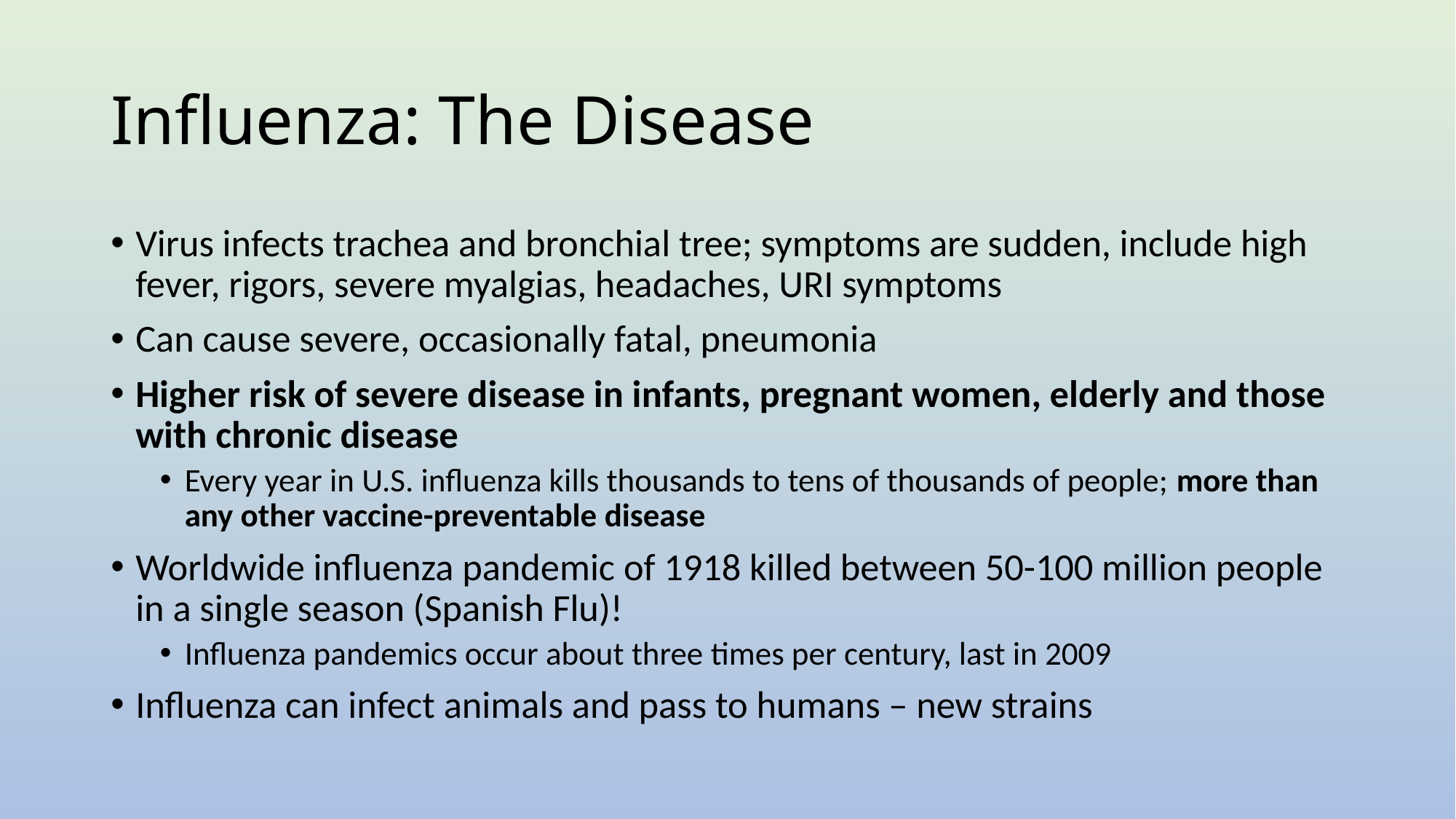

# Influenza: The Disease
Virus infects trachea and bronchial tree; symptoms are sudden, include high fever, rigors, severe myalgias, headaches, URI symptoms
Can cause severe, occasionally fatal, pneumonia
Higher risk of severe disease in infants, pregnant women, elderly and those with chronic disease
Every year in U.S. influenza kills thousands to tens of thousands of people; more than any other vaccine-preventable disease
Worldwide influenza pandemic of 1918 killed between 50-100 million people in a single season (Spanish Flu)!
Influenza pandemics occur about three times per century, last in 2009
Influenza can infect animals and pass to humans – new strains

## Slide 23
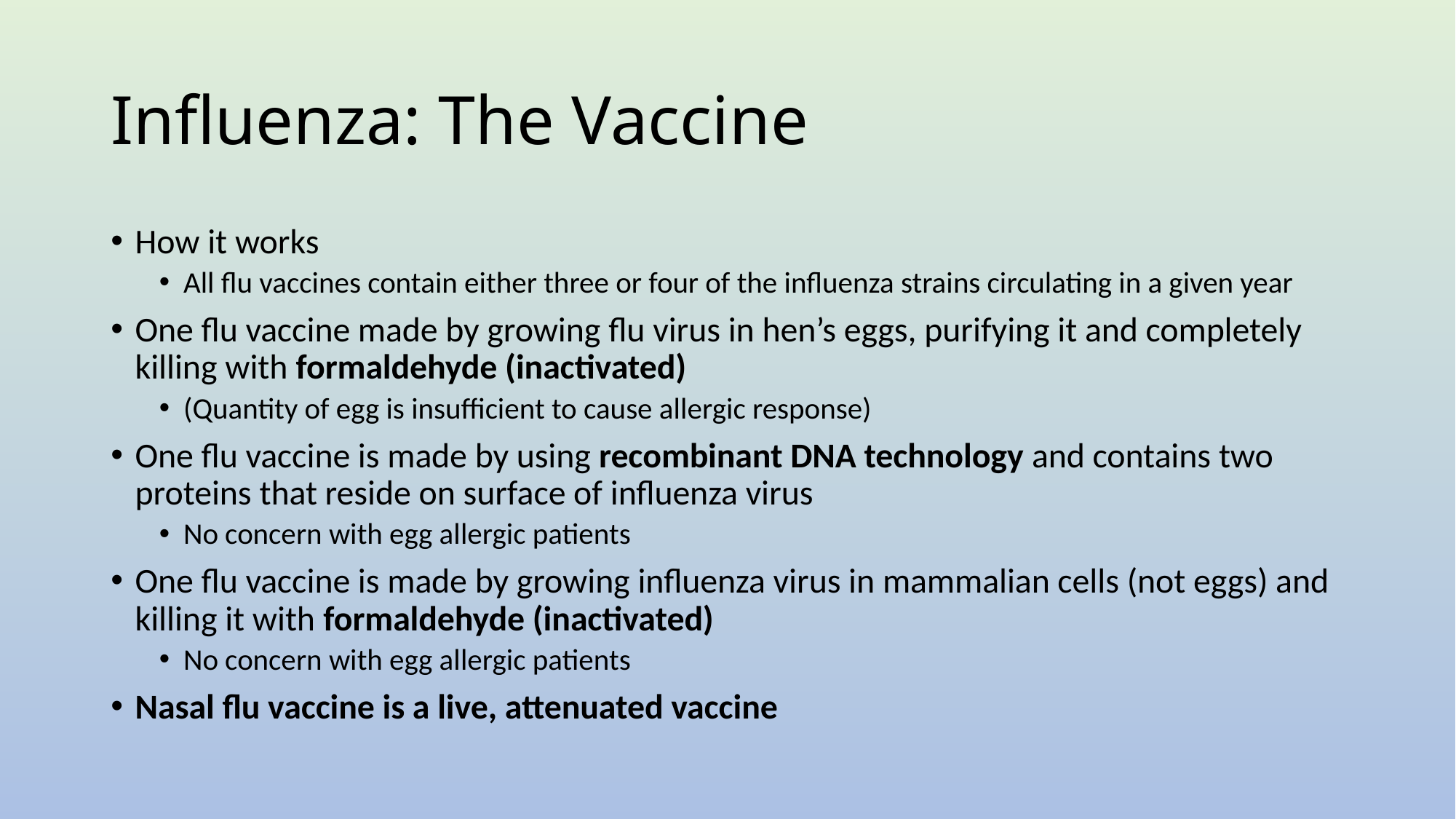

# Influenza: The Vaccine
How it works
All flu vaccines contain either three or four of the influenza strains circulating in a given year
One flu vaccine made by growing flu virus in hen’s eggs, purifying it and completely killing with formaldehyde (inactivated)
(Quantity of egg is insufficient to cause allergic response)
One flu vaccine is made by using recombinant DNA technology and contains two proteins that reside on surface of influenza virus
No concern with egg allergic patients
One flu vaccine is made by growing influenza virus in mammalian cells (not eggs) and killing it with formaldehyde (inactivated)
No concern with egg allergic patients
Nasal flu vaccine is a live, attenuated vaccine

## Slide 24
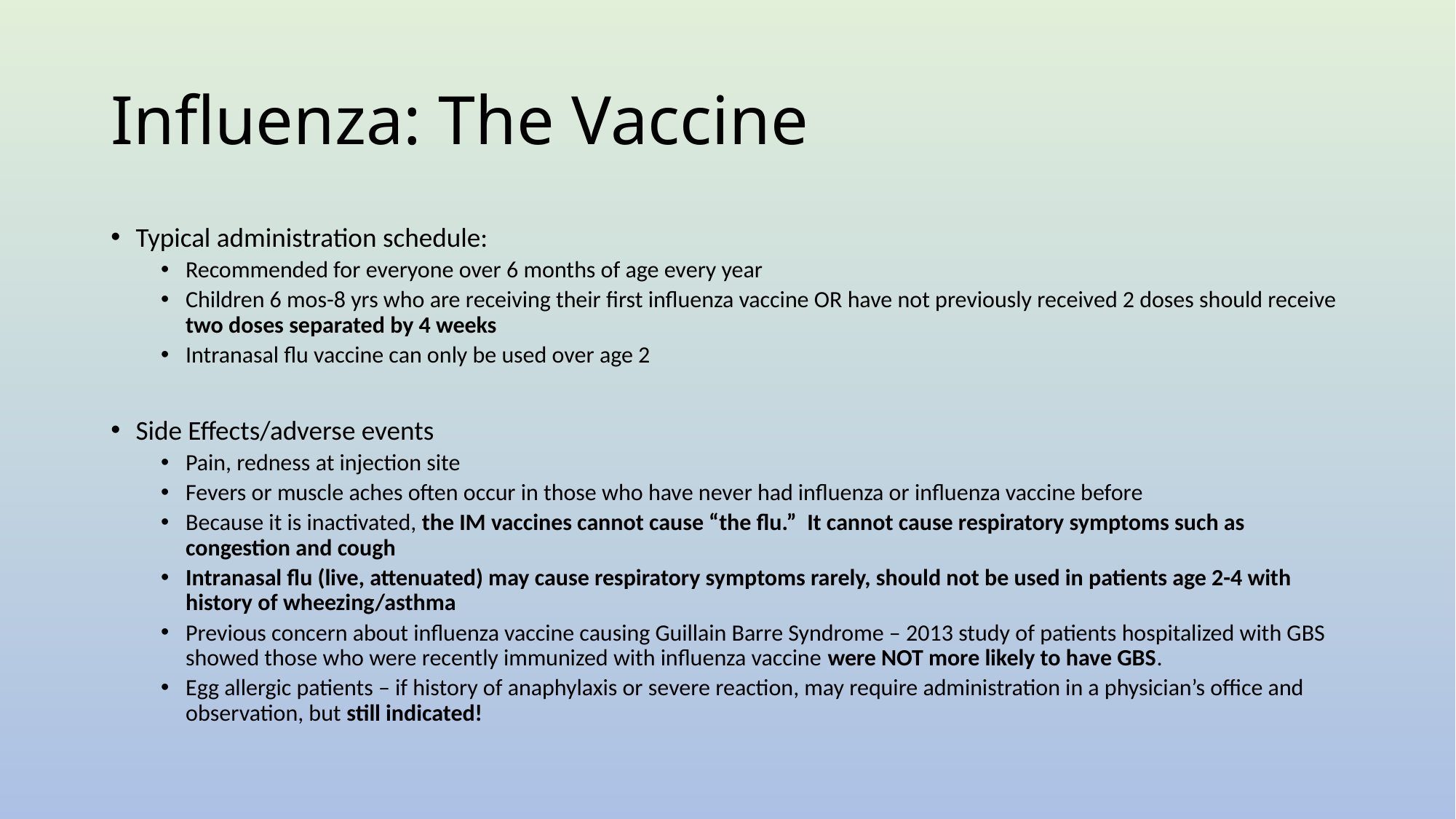

# Influenza: The Vaccine
Typical administration schedule:
Recommended for everyone over 6 months of age every year
Children 6 mos-8 yrs who are receiving their first influenza vaccine OR have not previously received 2 doses should receive two doses separated by 4 weeks
Intranasal flu vaccine can only be used over age 2
Side Effects/adverse events
Pain, redness at injection site
Fevers or muscle aches often occur in those who have never had influenza or influenza vaccine before
Because it is inactivated, the IM vaccines cannot cause “the flu.” It cannot cause respiratory symptoms such as congestion and cough
Intranasal flu (live, attenuated) may cause respiratory symptoms rarely, should not be used in patients age 2-4 with history of wheezing/asthma
Previous concern about influenza vaccine causing Guillain Barre Syndrome – 2013 study of patients hospitalized with GBS showed those who were recently immunized with influenza vaccine were NOT more likely to have GBS.
Egg allergic patients – if history of anaphylaxis or severe reaction, may require administration in a physician’s office and observation, but still indicated!

## Slide 25
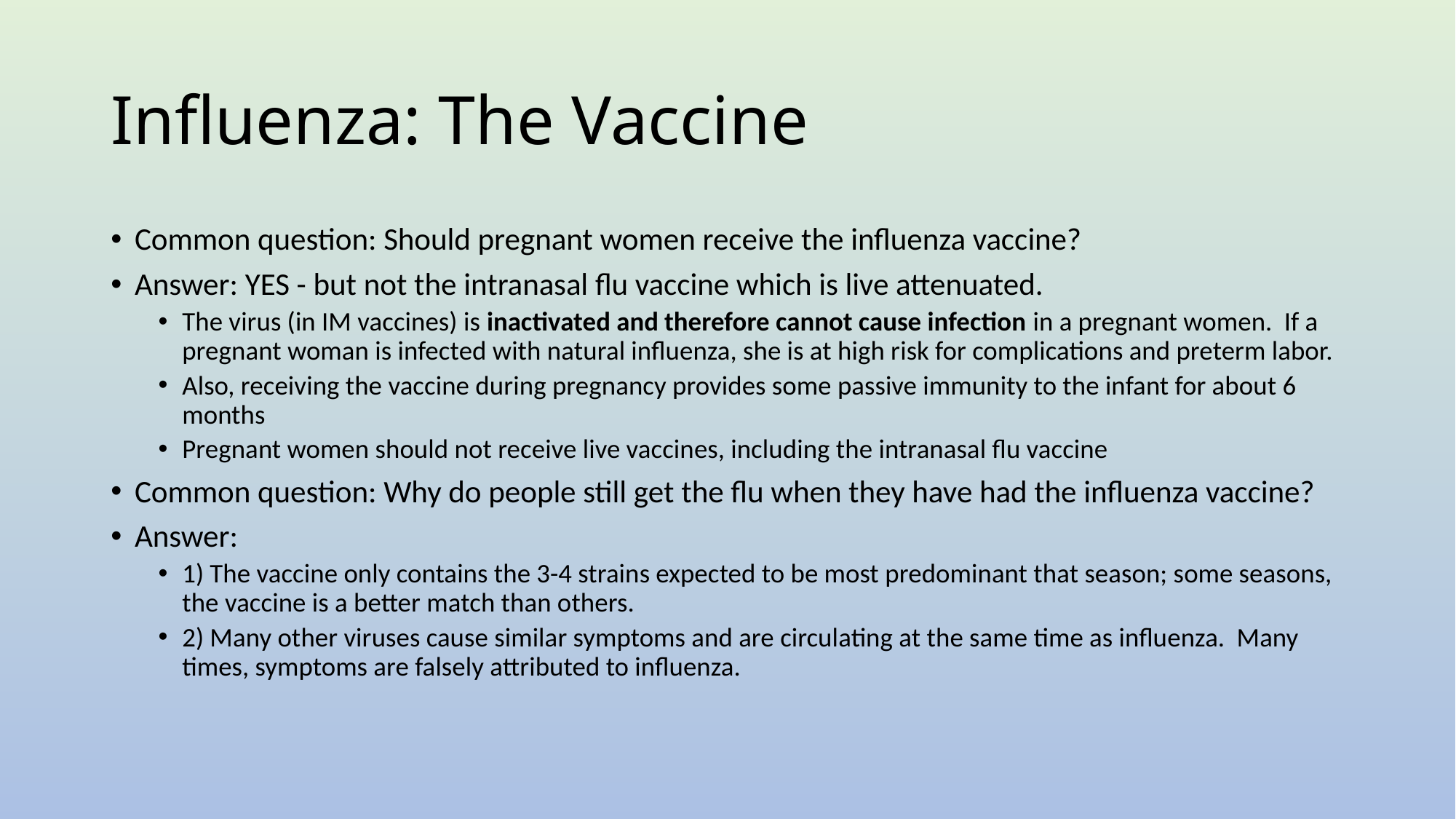

# Influenza: The Vaccine
Common question: Should pregnant women receive the influenza vaccine?
Answer: YES - but not the intranasal flu vaccine which is live attenuated.
The virus (in IM vaccines) is inactivated and therefore cannot cause infection in a pregnant women. If a pregnant woman is infected with natural influenza, she is at high risk for complications and preterm labor.
Also, receiving the vaccine during pregnancy provides some passive immunity to the infant for about 6 months
Pregnant women should not receive live vaccines, including the intranasal flu vaccine
Common question: Why do people still get the flu when they have had the influenza vaccine?
Answer:
1) The vaccine only contains the 3-4 strains expected to be most predominant that season; some seasons, the vaccine is a better match than others.
2) Many other viruses cause similar symptoms and are circulating at the same time as influenza. Many times, symptoms are falsely attributed to influenza.

## Slide 26
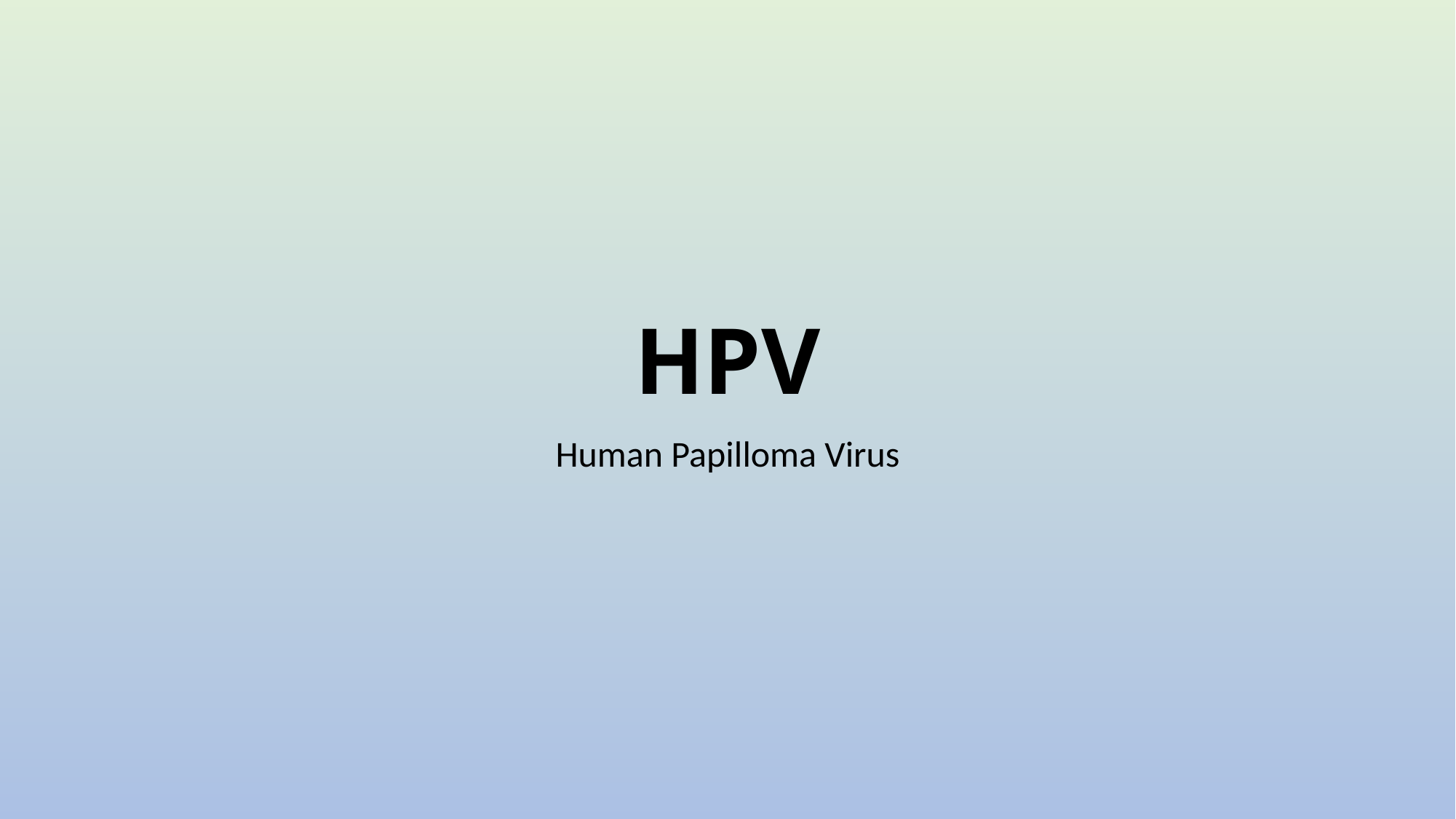

# HPV
Human Papilloma Virus

## Slide 27
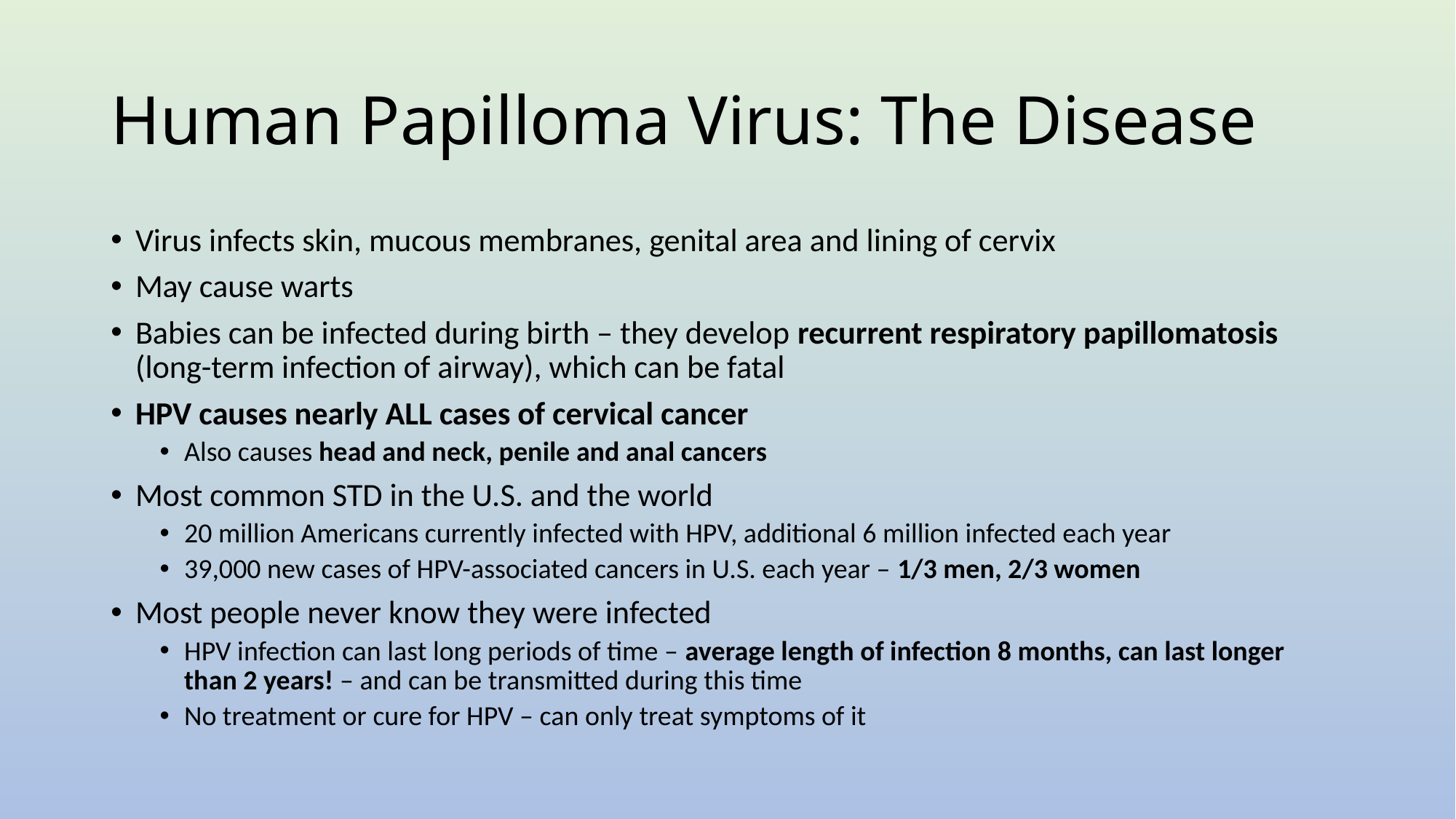

# Human Papilloma Virus: The Disease
Virus infects skin, mucous membranes, genital area and lining of cervix
May cause warts
Babies can be infected during birth – they develop recurrent respiratory papillomatosis (long-term infection of airway), which can be fatal
HPV causes nearly ALL cases of cervical cancer
Also causes head and neck, penile and anal cancers
Most common STD in the U.S. and the world
20 million Americans currently infected with HPV, additional 6 million infected each year
39,000 new cases of HPV-associated cancers in U.S. each year – 1/3 men, 2/3 women
Most people never know they were infected
HPV infection can last long periods of time – average length of infection 8 months, can last longer than 2 years! – and can be transmitted during this time
No treatment or cure for HPV – can only treat symptoms of it

## Slide 28
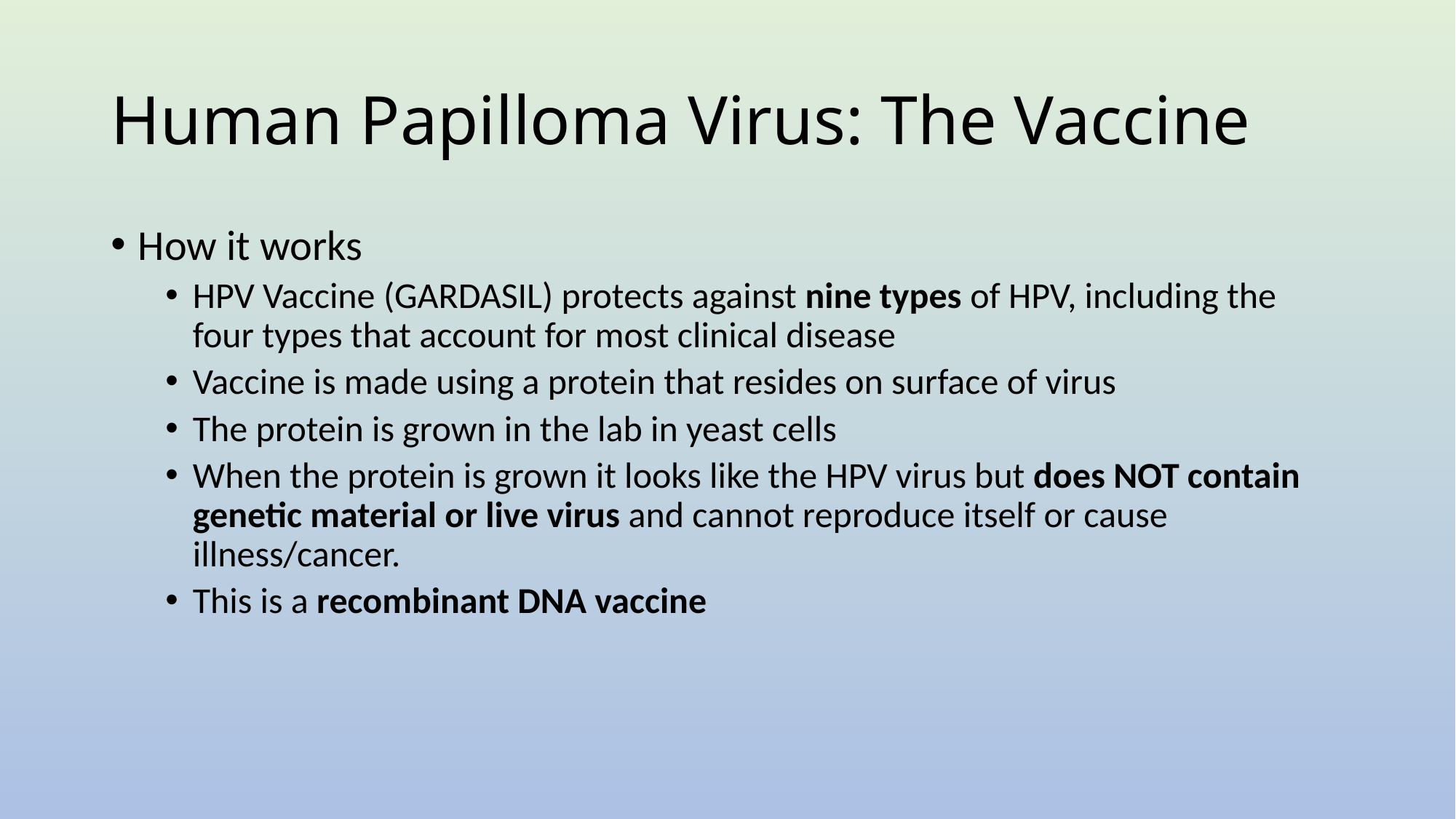

# Human Papilloma Virus: The Vaccine
How it works
HPV Vaccine (GARDASIL) protects against nine types of HPV, including the four types that account for most clinical disease
Vaccine is made using a protein that resides on surface of virus
The protein is grown in the lab in yeast cells
When the protein is grown it looks like the HPV virus but does NOT contain genetic material or live virus and cannot reproduce itself or cause illness/cancer.
This is a recombinant DNA vaccine

## Slide 29
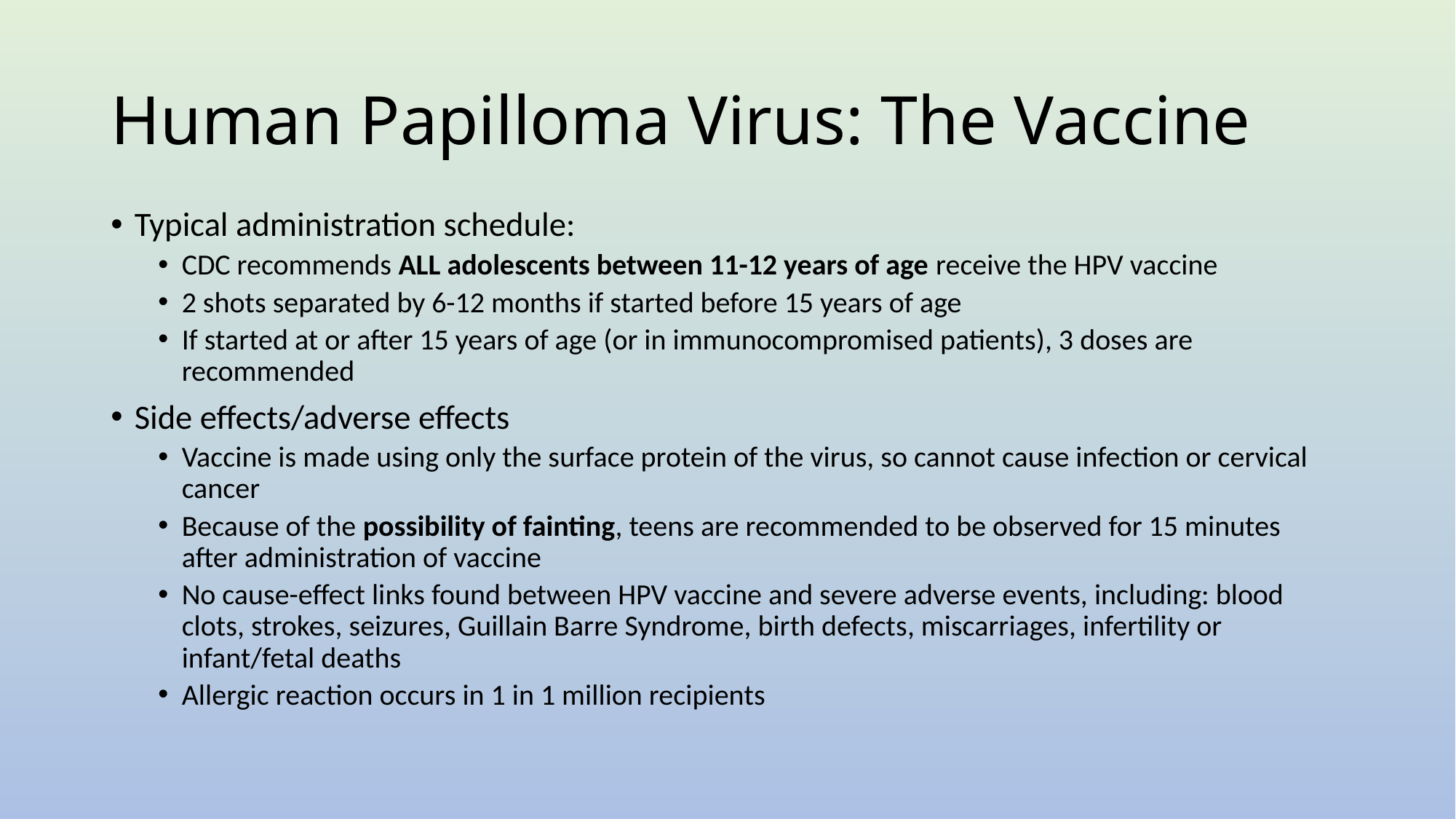

# Human Papilloma Virus: The Vaccine
Typical administration schedule:
CDC recommends ALL adolescents between 11-12 years of age receive the HPV vaccine
2 shots separated by 6-12 months if started before 15 years of age
If started at or after 15 years of age (or in immunocompromised patients), 3 doses are recommended
Side effects/adverse effects
Vaccine is made using only the surface protein of the virus, so cannot cause infection or cervical cancer
Because of the possibility of fainting, teens are recommended to be observed for 15 minutes after administration of vaccine
No cause-effect links found between HPV vaccine and severe adverse events, including: blood clots, strokes, seizures, Guillain Barre Syndrome, birth defects, miscarriages, infertility or infant/fetal deaths
Allergic reaction occurs in 1 in 1 million recipients

## Slide 30
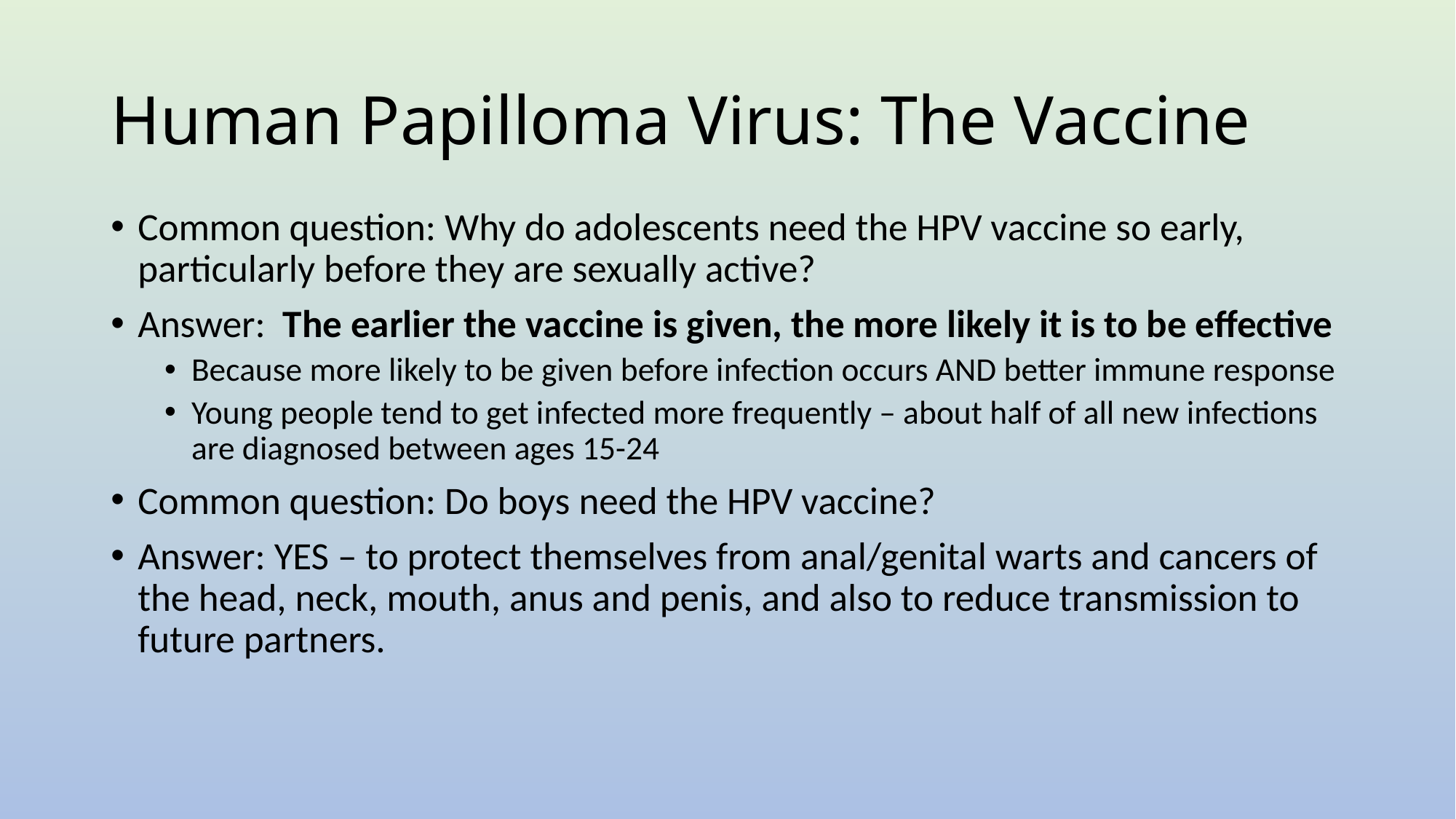

# Human Papilloma Virus: The Vaccine
Common question: Why do adolescents need the HPV vaccine so early, particularly before they are sexually active?
Answer: The earlier the vaccine is given, the more likely it is to be effective
Because more likely to be given before infection occurs AND better immune response
Young people tend to get infected more frequently – about half of all new infections are diagnosed between ages 15-24
Common question: Do boys need the HPV vaccine?
Answer: YES – to protect themselves from anal/genital warts and cancers of the head, neck, mouth, anus and penis, and also to reduce transmission to future partners.

## Slide 31
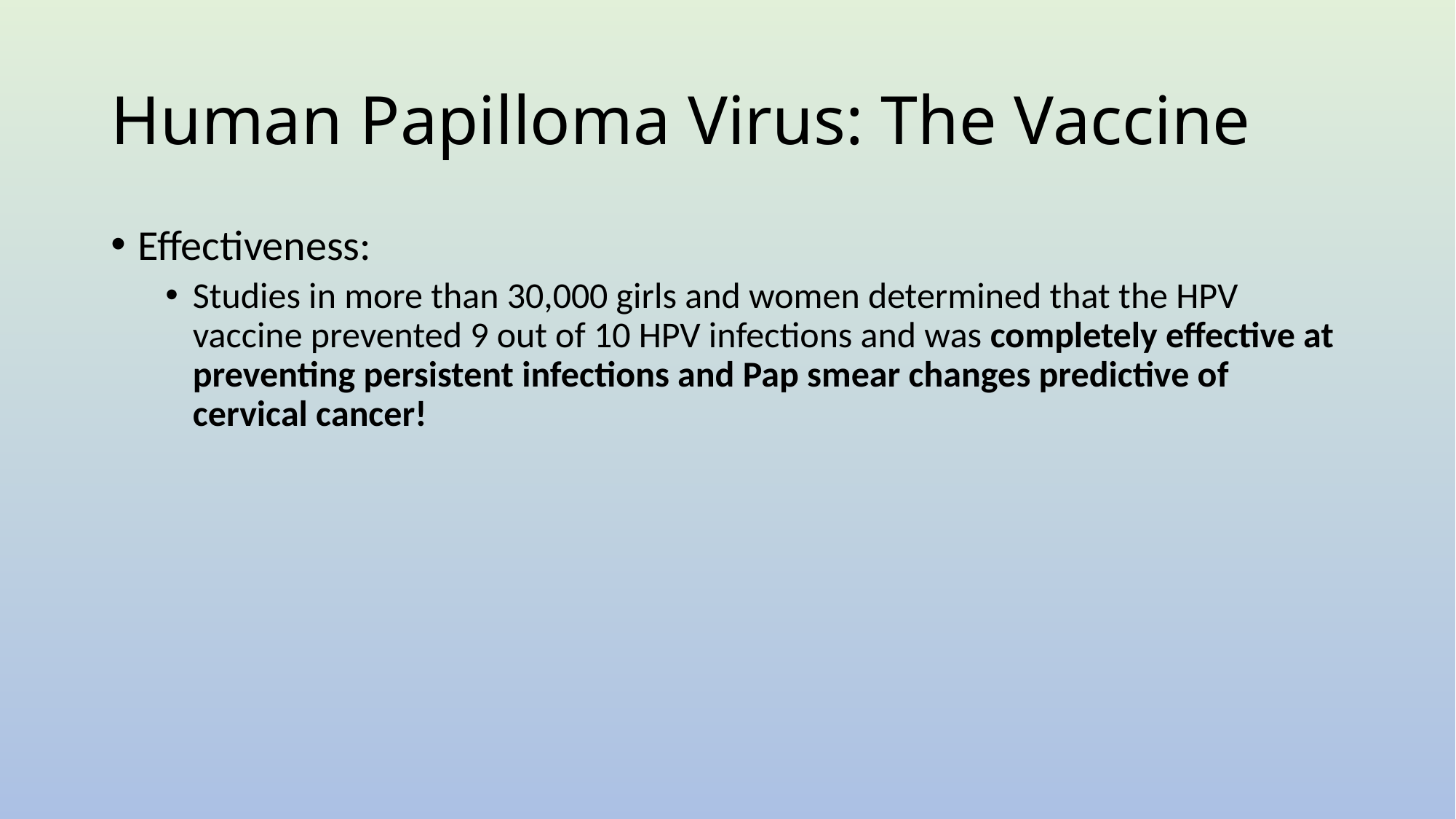

# Human Papilloma Virus: The Vaccine
Effectiveness:
Studies in more than 30,000 girls and women determined that the HPV vaccine prevented 9 out of 10 HPV infections and was completely effective at preventing persistent infections and Pap smear changes predictive of cervical cancer!

## Slide 32
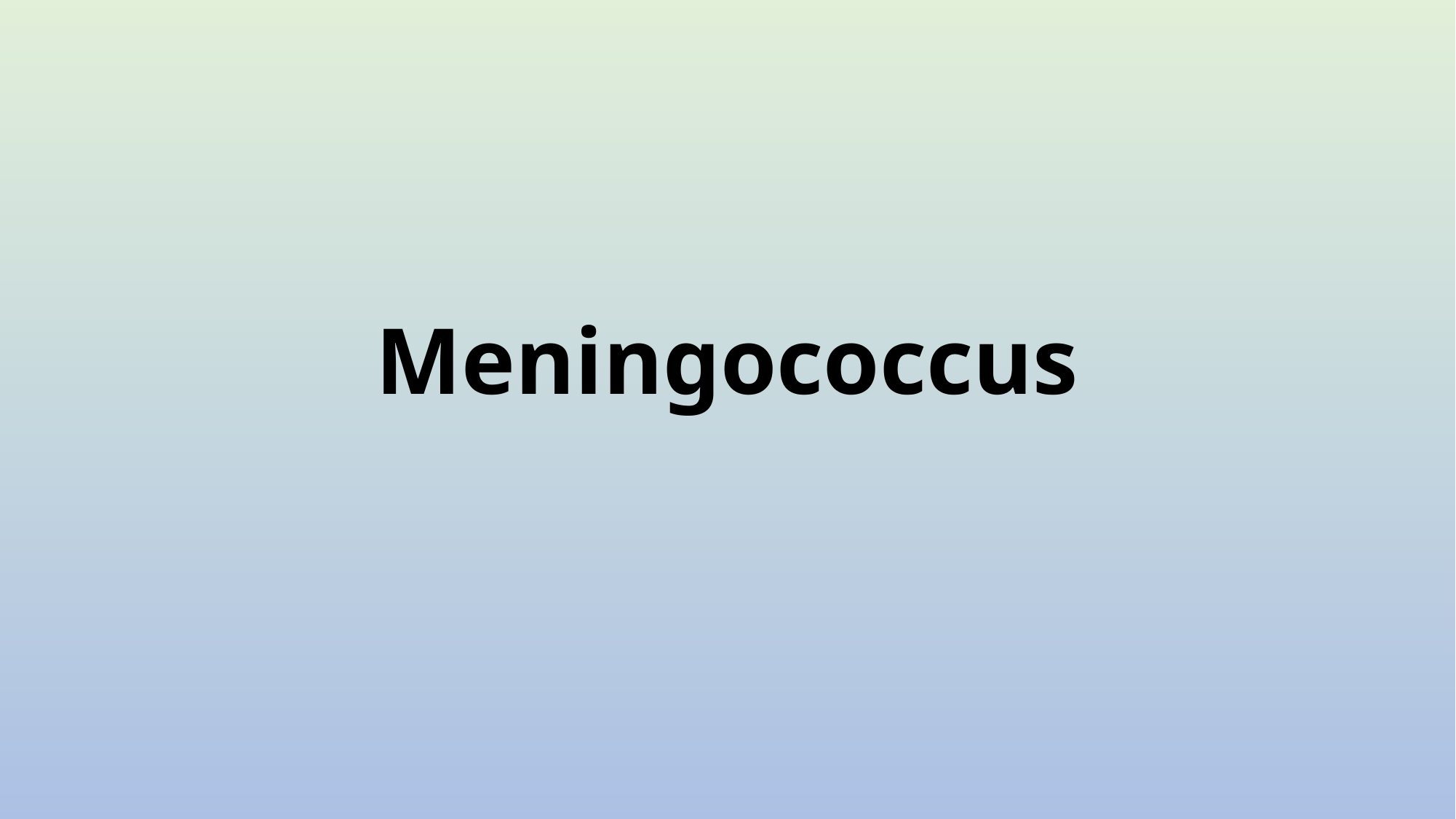

# Meningococcus

## Slide 33
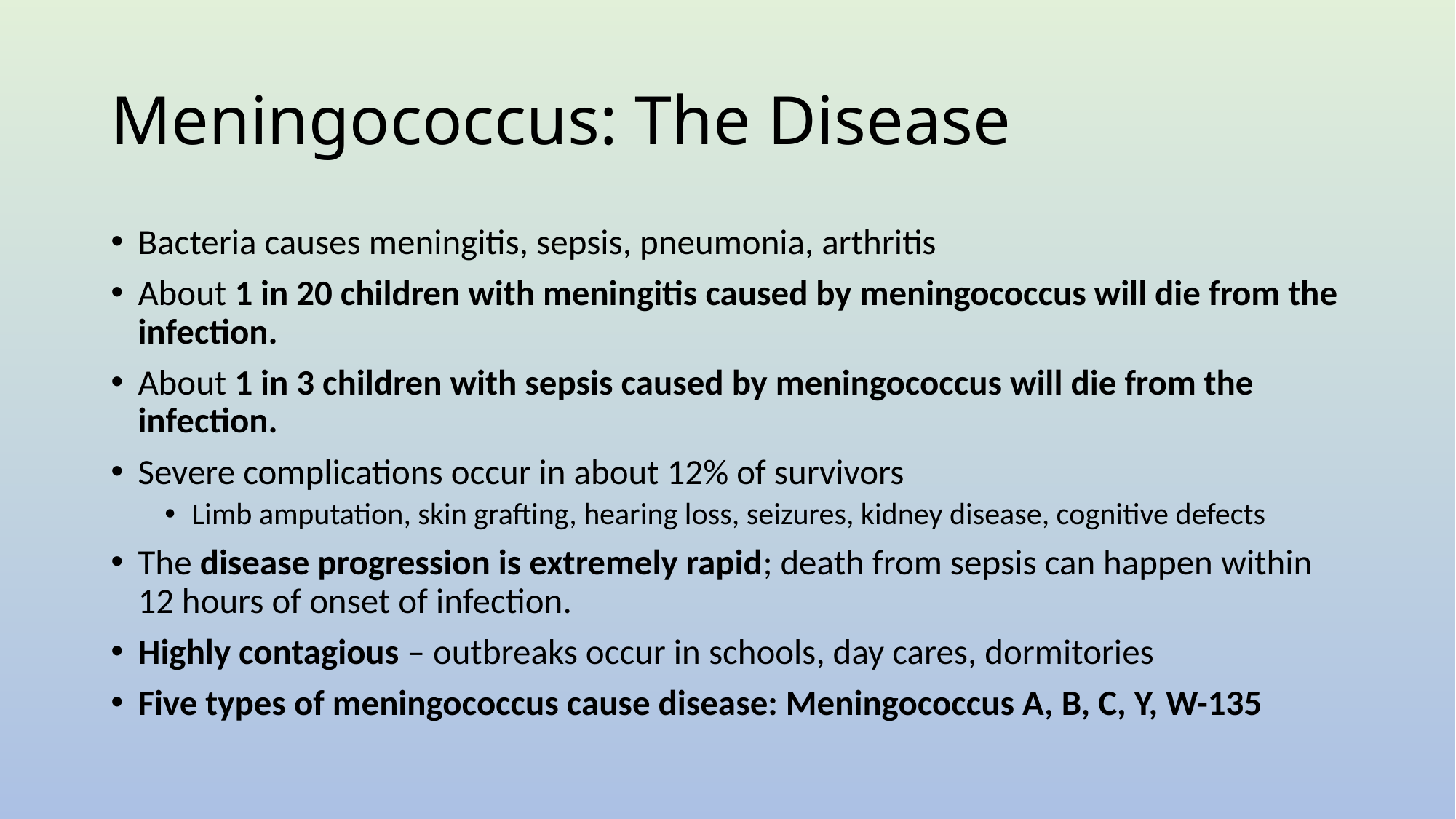

# Meningococcus: The Disease
Bacteria causes meningitis, sepsis, pneumonia, arthritis
About 1 in 20 children with meningitis caused by meningococcus will die from the infection.
About 1 in 3 children with sepsis caused by meningococcus will die from the infection.
Severe complications occur in about 12% of survivors
Limb amputation, skin grafting, hearing loss, seizures, kidney disease, cognitive defects
The disease progression is extremely rapid; death from sepsis can happen within 12 hours of onset of infection.
Highly contagious – outbreaks occur in schools, day cares, dormitories
Five types of meningococcus cause disease: Meningococcus A, B, C, Y, W-135

## Slide 34
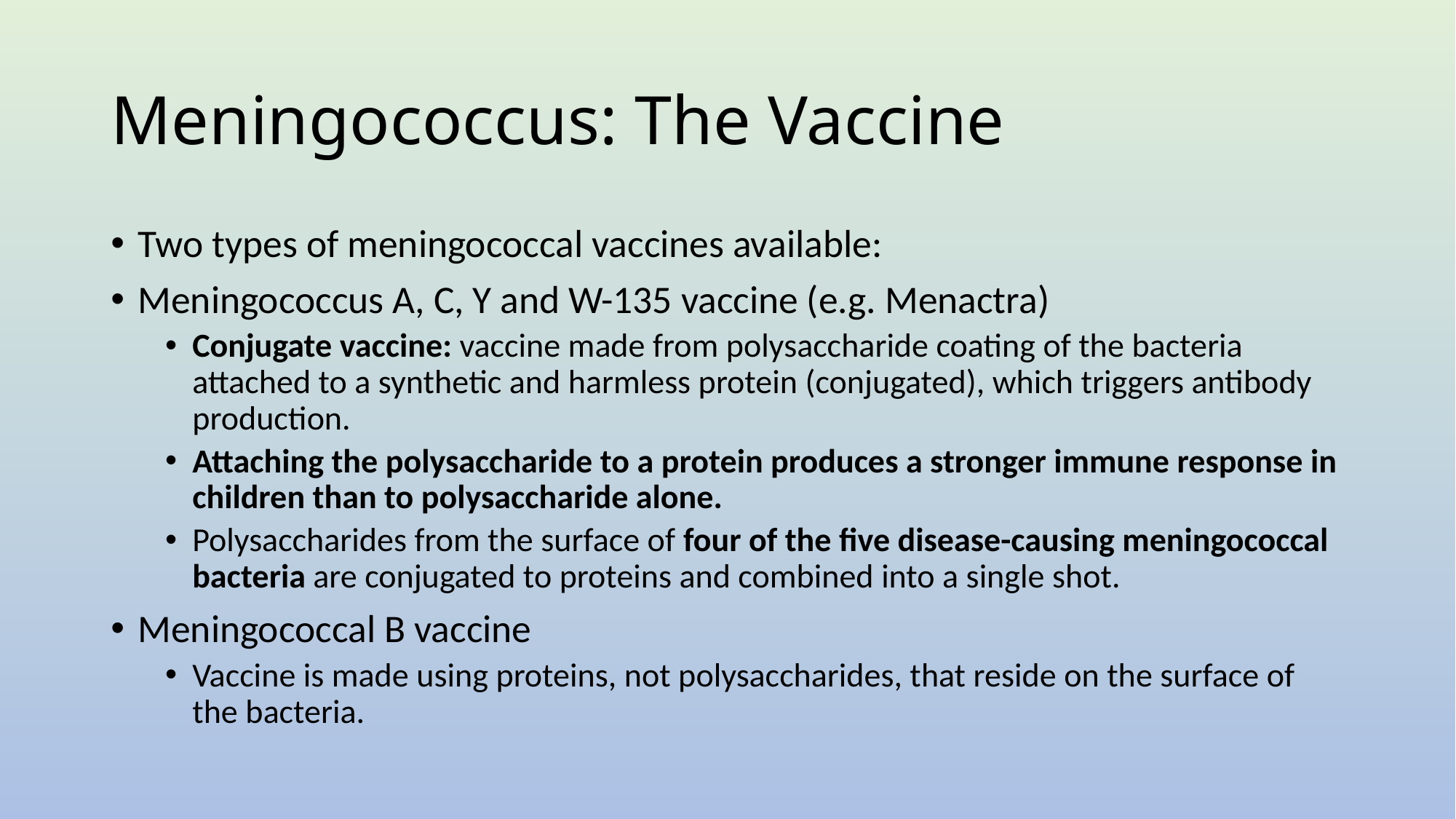

# Meningococcus: The Vaccine
Two types of meningococcal vaccines available:
Meningococcus A, C, Y and W-135 vaccine (e.g. Menactra)
Conjugate vaccine: vaccine made from polysaccharide coating of the bacteria attached to a synthetic and harmless protein (conjugated), which triggers antibody production.
Attaching the polysaccharide to a protein produces a stronger immune response in children than to polysaccharide alone.
Polysaccharides from the surface of four of the five disease-causing meningococcal bacteria are conjugated to proteins and combined into a single shot.
Meningococcal B vaccine
Vaccine is made using proteins, not polysaccharides, that reside on the surface of the bacteria.

## Slide 35
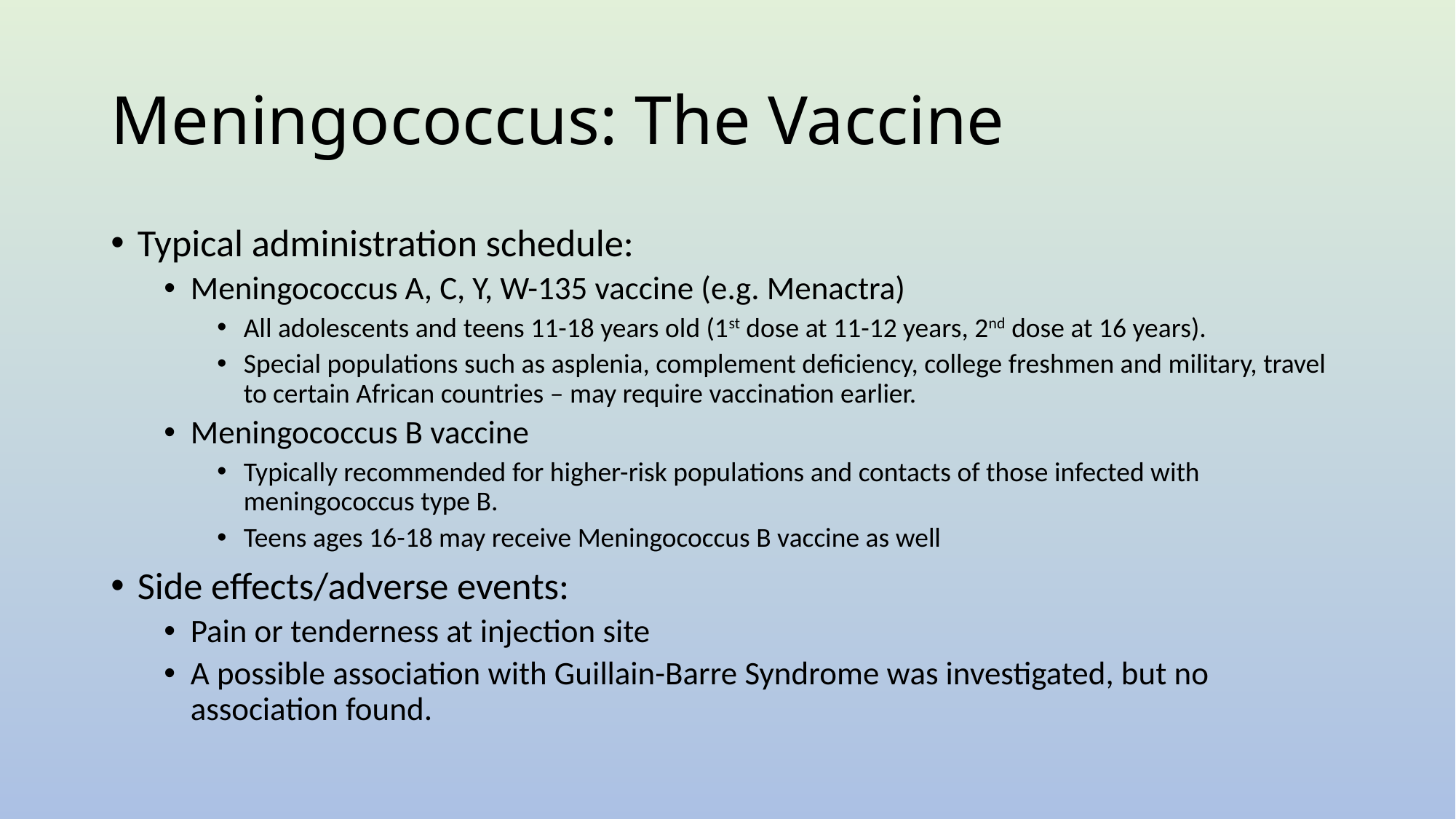

# Meningococcus: The Vaccine
Typical administration schedule:
Meningococcus A, C, Y, W-135 vaccine (e.g. Menactra)
All adolescents and teens 11-18 years old (1st dose at 11-12 years, 2nd dose at 16 years).
Special populations such as asplenia, complement deficiency, college freshmen and military, travel to certain African countries – may require vaccination earlier.
Meningococcus B vaccine
Typically recommended for higher-risk populations and contacts of those infected with meningococcus type B.
Teens ages 16-18 may receive Meningococcus B vaccine as well
Side effects/adverse events:
Pain or tenderness at injection site
A possible association with Guillain-Barre Syndrome was investigated, but no association found.

## Slide 36
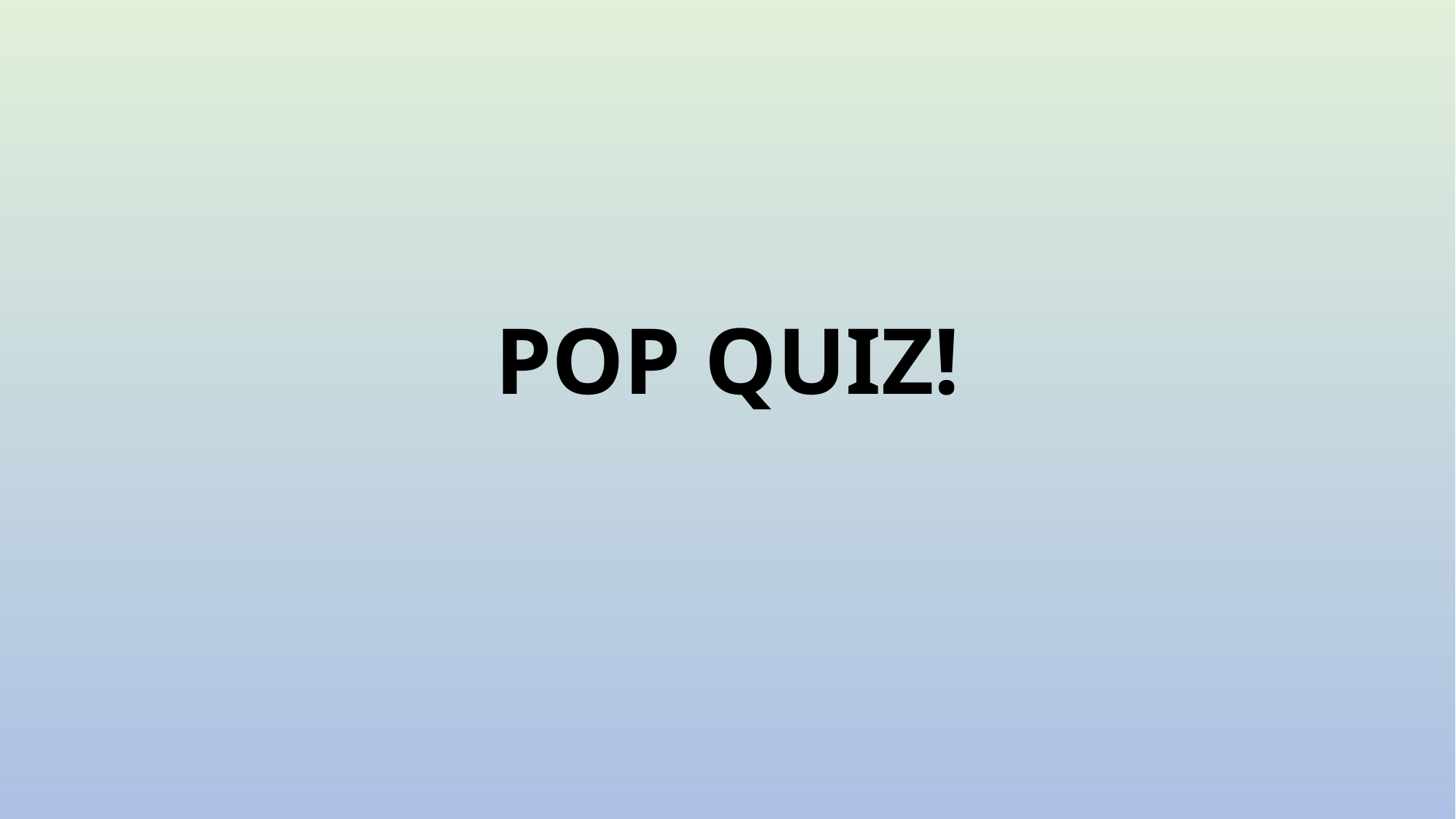

# POP QUIZ!

## Slide 37
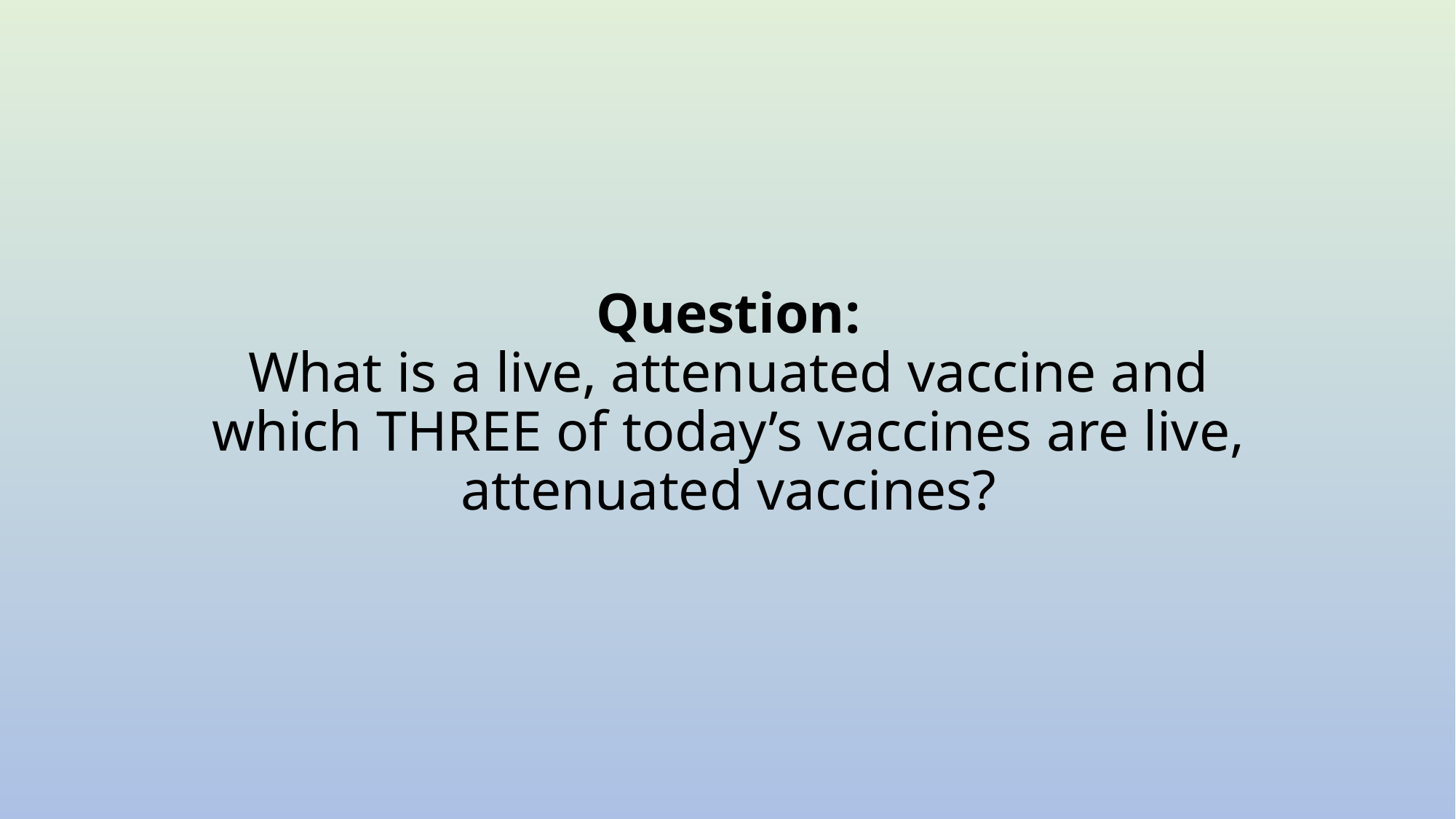

# Question: What is a live, attenuated vaccine and which THREE of today’s vaccines are live, attenuated vaccines?

## Slide 38
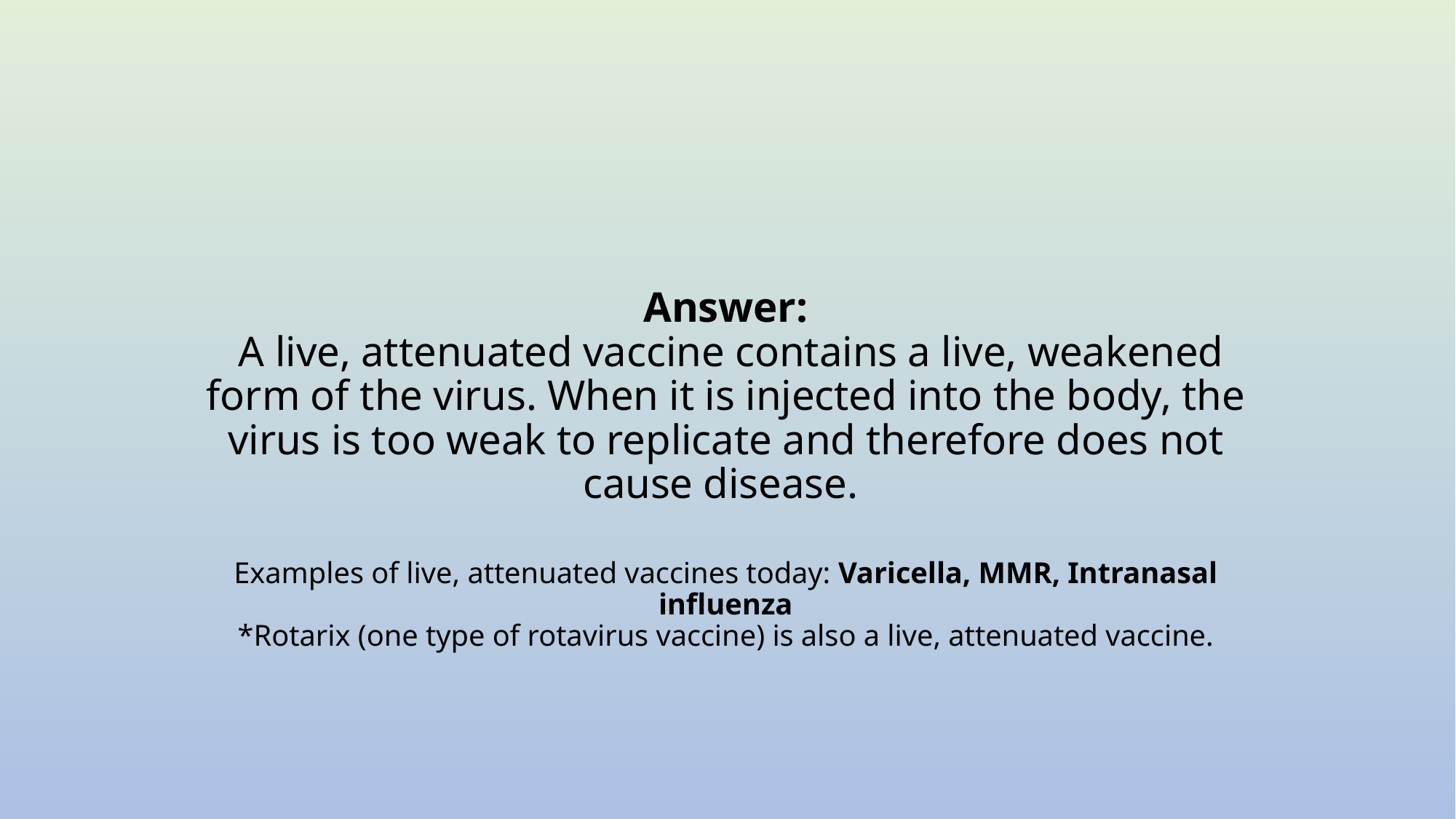

# Answer: A live, attenuated vaccine contains a live, weakened form of the virus. When it is injected into the body, the virus is too weak to replicate and therefore does not cause disease. Examples of live, attenuated vaccines today: Varicella, MMR, Intranasal influenza *Rotarix (one type of rotavirus vaccine) is also a live, attenuated vaccine.

## Slide 39
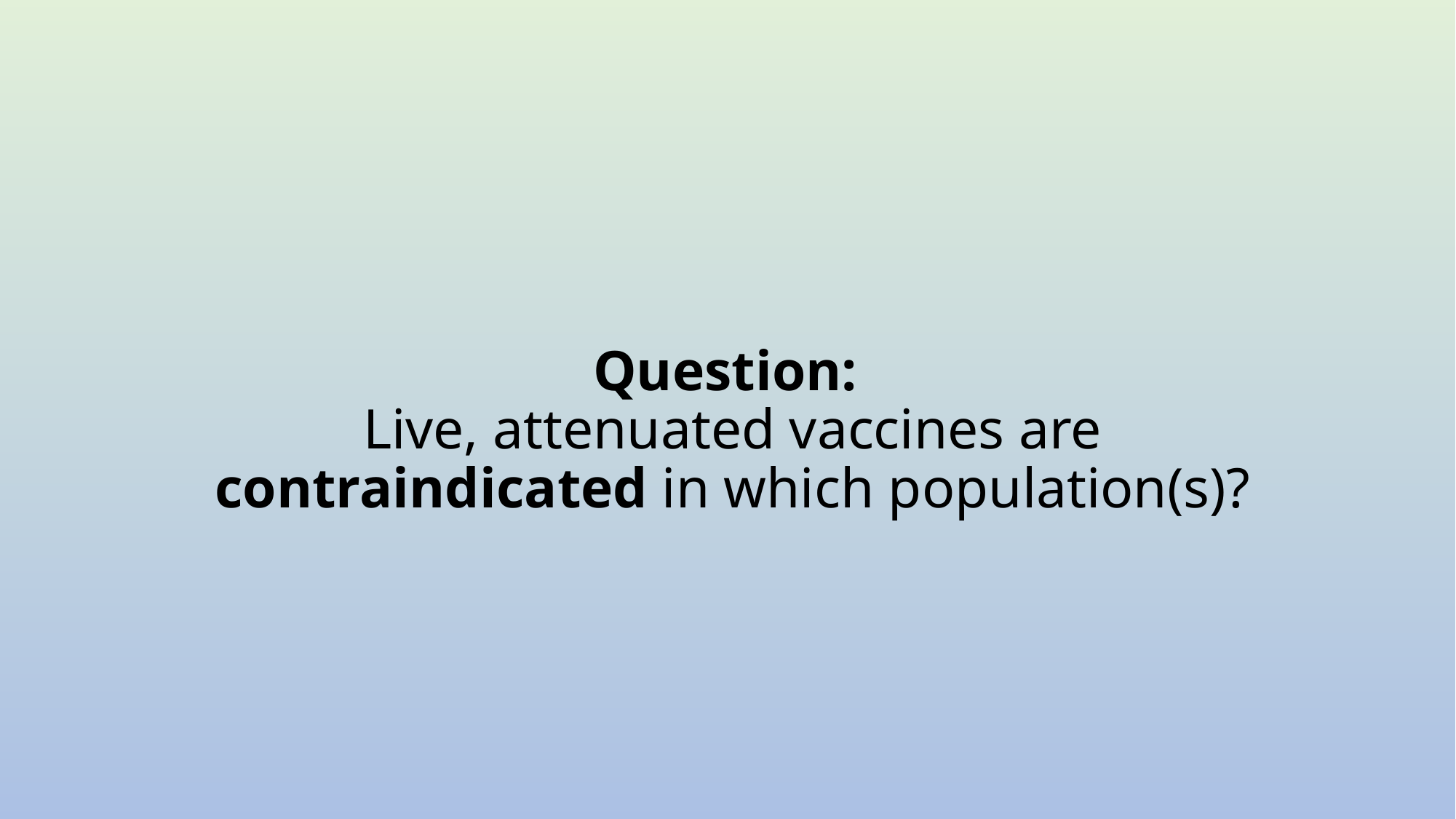

# Question: Live, attenuated vaccines are contraindicated in which population(s)?

## Slide 40
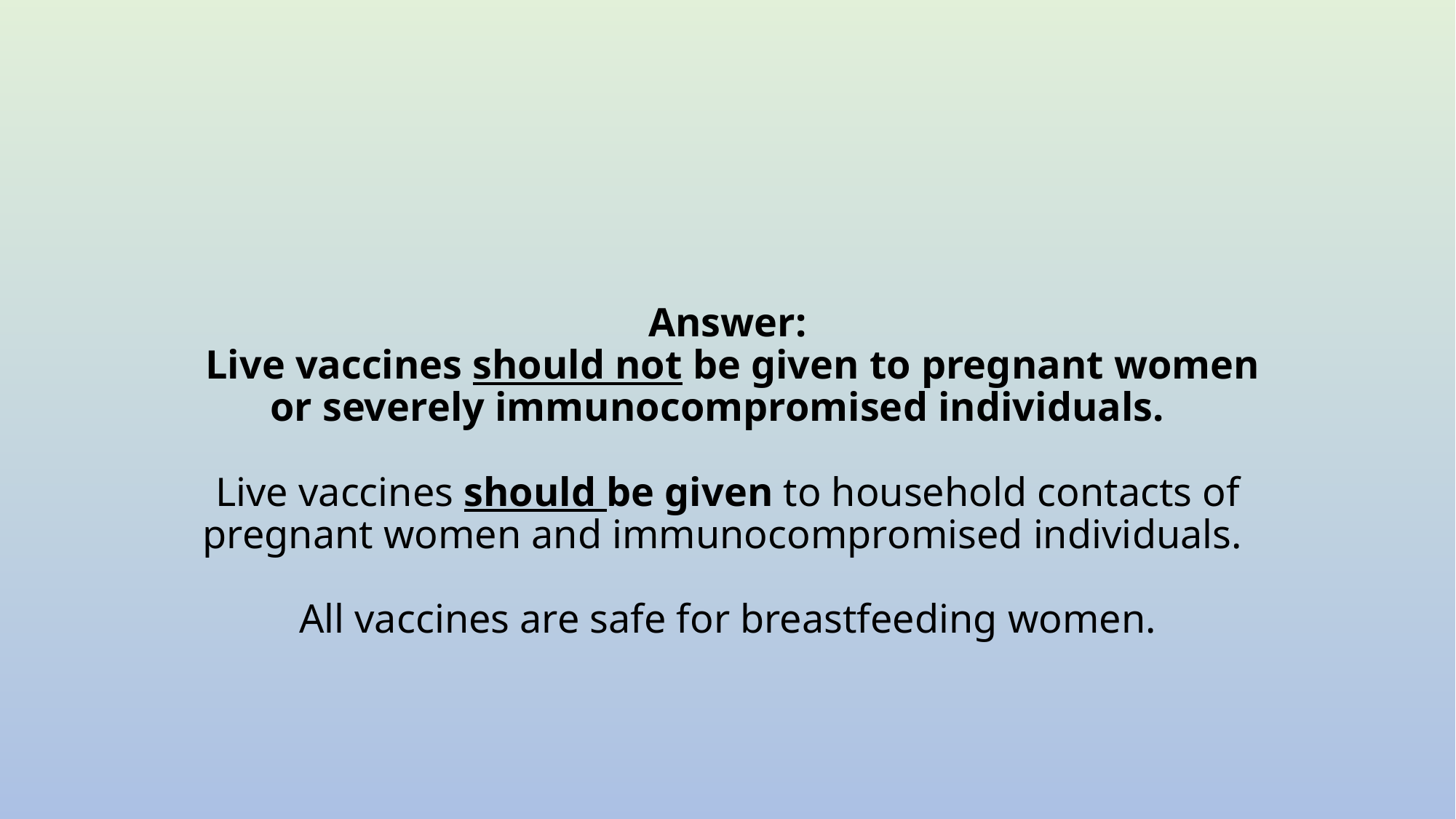

# Answer: Live vaccines should not be given to pregnant women or severely immunocompromised individuals. Live vaccines should be given to household contacts of pregnant women and immunocompromised individuals. All vaccines are safe for breastfeeding women.

## Slide 41
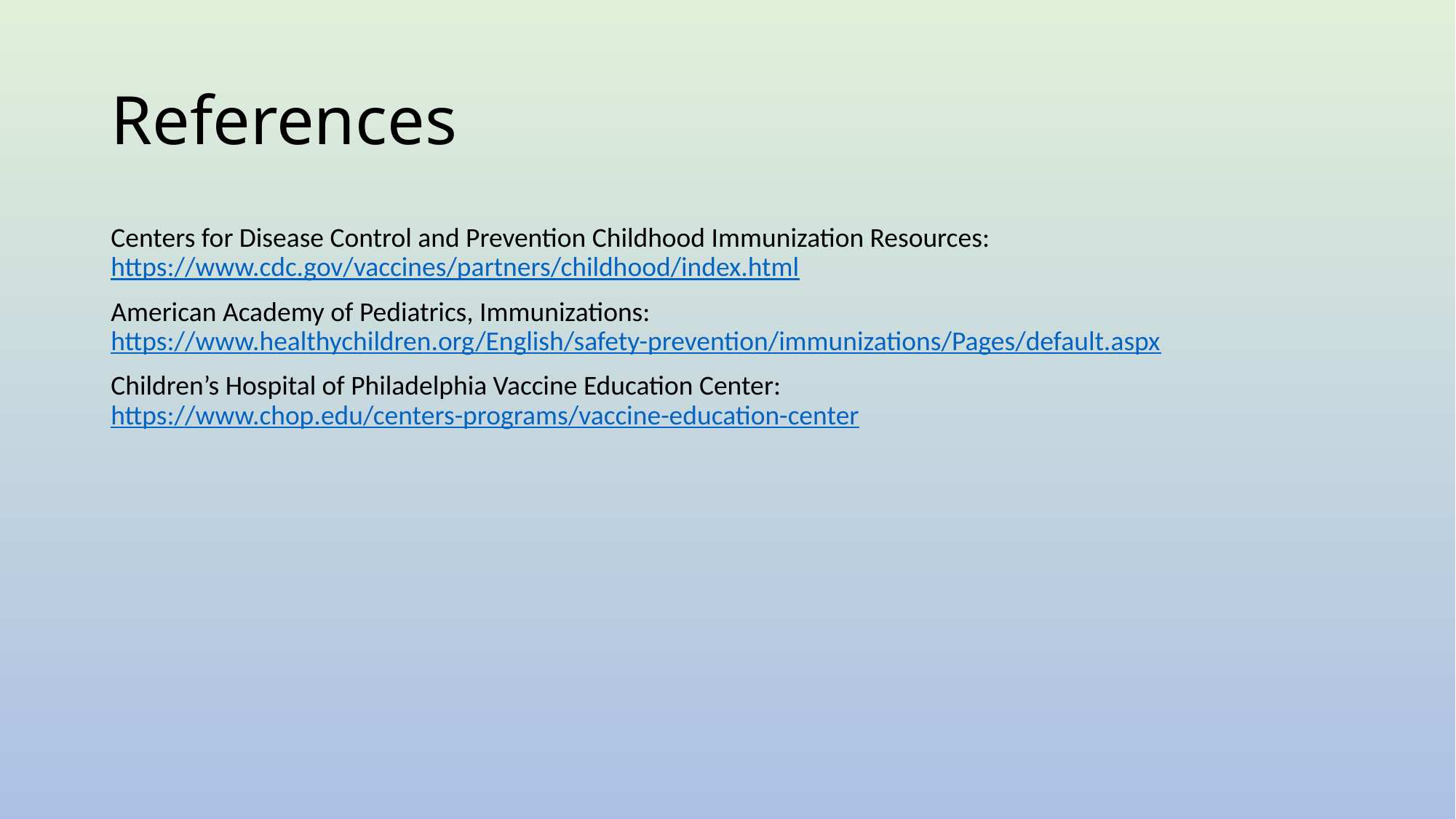

# References
Centers for Disease Control and Prevention Childhood Immunization Resources: https://www.cdc.gov/vaccines/partners/childhood/index.html
American Academy of Pediatrics, Immunizations: https://www.healthychildren.org/English/safety-prevention/immunizations/Pages/default.aspx
Children’s Hospital of Philadelphia Vaccine Education Center: https://www.chop.edu/centers-programs/vaccine-education-center
