# Supplementary material for: Addressing Vaccine Hesitancy Through a Comprehensive Resident Vaccine Curriculum
Source: MedEdPORTAL. 2022 Dec 27;18:11292. doi: 10.15766/mep_2374-8265.11292 (PMC9792628; doi:10.15766/mep_2374-8265.11292)
Supplement: Supplementary file 1 — Vaccine Curriculum Facilitator Guide.docxVaccines Part 1.pptxVaccines Part 2.pptxVaccines Part 3 - Myths and Facts.pptxVaccines Part 4 - Communication Skills.pptxVaccine Hesitancy Communication Cases.docxVaccine Pretest.docxVaccine Posttest.docxPre- and Posttest Answer Key.docxSP Case and Notes for SP.docxSP Case Development Tool.docxSP Case - Learner Version.docxSP Assessment Checklist.docx [file mep_2374-8265.11292-s001.zip › D. Vaccines Part 3 - Myths and Facts.pptx]

## Slide 1
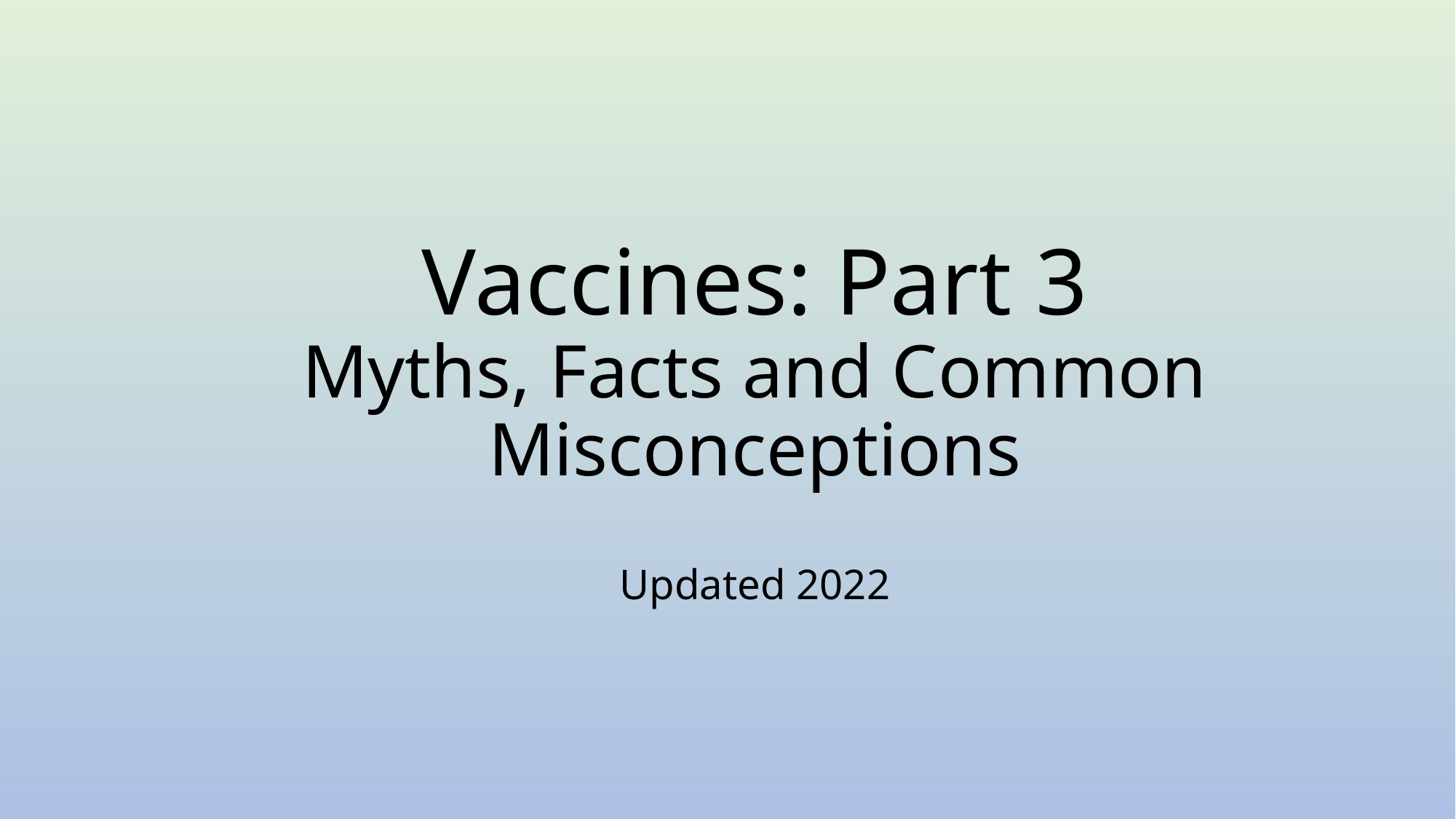

# Vaccines: Part 3 Myths, Facts and Common Misconceptions Updated 2022

## Slide 2
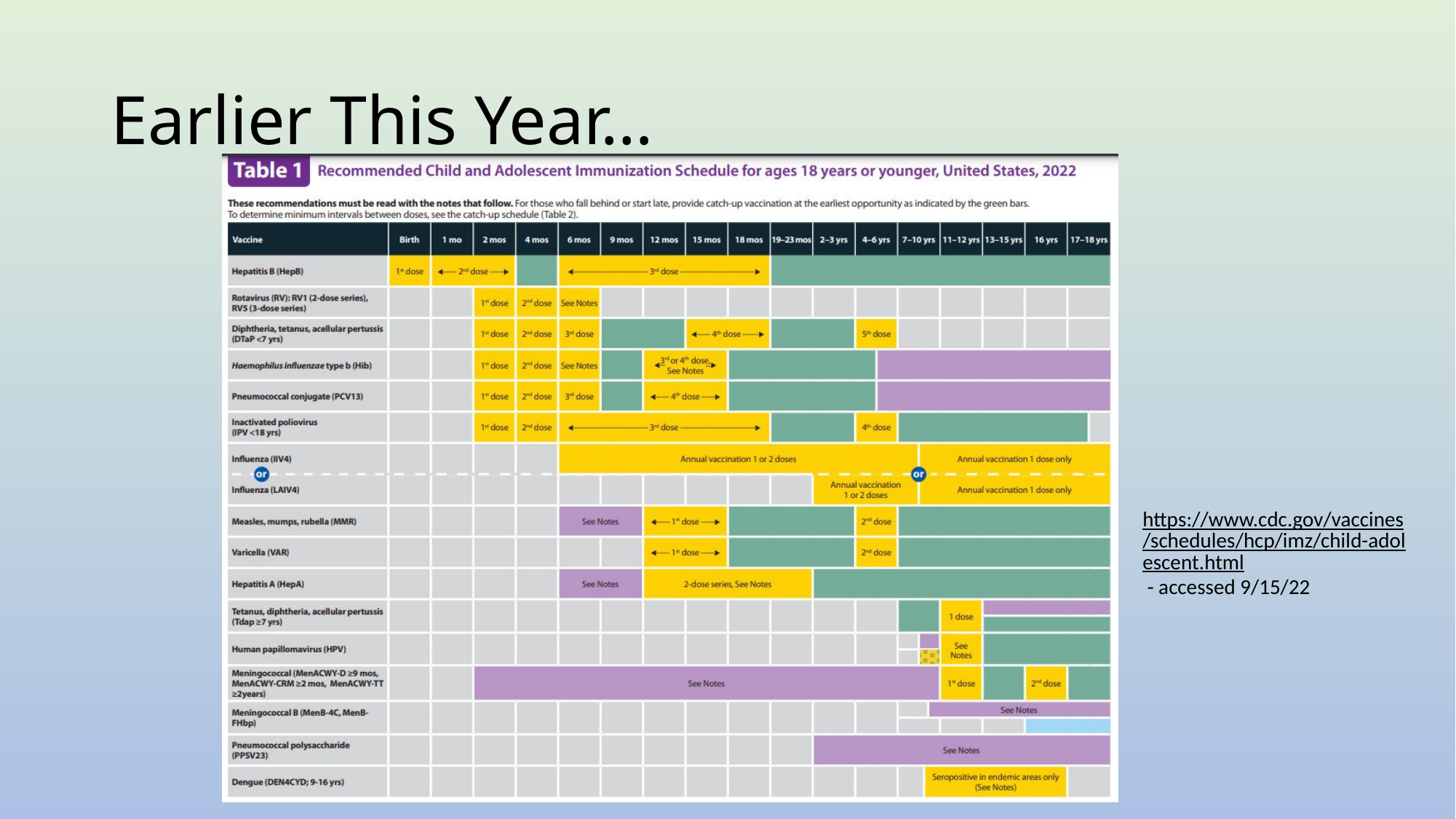

# Earlier This Year…
https://www.cdc.gov/vaccines/schedules/hcp/imz/child-adolescent.html - accessed 9/15/22

## Slide 3
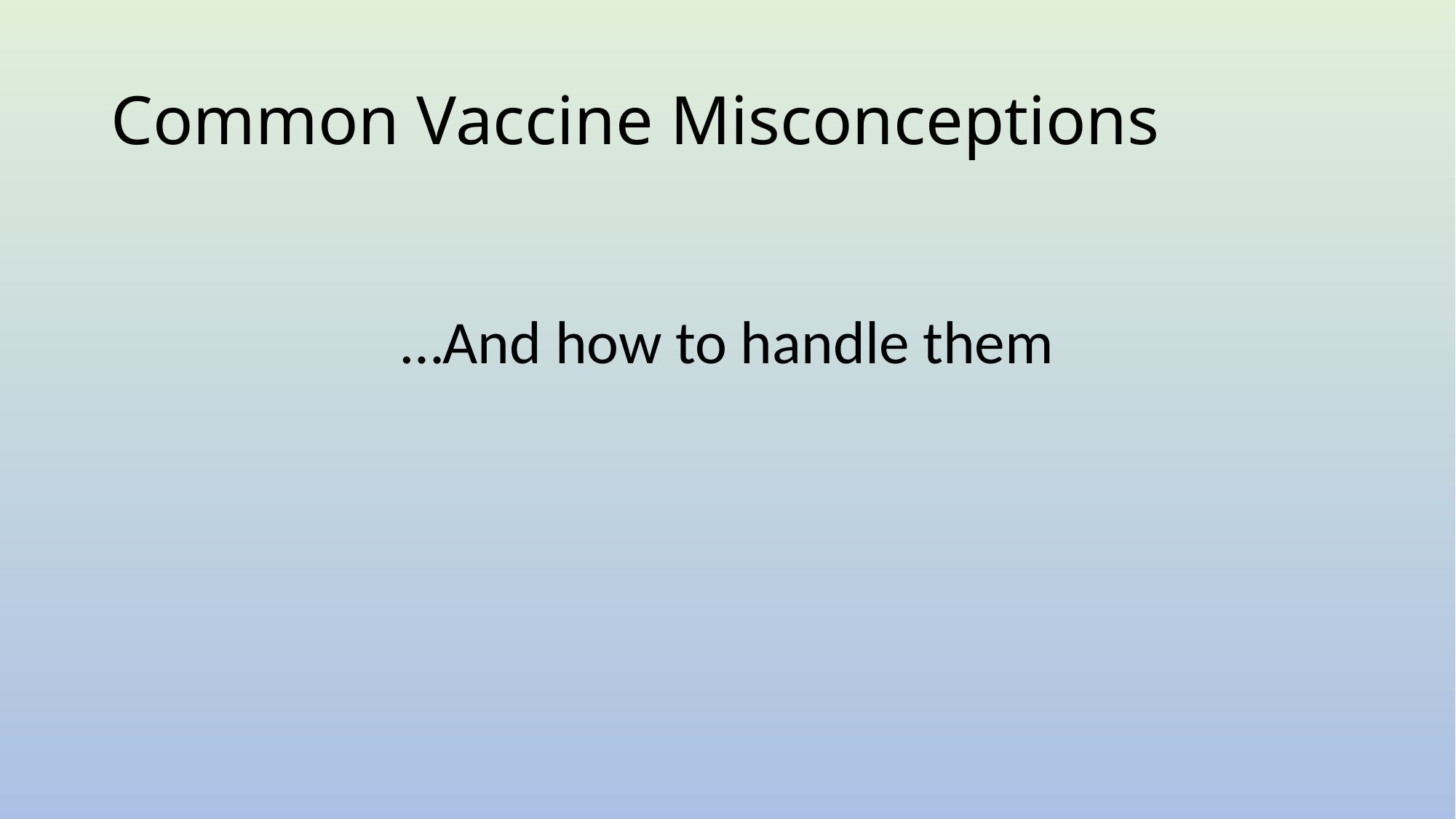

# Common Vaccine Misconceptions
…And how to handle them

## Slide 4
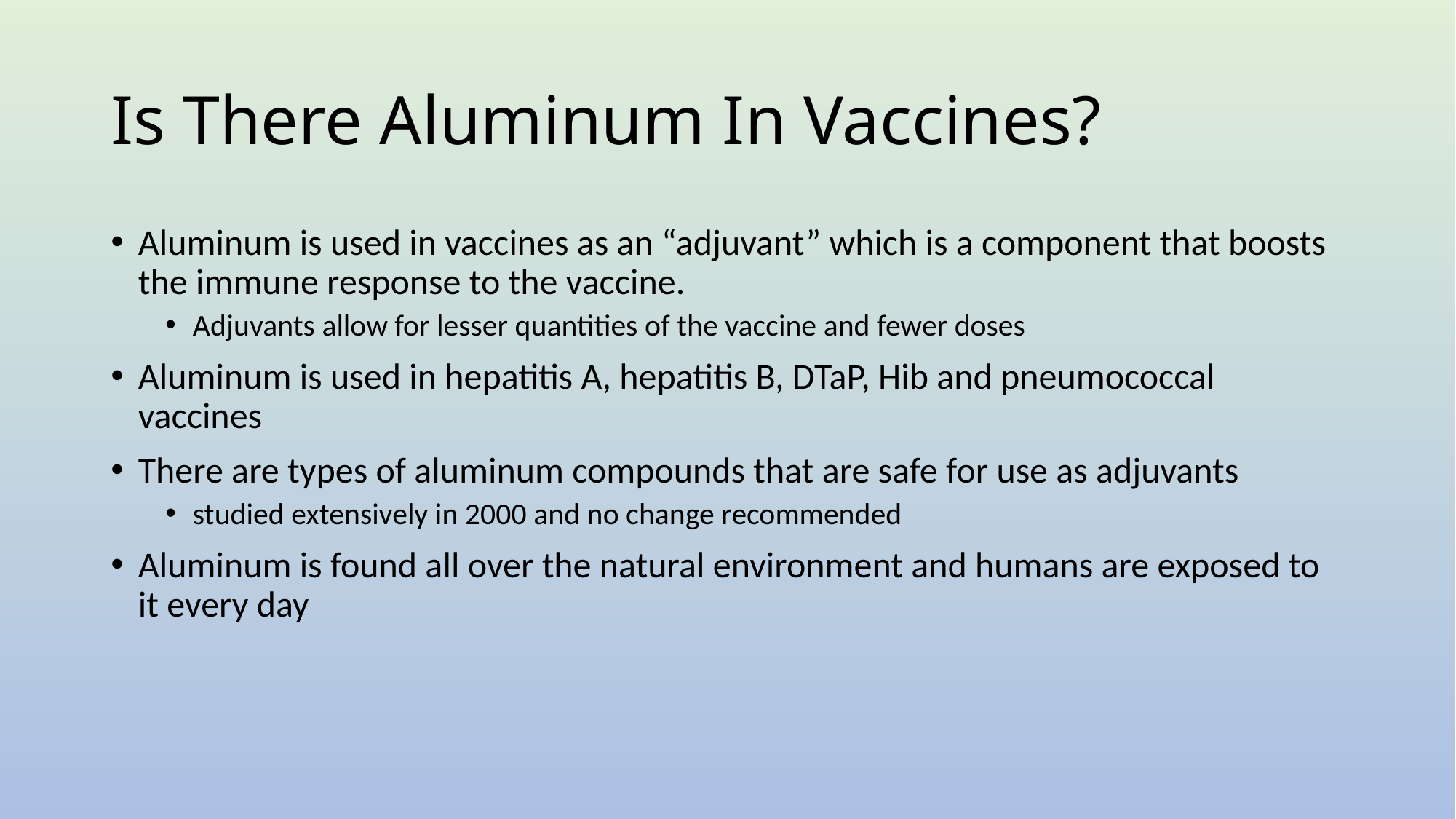

# Is There Aluminum In Vaccines?
Aluminum is used in vaccines as an “adjuvant” which is a component that boosts the immune response to the vaccine.
Adjuvants allow for lesser quantities of the vaccine and fewer doses
Aluminum is used in hepatitis A, hepatitis B, DTaP, Hib and pneumococcal vaccines
There are types of aluminum compounds that are safe for use as adjuvants
studied extensively in 2000 and no change recommended
Aluminum is found all over the natural environment and humans are exposed to it every day

## Slide 5
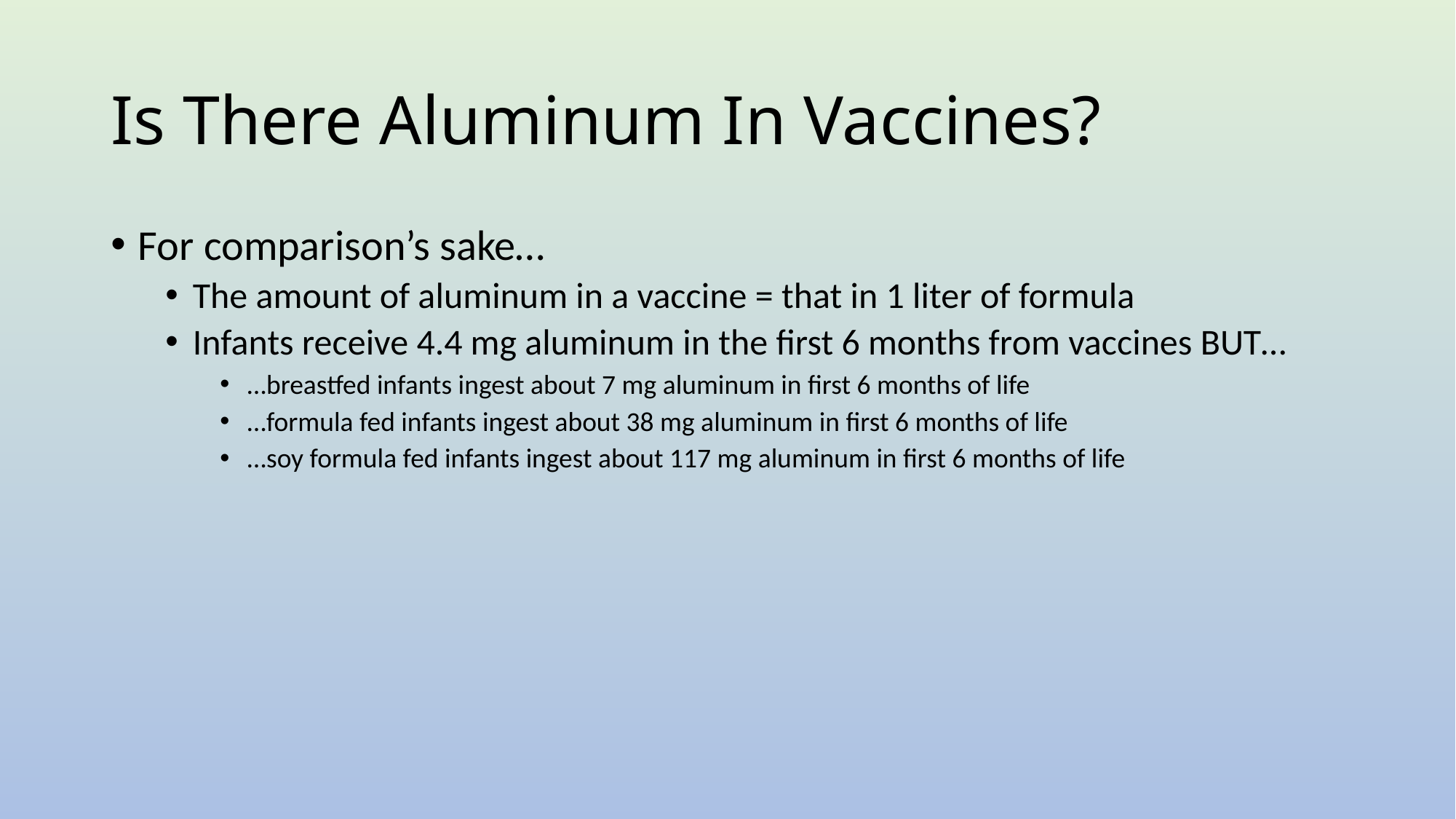

# Is There Aluminum In Vaccines?
For comparison’s sake…
The amount of aluminum in a vaccine = that in 1 liter of formula
Infants receive 4.4 mg aluminum in the first 6 months from vaccines BUT…
…breastfed infants ingest about 7 mg aluminum in first 6 months of life
…formula fed infants ingest about 38 mg aluminum in first 6 months of life
…soy formula fed infants ingest about 117 mg aluminum in first 6 months of life

## Slide 6
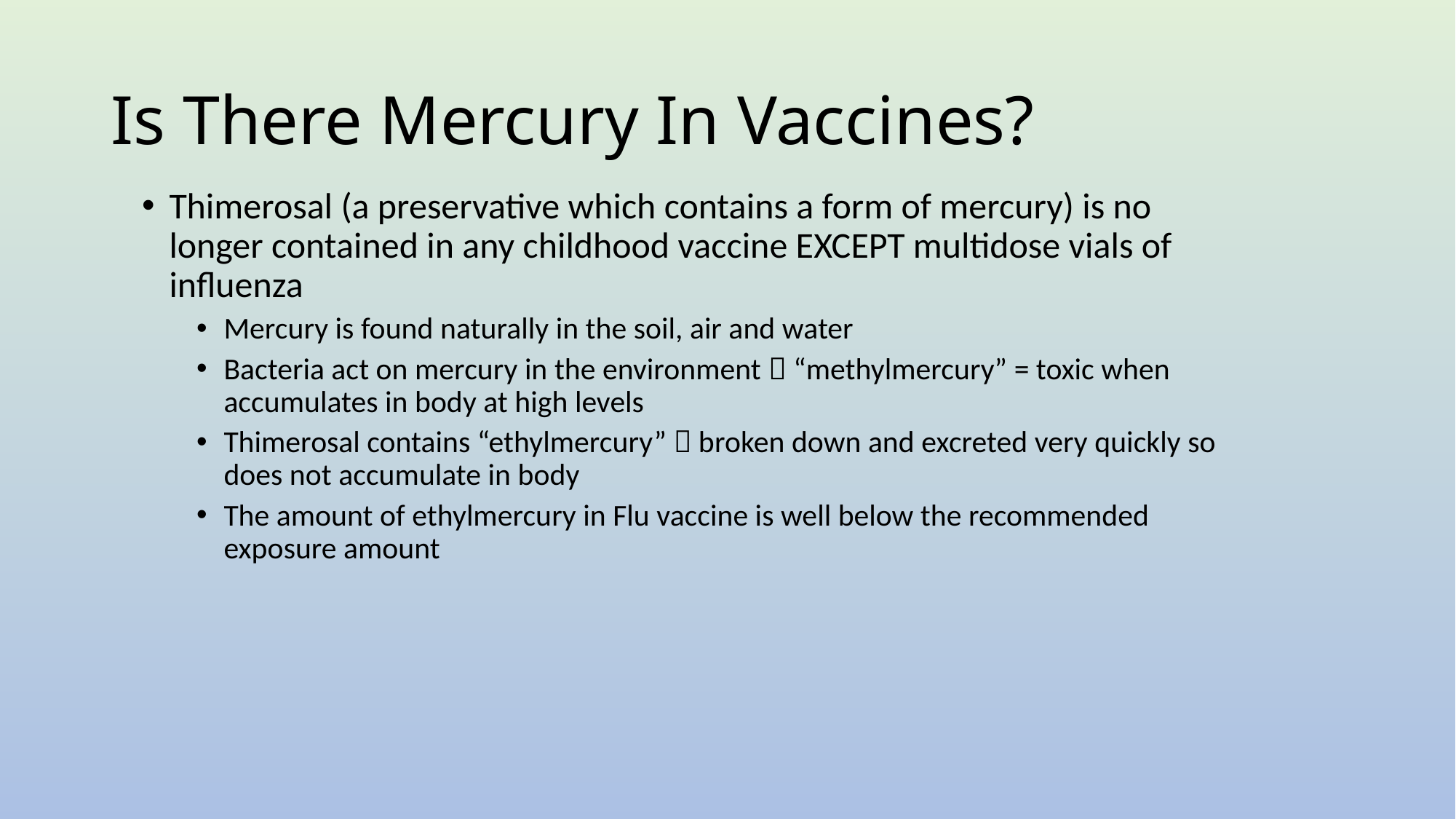

# Is There Mercury In Vaccines?
Thimerosal (a preservative which contains a form of mercury) is no longer contained in any childhood vaccine EXCEPT multidose vials of influenza
Mercury is found naturally in the soil, air and water
Bacteria act on mercury in the environment  “methylmercury” = toxic when accumulates in body at high levels
Thimerosal contains “ethylmercury”  broken down and excreted very quickly so does not accumulate in body
The amount of ethylmercury in Flu vaccine is well below the recommended exposure amount

## Slide 7
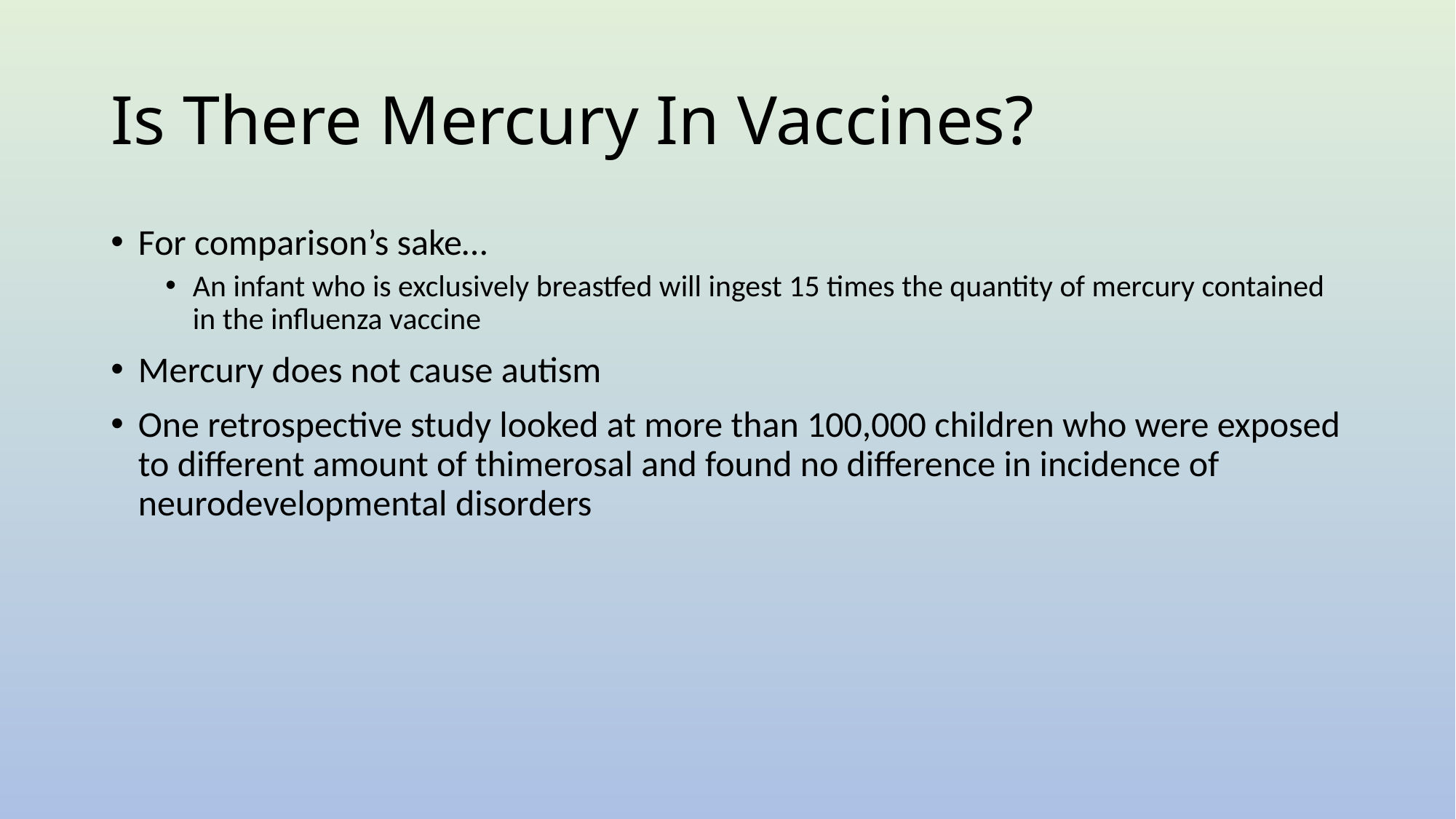

# Is There Mercury In Vaccines?
For comparison’s sake…
An infant who is exclusively breastfed will ingest 15 times the quantity of mercury contained in the influenza vaccine
Mercury does not cause autism
One retrospective study looked at more than 100,000 children who were exposed to different amount of thimerosal and found no difference in incidence of neurodevelopmental disorders

## Slide 8
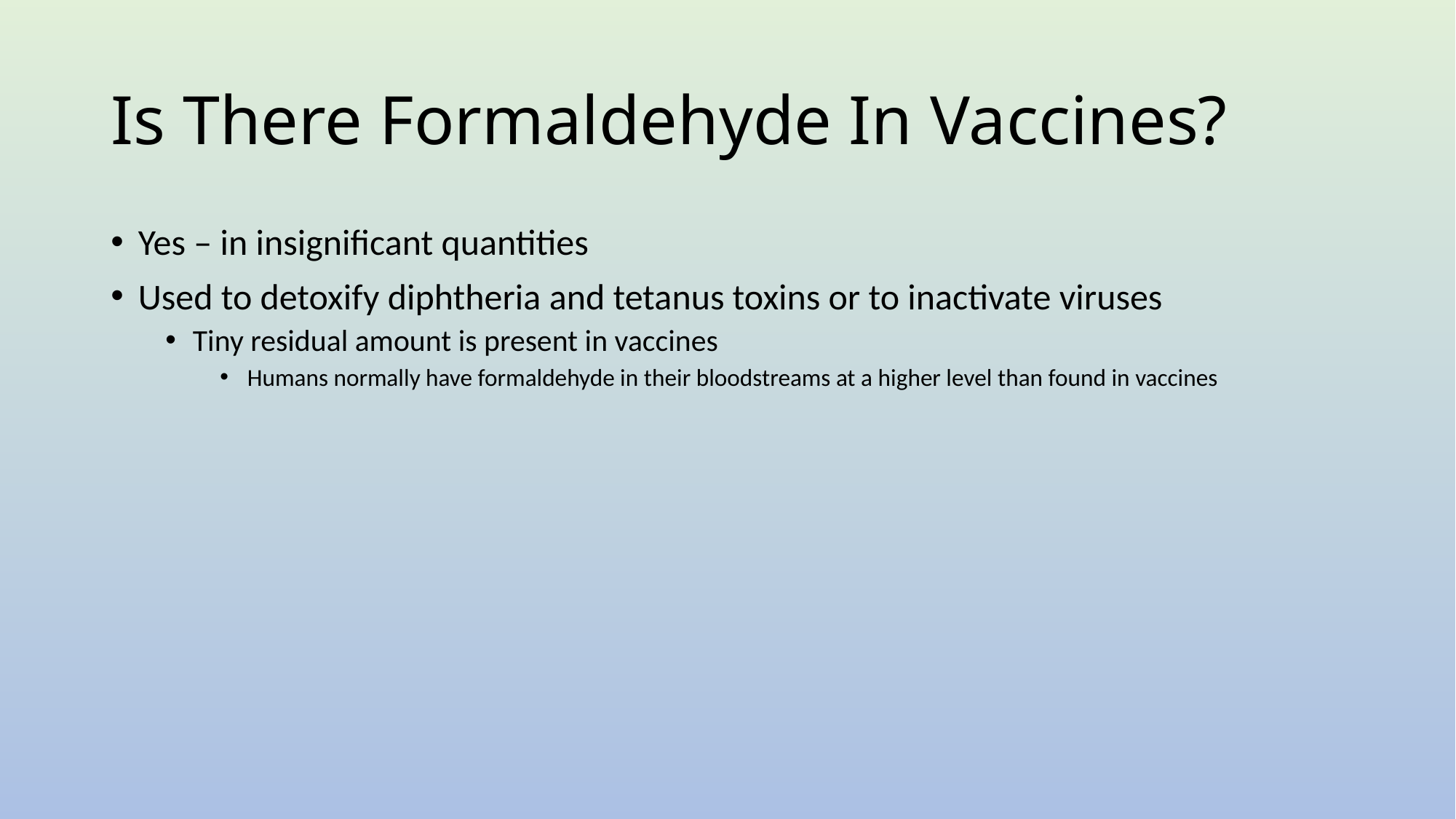

# Is There Formaldehyde In Vaccines?
Yes – in insignificant quantities
Used to detoxify diphtheria and tetanus toxins or to inactivate viruses
Tiny residual amount is present in vaccines
Humans normally have formaldehyde in their bloodstreams at a higher level than found in vaccines

## Slide 9
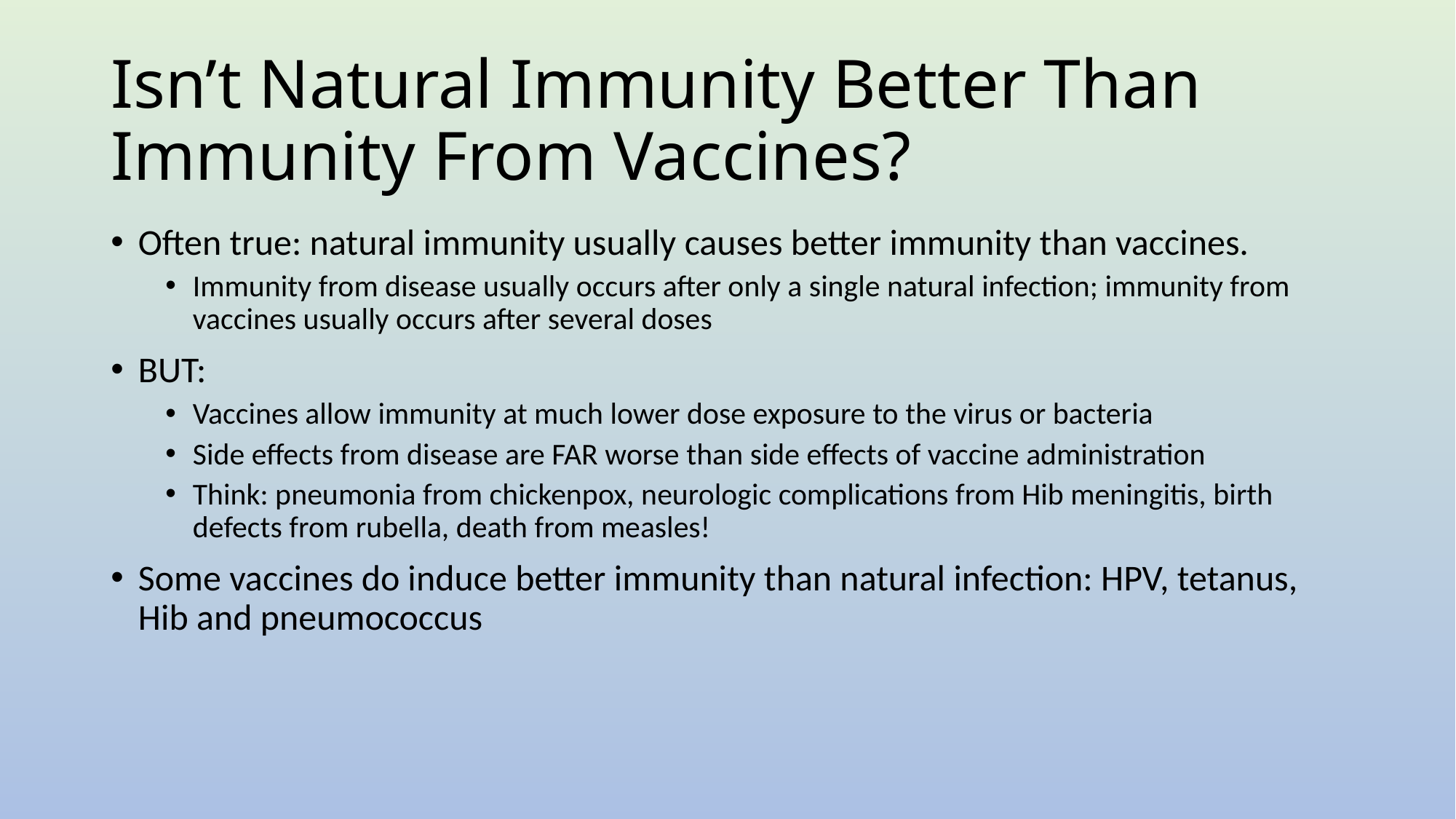

# Isn’t Natural Immunity Better Than Immunity From Vaccines?
Often true: natural immunity usually causes better immunity than vaccines.
Immunity from disease usually occurs after only a single natural infection; immunity from vaccines usually occurs after several doses
BUT:
Vaccines allow immunity at much lower dose exposure to the virus or bacteria
Side effects from disease are FAR worse than side effects of vaccine administration
Think: pneumonia from chickenpox, neurologic complications from Hib meningitis, birth defects from rubella, death from measles!
Some vaccines do induce better immunity than natural infection: HPV, tetanus, Hib and pneumococcus

## Slide 10
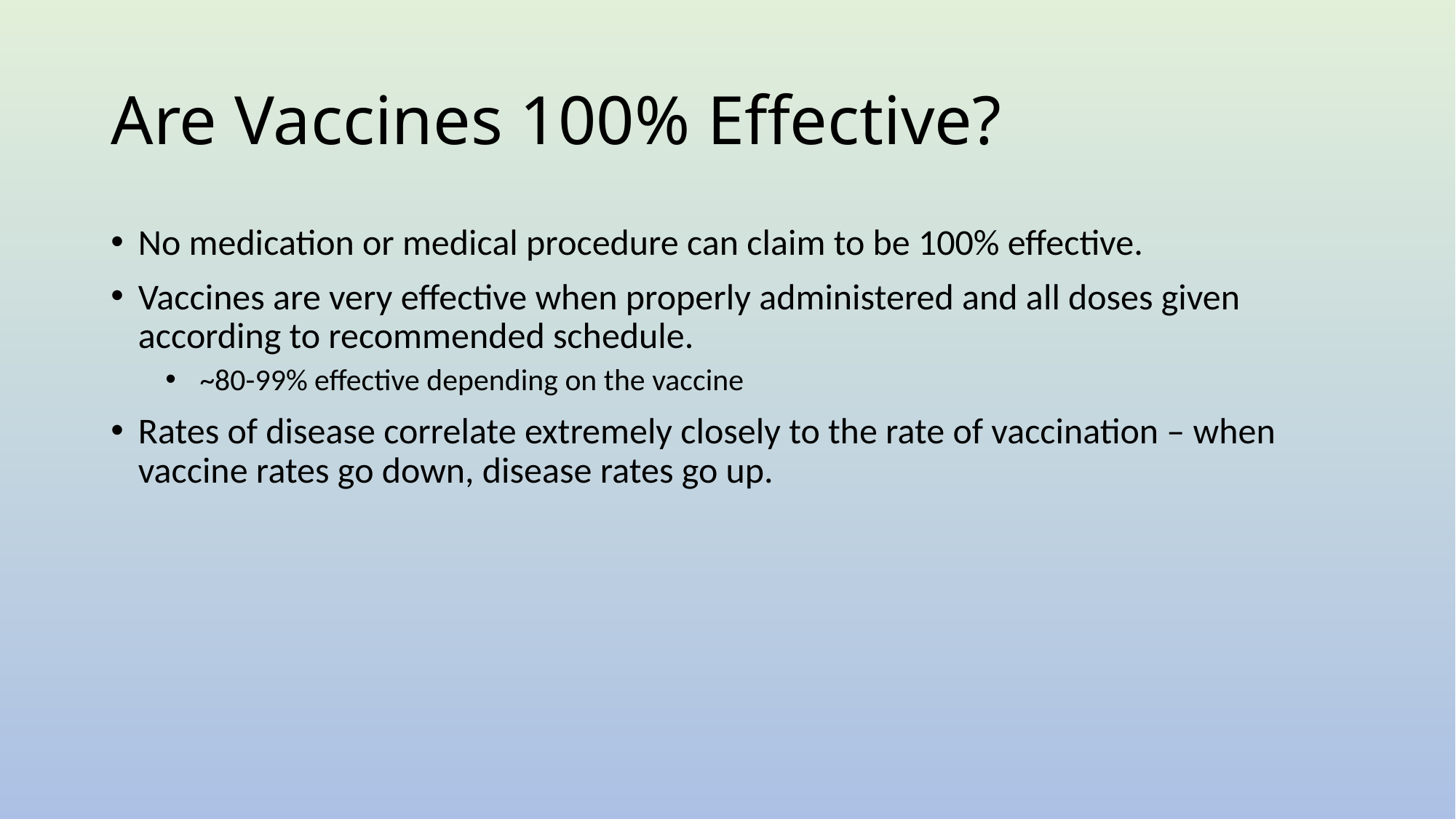

# Are Vaccines 100% Effective?
No medication or medical procedure can claim to be 100% effective.
Vaccines are very effective when properly administered and all doses given according to recommended schedule.
 ~80-99% effective depending on the vaccine
Rates of disease correlate extremely closely to the rate of vaccination – when vaccine rates go down, disease rates go up.

## Slide 11
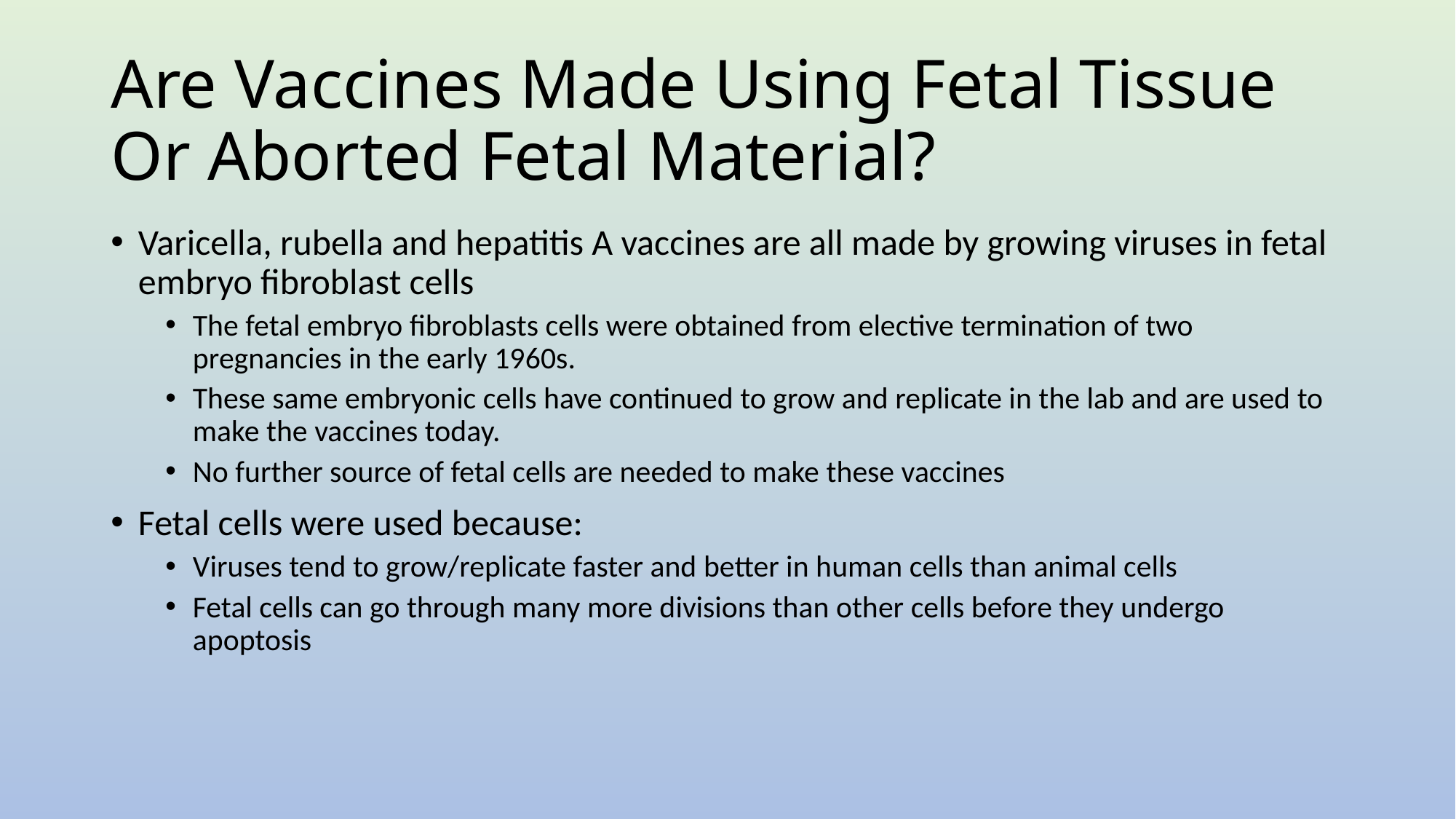

# Are Vaccines Made Using Fetal Tissue Or Aborted Fetal Material?
Varicella, rubella and hepatitis A vaccines are all made by growing viruses in fetal embryo fibroblast cells
The fetal embryo fibroblasts cells were obtained from elective termination of two pregnancies in the early 1960s.
These same embryonic cells have continued to grow and replicate in the lab and are used to make the vaccines today.
No further source of fetal cells are needed to make these vaccines
Fetal cells were used because:
Viruses tend to grow/replicate faster and better in human cells than animal cells
Fetal cells can go through many more divisions than other cells before they undergo apoptosis

## Slide 12
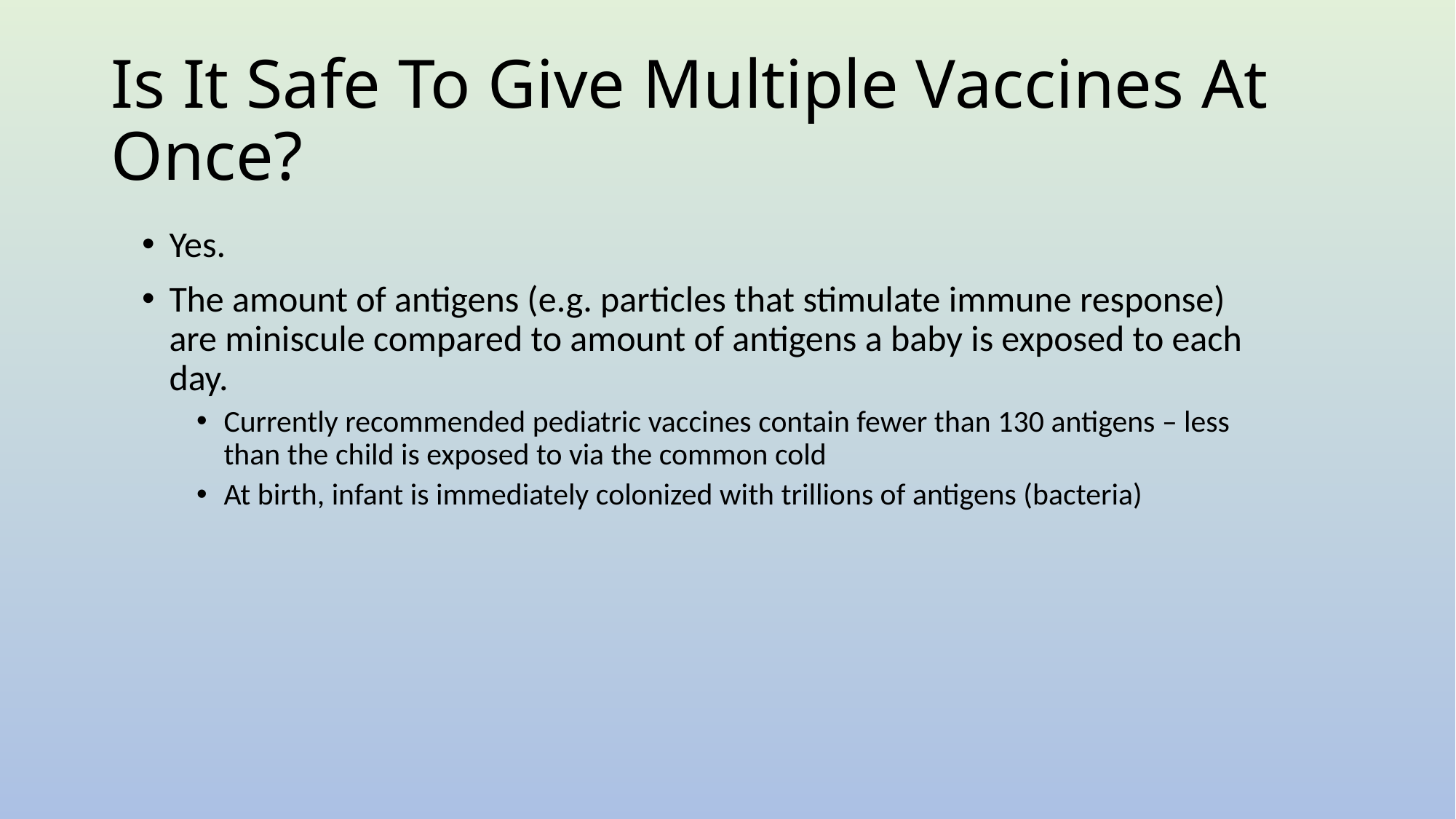

# Is It Safe To Give Multiple Vaccines At Once?
Yes.
The amount of antigens (e.g. particles that stimulate immune response) are miniscule compared to amount of antigens a baby is exposed to each day.
Currently recommended pediatric vaccines contain fewer than 130 antigens – less than the child is exposed to via the common cold
At birth, infant is immediately colonized with trillions of antigens (bacteria)

## Slide 13
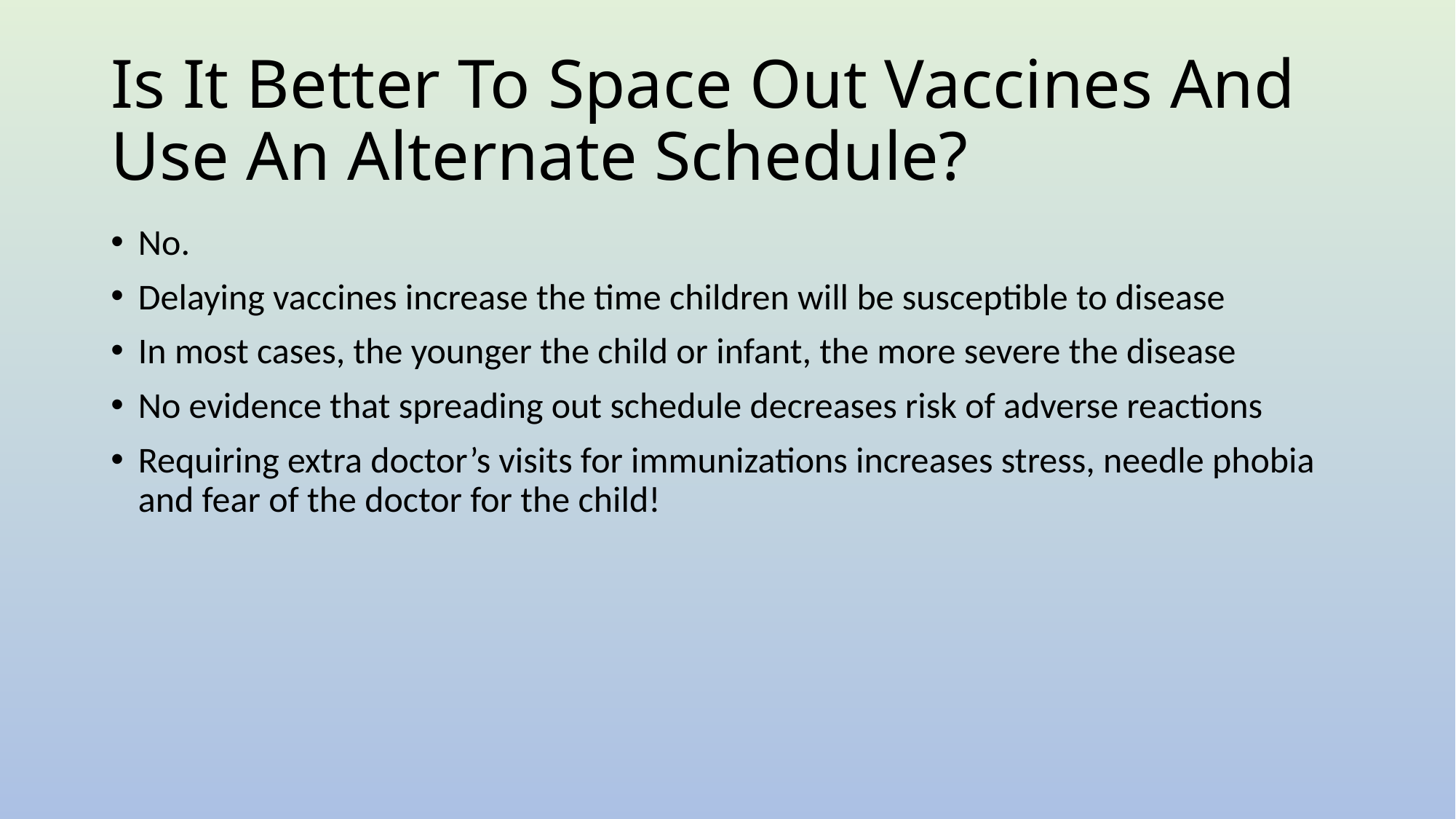

# Is It Better To Space Out Vaccines And Use An Alternate Schedule?
No.
Delaying vaccines increase the time children will be susceptible to disease
In most cases, the younger the child or infant, the more severe the disease
No evidence that spreading out schedule decreases risk of adverse reactions
Requiring extra doctor’s visits for immunizations increases stress, needle phobia and fear of the doctor for the child!

## Slide 14
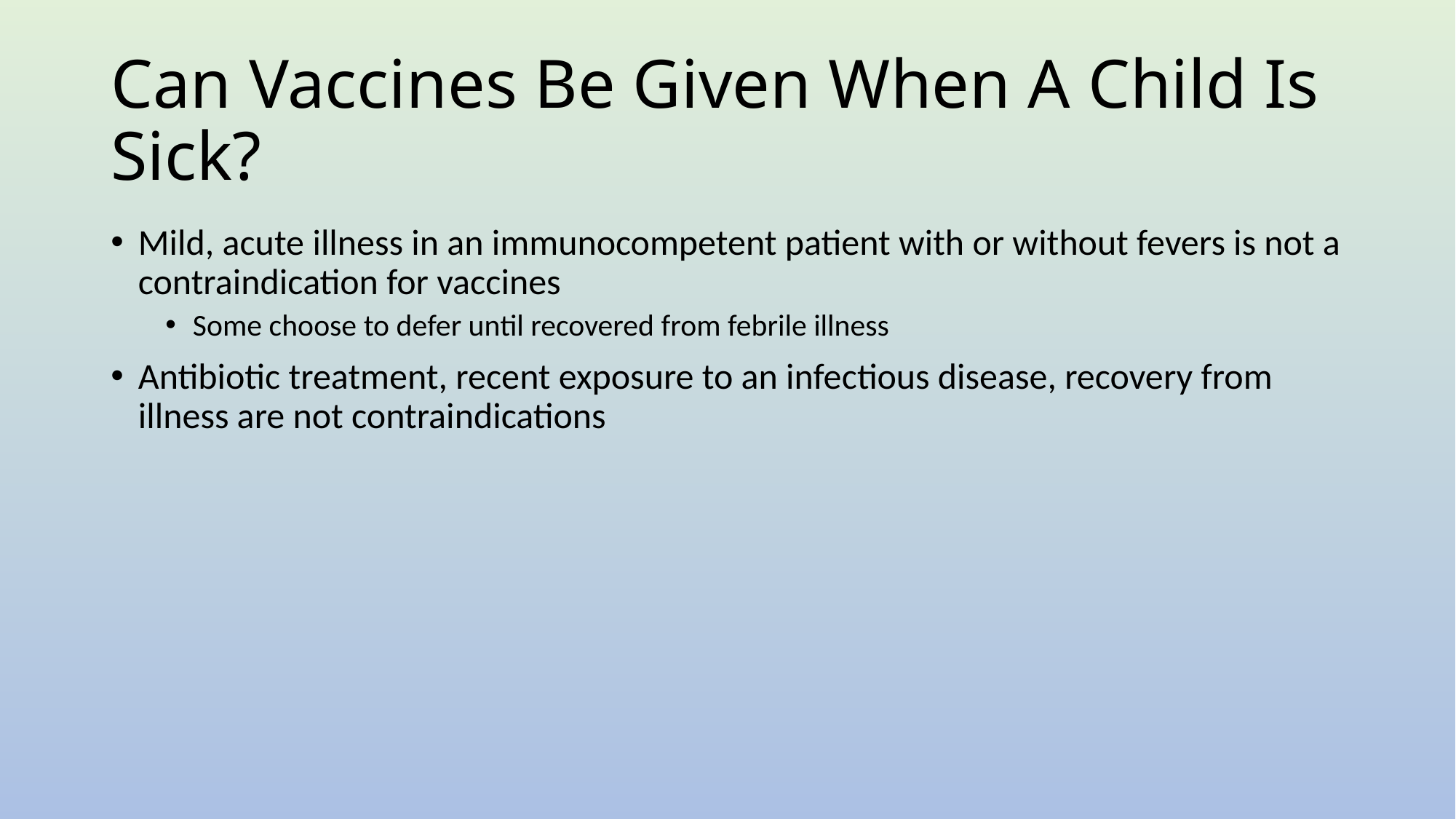

# Can Vaccines Be Given When A Child Is Sick?
Mild, acute illness in an immunocompetent patient with or without fevers is not a contraindication for vaccines
Some choose to defer until recovered from febrile illness
Antibiotic treatment, recent exposure to an infectious disease, recovery from illness are not contraindications

## Slide 15
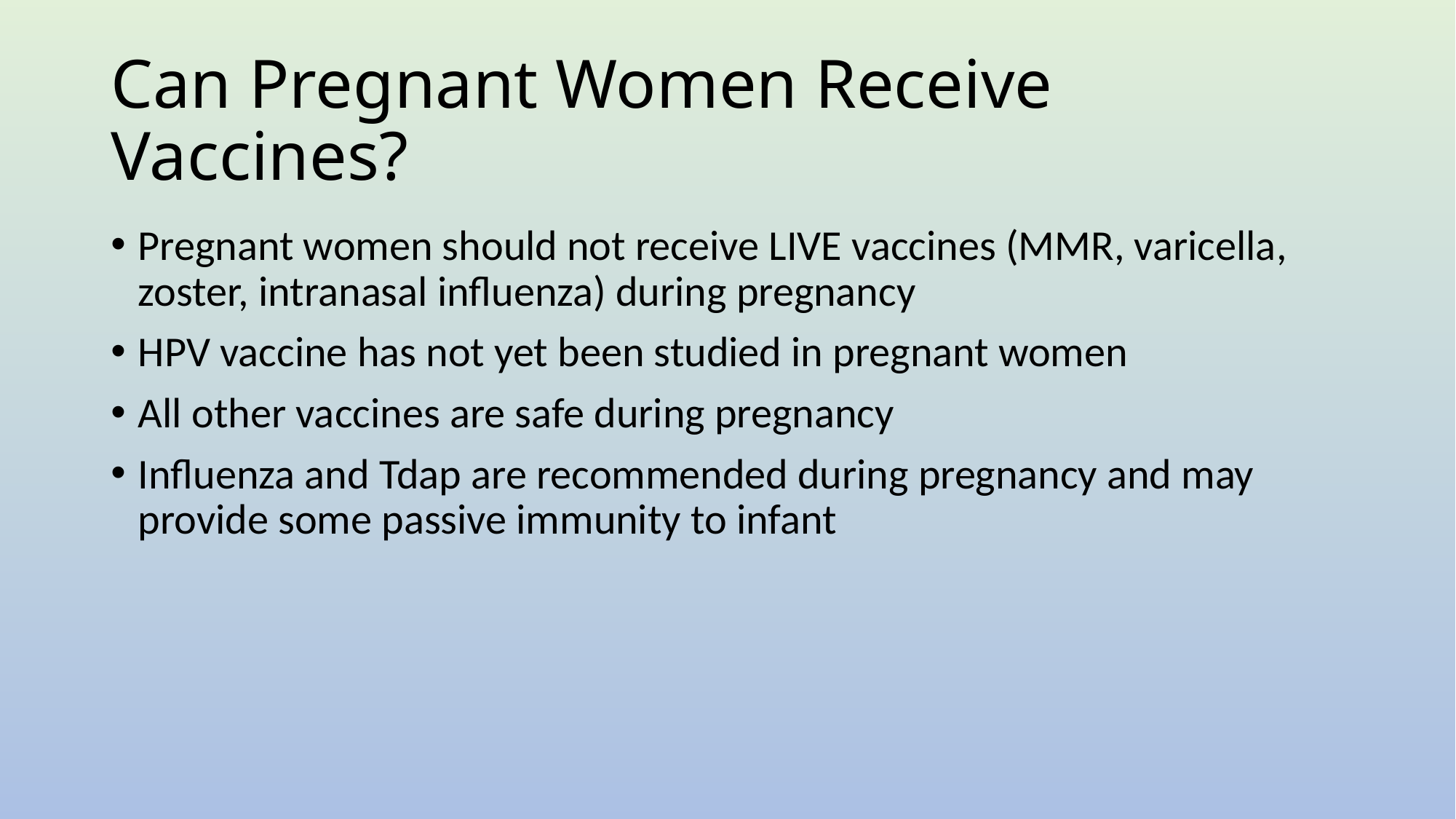

# Can Pregnant Women Receive Vaccines?
Pregnant women should not receive LIVE vaccines (MMR, varicella, zoster, intranasal influenza) during pregnancy
HPV vaccine has not yet been studied in pregnant women
All other vaccines are safe during pregnancy
Influenza and Tdap are recommended during pregnancy and may provide some passive immunity to infant

## Slide 16
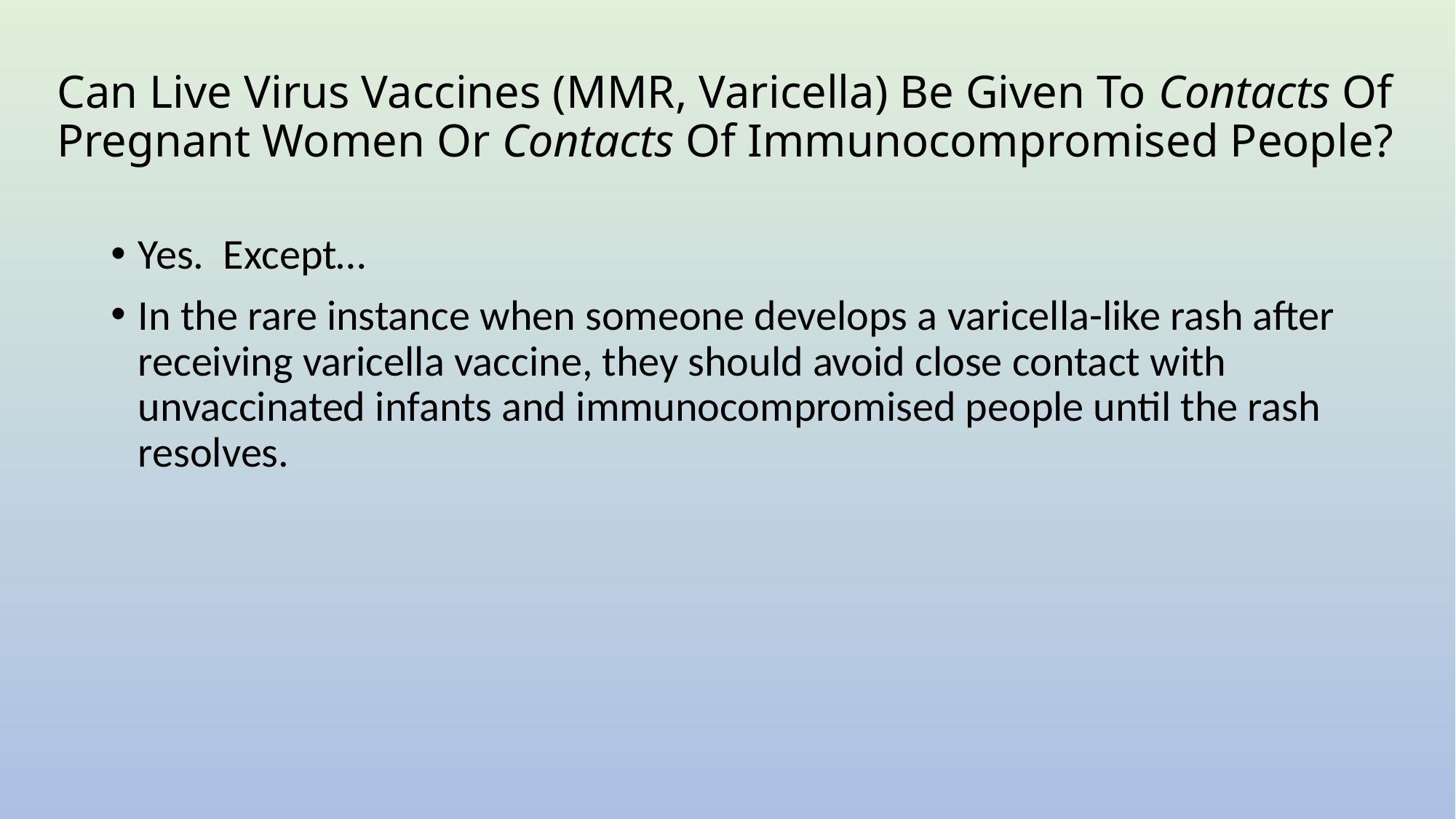

# Can Live Virus Vaccines (MMR, Varicella) Be Given To Contacts Of Pregnant Women Or Contacts Of Immunocompromised People?
Yes. Except…
In the rare instance when someone develops a varicella-like rash after receiving varicella vaccine, they should avoid close contact with unvaccinated infants and immunocompromised people until the rash resolves.

## Slide 17
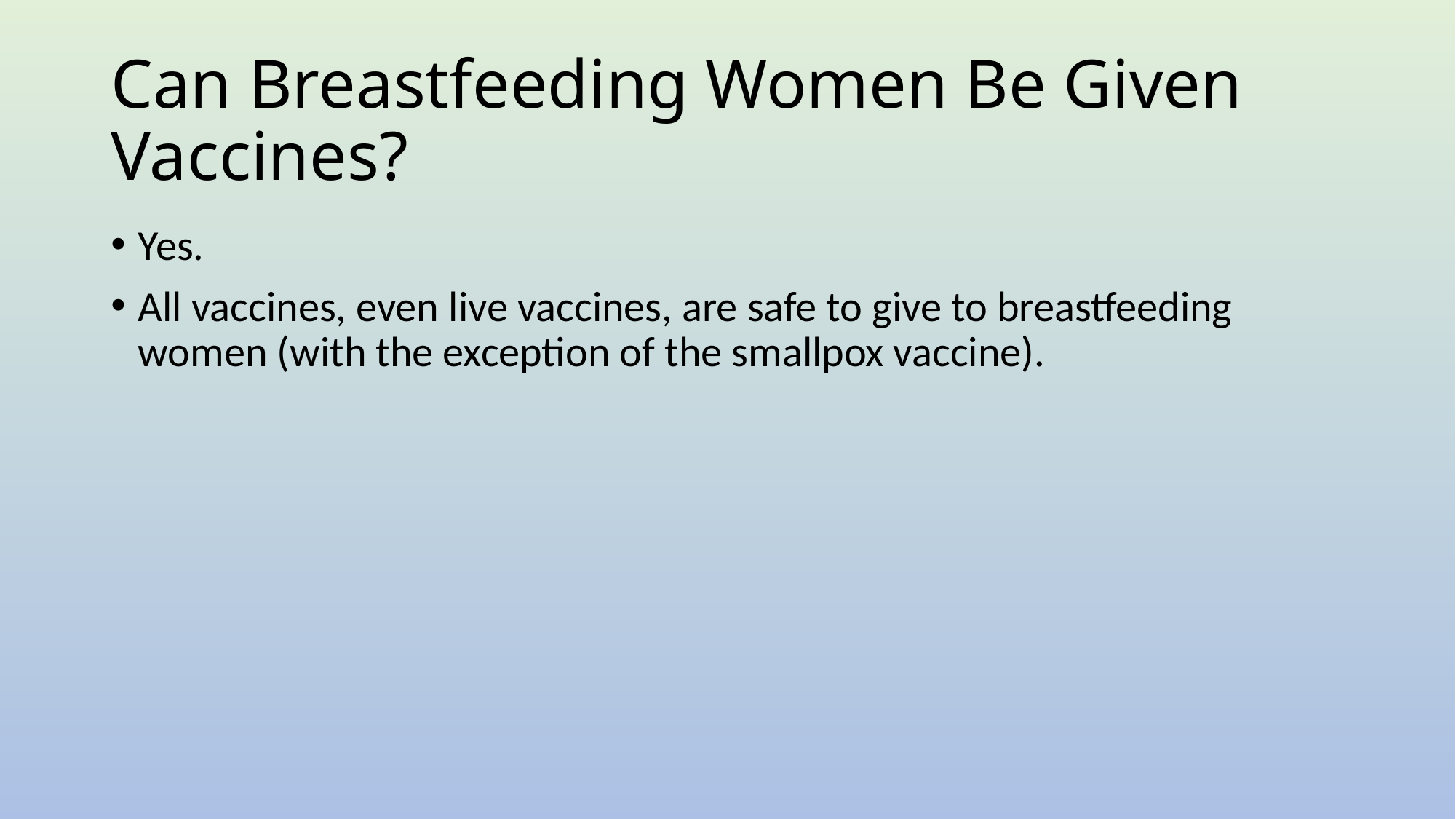

# Can Breastfeeding Women Be Given Vaccines?
Yes.
All vaccines, even live vaccines, are safe to give to breastfeeding women (with the exception of the smallpox vaccine).

## Slide 18
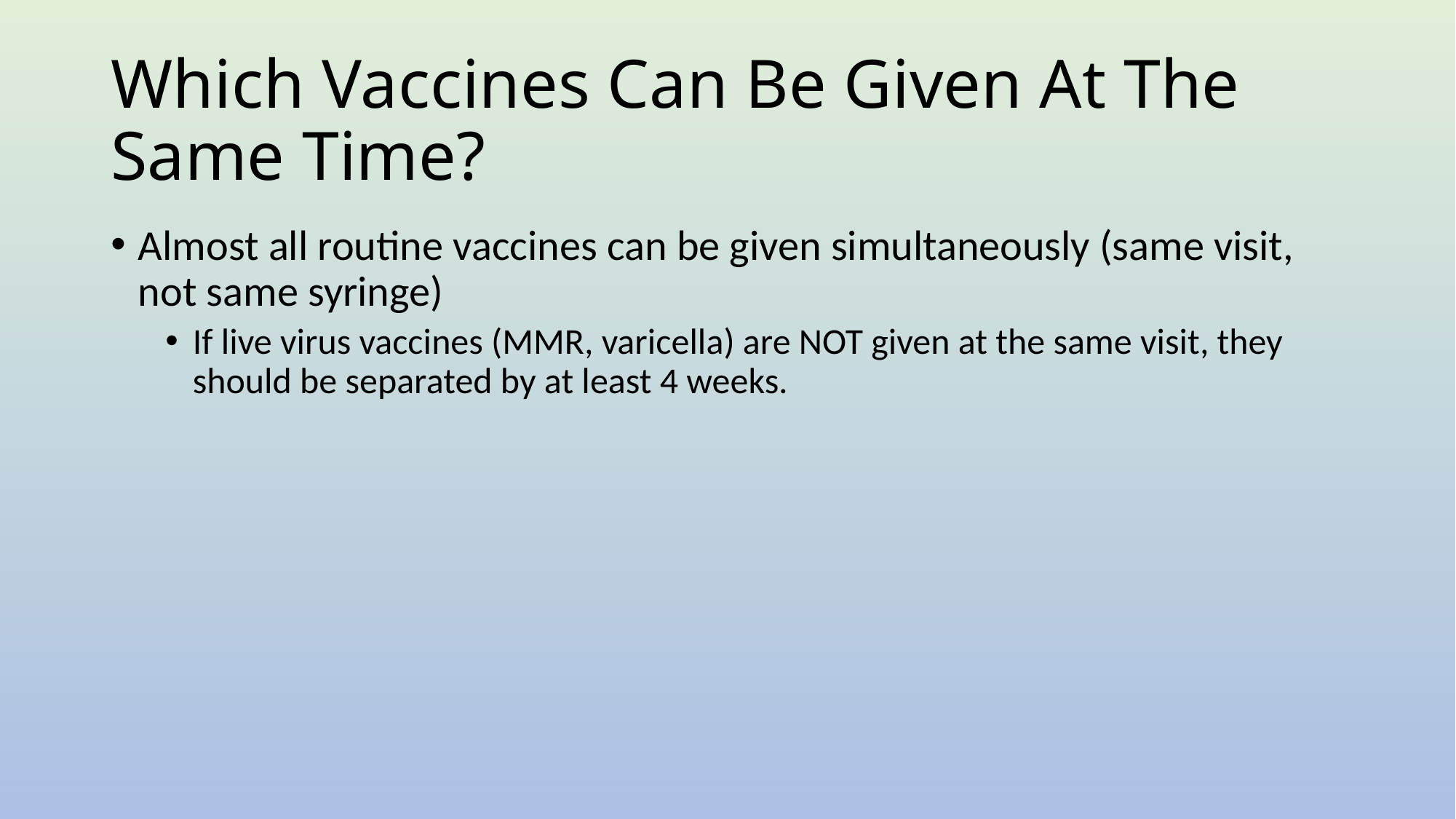

# Which Vaccines Can Be Given At The Same Time?
Almost all routine vaccines can be given simultaneously (same visit, not same syringe)
If live virus vaccines (MMR, varicella) are NOT given at the same visit, they should be separated by at least 4 weeks.

## Slide 19
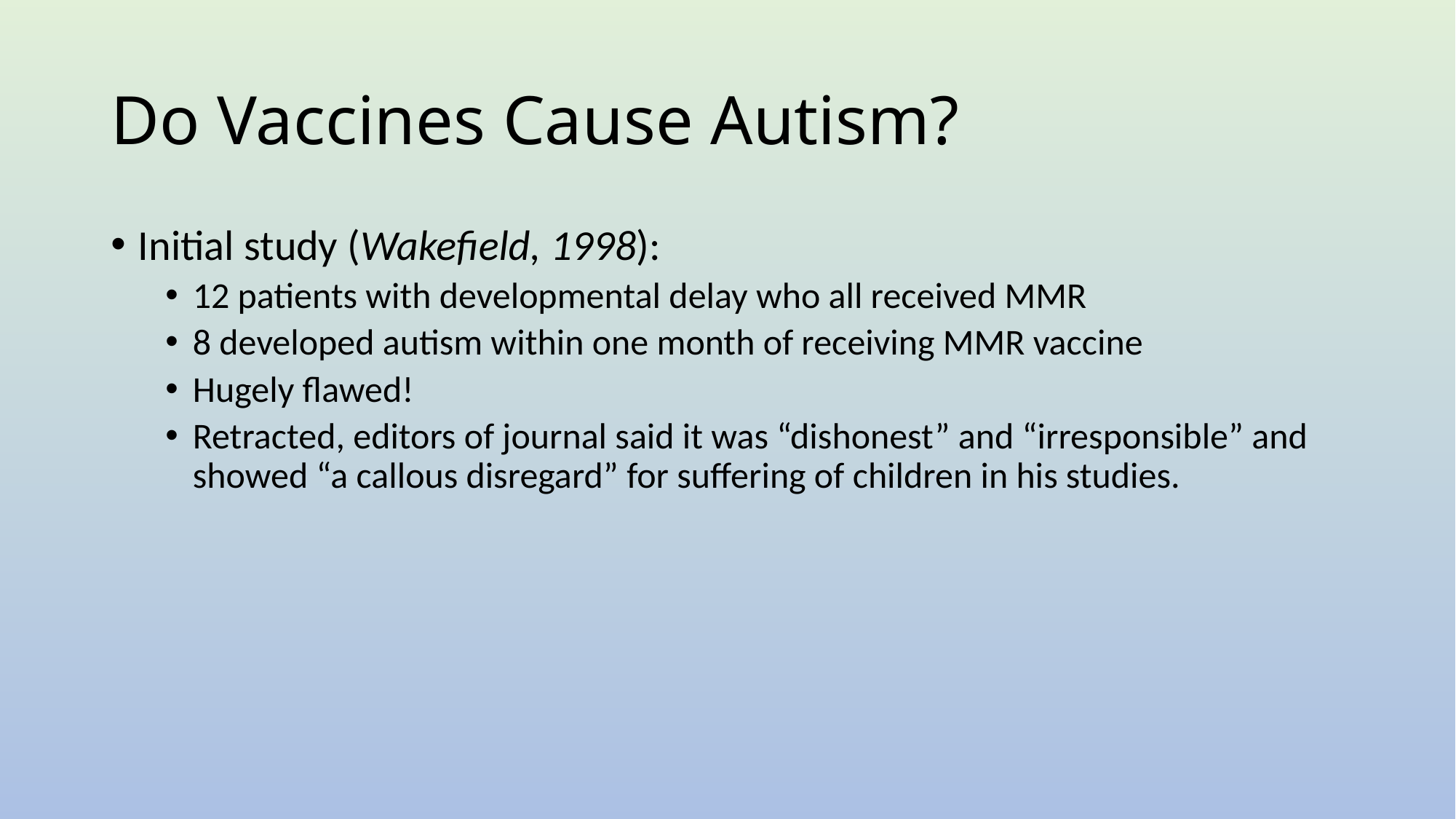

# Do Vaccines Cause Autism?
Initial study (Wakefield, 1998):
12 patients with developmental delay who all received MMR
8 developed autism within one month of receiving MMR vaccine
Hugely flawed!
Retracted, editors of journal said it was “dishonest” and “irresponsible” and showed “a callous disregard” for suffering of children in his studies.

## Slide 20
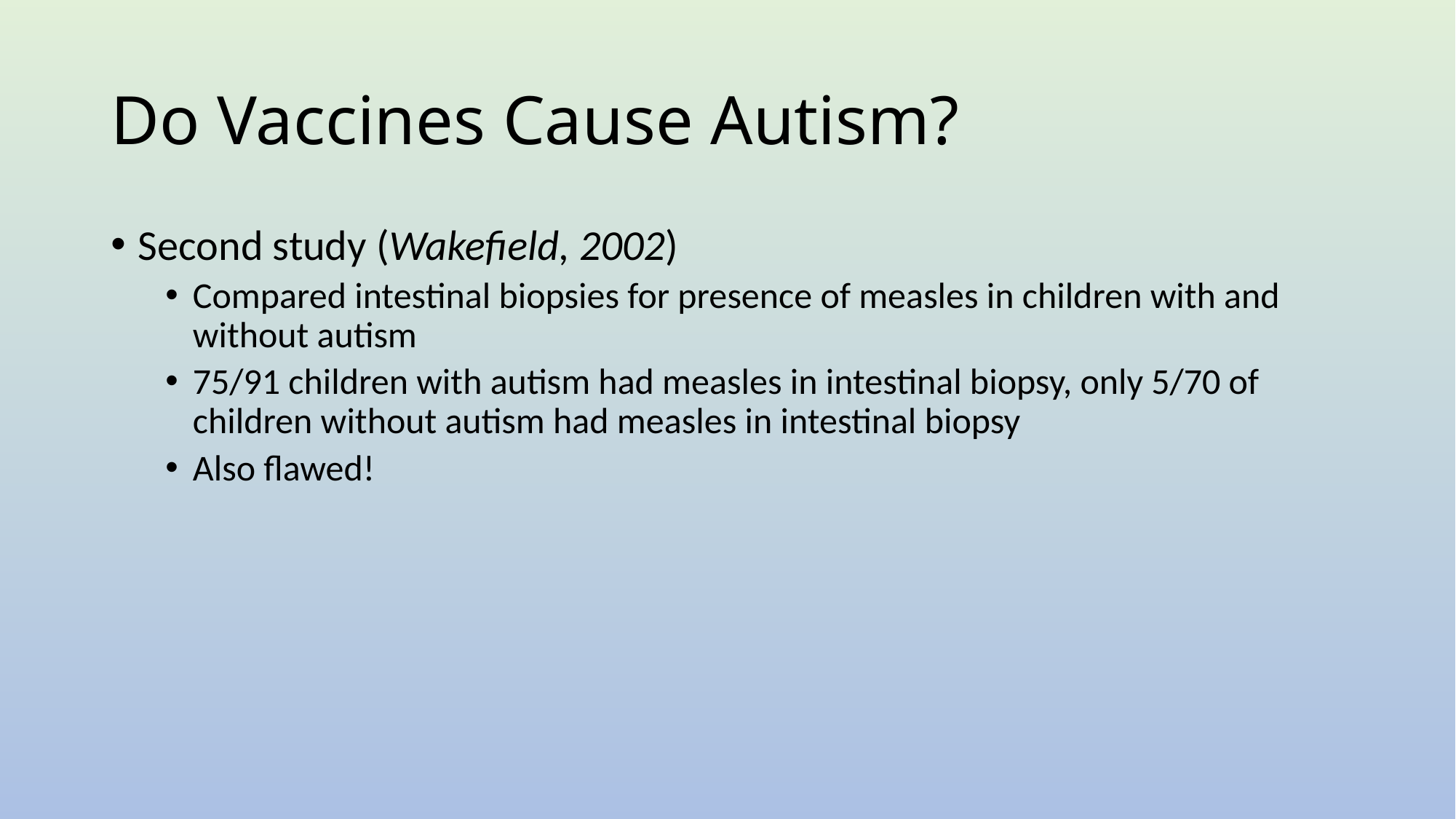

# Do Vaccines Cause Autism?
Second study (Wakefield, 2002)
Compared intestinal biopsies for presence of measles in children with and without autism
75/91 children with autism had measles in intestinal biopsy, only 5/70 of children without autism had measles in intestinal biopsy
Also flawed!

## Slide 21
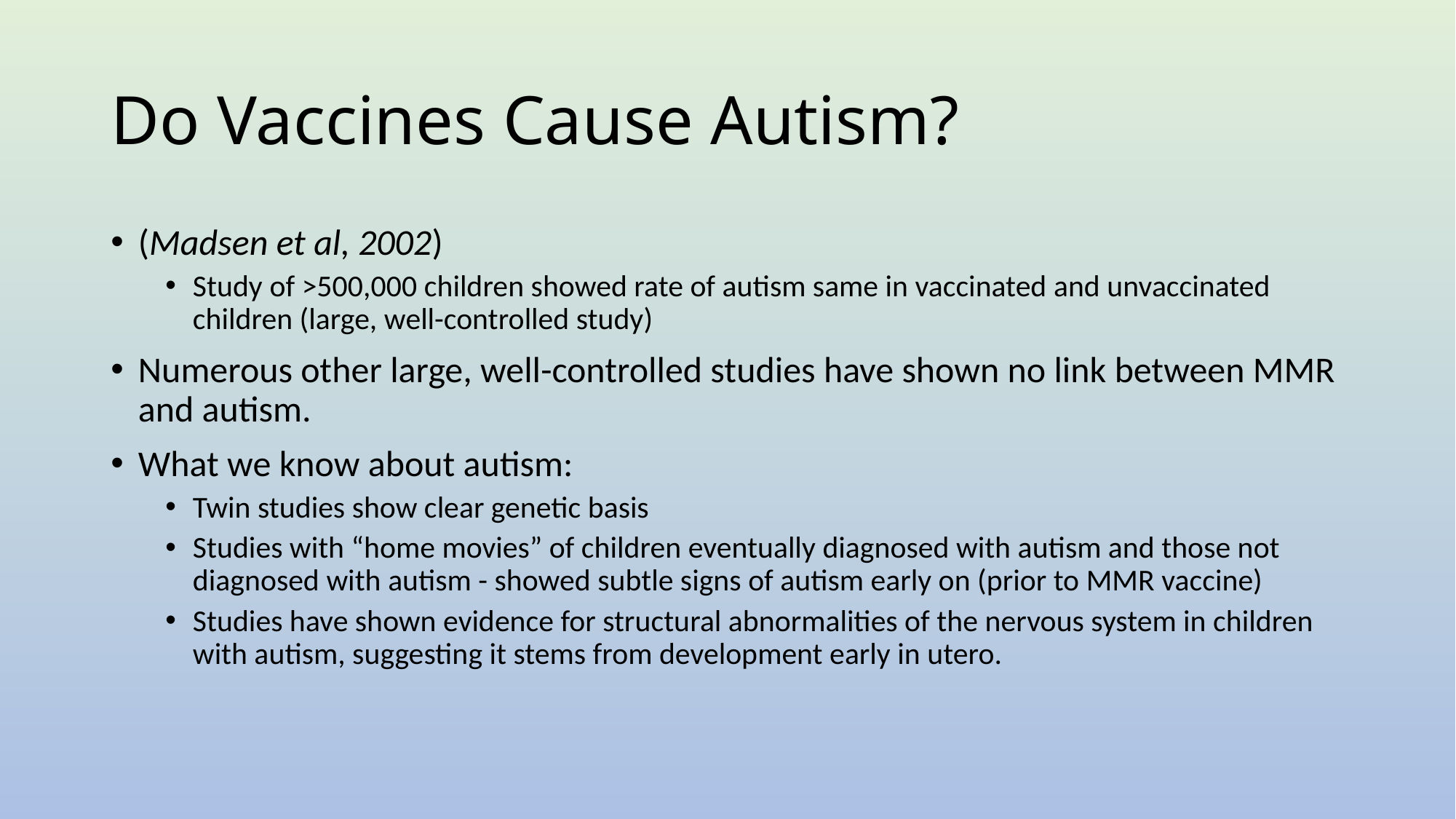

# Do Vaccines Cause Autism?
(Madsen et al, 2002)
Study of >500,000 children showed rate of autism same in vaccinated and unvaccinated children (large, well-controlled study)
Numerous other large, well-controlled studies have shown no link between MMR and autism.
What we know about autism:
Twin studies show clear genetic basis
Studies with “home movies” of children eventually diagnosed with autism and those not diagnosed with autism - showed subtle signs of autism early on (prior to MMR vaccine)
Studies have shown evidence for structural abnormalities of the nervous system in children with autism, suggesting it stems from development early in utero.

## Slide 22
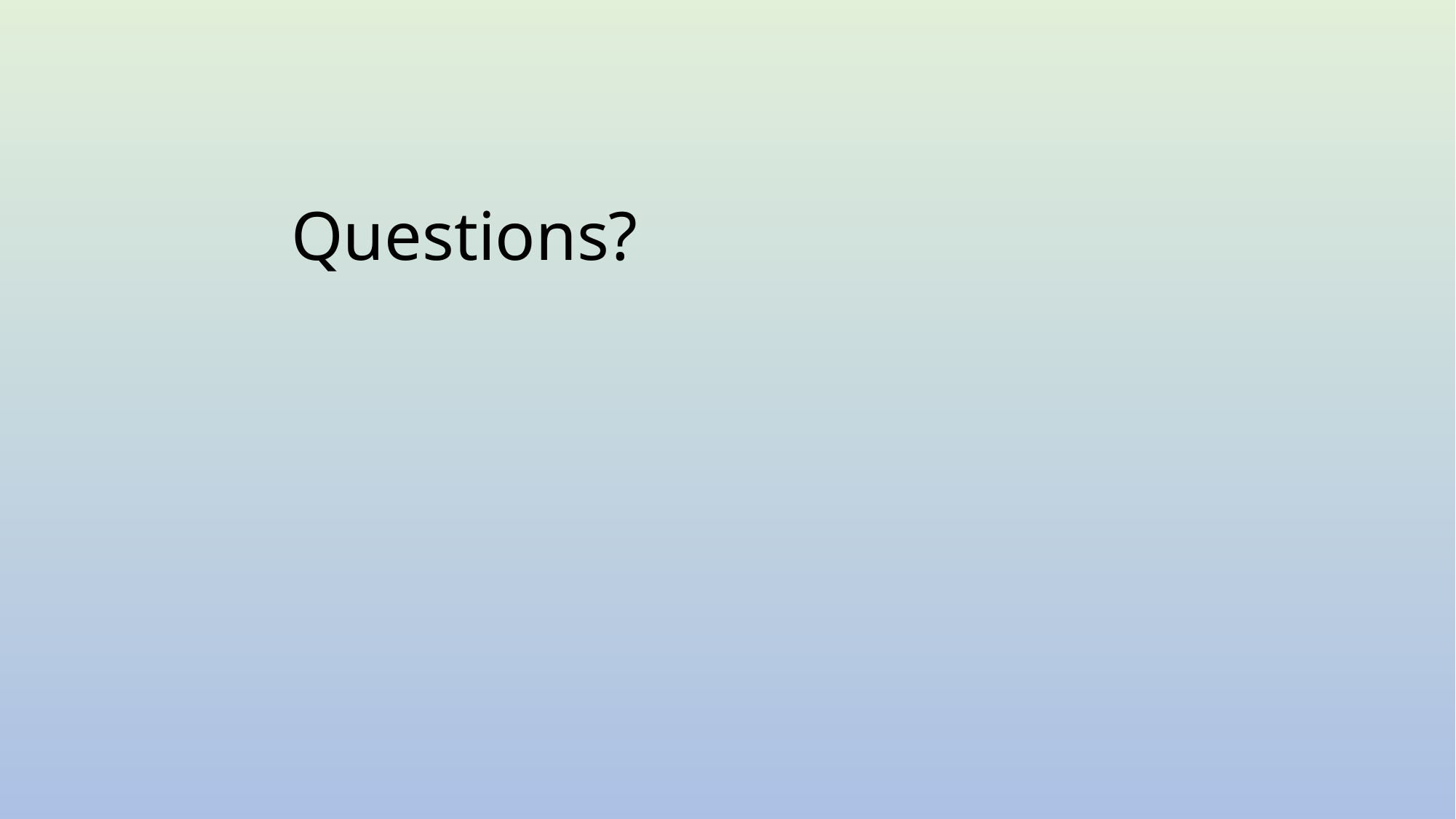

Questions?

## Slide 23
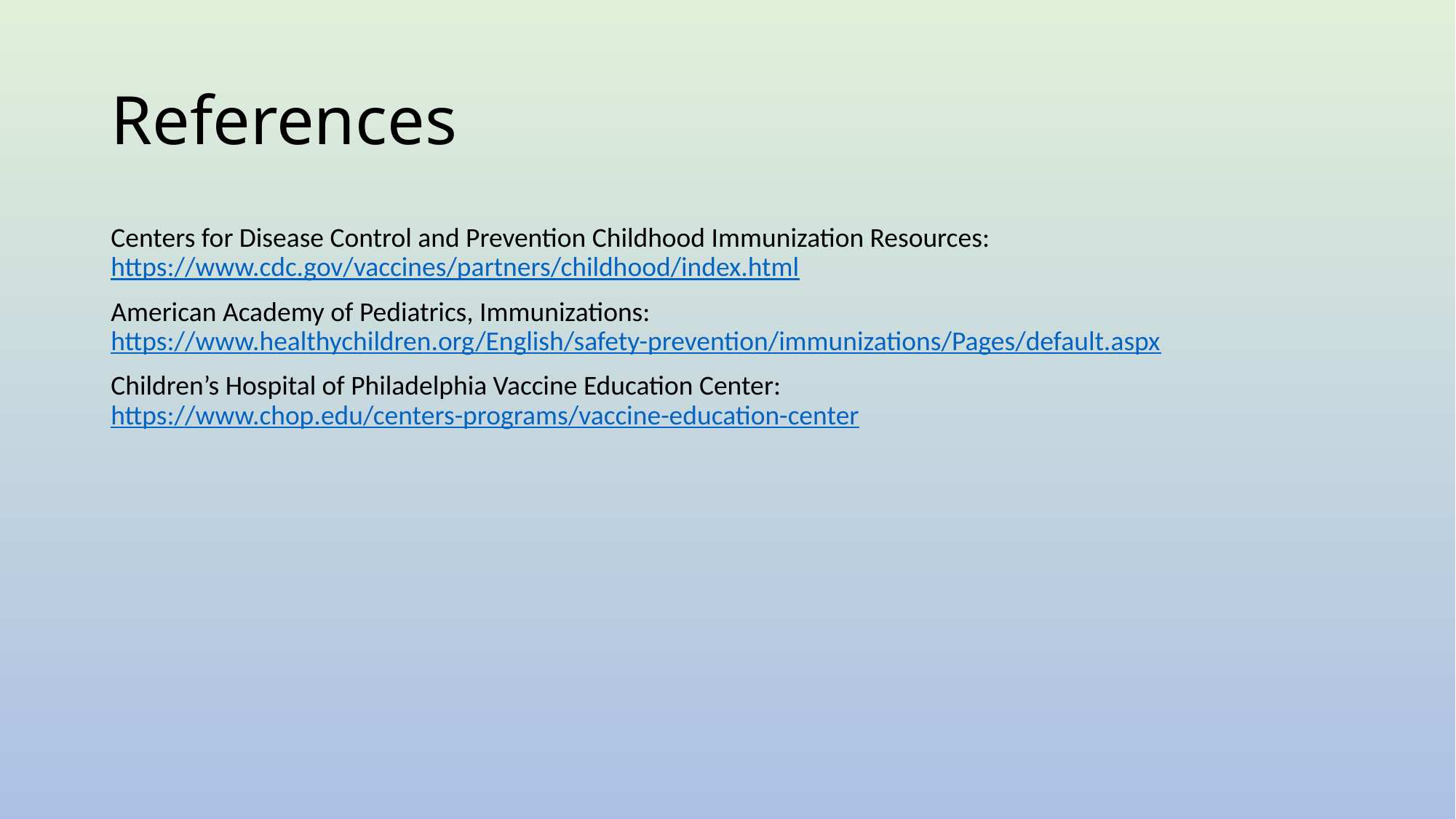

# References
Centers for Disease Control and Prevention Childhood Immunization Resources: https://www.cdc.gov/vaccines/partners/childhood/index.html
American Academy of Pediatrics, Immunizations: https://www.healthychildren.org/English/safety-prevention/immunizations/Pages/default.aspx
Children’s Hospital of Philadelphia Vaccine Education Center: https://www.chop.edu/centers-programs/vaccine-education-center
