# Supplementary material for: Addressing Vaccine Hesitancy Through a Comprehensive Resident Vaccine Curriculum
Source: MedEdPORTAL. 2022 Dec 27;18:11292. doi: 10.15766/mep_2374-8265.11292 (PMC9792628; doi:10.15766/mep_2374-8265.11292)
Supplement: Supplementary file 1 — Vaccine Curriculum Facilitator Guide.docxVaccines Part 1.pptxVaccines Part 2.pptxVaccines Part 3 - Myths and Facts.pptxVaccines Part 4 - Communication Skills.pptxVaccine Hesitancy Communication Cases.docxVaccine Pretest.docxVaccine Posttest.docxPre- and Posttest Answer Key.docxSP Case and Notes for SP.docxSP Case Development Tool.docxSP Case - Learner Version.docxSP Assessment Checklist.docx [file mep_2374-8265.11292-s001.zip › E. Vaccines Part 4 - Communication Skills.pptx]

## Slide 1
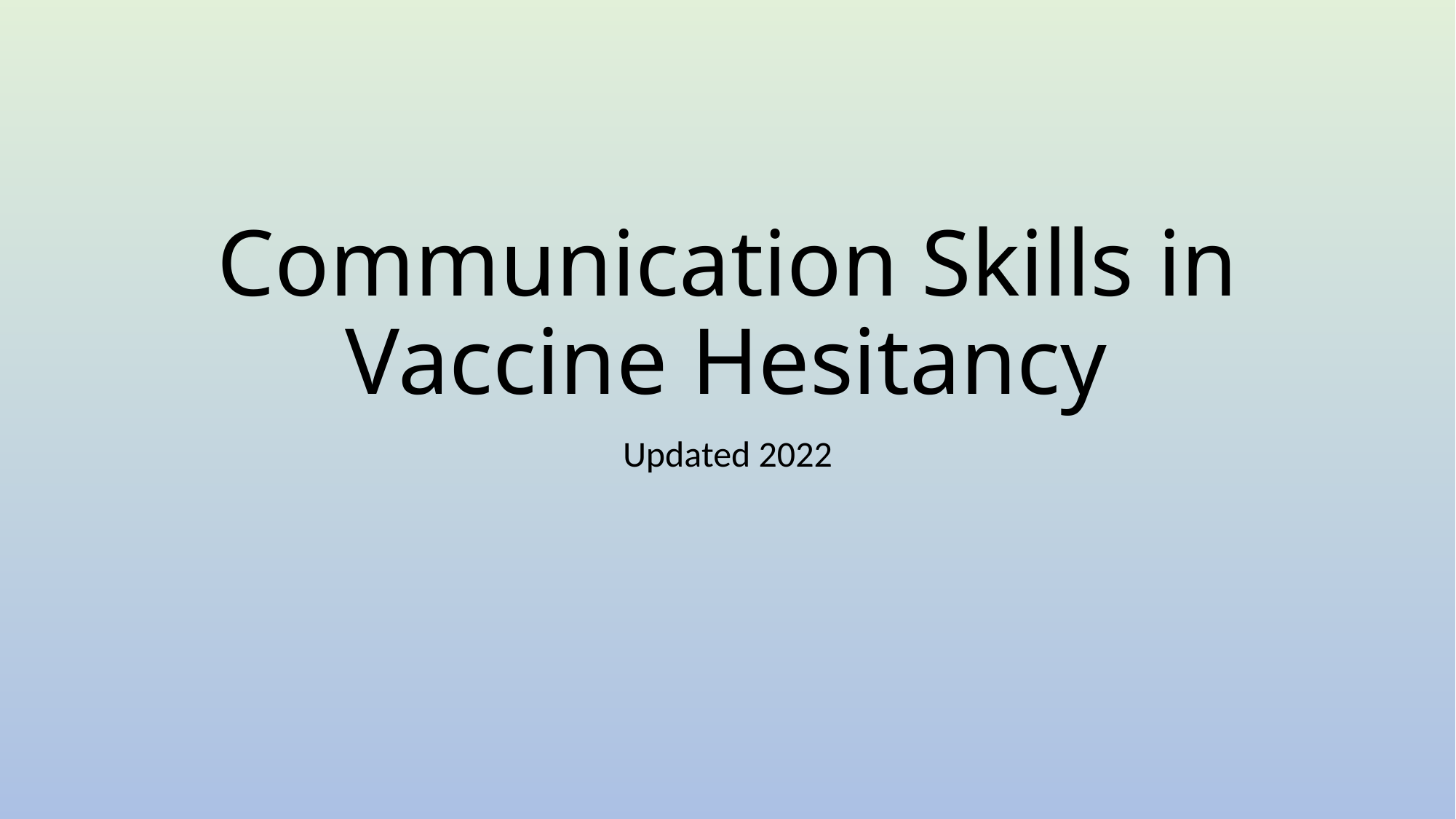

# Communication Skills in Vaccine Hesitancy
Updated 2022

## Slide 2
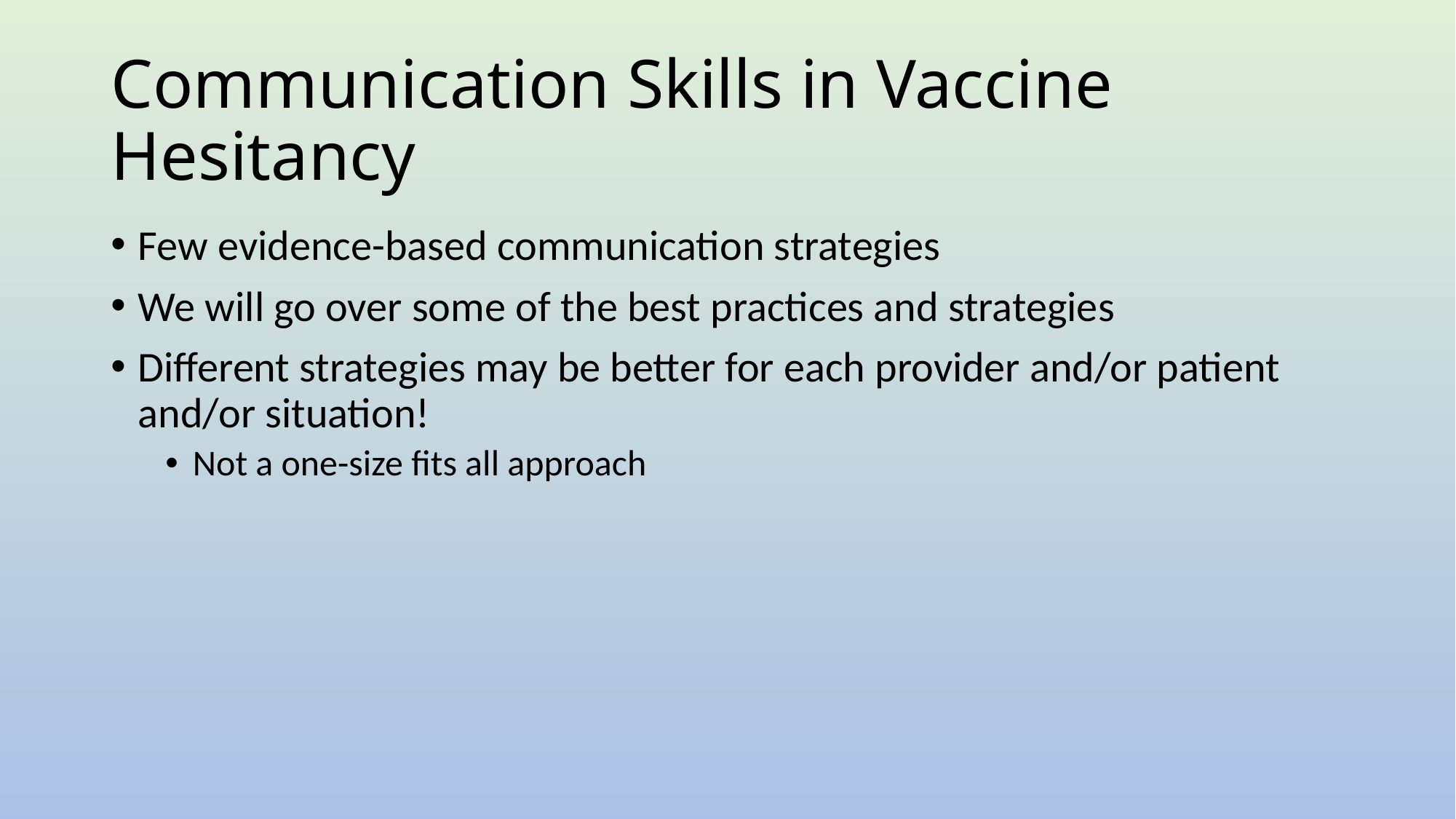

# Communication Skills in Vaccine Hesitancy
Few evidence-based communication strategies
We will go over some of the best practices and strategies
Different strategies may be better for each provider and/or patient and/or situation!
Not a one-size fits all approach

## Slide 3
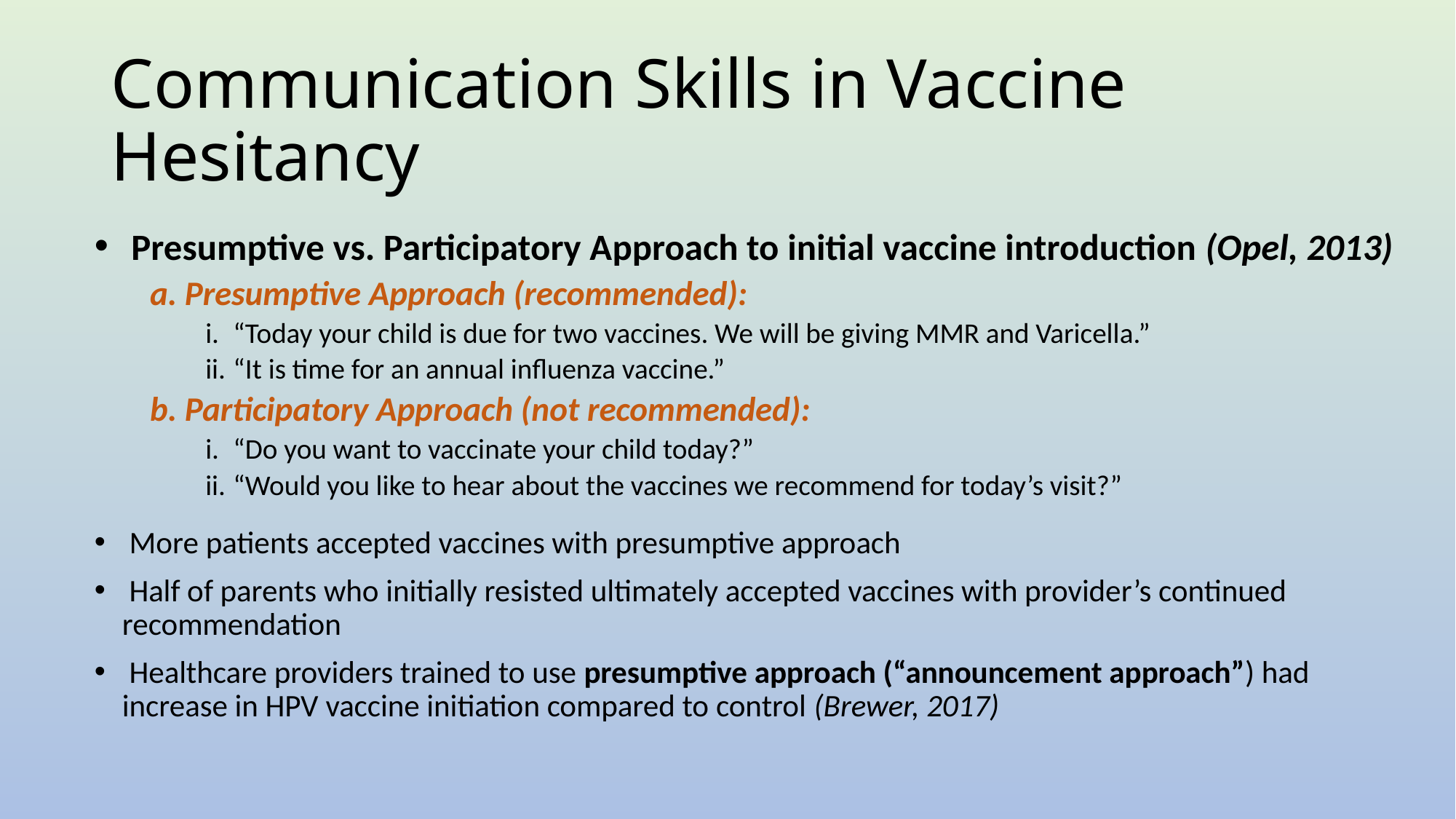

# Communication Skills in Vaccine Hesitancy
 Presumptive vs. Participatory Approach to initial vaccine introduction (Opel, 2013)
Presumptive Approach (recommended):
“Today your child is due for two vaccines. We will be giving MMR and Varicella.”
“It is time for an annual influenza vaccine.”
Participatory Approach (not recommended):
“Do you want to vaccinate your child today?”
“Would you like to hear about the vaccines we recommend for today’s visit?”
 More patients accepted vaccines with presumptive approach
 Half of parents who initially resisted ultimately accepted vaccines with provider’s continued recommendation
 Healthcare providers trained to use presumptive approach (“announcement approach”) had increase in HPV vaccine initiation compared to control (Brewer, 2017)

## Slide 4
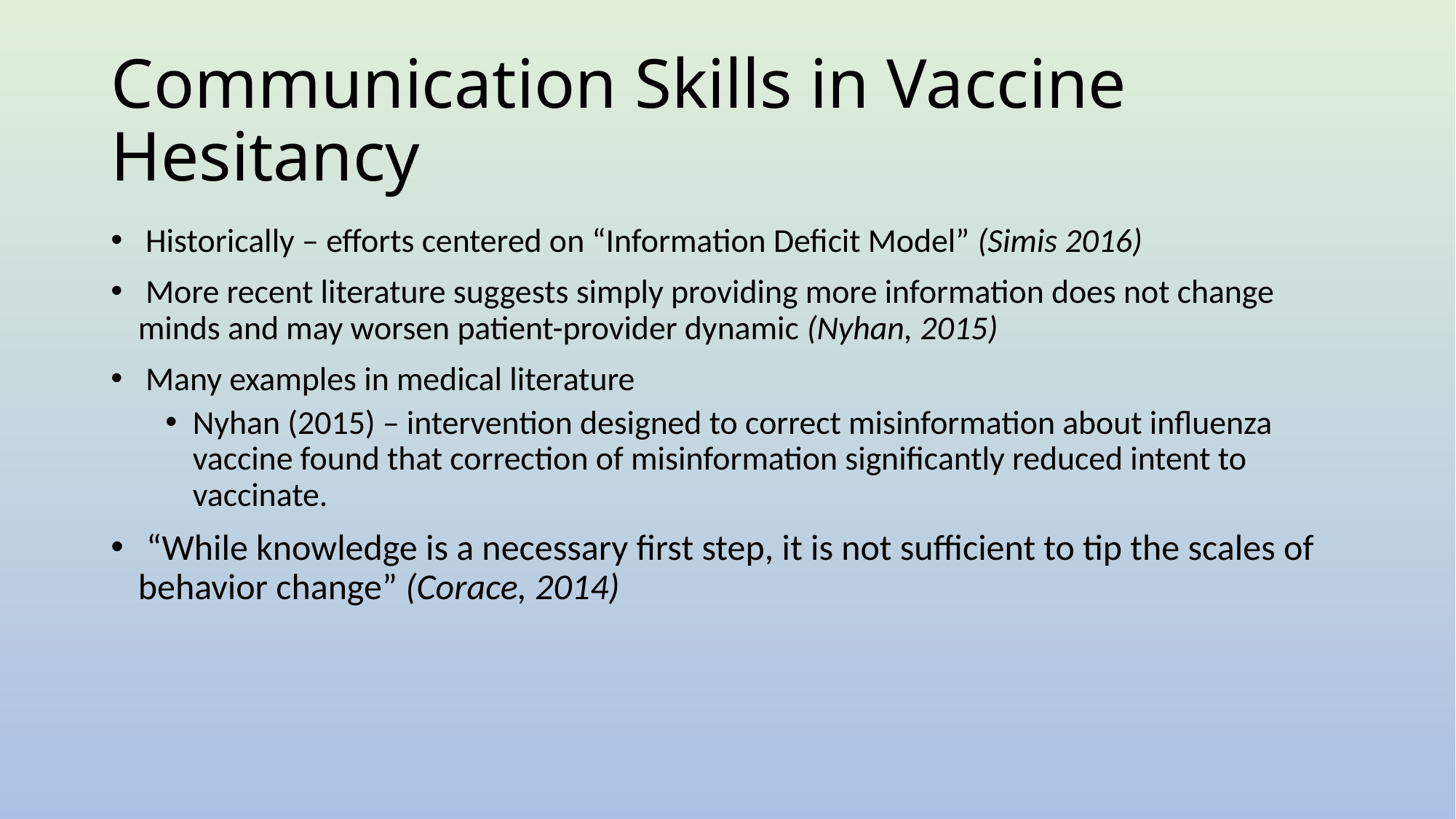

# Communication Skills in Vaccine Hesitancy
 Historically – efforts centered on “Information Deficit Model” (Simis 2016)
 More recent literature suggests simply providing more information does not change minds and may worsen patient-provider dynamic (Nyhan, 2015)
 Many examples in medical literature
Nyhan (2015) – intervention designed to correct misinformation about influenza vaccine found that correction of misinformation significantly reduced intent to vaccinate.
 “While knowledge is a necessary first step, it is not sufficient to tip the scales of behavior change” (Corace, 2014)

## Slide 5
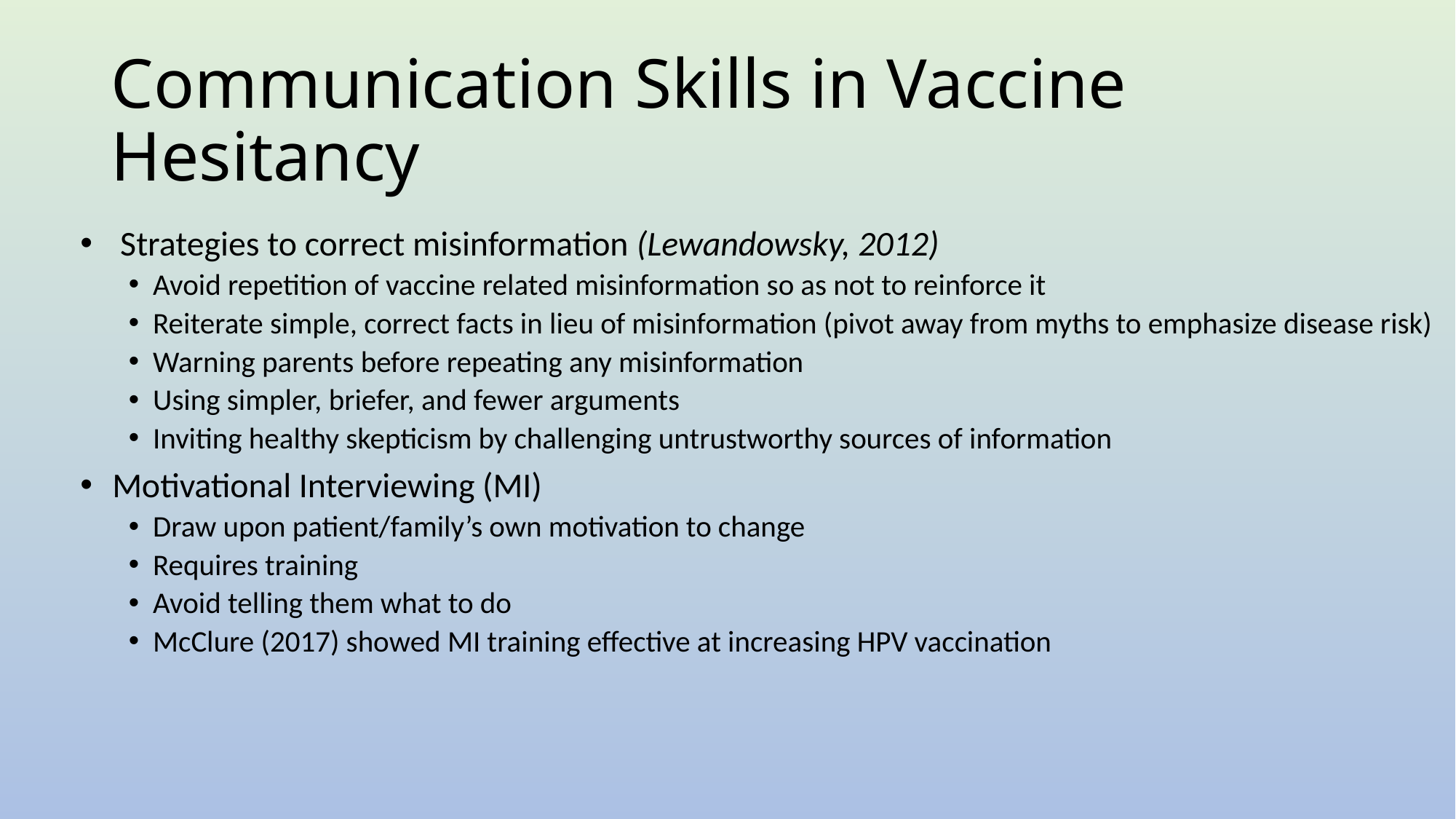

# Communication Skills in Vaccine Hesitancy
 Strategies to correct misinformation (Lewandowsky, 2012)
Avoid repetition of vaccine related misinformation so as not to reinforce it
Reiterate simple, correct facts in lieu of misinformation (pivot away from myths to emphasize disease risk)
Warning parents before repeating any misinformation
Using simpler, briefer, and fewer arguments
Inviting healthy skepticism by challenging untrustworthy sources of information
 Motivational Interviewing (MI)
Draw upon patient/family’s own motivation to change
Requires training
Avoid telling them what to do
McClure (2017) showed MI training effective at increasing HPV vaccination

## Slide 6
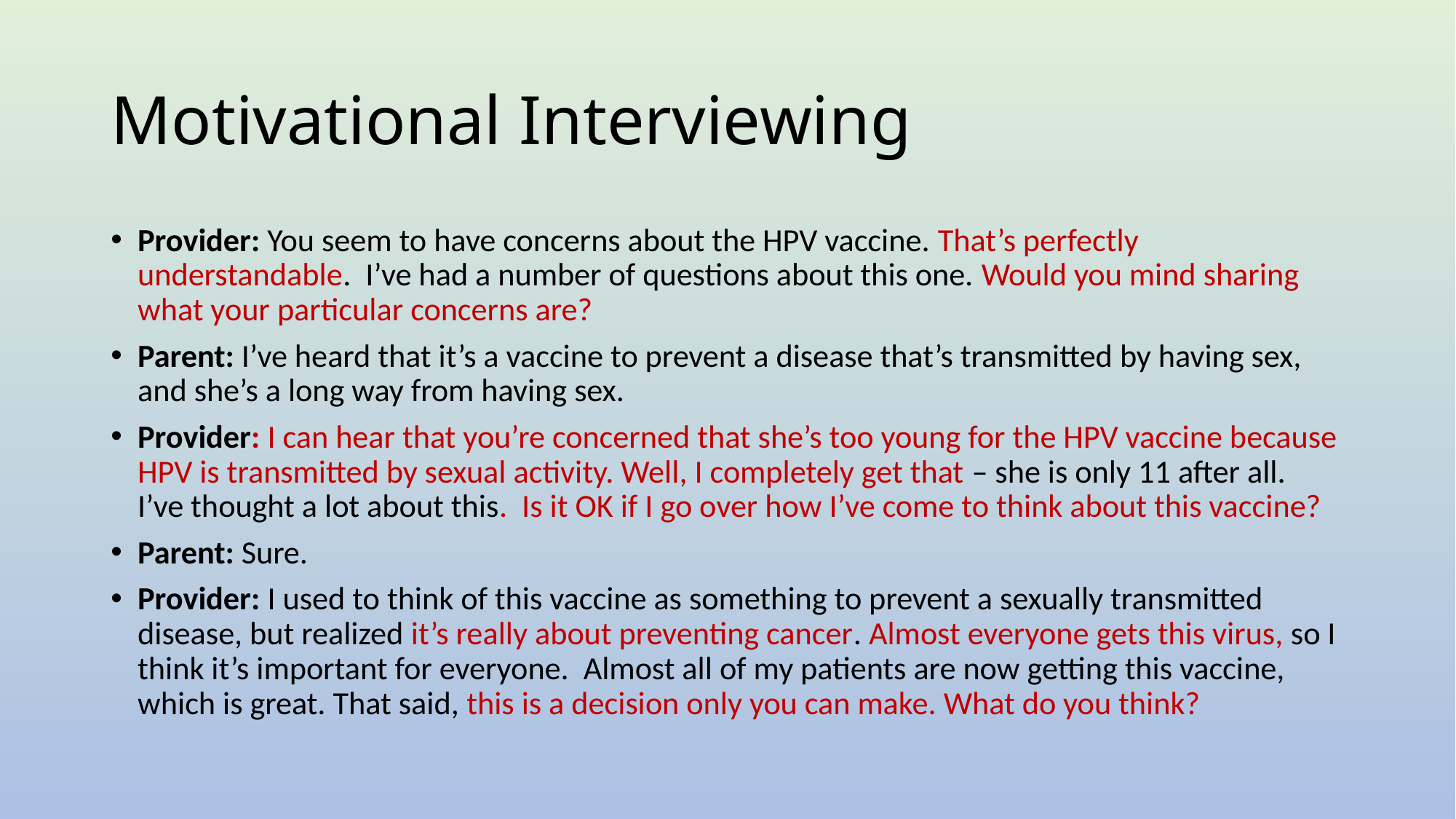

# Motivational Interviewing
Provider: You seem to have concerns about the HPV vaccine. That’s perfectly understandable. I’ve had a number of questions about this one. Would you mind sharing what your particular concerns are?
Parent: I’ve heard that it’s a vaccine to prevent a disease that’s transmitted by having sex, and she’s a long way from having sex.
Provider: I can hear that you’re concerned that she’s too young for the HPV vaccine because HPV is transmitted by sexual activity. Well, I completely get that – she is only 11 after all. I’ve thought a lot about this. Is it OK if I go over how I’ve come to think about this vaccine?
Parent: Sure.
Provider: I used to think of this vaccine as something to prevent a sexually transmitted disease, but realized it’s really about preventing cancer. Almost everyone gets this virus, so I think it’s important for everyone. Almost all of my patients are now getting this vaccine, which is great. That said, this is a decision only you can make. What do you think?

## Slide 7
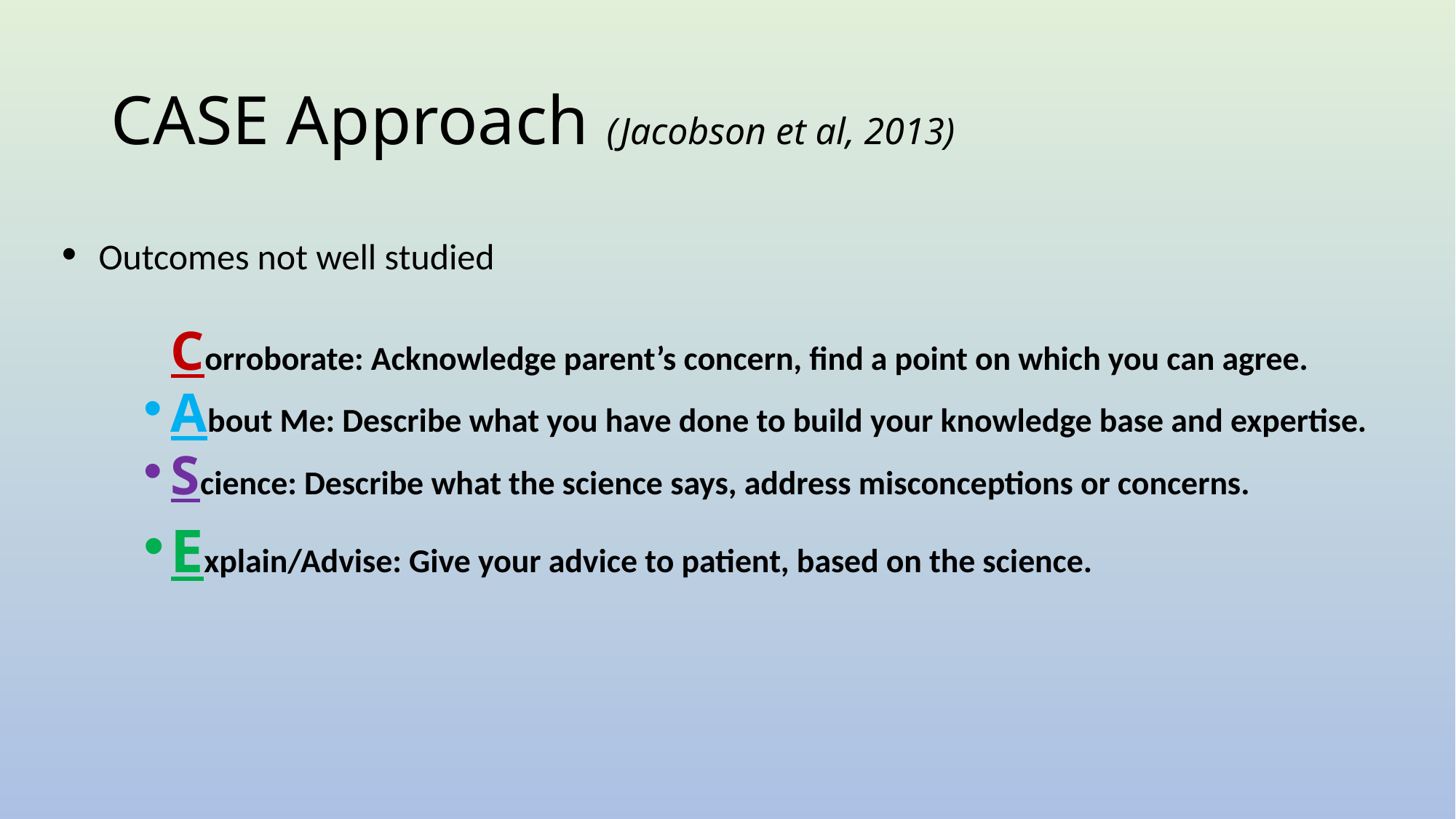

# CASE Approach (Jacobson et al, 2013)
 Outcomes not well studied
	Corroborate: Acknowledge parent’s concern, find a point on which you can agree.
About Me: Describe what you have done to build your knowledge base and expertise.
Science: Describe what the science says, address misconceptions or concerns.
Explain/Advise: Give your advice to patient, based on the science.

## Slide 8
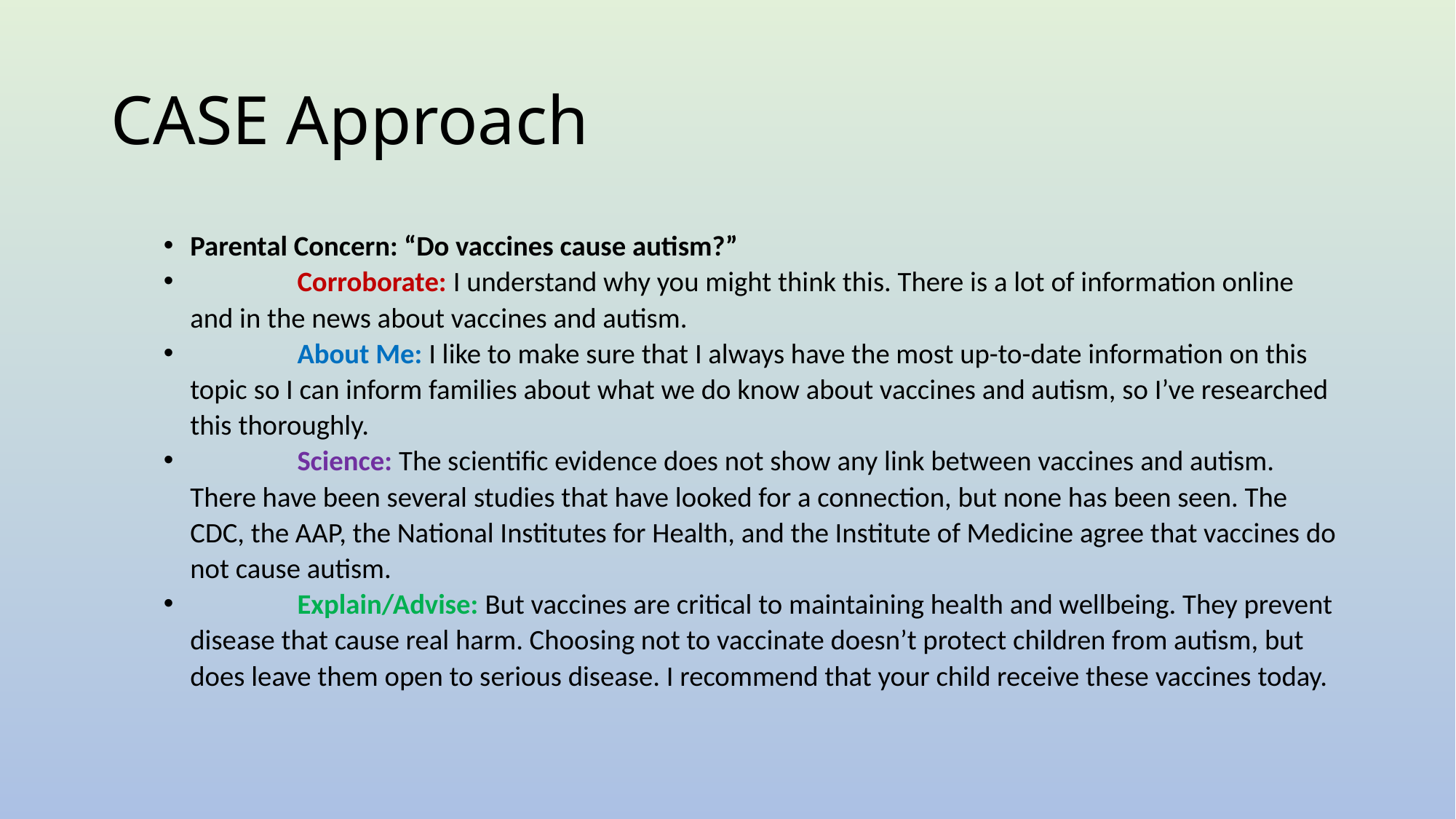

# CASE Approach
Parental Concern: “Do vaccines cause autism?”
	Corroborate: I understand why you might think this. There is a lot of information online and in the news about vaccines and autism.
	About Me: I like to make sure that I always have the most up-to-date information on this topic so I can inform families about what we do know about vaccines and autism, so I’ve researched this thoroughly.
	Science: The scientific evidence does not show any link between vaccines and autism. There have been several studies that have looked for a connection, but none has been seen. The CDC, the AAP, the National Institutes for Health, and the Institute of Medicine agree that vaccines do not cause autism.
	Explain/Advise: But vaccines are critical to maintaining health and wellbeing. They prevent disease that cause real harm. Choosing not to vaccinate doesn’t protect children from autism, but does leave them open to serious disease. I recommend that your child receive these vaccines today.

## Slide 9
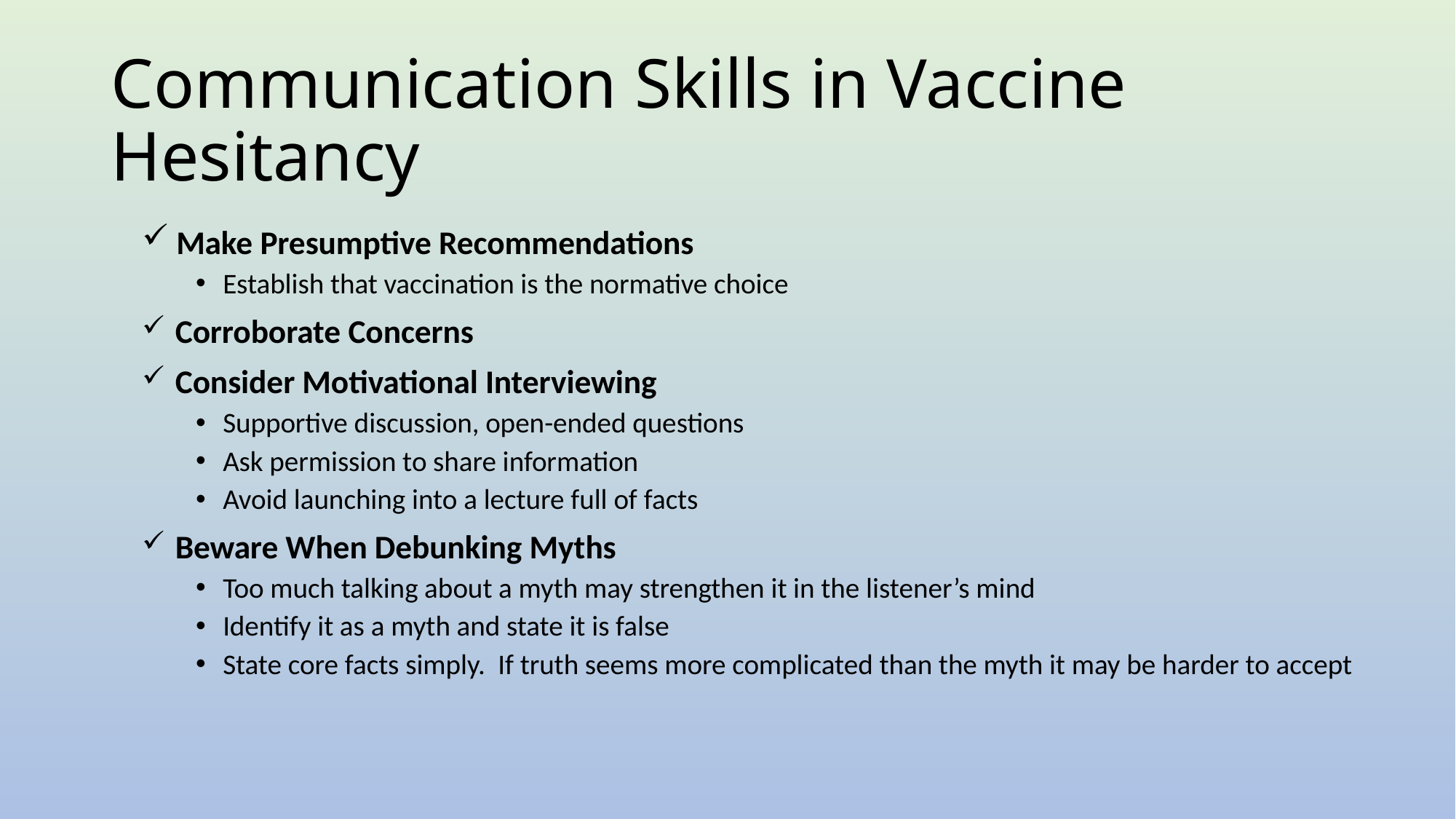

# Communication Skills in Vaccine Hesitancy
 Make Presumptive Recommendations
Establish that vaccination is the normative choice
 Corroborate Concerns
 Consider Motivational Interviewing
Supportive discussion, open-ended questions
Ask permission to share information
Avoid launching into a lecture full of facts
 Beware When Debunking Myths
Too much talking about a myth may strengthen it in the listener’s mind
Identify it as a myth and state it is false
State core facts simply. If truth seems more complicated than the myth it may be harder to accept

## Slide 10
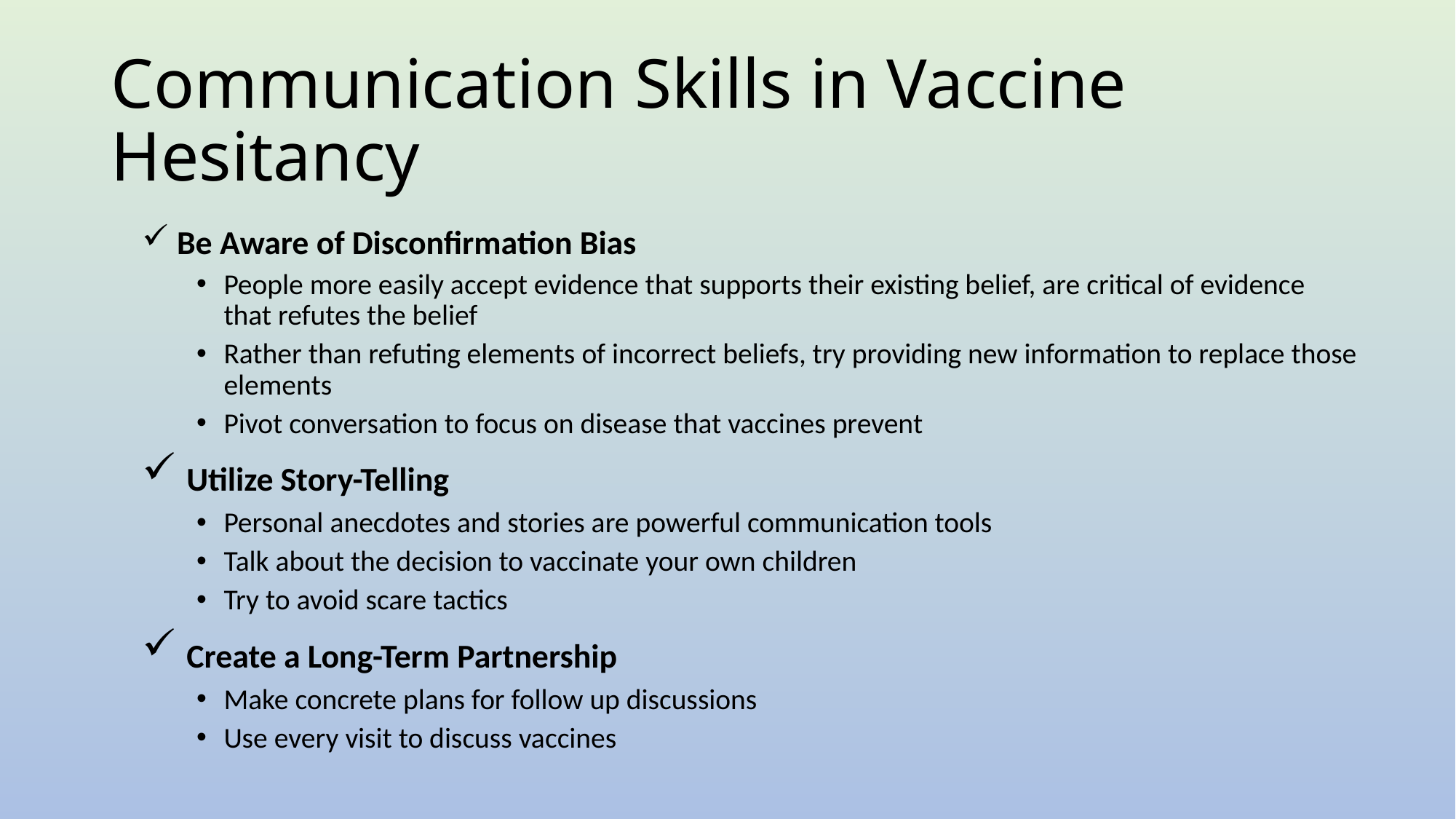

# Communication Skills in Vaccine Hesitancy
 Be Aware of Disconfirmation Bias
People more easily accept evidence that supports their existing belief, are critical of evidence that refutes the belief
Rather than refuting elements of incorrect beliefs, try providing new information to replace those elements
Pivot conversation to focus on disease that vaccines prevent
 Utilize Story-Telling
Personal anecdotes and stories are powerful communication tools
Talk about the decision to vaccinate your own children
Try to avoid scare tactics
 Create a Long-Term Partnership
Make concrete plans for follow up discussions
Use every visit to discuss vaccines

## Slide 11
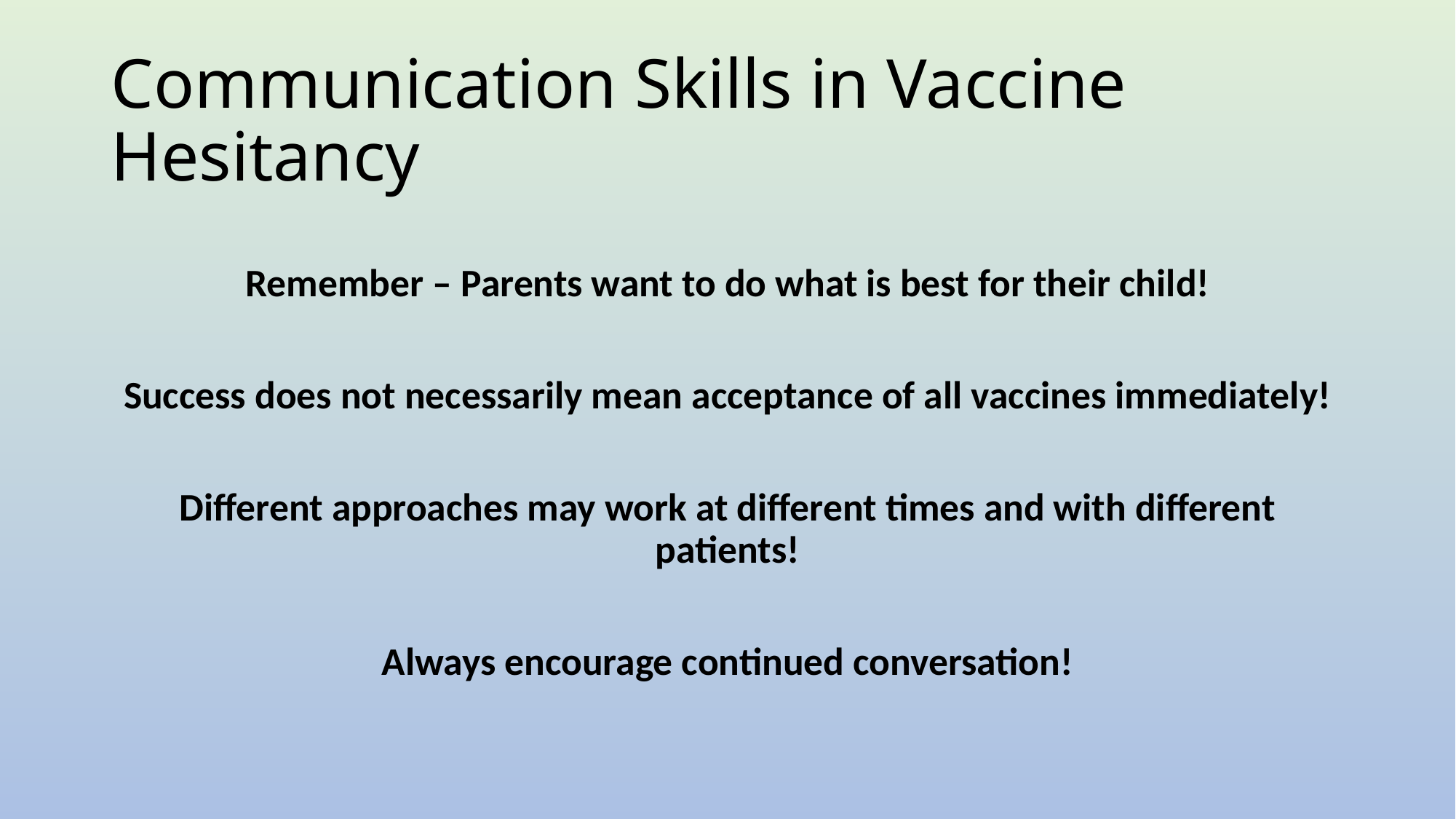

# Communication Skills in Vaccine Hesitancy
Remember – Parents want to do what is best for their child!
Success does not necessarily mean acceptance of all vaccines immediately!
Different approaches may work at different times and with different patients!
Always encourage continued conversation!

## Slide 12
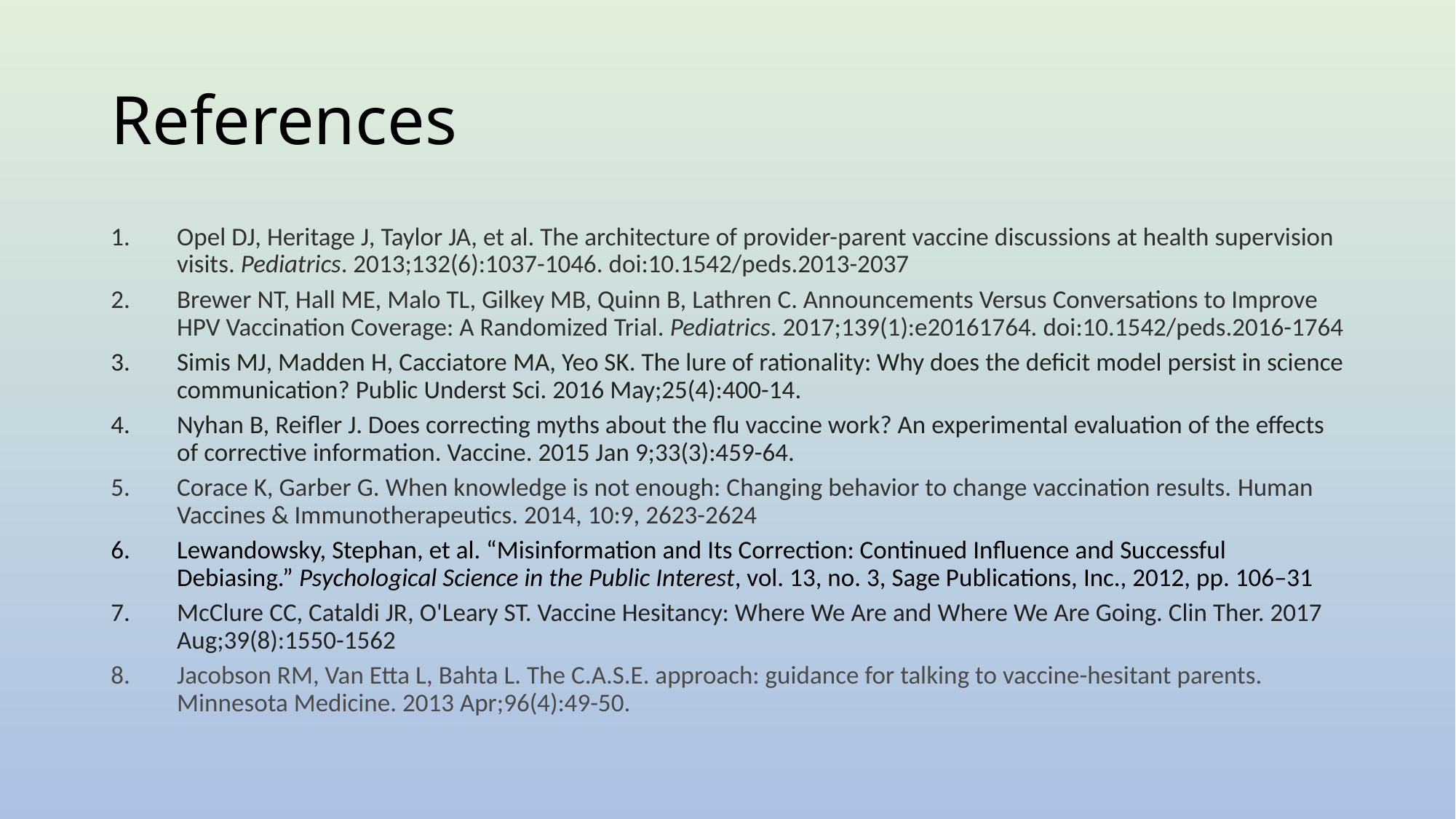

# References
Opel DJ, Heritage J, Taylor JA, et al. The architecture of provider-parent vaccine discussions at health supervision visits. Pediatrics. 2013;132(6):1037-1046. doi:10.1542/peds.2013-2037
Brewer NT, Hall ME, Malo TL, Gilkey MB, Quinn B, Lathren C. Announcements Versus Conversations to Improve HPV Vaccination Coverage: A Randomized Trial. Pediatrics. 2017;139(1):e20161764. doi:10.1542/peds.2016-1764
Simis MJ, Madden H, Cacciatore MA, Yeo SK. The lure of rationality: Why does the deficit model persist in science communication? Public Underst Sci. 2016 May;25(4):400-14.
Nyhan B, Reifler J. Does correcting myths about the flu vaccine work? An experimental evaluation of the effects of corrective information. Vaccine. 2015 Jan 9;33(3):459-64.
Corace K, Garber G. When knowledge is not enough: Changing behavior to change vaccination results. Human Vaccines & Immunotherapeutics. 2014, 10:9, 2623-2624
Lewandowsky, Stephan, et al. “Misinformation and Its Correction: Continued Influence and Successful Debiasing.” Psychological Science in the Public Interest, vol. 13, no. 3, Sage Publications, Inc., 2012, pp. 106–31
McClure CC, Cataldi JR, O'Leary ST. Vaccine Hesitancy: Where We Are and Where We Are Going. Clin Ther. 2017 Aug;39(8):1550-1562
Jacobson RM, Van Etta L, Bahta L. The C.A.S.E. approach: guidance for talking to vaccine-hesitant parents. Minnesota Medicine. 2013 Apr;96(4):49-50.
