# Supplementary material for: Addressing Vaccine Hesitancy Through a Comprehensive Resident Vaccine Curriculum
Source: MedEdPORTAL. 2022 Dec 27;18:11292. doi: 10.15766/mep_2374-8265.11292 (PMC9792628; doi:10.15766/mep_2374-8265.11292)
Supplement: Supplementary file 1 — Vaccine Curriculum Facilitator Guide.docxVaccines Part 1.pptxVaccines Part 2.pptxVaccines Part 3 - Myths and Facts.pptxVaccines Part 4 - Communication Skills.pptxVaccine Hesitancy Communication Cases.docxVaccine Pretest.docxVaccine Posttest.docxPre- and Posttest Answer Key.docxSP Case and Notes for SP.docxSP Case Development Tool.docxSP Case - Learner Version.docxSP Assessment Checklist.docx [file mep_2374-8265.11292-s001.zip › F. Vaccine Hesitancy Communication Cases.docx]

**Counseling Vaccine Hesitant Families**

Cases

Case 1:

You are the parent of Samuel, a healthy 11-year-old male. You brought Samuel to the clinic for his annual sports physical prior to the start of basketball season. You are surprised to learn that Samuel is due for the HPV vaccine. You had heard that this vaccine was intended only for sexually active people and Samuel is only 11 years old. You had also heard that it was a vaccine for women. You are confused by this recommendation and hesitant to have Samuel receive the immunization without learning more and speaking to Samuel’s other parent.

Case 2:

You are the parent of Kayla, a 2-year-old girl with a history of moderate persistent asthma. You brought Kayla to the clinic today for evaluation of a rash. The physician says the rash is due to something called contact dermatitis, likely related to wearing your jewelry during play. The physician recommends that Kayla receive the influenza vaccine today. You are hesitant. You have never given Kayla the influenza vaccine and you have heard that it can cause the flu. You are also worried that you should not give her the vaccine when she is “sick.”

Case 3:

You are the parent of Jack, a 6-hour old healthy boy. Jack was born at 36 weeks but is doing well and has remained with you since birth. The pediatrician has informed you that Jack will receive the hepatitis B vaccine today. You have not heard of a newborn receiving a vaccine before and you are worried about the possible adverse effects of giving a vaccine to such a young baby. You know that you do not have hepatitis and wonder why the infant must receive this immunization.
