# Supplementary material for: Addressing Vaccine Hesitancy Through a Comprehensive Resident Vaccine Curriculum
Source: MedEdPORTAL. 2022 Dec 27;18:11292. doi: 10.15766/mep_2374-8265.11292 (PMC9792628; doi:10.15766/mep_2374-8265.11292)
Supplement: Supplementary file 1 — Vaccine Curriculum Facilitator Guide.docxVaccines Part 1.pptxVaccines Part 2.pptxVaccines Part 3 - Myths and Facts.pptxVaccines Part 4 - Communication Skills.pptxVaccine Hesitancy Communication Cases.docxVaccine Pretest.docxVaccine Posttest.docxPre- and Posttest Answer Key.docxSP Case and Notes for SP.docxSP Case Development Tool.docxSP Case - Learner Version.docxSP Assessment Checklist.docx [file mep_2374-8265.11292-s001.zip › G. Vaccine Pretest.docx]

Vaccine Curriculum PRETEST

1. What is your current year of training?

___PL1 ___PL2 ___PL3

2. When and where do you have continuity clinic?

___ Mon PM ___ Tues AM ___ Tues PM ___ Weds AM

___ Weds PM ___ Thurs AM ___ Thurs PM ___ Fri AM

3. What is your OVERALL comfort level in discussing vaccines with vaccine-hesitant patients/families?

Not comfortable at all Slightly comfortable Moderately comfortable Mostly comfortable Completely comfortable

1 2 3 4 5

4. How comfortable do you feel with each of the following aspects of communicating with vaccine-hesitant patient/families?:

Discussing clinical features and risks of vaccine-preventable diseases:

Not comfortable at all Slightly comfortable Moderately comfortable Mostly comfortable Completely comfortable

1 2 3 4 5

Discussing potential risks and side effects of vaccines:

Not comfortable at all Slightly comfortable Moderately comfortable Mostly comfortable Completely comfortable

1 2 3 4 5

Creating a plan for continued discussion about vaccines at subsequent visits:

Not comfortable at all Slightly comfortable Moderately comfortable Mostly comfortable Completely comfortable

1 2 3 4 5

Discussing vaccine ingredients and their risks (*e.g., mercury content, aluminum content)*:

Not comfortable at all Slightly comfortable Moderately comfortable Mostly comfortable Completely comfortable

1 2 3 4 5

Discussing common vaccine misconceptions (*e.g., vaccines cause autism, natural immunity vs vaccine-induced immunity)*

Not comfortable at all Slightly comfortable Moderately comfortable Mostly comfortable Completely comfortable

1 2 3 4 5

Discussing indications and contraindications for vaccine administration (*e.g., immunocompromised patients, pregnant women)*

Not comfortable at all Slightly comfortable Moderately comfortable Mostly comfortable Completely comfortable

1 2 3 4 5

Using communication skills that have been found to be useful in discussions with vaccine-hesitant patient/families:

Not comfortable at all Slightly comfortable Moderately comfortable Mostly comfortable Completely comfortable

1 2 3 4 5

5. Which communication technique has been shown to be effective in improving vaccination rates?

___A “participatory” approach - start by asking the patient or family if they would like to talk about the vaccines that are due for the visit

___A “presumptive” approach – start by telling the patient or family which vaccines are due without initially asking for their input

___A “punitive” approach – gently, but firmly, scold the patient or family if they refuse vaccinations

___A “predetermined” approach – decide with the family at the prior visit which vaccinations will be given

___I do not know

6. As a communication technique used in discussing vaccines with vaccine-hesitant families, what does the CASE approach framework stand for?

___*Collaborate, Ask, Stay Engaged, Explain/Advise*

___*Corroborate, About Me, Science, Explain/Advise*

___*Corroborate, Ask, Stay Engaged, Exit*

___*Collaborate, Advise, Science, Explain/Advise*

___I do not know

7. True or False: The varicella vaccine is more likely to cause shingles later in life than a natural varicella infection.

___TRUE

___FALSE

8. Which of these statements is/are TRUE regarding mercury content in vaccines?

___Mercury is currently only contained in the influenza vaccine

___Mercury is present in several pediatric vaccines, but in amounts that are insignificant and safe for the body

___There is no longer any mercury contained in pediatric vaccines

___The form of mercury contained in vaccines builds up quickly in the body and is difficult to excrete.

9. True or False: The Influenza vaccine can cause a mild Influenza infection shortly after administration.

___TRUE

___FALSE

10. True or False: Vaccines should NOT be given when a patient has a fever.

___TRUE

___FALSE

11. True or False: A child is more likely to contract Pertussis from an adolescent or adult than from another child.

___TRUE

___FALSE

12. Which of these statements is TRUE regarding the Hepatitis B virus?

___Hepatitis B can only be contracted during birth if the mother tested positive for Hepatitis B during pregnancy.

___Hepatitis B is highly contagious and can even be transmitted through casual contact such as sharing a razor or toothbrush.

___Hepatitis B is most commonly transmitted by a patient who already has symptoms of liver failure.

___The earlier a baby or child is infected with Hepatitis B, the less likely they are to have serious and chronic complications such as liver cancer or liver failure.

13. Which of these statements is TRUE regarding the live attenuated vaccines?

___Live vaccines should never be given to pregnant women

___Live vaccines should never be given to household contacts of pregnant women

___Live vaccines should never be given to breastfeeding women

___Live vaccines should never be given to household contacts of immunocompromised individuals

14. Which of the following is a potentially severe, though rare, side effect of the Rotavirus vaccine?

___Severe Rotavirus infection and dehydration

___Intussusception

___Encephalitis

___Seizure

___There have been no serious side effects attributed to the Rotavirus vaccine

15. The CDC recommends initiation of the Human Papilloma Virus vaccine starting at what age?

___Age 11 in girls and age 12 in boys

___Age 11-12 in both girls and boys

___Age 12 in girls and age 13 in boys

___Age 12-13 in both girls and boys

16. Which of these statements is TRUE regarding the Measles, Mumps and Rubella vaccine?

___Some components of this vaccine are made using hen’s eggs or chick embryos and therefore should not be given to egg-allergic patients.

___Some components of this vaccine are made using hen’s eggs or chick embryos but are safe and recommended to be given to egg-allergic patients.

___This vaccine does not use eggs and is safe and recommended to be given to egg-allergic patients.

17. True or False: Studies have shown there is a slightly increased risk of Guillain Barre Syndrome after receiving the Influenza vaccine.

___TRUE

___FALSE

18. Which of these statements is TRUE regarding the Pertussis vaccine?

___The acellular Pertussis vaccine has a better safety profile and is more effective than the older “whole cell” version of the vaccine.

___The acellular Pertussis vaccine may be associated with local pain/swelling, but has no known serious side effects.

___The acellular Pertussis vaccine is associated with hypotonic-hyporesponsive syndrome in rare patients.

___The acellular Pertussis vaccine contains weakened bacterial cells.
