# Supplementary material for: Addressing Vaccine Hesitancy Through a Comprehensive Resident Vaccine Curriculum
Source: MedEdPORTAL. 2022 Dec 27;18:11292. doi: 10.15766/mep_2374-8265.11292 (PMC9792628; doi:10.15766/mep_2374-8265.11292)
Supplement: Supplementary file 1 — Vaccine Curriculum Facilitator Guide.docxVaccines Part 1.pptxVaccines Part 2.pptxVaccines Part 3 - Myths and Facts.pptxVaccines Part 4 - Communication Skills.pptxVaccine Hesitancy Communication Cases.docxVaccine Pretest.docxVaccine Posttest.docxPre- and Posttest Answer Key.docxSP Case and Notes for SP.docxSP Case Development Tool.docxSP Case - Learner Version.docxSP Assessment Checklist.docx [file mep_2374-8265.11292-s001.zip › J. SP Case and Notes for SP.docx]

**Standardized Patient Role:**

**You are the parent of a 2-year-old girl, Ashley. You have brought her to clinic today for a runny nose and cough. She has not had fevers. The doctor has diagnosed her with a viral upper respiratory infection and instructed you that it will run its course. She is a bit fussy and tired, but otherwise is doing OK.**

**The doctor now returns to the room toward the end of the visit and recommends that Ashley receive the influenza vaccine. You are hesitant about this. You have never given Ashley the influenza vaccine and you have heard that it can cause the flu. You have a 5-month-old infant at home and do not want Ashley to get the vaccine and then pass the flu on to the infant. You are also worried that you should not give her the vaccine when she is already sick. Finally, you have been hearing that the flu vaccine doesn’t even work. The physician discusses these concerns with you during the visit.**

Notes for the SP:

-When the physician (the learner) introduces the topic of flu vaccination with you, please shake your head immediately and say something along the lines of “No, no… we don’t want her to get that,” but do not initially offer a reason why.

-If the physician asks why you are hesitant about the vaccine, at first please say “Oh, I just don’t think it is right for her… maybe next time we come in we can talk about it.”

-If the physician probes to understand why you are hesitant about the vaccine (e.g., asks a second time), you can offer the reason that you “heard it actually gives people the flu.”

-Once the physician (hopefully) addresses that concern, please offer that you also “have a 5-month-old infant at home, and won’t it put the baby at risk if Ashley gets the shot?”

-Once the physician (hopefully) addresses that concern, say, “well I heard the flu shot doesn’t even work this year, so it isn’t worth the risks.”

-The physician should address that concern as well. IF the physician has not already mentioned the potential side effects of the vaccine, please ask “but aren’t there any side effects from getting the flu shot?”

-Once he or she addresses that, please say “Well, I don’t want her to get the shot when she is so sick, so I guess we can’t do it today anyway. Maybe next time.”

-The physician should address that concern and make a plan with you going forward – most likely for you to return to the clinic at a later date to either get the vaccine OR to discuss the vaccine further. If the physician continues to push to get the vaccine today, please say you’d like to “think about it more” or “discuss with my spouse” – don’t agree to the vaccine just yet. This ends the visit.

-If the physician gets confrontational or not emphatic enough or does not seem to try to understand your concerns, you should get defensive and less interested in discussing with them.
