# Supplementary material for: Addressing Vaccine Hesitancy Through a Comprehensive Resident Vaccine Curriculum
Source: MedEdPORTAL. 2022 Dec 27;18:11292. doi: 10.15766/mep_2374-8265.11292 (PMC9792628; doi:10.15766/mep_2374-8265.11292)
Supplement: Supplementary file 1 — Vaccine Curriculum Facilitator Guide.docxVaccines Part 1.pptxVaccines Part 2.pptxVaccines Part 3 - Myths and Facts.pptxVaccines Part 4 - Communication Skills.pptxVaccine Hesitancy Communication Cases.docxVaccine Pretest.docxVaccine Posttest.docxPre- and Posttest Answer Key.docxSP Case and Notes for SP.docxSP Case Development Tool.docxSP Case - Learner Version.docxSP Assessment Checklist.docx [file mep_2374-8265.11292-s001.zip › L. SP Case - Learner Version.docx]

**SP Case – Learner Version**

**You have just finished seeing a 2-year-old patient, Ashley, for a sick visit. She had a runny nose, cough, and no fevers. You diagnosed her with a viral URI, and she is overall well-appearing although tired and fussy.**

**You left the room to complete some paperwork and realized that she did not have a flu shot this year. You return now to the room to discuss the flu vaccine with her parent. You have heard from your colleagues, previously, that the parent has been very hesitant about giving vaccines in the past.**
