# Supplementary material for: Addressing Vaccine Hesitancy Through a Comprehensive Resident Vaccine Curriculum
Source: MedEdPORTAL. 2022 Dec 27;18:11292. doi: 10.15766/mep_2374-8265.11292 (PMC9792628; doi:10.15766/mep_2374-8265.11292)
Supplement: Supplementary file 1 — Vaccine Curriculum Facilitator Guide.docxVaccines Part 1.pptxVaccines Part 2.pptxVaccines Part 3 - Myths and Facts.pptxVaccines Part 4 - Communication Skills.pptxVaccine Hesitancy Communication Cases.docxVaccine Pretest.docxVaccine Posttest.docxPre- and Posttest Answer Key.docxSP Case and Notes for SP.docxSP Case Development Tool.docxSP Case - Learner Version.docxSP Assessment Checklist.docx [file mep_2374-8265.11292-s001.zip › M. SP Assessment Checklist.docx]

**Standardized Patient Encounter: Assessment Checklist**

| **Behavior** | **0 points** | **1 point** |
| --- | --- | --- |
| **Conversation opening** | Uses confrontational approach (e.g., “I hear you do not want to vaccinate).  OR  Uses participatory approach (e.g., “Shall we talk about possible vaccinations today?” or “I wanted to chat about XX vaccines today”)  OR Any opening that does not follow presumptive approach | Uses *best practice* presumptive approach (e.g., “Your child is due to receive XX vaccines today”) |
| **Verbal language** | Uses judgmental or confrontational verbal language at any point in conversation | Uses non-judgmental and non-confrontational verbal language throughout |
| **Body language** | Uses judgmental or confrontational body language at any point in conversation OR does not sit down for conversation | Uses non-judgmental and non-confrontational body language throughout AND sits down for the conversation |
| **Use of eye contact** | Poor or no eye contact | Good eye contact |
| **Identification of parental concern** | Makes assumptions about parental concerns (e.g., “You are probably worried about autism”) OR does not seek to understand source of parental concern | Asks open-ended questions to elicit parental concerns. |
| **Corroboration** | Does not attempt to empathize with parent OR does not corroborate parental concerns OR does not appear genuine | Expresses empathy AND corroborates parental concerns; appears genuine |
| **Expresses experience** | Does not explain own background and ability to respond to immunization questions. | Explains own background and ability to respond to immunization questions. |
| **Knowledge** | Provides inaccurate or no information to address parental vaccine concerns OR does not do so in a confident manner. | Provides accurate information to refute common immunization concerns in a confident manner. |
| **Side effects** | Does not address or know any potential side effects of vaccine OR gives inaccurate information | Accurately addresses potential side effects of vaccine |
| **Vaccine-preventable illness** | Does not discuss risks or sequelae of the vaccine-preventable disease OR gives inaccurate information | Accurately discusses risks or sequelae of the vaccine-preventable disease. |
| **Additional resources** | Does not provide or suggest additional resources for the parent | Provides or suggests additional resources for the parent |
| **Plan for future** | Does not suggest follow up plans to either receive the vaccine or continue discussion OR allows vague plans such as “let’s discuss this again in the future” | Makes concrete plans/arranges follow up to either receive the vaccine or continue discussion e.g., “let’s make an appointment again next week to talk about this” |
