# Supplementary material for: Addressing Vaccine Hesitancy Through a Comprehensive Resident Vaccine Curriculum
Source: MedEdPORTAL. 2022 Dec 27;18:11292. doi: 10.15766/mep_2374-8265.11292 (PMC9792628; doi:10.15766/mep_2374-8265.11292)
Supplement: Supplementary file 1 — Vaccine Curriculum Facilitator Guide.docxVaccines Part 1.pptxVaccines Part 2.pptxVaccines Part 3 - Myths and Facts.pptxVaccines Part 4 - Communication Skills.pptxVaccine Hesitancy Communication Cases.docxVaccine Pretest.docxVaccine Posttest.docxPre- and Posttest Answer Key.docxSP Case and Notes for SP.docxSP Case Development Tool.docxSP Case - Learner Version.docxSP Assessment Checklist.docx [file mep_2374-8265.11292-s001.zip › K. SP Case Development Tool.docx]

*MedEdPORTAL* Standardized Patient Case Development Tool

Date of Case Creation: May 2019

Primary Case Author: Zarina Norton, MD

Secondary Case Author: Kaitlyn Olson, MD

Standardized Patient Educator: Kerensa Peterson

Name of Case: Parental Vaccine Hesitancy – Flu Vaccine

Name of educational and or assessment activity: Pediatric Resident Vaccine Hesitancy Curriculum

Patient Name: Ashley (2-year-old daughter of Standardized Patient) – child not physically in the room at the time

Chief Complaint: Vaccine hesitancy

Most likely Diagnosis and Differential with rationale from history and/or physical exam: N/A

Challenge question: Address concerns of the vaccine hesitant parent regarding the flu vaccine

Domains: Check all that apply

XX Professionalism

XX Communication and Interpersonal skills

- Medical History
- Physical exam

XX Shared Decision Making

XX Patient Education

- Clinical Reasoning
- Documentation
- Handoff
- Presentation
- Other:

Type and level of learner: Pediatric Resident (PGY-1, PGY-2, PGY-3)

Case Objectives: please list specific objectives for each of the domains you have checked above:

1. Demonstrate use of best practices in vaccine hesitancy communication.

2. Engage in shared-decision making with parent to make a plan for continued discussion and follow up.

3. Educate parent about myths and facts related to flu vaccine and the risks of natural influenza infection.

| SETTING: outpatient, in patient, ED, home, nursing home, rehab, group etc. | Outpatient primary care clinic |
| --- | --- |
| PATIENT PROFILE: Information about the “patient” that helps select an SP and helps the learner get an understanding of them as a person. SP will know more information about the patient than learner will ever ask but allows SP to portray a fully developed patient personality. If none of the items below are particulars for the case please write “all may be used.” | |
| Age range | 20-45 |
| Religious/spiritual background | All may be used |
| Sex (e.g., male, female, intersex, transwoman, transman) | All may be used |
| Sexual Orientation (e.g., heterosexual, lesbian, gay, bisexual, pansexual, queer, asexual) | All may be used |
| Gender expression (e.g., man, woman, gender queer) | All may be used |
| Race/ethnicity: | All may be used |
| Physical description (e.g., BMI, height range) | All may be used |
| Physical limitations | All may be used |
| Patient appearance (e.g., disheveled, hospital gown, business casual, casual) | All may be used |
| Moulage + location (e.g., none, bruises, scars, body piercing, tattoos) | All may be used |
| Affect (e.g., pleasant, cooperative) | Skeptical but cooperative and pleasant |
| Family group (e.g., who is family, who they live with) | Has a 2 year old daughter, Ashley, and a 5month old baby at home. |
| Education | All may be used |
| Level of health literacy | All may be used |
| Employment, if any - present and past, noting any current stresses | All may be used |
| Home/homeless - type of dwelling, number of stories, owned or rented | All may be used |
| Financial situation- any current stresses | All may be used |
| Insurance Status (e.g., un/under/insured, public/private, HMO/PPO) | All may be used |
| Habits (i.e., diet, exercise, caffeine, smoking, alcohol, drugs) | All may be used |
| Activities (i.e., hobbies, sports, clubs, friends) | All may be used |
| Typical day - what is the usual daily routine | All may be used |

| CASE INFORMATION | |
| --- | --- |
| Chief Concern: What the patient will say when greeted by the student. The patient’s primary reason for seeking medical care often stated in his/own words. | Vaccine hesitancy – initial refusal of vaccine (the encounter starts after the primary visit has been completed – resident returns back to the room to let them know the child is due for a flu shot) |
| Additional Concerns: Other, if any, concerns the patient has today (i.e., symptoms, requests, expectations, etc.) that will become part of set agenda. | N/A |
|  | |
| THE PATIENT STORY: The SP will be asked to tell their symptom story and the personal and emotion impact for each of their concerns. You will want to write this is the patient voice. The symptom story should be able to answer this question: “Tell me more about [chief concern/additional concern], starting at the beginning and bringing me up to now.”  The personal context should be able to answer questions concerning the broader personal/psychosocial context of symptoms, especially the patient beliefs/attributions.  The emotional context should be able to ask how are you doing with this, how does this make you feel, how has this affected you emotionally? IMPACT: How has this affected your life? How has this been for your family? | You are the mother or father of a 2 year old girl, Ashley. You have brought her to clinic today for a runny nose and cough. She has not had fevers. The doctor has diagnosed her with a viral upper respiratory infection and instructed you that it will run its course. She is a bit fussy and tired, but otherwise is doing OK.  The doctor now returns to the room toward the end of the visit and recommends that Ashley receive the influenza vaccine. You are hesitant about this. You have never given Ashley the influenza vaccine and you have heard that it can cause the flu. You have a 5 month old infant at home and do not want Ashley to get the vaccine and then pass the flu on to the infant. You are also worried that you should not give her the vaccine when she is already sick. Finally, you have been hearing that the flu vaccine doesn’t even work. The physician discusses these concerns with you during the visit.    Notes for the SP:  -When the physician (the learner) introduces the topic of flu vaccination with you, please shake your head immediately and say something along the lines of “No, no… we don’t want her to get that”, but do not initially offer a reason why.  -If the physician asks why you are hesitant about the vaccine, at first please say “Oh, I just don’t think it is right for her… maybe next time we come in we can talk about it”.  -If the physician probes to understand why you are hesitant about the vaccine (e.g. asks a second time), you can offer the reason that you “heard it actually gives people the flu”.  -Once the physician (hopefully) addresses that concern, please offer that you also “have a 5 month old infant at home, and won’t it put the baby at risk if Ashley gets the shot”?  -Once the physician (hopefully) addresses that concern, say, “well I heard the flu shot doesn’t even work this year so it isn’t worth the risks”.  -The physician should address that concern as well. IF the physician has not already mentioned the potential side effects of the vaccine, please ask “but aren’t there any side effects from getting the flu shot?”  -Once he or she addresses that, please say “Well, I don’t want her to get the shot when she is so sick, so I guess we can’t do it today anyway. Maybe next time”.  -The physician should address that concern and make a plan with you going forward – most likely for you to return to the clinic at a later date to either get the vaccine OR to discuss the vaccine further. If the physician continues to push to get the vaccine today, please say you’d like to “think about it more” or “discuss with my spouse” – don’t agree to the vaccine just yet. This ends the visit.  -If the physician gets confrontational or not emphatic enough or does not seem to try to understand your concerns, you should get defensive and less interested in discussing with them. |
| HISTORY OF PRESENT ILLNESS: Although some of the HPI will be given in the patient’s symptom story, the learners will expand the story during the direct question section. Below describe the detailed history, usually about the chief concern, which the student must develop in order to make a useful assessment of the problem: | |
|  | |
| Onset (when; gradual or sudden) | N/A |
| Setting (what was going on or where was patient when symptoms first noticed?) | N/A |
| Duration (how long) | N/A |
| Time relationships (frequency, constant or intermittent) | N/A |
| Location | N/A |
| Radiation | N/A |
| Quality | N/A |
| Amount | N/A |
| Aggravated by what | N/A |
| Relieved by what | N/A |
| Associated with what | N/A |
| Attitude (what does the patient think is the problem, and how does he/she feel about it) | N/A |
| Overall course | N/A |
| REVIEW OF SYSTEMS: Significant positives and negatives | |
|  | N/A |
|  |  |
|  |  |
|  |  |
|  | |
| Past medical history | N/A |
| Medication allergies (Name and reaction) | N/A |
| Environmental allergies (Name and reaction) | N/A |
| Illnesses | N/A |
| Vaccinations | N/A |
| Surgeries | N/A |
| Accidents/ injuries/ trauma | N/A |
| Hospitalization | N/A |
|  | |
| Inclusive sexual and reproductive history | |
| Sexual practices  Sexual partners  Protection: Use of safer sex practices  Use of birth control if appropriate  Risk of intimate partner violence | N/A |
| Ob/GYN HISTORY | Age of onset of menses  Age of menopause  Number of pregnancies  Number of live births  Number of miscarriages  Number of abortions |
| Medications | Prescription/dose/reason  Over the counter/dose/reason  Herbs/supplements/dose/reason  Other: |
| Immunizations | - Tetanus - Flu - Hepatitis - Pneumovax - HPV - Other |
| Tobacco products:   - Cigarettes - Cigar - Pipe - Chew - E-cigarettes | - Never - Past- year started/year quit - Current   - Quantity   - # of years |
| Alcohol   - Beer - Wine - Liquor - Other | - Never - Past- year started/year quit - Current   - Quantity   - # of years |
| Drugs   - Weed - Cocaine - Heroin - Meth - Other - IV - Inhalants - Other | - Never - Past- year started/year quit - Current   - Quantity - # of years |
| Diet (describe) | N/A |
| Exercise (describe) | N/A |
| List any other important social history or information important to this case | See description above |
| Family history | N/A |
| Mother, Father, Siblings, Grandparents, and other significant findings. | N/A |
|  |  |
| Physical Exam- List exam maneuvers expected for this case and any abnormal findings that SP will simulate. (tenderness, hyper-hypo reflex, rebound, weakness etc. ) | |
| PHYSICAL EXAM FINDINGS | N/A |
| 1. Written in layman’s terms | N/A |
| 1. General appearance- affect, appearance, position of patient at opening (i.e. sitting, laying down, holding abdomen etc.) | N/A |
| 1. Vital signs | N/A |
| 1. Specific findings and affect | N/A |
| 1. Response to certain physical movements | N/A |
|  |  |
| DIAGNOSIS AND DIFFERENTIAL |  |
| Diagnosis with support from positive and negative history and PE findings | N/A |
| Differential with support from positive and negative history and PE findings | N/A |
|  |  |
| MANAGEMENT OR DIAGNOSTIC PLAN | Resident to make a plan with the parent for continued discussion and follow up, provide resources and education regarding flu vaccine. |
|  |  |
| PROFESSIONALISM ISSUES OR CHALLENGES: | Resident to maintain professionalism while developing an understanding of the parent’s concerns, providing education, debunking myths and formulating a plan in conjunction with the parent.  Resident to utilize best practices of vaccine hesitancy communication – presumptive instead of participatory approach to vaccine, non-judgmental language, display empathy and ask for parental concerns, corroborate concerns, debunk false claims, engage parent in discussion and make plans for continued discussion. |
