# Supplementary material for: Addressing Vaccine Hesitancy Through a Comprehensive Resident Vaccine Curriculum
Source: MedEdPORTAL. 2022 Dec 27;18:11292. doi: 10.15766/mep_2374-8265.11292 (PMC9792628; doi:10.15766/mep_2374-8265.11292)
Supplement: Supplementary file 1 — Vaccine Curriculum Facilitator Guide.docxVaccines Part 1.pptxVaccines Part 2.pptxVaccines Part 3 - Myths and Facts.pptxVaccines Part 4 - Communication Skills.pptxVaccine Hesitancy Communication Cases.docxVaccine Pretest.docxVaccine Posttest.docxPre- and Posttest Answer Key.docxSP Case and Notes for SP.docxSP Case Development Tool.docxSP Case - Learner Version.docxSP Assessment Checklist.docx [file mep_2374-8265.11292-s001.zip › Educational Summary Report_11292.pdf]

MedEdPORTAL® The AAMC Journal of Teaching and Learning Resources

Original Publication Open Access

Addressing Vaccine Hesitancy Through a Comprehensive Resident Vaccine Curriculum

Zarina S. Norton, MD*, Kaitlyn B. Olson, MD, Sandra M. Sanguino, MD, MPH

*Corresponding author: znorton@luriechildrens.org

Abstract

Introduction: Vaccine hesitancy can lead to incomplete vaccination, increased risk of vaccine-preventable diseases, and distrust or conflict between physicians and patients. Yet many physicians are uncomfortable navigating vaccine hesitancy and educating vaccine-hesitant patients and families. We developed a vaccine hesitancy curriculum to increase vaccine knowledge, comfort, and communication skills in pediatric residents. **Methods:** The curriculum consisted of four interactive 40-minute sessions delivered to pediatric residents over 10 months. The first two sessions discussed recommended childhood vaccines, the third session examined common vaccine misconceptions, and the final session reviewed vaccine hesitancy–specific communication skills, incorporating practice through role-playing. Residents completed pre- and posttests assessing knowledge and comfort as well as receiving a standardized patient (SP) assessment of vaccine-specific communication skills after the curriculum. **Results:** Thirty-five residents were in the educational intervention group and 35 in a control group. Pretest scores did not differ significantly between the groups. The mean knowledge score for the intervention group increased from 47% on the pretest to 66% on the posttest. The mean self-reported comfort score (1 = *low comfort*, 5 = *high comfort*) for the intervention group increased from 2.9 on the pretest to 3.8 on the posttest. The control group showed no difference between pre- and posttest scores for knowledge or comfort. The mean postintervention SP assessment score was significantly higher for the intervention group (78%) than the control group (52%). **Discussion:** Implementation of a comprehensive vaccine hesitancy curriculum resulted in improved vaccine knowledge, self-reported comfort, and communication skills among pediatric residents.

Keywords
Vaccine Hesitancy, Clinical/Procedural Skills Training, Communication Skills, Pediatrics, Preventive Medicine, Primary Care, Standardized Patient

Educational Objectives

By the end of this activity, learners will be able to:

1. Recall and discuss facts related to routine pediatric vaccines and their benefits, components, and possible side effects/complications.
2. Identify common misconceptions about pediatric vaccines and formulate responses to concerns commonly identified by vaccine-hesitant patients and families.
3. Demonstrate best-practice principles of communication with vaccine-hesitant patients and families.
4. Apply communication techniques in simulated patient discussions.

Citation:
Norton ZS, Olson KB, Sanguino SM. Addressing vaccine hesitancy through a comprehensive resident vaccine curriculum. *MedEdPORTAL*. 2022;18:11292.
https://doi.org/10.15766/mep_2374-8265.11292

Introduction

Vaccine hesitancy remains prevalent and was listed as a top 10 threat to global health in 2019 by the World Health Organization.[1] Freed and colleagues reported that almost one in eight parents surveyed had refused at least one recommended pediatric vaccine.[2] In 2018-2019, only 62% of children and 45% of adults received flu vaccines,[3] and hesitancy regarding COVID-19 vaccination has created additional challenges during the COVID-19 pandemic.[4,5]

Vaccine hesitancy can lead to delayed or incomplete vaccination and an increased risk of vaccine-preventable diseases. In 2017, the national up-to-date rate for the routine vaccines due in the first 15 months of life was only 70%.[6] As a result of vaccine hesitancy, there have been resurgences of several vaccine-preventable diseases. For instance, more measles cases were

Copyright © 2022 Norton et al. This is an open-access publication distributed under the terms of the Creative Commons Attribution license.

reported in the United States in the first half of 2019 than in any full year since 1994.[7]

Vaccine hesitancy can also lead to distrust or conflict between physicians and patients.[8] One study reported that nearly 30% of pediatricians surveyed had dismissed at least one patient from their practice due to vaccine refusal.[9] It is well documented in pediatric, family practice, and internal medicine literature that many primary care physicians are uncomfortable addressing vaccine concerns even though they believe doing so to be important to their practice.[10-13] Studies have repeatedly shown that strong physician vaccine recommendations increase patient vaccine confidence and compliance.[14-16] Thus, physicians must be equipped with the knowledge and skills to discuss vaccine hesitancy with patients and their families.

Only a few published curricula address this clinical and educational need. Morhardt and colleagues showed that a small cohort of pediatric residents had increased self-perceived comfort level, self-perceived competence, and performance in a role-playing exercise after receiving a curriculum on vaccine hesitancy.[17] However, their curricular materials largely provided general guidance on topics to cover rather than comprehensive, ready-to-deliver educational content covering each vaccine in detail, vaccine misconceptions, and communication skills training. Additionally, the communication skills portion of their curriculum primarily recommended the CASE (corroborate, about me, science, explain/advise) approach[18] rather than providing learners with other principles of vaccine hesitancy communication to develop a broad skill set for differing clinical scenarios. Real and colleagues demonstrated a decrease in influenza vaccine refusal rates in patients of residents who underwent a virtual reality curriculum on vaccine hesitancy, compared to patients of residents who had not received the curriculum.[19] However, virtual reality may not be a readily accessible training option for many residency programs. Other published curricula focus primarily on knowledge acquisition rather than communication skills training.[20,21] We are not aware of any randomized controlled studies evaluating the impact of a comprehensive vaccine hesitancy curriculum on resident knowledge and vaccine hesitancy communication skills. Given the prevalence of vaccine hesitancy towards influenza and COVID-19, as well as routine childhood vaccinations, this gap in training needs to be addressed in pediatrics and across disciplines.

We developed a resident vaccine hesitancy curriculum to address this gap. We hypothesized that residents who took the curriculum would show improvements in vaccine knowledge, comfort level, and communication skills in vaccine hesitancy discussions compared to residents who did not take the curriculum.

Methods

Setting and Population

We evaluated a comprehensive vaccine hesitancy curriculum using a randomized controlled trial design. Participants included all residents at a pediatric residency continuity clinic site affiliated with our academic tertiary care pediatric hospital. The institutional review board at our institution deemed further review of this project not necessary.

Curriculum Design and Implementation

We used Kern's framework for curriculum development.[22] We conducted a targeted needs assessment by emailed survey to guide curriculum design. The vaccine hesitancy curriculum was implemented based on a developed facilitator's guide (Appendix A) during predesignated protected educational time in continuity clinic sessions over the course of 10 months. The curriculum consisted of two 40-minute educational sessions on the recommended pediatric vaccines, including benefits, composition, side effects, and potential complications of each vaccine (Appendices B and C); one 40-minute educational session on addressing common vaccine misconceptions (Appendix D); and one 40-minute session on vaccine communication skills, during which residents had the opportunity to role-play using brief vignettes of common parental vaccine concerns with real-time instructor guidance (Appendices E and F). The communication skills session reviewed published communication frameworks and focused on overarching best practices in vaccine communication, such as corroboration of parental concerns, use of open-ended questions and nonjudgmental language, establishment of follow-up, use of a longitudinal approach, and use of a presumptive instead of participatory initial approach to vaccine discussions.[10,14,18,23,24]

The presentation slide sets for each of the four sessions were developed by our curriculum development team with input from local experts on vaccines, primary care, and communication. Information about each pediatric vaccine and general vaccine misconceptions was primarily obtained from resources such as the American Academy of Pediatrics and Centers for Disease Control and Prevention websites, as well as the Children's Hospital of Philadelphia's Vaccine Education Center.[25-27]

Each educational session had one facilitator and six to eight learners and took place during protected education time at the beginning of continuity clinic sessions. Third-year residents served as the facilitators for the first three educational sessions.

Copyright © 2022 Norton et al. This is an open-access publication distributed under the terms of the Creative Commons Attribution license.

In our continuity clinic model, most educational sessions occurring during protected education time were developed and led by residents. In keeping with this practice, we identified dates when third-year residents were scheduled to lead education in each clinic, asked each resident if they were willing to deliver a component of the vaccine hesitancy curriculum, and scheduled our curricular sessions accordingly. Our curriculum development team reviewed slide sets with the resident facilitators ahead of time, answering any questions they had about content and/or delivery. Residents did not require any other prior knowledge or training before leading sessions. Continuity clinic faculty were present during each session to provide additional support.

A member of our curriculum development team facilitated the communication skills workshops with the support of continuity clinic faculty. We chose to have a member of our team, rather than residents, lead the communication skills workshops because we felt some prior knowledge of communication techniques and experience with vaccine hesitancy communication would be beneficial. The facilitator was familiar with literature on vaccine hesitancy communication.

Curriculum Evaluation and Learner Assessment

We evaluated the curriculum using a randomized controlled trial design. For block randomization, we used a random number generator[28] to assign four of the eight half-day continuity clinic sessions at our continuity clinic site to the intervention group; all residents who attended those clinics received the longitudinal curriculum. The other four clinic sessions served as a control group and continued standard practice, in which residents themselves developed and delivered educational sessions on vaccines, ranging from one to two sessions per clinic over the course of a year. The educational sessions in the control group clinics varied greatly by clinic session and included some discussion of vaccines but not necessarily of vaccine hesitancy. None of the control group didactic sessions included discussion of specific communication skills or allowed time for role-playing or practice. Although our curriculum was not strictly confidential, we asked that residents in the intervention group not share curricular materials until the evaluation period was complete, making it unlikely that residents in the control group had specific knowledge of our curriculum's content or evaluation plans. Residents attended only their assigned clinic session, minimizing contamination between the intervention and control groups.

Both the control and intervention groups were given a written pretest to assess vaccine knowledge prior to the start of the curriculum and a written posttest at the end of the evaluation period (Appendices G, H, and I). The pre- and posttests consisted of 12 identical multiple-choice questions assessing knowledge about vaccines, such as side effects, indications, and composition of vaccines. Approximately 25 knowledge assessment questions were originally developed by our curriculum development team; however, after iterative review by our team and continuity clinic faculty, 12 questions were deemed most relevant to include in the pre- and posttests. Two additional questions assessed residents' self-perceived comfort level with vaccine hesitancy discussions using an anchored rating scale (1 = *low comfort*, 5 = *high comfort*). There were also two questions about communication skills, which were ultimately not used in the analysis, as we chose to assess communication skills through a standardized patient (SP) exercise instead.

To assess communication skills, residents in both groups engaged in an SP encounter at the end of the evaluation period, during which a trained SP portrayed a vaccine-hesitant parent. The case was developed by our curriculum development team, using the commonly encountered scenario of a parent expressing hesitancy about the influenza vaccine (Appendices J and K). SPs were hired through our medical school's SP program. SP training was led by the medical school's SP educator in conjunction with our curriculum development team and included a detailed review of the case and evaluation method and numerous practice sessions with individuals who were not involved in the curriculum or evaluation.

SP encounters took place in a patient care room in the resident continuity clinic site once during each of the eight continuity clinic sessions. Each session lasted approximately 3 hours, during which five to eight residents completed the SP encounter. Only residents who were present in clinic that day were able to participate in the SP encounter; residents were unable to participate if they were absent from clinic due to vacation, illness, or night shift. Because the SP encounter assessments were done during continuity clinic sessions, evaluators were not masked to resident control or intervention group assignment. SPs, however, were masked to resident group assignment.

Residents were given a brief case vignette (Appendix L) just prior to entering the SP encounter. No other materials were required. Each SP encounter lasted 7-10 minutes. Although the SP portion of the encounter was scripted, the varying length of encounters was attributable to variable thoroughness of resident answers to scripted SP questions (such as questions about vaccine side effects, contraindications, and effectiveness).

To assess residents during SP encounters, we developed a performance checklist using a modified Delphi method, with

Copyright © 2022 Norton et al. This is an open-access publication distributed under the terms of the Creative Commons Attribution license.

iterative input from local experts in the fields of primary care, communication, and vaccines (Appendix M). The 12-item checklist included general communication techniques as well as vaccine hesitancy best practices.[10,14,18,23,24] Some of the 12 items were initially graded 0, 1, or 2 to account for partial completion of an item and to better provide granular formative feedback to each resident after encounters. We dichotomized our data so that scores of 0 or 1 were counted as not completed (0) and scores of 2 were counted as completed (1); this was an a priori decision for analytic consistency across all items. One of two evaluators completed the checklist while observing each SP encounter. We used 10 pilot sessions with individuals who were not participants in the curriculum or evaluation to ensure interrater reliability.

Resident participation in pretests, posttests, and SP encounters was voluntary and primarily for the purpose of curriculum evaluation, rather than being formative or summative assessment. We did not use identifiers or link pre- and posttest scores to individual residents; thus, scores were not returned to residents for feedback. SP encounters were not anonymous; evaluators discussed scores with each participating resident immediately after their SP encounter and provided formative feedback.

Data Analysis

Data were collected in a deidentified manner. Group means for pre- and posttest knowledge scores, an anchored rating scale for comfort level, and postcurriculum SP encounter performance scores were compared using analyses of variance to account for nesting of participants within clinic sessions. We assessed the effect size of the intervention on these metrics using η^2 for the knowledge and comfort data and Cohen's *d* coefficient for the SP data. Cronbach's alpha coefficient was calculated to assess the internal consistency of the comfort scale. Each SP encounter was scored by one of two evaluators, and interrater reliability was established prior to assessments (Cohen's $\kappa = .97$). We used Stata 17.0 (StataCorp) to conduct all statistical analyses, with two-tailed tests using a *p* value of $<.05$ considered statistically significant. We conducted skewness and kurtosis tests for normality; in all cases, the null hypotheses that the data were normal were not rejected.

Results

Knowledge Outcomes

The 70 pediatric residents at the participating continuity clinic site were randomized based on clinic session to the intervention group ($n = 35$) or the control group ($n = 35$). Because residents were randomly assigned to clinic sessions prior to starting residency, the eight clinic sessions had similar compositions of residents, with equal distribution of resident gender, postgraduate year, and race/ethnicity (Table 1). Fifty-three residents (76%) completed the pretest, and 58 residents (83%) completed the posttest.

Pretest knowledge scores did not differ significantly between the control and intervention groups (Table 2). For the intervention group, the mean score on the 12 didactic knowledge questions increased from 47% (95% CI, 44%-51%) on the pretest to 66% (95% CI, 62%-70%) on the posttest, with an η^2 of .30. There was no significant difference between pre- and posttest knowledge

Table 1. Control and Intervention Group Resident Demographics and Curriculum Evaluation Data

	Control Group[a]				Intervention Group[b]			
Demographic	No. (%)	Completed Pretests: No. (%)[c]	Completed Posttests: No. (%)[d]	Completed SP Encounters: No. (%)[c]	No. (%)	Completed Pretests: No. (%)[e]	Completed Posttests: No. (%)[d]	Completed SP Encounters: No. (%)[f]
Training level								
PGY 1	11 (31)	9 (36)	10 (34)	9 (36)	11 (31)	10 (36)	10 (34)	8 (33)
PGY 2	12 (34)	8 (32)	8 (28)	7 (28)	12 (34)	9 (32)	10 (34)	7 (29)
PGY 3	12 (34)	8 (32)	11 (38)	9 (36)	12 (34)	9 (32)	9 (31)	9 (38)
Sex								
Female	26 (74)				28 (80)			
Male	9 (26)				7 (20)			
Race/ethnicity								
Asian	4 (11)				3 (9)			
Black	1 (3)				1 (3)			
Hispanic/Latinx	1 (3)				1 (3)			
White	29 (83)				30 (85)			

Abbreviations: PGY, postgraduate year; SP, standardized patient.
[a] $N = 35$. Clinic sessions: Tuesday AM, Wednesday PM, Thursday AM, Friday AM.
[b] $N = 35$. Clinic sessions: Monday PM, Tuesday PM, Wednesday AM, Thursday PM.
[c] $N = 25$.
[d] $N = 29$.
[e] $N = 28$.
[f] $N = 24$.

Copyright © 2022 Norton et al. This is an open-access publication distributed under the terms of the Creative Commons Attribution license.

Table 2. Resident Performance on Didactic Knowledge Portion of Pre- and Posttests

	Didactic Knowledge Scores					
	Pretest		Posttest			
Group	*M* (95% CI)	No.	*M* (95% CI)	No.	*p*	Effect Size η^2 (95% CI)
Control	43% (37%-48%)	25	40% (36%-45%)	29	.44	
Intervention	47% (44%-51%)	28	66% (62%-70%)	29	<.001	.30 (.10-.50)

Table 4. Resident Performance on SP Assessments

	SP Scores			
Group	*M* (95% CI)	No.	*p*	Effect Size Cohen's *d* (95% CI)
Control	52% (49%-56%)	25		
Intervention	78% (73%-82%)	24	<.001	2.5 (1.8-3.3)

Abbreviation: SP, standardized patient.

scores for the control group, which scored a mean of 43% (95% CI, 37%-48%) on the pretest and 40% (95% CI, 36%-45%) on the posttest.

Comfort-Level Outcomes

Pretest comfort-level scores did not differ significantly between the control and intervention groups (Table 3). For the intervention group, the mean score on the comfort-level questions increased from 2.9 (95% CI, 2.7-3.1) on the pretest to 3.8 (95% CI, 3.6-3.9) on the posttest. There was no significant difference between pre- and posttest comfort scores for the control group, which scored a mean of 2.9 (95% CI, 2.7-3.3) on the pretest and 3.0 (95% CI, 2.8-3.2) on the posttest. Cronbach's alpha for comfort-level scales was .86.

SP Encounter Evaluation

Forty-nine residents (70%) participated in the voluntary SP assessment. This included 25 of 35 residents from the control group (71%) and 24 of 35 residents from the intervention group (69%). Of note, six of 35 residents from the control group and seven of 35 residents from the intervention group were absent on their assigned clinic day due to vacation, illness, or night shift, and thus were unable to participate in the SP assessment. The mean total score for the control group was 52% (95% CI, 49%-56%), while the intervention group mean score was 78% (95% CI, 73%-82%). The Cohen's *d* coefficient was 2.5, indicating a large effect size of the intervention (Table 4).

Discussion

In this curricular evaluation, we demonstrated that a comprehensive vaccine hesitancy curriculum resulted in significant increases in pediatric resident vaccine knowledge, improved self-perceived comfort, and improved communication skills and performance in simulated vaccine hesitancy discussions. Many studies have focused on decreasing vaccine hesitancy through educational interventions at the community, family, or patient level.[29] Few have sought to train physician trainees to effectively address vaccine hesitancy, and none have demonstrated success of a comprehensive curriculum in improving trainees' knowledge, comfort, and communication skills in vaccine hesitancy discussions compared to a control group. Although reasons for vaccine hesitancy are multifactorial, training physicians to effectively discuss these issues with patients and families is crucial and can mitigate vaccine refusal and decrease rates of vaccine-preventable disease. As previously noted, we demonstrated increased comfort level with vaccine hesitancy in pediatric residents after the curriculum. Although comfort level is generally not considered a robust outcome in educational studies, in this case, increased comfort level could facilitate discussions between physicians and families, which have been shown to increase vaccine acceptance over time.[10,14]

Table 3. Resident Performance on Comfort-Level Portion of Pre- and Posttests

	Self-Reported Comfort-Level Scores[a]					
	Pretest		Posttest			
Group	*M* (95% CI)	No.	*M* (95% CI)	No.	*p*	Effect Size η^2 (95% CI)
Control	2.9 (2.7-3.3)	25	3.0 (2.8-3.2)	29	.99	
Intervention	2.9 (2.7-3.1)	28	3.8 (3.6-3.9)	29	.002	.51 (.31-.64)

[a]Scored using an anchored rating scale (1 = *low comfort*, 5 = *high comfort*).

Our experience indicates the feasibility of incorporating the curriculum within the constraints of a busy resident schedule; in our case, the curriculum was implemented during already scheduled education time, via four relatively brief (40-minute) sessions throughout the academic year. Informally collected feedback about residents' experience with the curriculum was overwhelmingly positive, although some feedback indicated that more time for each session might have been valuable. The curriculum itself did not have an associated cost; the hiring of SPs for evaluation of the curriculum cost less than $1,000. Faculty time was provided in kind.

The evaluation had several limitations. All 70 enrolled residents did not complete all portions of the evaluation, as they were voluntary. It is possible that those who chose to participate in the evaluation components were already more knowledgeable, comfortable, and/or skilled regarding vaccine hesitancy. Nevertheless, we demonstrated significant improvements for the intervention group compared to the control group in all areas measured. As we did not use identifiers, we were unable to track individual increases in pre- and posttest scores or compare knowledge, comfort-level, and SP scores for individual residents.

Copyright © 2022 Norton et al. This is an open-access publication distributed under the terms of the Creative Commons Attribution license.

In an effort to optimize resident availability to participate in the voluntary SP encounter assessments, the encounters were conducted during each continuity clinic session at the clinic site. However, because the SP encounter assessments were done in clinic sessions, evaluators were not masked to resident group assignment.

The evaluation was conducted at a single residency program's academic continuity clinic site, which may limit generalizability to some degree. However, we believe the curriculum could be adapted for a variety of settings. The curricular components could be delivered during educational time, such as resident conferences or academic half-days, or as continuing medical education. Didactic portions of the curriculum could be used for self-directed learning. Vaccine hesitancy is not limited to the pediatric population, as evidenced by hesitancy about influenza and COVID-19 vaccination in the general population. Thus, the curriculum could be utilized by medical providers across disciplines who routinely discuss or administer vaccines (e.g., family medicine or internal medicine trainees, new or established primary care physicians or nurse practitioners). We have already successfully delivered a modified version of our curriculum to fourth-year medical students during their end-of-year boot camp.

As this was an educational evaluation, our primary outcomes were resident knowledge and skill acquisition. Ultimately, it will be important to assess the impact of the curriculum on outcomes such as parental vaccine hesitancy, vaccine acceptance, and rates of vaccine-preventable diseases.

Conclusion

A low-cost, comprehensive, longitudinal curriculum on vaccine hesitancy resulted in increases in vaccine knowledge, improved comfort level, and improved communication skills and performance in simulated vaccine hesitancy discussions. The curriculum should be studied further to evaluate its effectiveness in other groups of learners and its impact on parent and patient outcomes.

Appendices

A. Vaccine Curriculum Facilitator Guide.docx
B. Vaccines Part 1.pptx
C. Vaccines Part 2.pptx
D. Vaccines Part 3 - Myths and Facts.pptx
E. Vaccines Part 4 - Communication Skills.pptx
F. Vaccine Hesitancy Communication Cases.docx
G. Vaccine Pretest.docx
H. Vaccine Posttest.docx
I. Pre- and Posttest Answer Key.docx
J. SP Case and Notes for SP.docx
K. SP Case Development Tool.docx
L. SP Case - Learner Version.docx
M. SP Assessment Checklist.docx

All appendices are peer reviewed as integral parts of the Original Publication.

Zarina S. Norton, MD: Assistant Professor, Department of Pediatrics, Ann & Robert H. Lurie Children's Hospital of Chicago; Assistant Professor, Department of Medical Education, Northwestern University Feinberg School of Medicine; ORCID: https://orcid.org/0000-0002-5424-8955

Kaitlyn B. Olson, MD: General Pediatrician, Department of Pediatrics, Cottage Children's Medical Center

Sandra M. Sanguino, MD, MPH: Associate Professor, Department of Pediatrics, Ann & Robert H. Lurie Children's Hospital of Chicago; Associate Professor, Department of Medical Education, and Senior Associate Dean for Medical Education, Northwestern University Feinberg School of Medicine

Acknowledgments
We would like to acknowledge the pediatric residents who actively participated in this curriculum and voluntarily participated in components of curricular evaluation; the Feinberg School of Medicine's Standardized Patient Program for providing guidance and standardized patients, as well as the Standardized Patient Educator, Ms. Kerensa Peterson; the Augusta Webster Faculty Foundation Fellowship program, an internal educational grant through which we received funding for standardized patients, and Dr. Mark Adler, who runs the grant program and provided mentorship; and the continuity clinic director, Dr. Maheen Quadri, for her help and support.

Disclosures
None to report.

Funding/Support
None to report.

Prior Presentations
Norton ZS. Development and evaluation of a longitudinal vaccine hesitancy curriculum. Presented at: Association of Pediatric Program Directors Spring Meeting; March 26-29, 2019; New Orleans, LA.

Norton ZS. Development and evaluation of a comprehensive longitudinal curriculum on vaccine hesitancy. Presented at: Pediatric Academic Societies Annual Meeting; April 24-May 1, 2019; Baltimore, MD.

Copyright © 2022 Norton et al. This is an open-access publication distributed under the terms of the Creative Commons Attribution license.

Ethical Approval

The Ann & Robert H. Lurie Children's Hospital of Chicago's Institutional Review Board deemed further review of this project not necessary.

References

1. Ten threats to global health in 2019. World Health Organization. Accessed November 4, 2022. https://www.who.int/news-room/spotlight/ten-threats-to-global-health-in-2019
2. Freed GL, Clark SJ, Butchart AT, Singer DC, Davis MM. Parental vaccine safety concerns in 2009. *Pediatrics.* 2010;125(4): 654-659. https://doi.org/10.1542/peds.2009-1962
3. Flu vaccination coverage, United States, 2018-19 influenza season. Centers for Disease Control and Prevention. September 26, 2019. Updated January 26, 2022. Accessed November 4, 2022. https://www.cdc.gov/flu/fluvaxview/coverage-1819estimates.htm
4. Fisher KA, Bloomstone SJ, Walder J, Crawford S, Fouayzi H, Mazor KM. Attitudes toward a potential SARS-CoV-2 vaccine: a survey of U.S. adults. *Ann Intern Med.* 2020;173(12):964-973. https://doi.org/10.7326/M20-3569
5. Dror AA, Eisenbach N, Taiber S, et al. Vaccine hesitancy: the next challenge in the fight against COVID-19. *Eur J Epidemiol.* 2020;35(8):775-779. https://doi.org/10.1007/s10654-020-00671-y
6. Hill HA, Elam-Evans LD, Yankey D, Singleton JA, Kang Y. Vaccination coverage among children aged 19-35 months—United States, 2017. *MMWR Morb Mortal Wkly Rep.* 2018;67(40):1123-1128. https://doi.org/10.15585/mmwr.mm6740a4
7. U.S. measles cases in first five months of 2019 surpass total cases per year for past 25 years. Centers for Disease Control and Prevention. May 30, 2019. Accessed November 4, 2022. https://www.cdc.gov/media/releases/2019/p0530-us-measles-2019.html
8. Yaqub O, Castle-Clarke S, Sevdalis N, Chataway J. Attitudes to vaccination: a critical review. *Soc Sci Med.* 2014;112:1-11. https://doi.org/10.1016/j.socscimed.2014.04.018
9. Leib S, Liberatos P, Edwards K. Pediatricians' experience with and response to parental vaccine safety concerns and vaccine refusals: a survey of Connecticut pediatricians. *Public Health Rep.* 2011;126(2)(suppl):13-23. https://doi.org/10.1177/00333549111260S203
10. Henrikson NB, Opel DJ, Grothaus L, et al. Physician communication training and parental vaccine hesitancy: a randomized trial. *Pediatrics.* 2015;136(1):70-79. https://doi.org/10.1542/peds.2014-3199
11. Sarnquist C, Sawyer M, Calvin K, et al. Communicating about vaccines and vaccine safety: what are medical residents learning and what do they want to learn? *J Public Health Manag Pract.* 2013;19(1):40-46. https://doi.org/10.1097/PHH.0b013e3182495776
12. Williams SE, Swan R. Formal training in vaccine safety to address parental concerns not routinely conducted in U.S. pediatric residency programs. *Vaccine.* 2014;32(26):3175-3178. https://doi.org/10.1016/j.vaccine.2014.04.001
13. Arora G, Lehman D, Charlu S, et al. Vaccine health beliefs and educational influences among pediatric residents. *Vaccine.* 2019;37(6):857-862. https://doi.org/10.1016/j.vaccine.2018.12.038
14. Opel DJ, Heritage J, Taylor JA, et al. The architecture of provider-parent vaccine discussions at health supervision visits. *Pediatrics.* 2013;132(6):1037-1046. https://doi.org/10.1542/peds.2013-2037
15. Opel DJ, Zhou C, Robinson JD, et al. Impact of childhood vaccine discussion format over time on immunization status. *Acad Pediatr.* 2018;18(4):430-436. https://doi.org/10.1016/j.acap.2017.12.009
16. Kahn KE, Santibanez TA, Zhai Y, Bridges CB. Association between provider recommendation and influenza vaccination status among children. *Vaccine.* 2018;36(24):3486-3497. https://doi.org/10.1016/j.vaccine.2018.04.077
17. Morhardt T, McCormack K, Cardenas V, Zank J, Wolff M, Burrows H. Vaccine curriculum to engage vaccine-hesitant families: didactics and communication techniques with simulated patient encounter. *MedEdPORTAL.* 2016;12:10400. https://doi.org/10.15766/mep_2374-8265.10400
18. Jacobson RM, Van Etta L, Bahta L. The C.A.S.E. approach: guidance for talking to vaccine-hesitant parents. *Minn Med.* 2013;96(4):49-50.
19. Real FJ, DeBlasio D, Beck AF, et al. A virtual reality curriculum for pediatric residents decreases rates of influenza vaccine refusal. *Acad Pediatr.* 2017;17(4):431-435. https://doi.org/10.1016/j.acap.2017.01.010
20. Shalansky RA, Wu M, Shen SC, et al. Evaluation of a pilot immunization curriculum to meet competency training needs of medical residents. *BMC Med Educ.* 2020;20:442. https://doi.org/10.1186/s12909-020-02349-1
21. Pahud B, Williams SE, Lee BR, et al. A randomized controlled trial of an online immunization curriculum. *Vaccine.* 2020;38(46): 7299-7307. https://doi.org/10.1016/j.vaccine.2020.09.043
22. Thomas PA, Kern DE, Hughes MT, Chen BY, eds. *Curriculum Development for Medical Education: A Six-Step Approach.* 3rd ed. Johns Hopkins University Press; 2016.
23. Loehr J, Savoy M. Strategies for addressing and overcoming vaccine hesitancy. *Am Fam Physician.* 2016;94(2):94-96.
24. McClure CC, Cataldi JR, ST O'Leary. Vaccine hesitancy: where we are and where we are going. *Clin Ther.* 2017;39(8):1550-1562. https://doi.org/10.1016/j.clinthera.2017.07.003

Copyright © 2022 Norton et al. This is an open-access publication distributed under the terms of the Creative Commons Attribution license.

25. Immunizations. American Academy of Pediatrics. Updated July 27, 2021. Accessed November 4, 2022. https://www.aap.org/en/patient-care/immunizations

26. Vaccines for your children. Centers for Disease Control and Prevention. Accessed November 4, 2022. https://www.cdc.gov/vaccines/parents/index.html

27. Vaccine Education Center. Children's Hospital of Philadelphia. Accessed November 4, 2022. https://www.chop.edu/centers-programs/vaccine-education-center

28. True random number generator. Random.org. Accessed November 4, 2022. https://www.random.org

29. Sadaf A, Richards JL, Glanz J, Salmon DA, Omer SB. A systematic review of interventions for reducing parental vaccine refusal and vaccine hesitancy. *Vaccine*. 2013;31(40):4293-4304. https://doi.org/10.1016/j.vaccine.2013.07.013

Received: April 28, 2022
Accepted: October 10, 2022
Published: December 27, 2022

Copyright © 2022 Norton et al. This is an open-access publication distributed under the terms of the Creative Commons Attribution license.
